# Approach Toward Stereoselective α‐Arylation by Pd/Cu‐Catalyzed Arylboration of Electron Deficient Alkenes

**DOI:** 10.1002/anie.202424073

**Published:** 2025-03-27

**Authors:** Suman Das, Maeve A. Reilly, Stanna K. Dorn, Allison M. Pearson, M. Kevin Brown

**Affiliations:** ^1^ Department of Chemistry Indiana University 800 E. Kirkwood Ave Bloomington 47401 Indiana

**Keywords:** Alkene, Boron, Carboboration, Copper, Palladium

## Abstract

Palladium‐catalyzed cross coupling of enolates—α‐arylation—is an established method for chemical synthesis. A major challenge in the field is control of stereochemistry for the α‐carbon. This is typically due to facile epimerization under the basic reaction conditions for α‐arylation. In this study, an alternative approach is presented that involves the Pd/Cu‐catalyzed arylboration of electron deficient alkenes. The products are generated with high levels of diastereoselectivity for a broad range of substitution patterns. Enantioselective variants are also presented in addition to product derivatizations.

## Graphical abstract

A strategy for the α‐arylation of carbonyl compounds is presented. The Cu‐ and Pd‐catalyzed reaction involves 1,2‐arylboration of electron deficient alkenes and proceeds with high levels of selectivity. The mechanism is addressed and suggests that a boron ligated enolate is responsible for high selectivity.

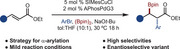

Cross‐coupling reactions have transformed the chemical synthesis landscape.^[^ ^1^, ^2^, ^3^, ^4^, ^5^, ^6^, ^7^ ^]^ Of the many classes of cross‐coupling reactions, Pd‐catalyzed α‐arylation of enolates has emerged as a powerful method.^[^ ^8^, ^9^, ^10^, ^11^, ^12^, ^13^ ^]^ This reaction combines aryl halides and a variety of carbonyl compounds to generate C─C bonds. The arylated carbonyl compounds have proved to be valuable intermediates in chemical synthesis. Despite immense progress in this area, one challenge that remains is the control of stereochemistry. The conditions for α‐arylation typically require strong base and, at times, elevated temperatures. This often results in in‐situ epimerization or racemization of the arylated carbonyl compound. Therefore, stereoselective Pd‐catalyzed α‐arylation typically involves the synthesis of quaternary carbons.^[^ ^14^, ^15^, ^16^, ^17^, ^18^, ^19^, ^20^, ^21^, ^22^, ^23^ ^]^

In recent years, alkene carboboration has emerged as an important class of reactions as simple components can be employed to rapidly assemble complex products.^[^ ^24^, ^25^, ^26^, ^27^, ^28^, ^29^, ^30^ ^]^ Of the many variants reported, Cu/Pd‐catalyzed arylboration is noteworthy as a variety of alkenes can undergo reaction.^[^ ^31^, ^32^, ^33^, ^34^, ^35^, ^36^, ^37^, ^38^, ^39^, ^40^, ^41^, ^42^, ^43^, ^44^, ^45^, ^46^, ^47^, ^48^, ^49^, ^50^, ^51^, ^52^, ^53^, ^54^, ^55^, ^56^, ^57^, ^58^, ^59^, ^60^, ^61^ ^]^ However, the translation of these methods to reactions of electron‐deficient alkenes is not well established.^[^ ^62^, ^63^, ^64^, ^65^, ^66^, ^67^, ^68^, ^69^, ^70^, ^71^, ^72^ ^]^ This is significant as the product is that of an α‐arylation of a carbonyl. In addition, due to the presence of the Bpin unit and, more importantly, mild reaction conditions, control of stereochemistry is likely facilitated.^[^ ^73^ ^]^ In only one example, Semba and Nakao report an alkene arylboration of β‐methyl crotonate in moderate yield and diastereoselectivity.^[^ ^46^ ^]^ In this manuscript, we present a generally effective method for the stereoselective arylboration of a variety of electron deficient alkenes.

Initial studies focused on the arylboration of ethyl cinnamate (**1**). Under standard reaction conditions previously identified (SIMesCuCl, RuPhosPdG3), the product was formed in low yield and moderate diastereomeric ration (DR) (Scheme 2a, Entry 1).^[^ ^31^, ^32^, ^33^, ^34^, ^35^, ^36^, ^37^, ^38^, ^39^, ^40^, ^41^, ^42^, ^43^ ^]^ Evaluation of other ligands for palladium revealed that larger ligands led to improved dr (compare Scheme 1a Entries 3 and 5). From this observation, APhos was evaluated and found to be uniquely effective for delivering the desired product in good yield and excellent selectivity as determined by GC analysis (Scheme 2a, Entries 8). The Cu‐catalyst was also varied; however, no further improvement was noted (Scheme 2a, Entries 9–11).

**Scheme 1:**
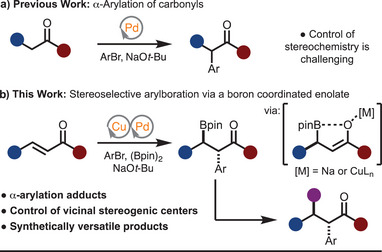
Approaches toward α‐arylation.

**Scheme 2:**
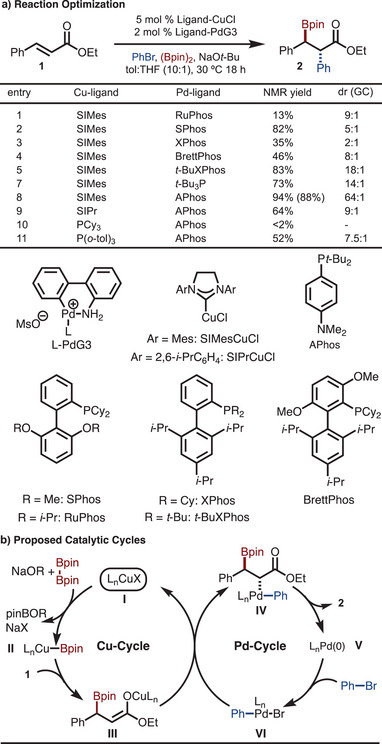
Reaction optimization and catalytic cycle. Yield of isolated and purified product in parentheses.

Under the optimized reaction conditions, a range of substrates were evaluated. First, *Z*‐ethyl cinnamate was investigated, which gave rise to the same diastereomer, confirming the stereoconvergent nature of this reaction. With respect to the arylbromide component, electron‐rich (Product **3**), electron‐deficient (Product **6**), and sterically‐demanding (Product **7**) substrates all functioned well. Various heterocycles such as indole (Product **10**), difluorodioxy (Product **11**), and various pyridines (Products **12** and **13**) led to formation of the products in good yield and diastereoselectivity. Finally, although alkenyl groups could be tolerated (Products **8** and **9**), lower levels of selectivity were observed.

Various cinnamate derivatives were also examined. Although the diastereoselectivity fluctuated as a function of the aryl group electronics with no clear trend, substrates with electron‐releasing (Product **14**) and accepting substituents (Product **15**) were all tolerated. However, sterically demanding substrates were less selective (Products **18** and **19**). In addition to arenes, simple heteroarenes such as thiophene (Product **21**) and furan (Product **22**) worked well. Alkyl substitution was also tolerated and the diastereoselectivity increased with larger substituents (compared Products **23–25**). The reaction functioned well with β,β‐disubstitution (Products **26** and **27**). When the substituents were different sizes, the selectivity was moderate (Product **28**).

The products illustrated in Scheme 3 are racemic; therefore, efforts were directed toward the development of an enantioselective variant (Scheme 4). Based on prior work from our lab,^[^ ^31^, ^32^, ^33^, ^34^, ^35^, ^36^, ^37^, ^38^, ^39^, ^40^, ^41^, ^42^, ^43^ ^]^ we targeted the use of the McQuade chiral NHC–Cu complex (**30**).^[^ ^74^, ^75^ ^]^ Under standard conditions with (Bpin)_2_, the desired product was generated in good yield and diastereoselectivity, but in 77:23 enantiomeric ratio (ER). To improve the enantioselectivity, we looked toward examining other diboron reagents. Initially, the more sterically demanding (BEpin)_2_ was examined; however, the reactivity and selectivity was similar to (Bpin)_2_. Next, both enantiomers of commercially available chiral diboron (Bpai)_2_ were investigated. Here a matched/mismatched effect was observed, with the (*R*,*R*,*R*,*S*)‐derived reagent giving rise to improved er (90:10 er) relative to the (*S,S,S,R*)‐derived reagent (75:25 er). *Z*‐ethyl cinnamate was also tested under the optimal conditions; however, lower levels of enantioselectivity were observed (65% yield, 63:37 er, 15:1 dr, same major enantiomer and diastereomer and reaction with *E*‐ethyl cinnamate, see the Supporting Information for details). Under these conditions, several other substrates were examined. First, an electron rich and electron poor arylbromide were tested and good levels of enantioselectivity were observed (Product **31** and **32**). However, use of 2‐bromo‐propene was less selective (Product **33**). With sterically demanding 2‐Me or 2‐OMe substituted aryl groups, improved enantioselectivity was observed relative to phenyl (Products **34** and **35**). Although electron rich (Product **36**) and electron poor (Product **37**) aryl groups functioned well, the sterically smaller furan gave rise to lower levels of enantioselectivity (Product **38**). Finally, reactions of alkyl substituted alkenes led to formation of Product **39–41** in good yield and enantioselectivities.

**Scheme 3:**
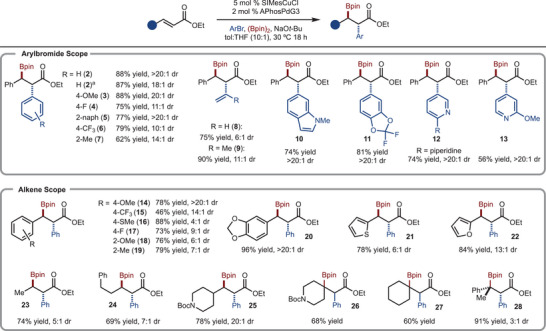
Substrate Scope. ^a)^From reaction of *Z*‐ethyl cinnamate.

**Scheme 4:**
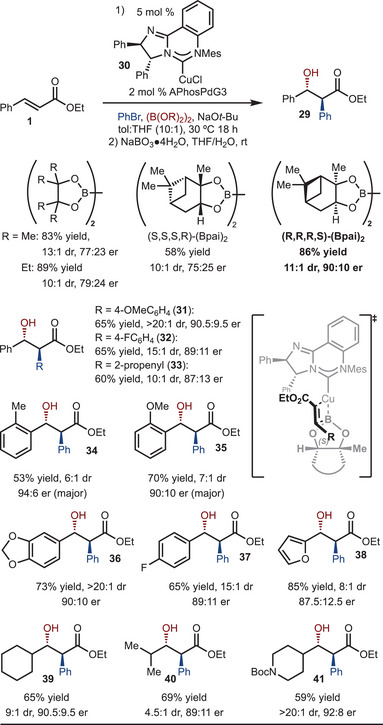
Enantioselective reaction. Substrates oxidized to facilitate analysis by HPLC with a chiral column.

Based on prior work, a model for selectivity is proposed in Scheme 4.^[^ ^31^, ^32^, ^33^, ^34^, ^35^, ^36^, ^37^, ^38^, ^39^, ^40^, ^41^, ^42^, ^43^, ^74^, ^75^ ^]^ Migratory insertion with the alkene likely occurs on the face opposite to the proximal phenyl group of the diamine component. Upon initial inspection, the Me group of the pinane subunit appears to have an adverse interaction with the β‐substituent of the alkene. However, it is suspected that the C─B rotates to position the adjacent H closer to the β‐substituent and facilitates alkene approach.

To demonstrate practicality, the reaction was conducted on a 5.0 mmol scale with little change in yield or selectivity (Scheme 5a). The product could be utilized in several transformations. First, oxidation of the C─B bond and subsequent reduction of the ester led to diol **42**. Treatment with 2,2‐dimethoxypropane led to formation of **43**, which allowed for confirmation of relative stereochemistry through comparison with known compounds.^[^ ^76^ ^]^ In addition, the C─B bond could be converted to various C─C bonds by coupling with furan (Product **44**),^[^ ^77^ ^]^ Matteson homologation (Product **45**),^[^ ^78^ ^]^ and Zweifel alkenylation (Product **46**) (Scheme 5b).^[^ ^79^ ^]^

**Scheme 5:**
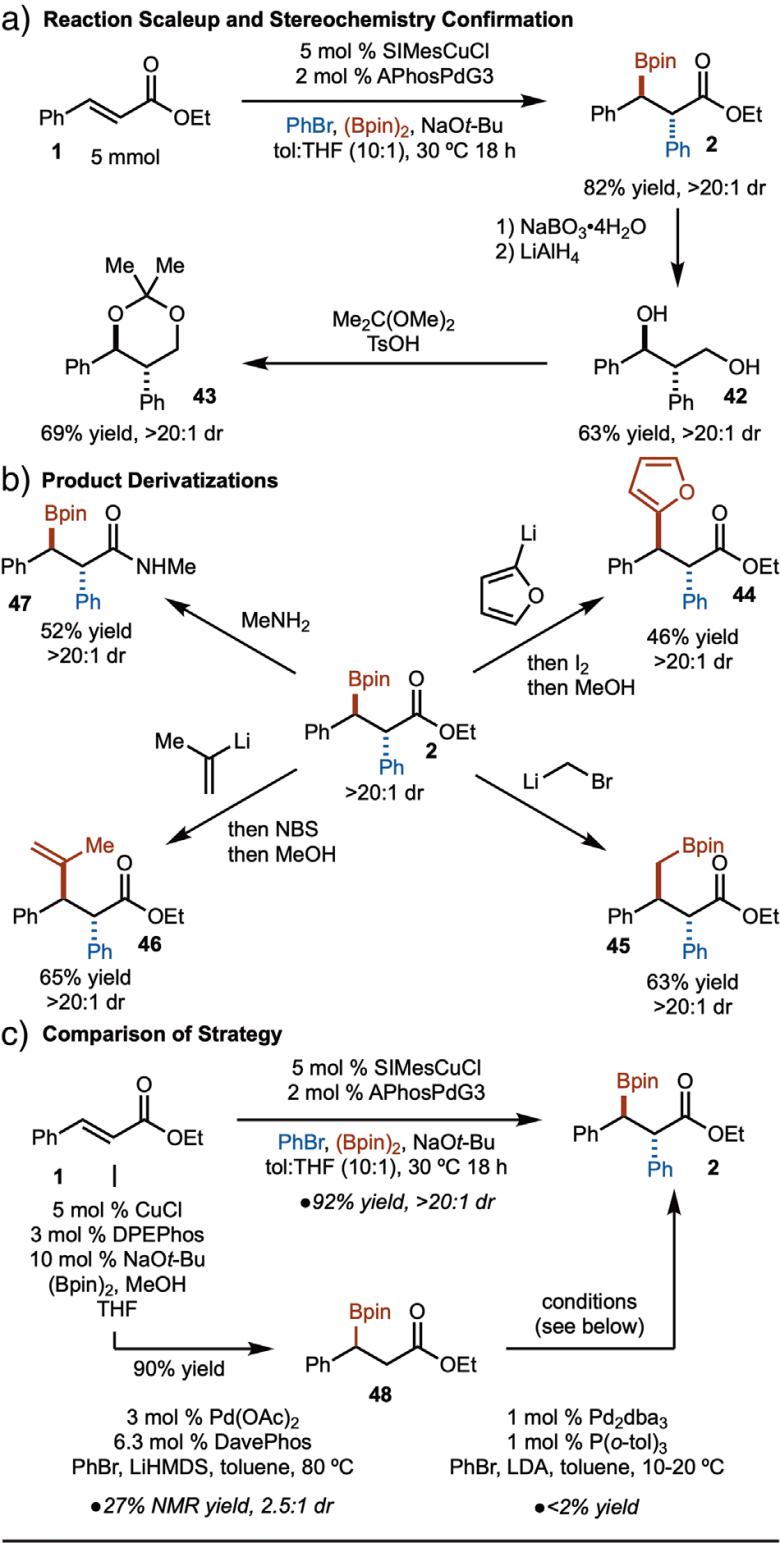
Further studies.

Finally, a comparison of strategies was conducted (Scheme 5c). As outlined, **2** can be generated in 92% yield and >20:1 dr from ethyl cinnamate in one step. However, an alternative two‐step pathway could involve borylation followed by α‐arylation. The borylation chemistry is well precedented on ethyl cinnamate (**1**); however, the α‐arylation of **48** is not known. In accordance with literature procedures,^[^ ^80^ ^]^ **48** was generated in 90% yield. Pd‐catalyzed α‐arylation was attempted under two standard sets of conditions;^[^ ^81^, ^82^ ^]^ however, in the best case, low yield and selectivity was observed for formation of **2**. This further underscores the value of the arylboration approach presented herein.

Finally, the mechanism of the reaction was investigated (Scheme 6). Based on prior studies, the reaction likely operates by the general catalytic cycles illustrated in Scheme 2b.^[^ ^31^, ^32^, ^33^, ^34^, ^35^, ^36^, ^37^, ^38^, ^39^, ^40^, ^41^, ^42^, ^43^, ^44^, ^45^, ^46^, ^47^, ^48^, ^49^, ^50^, ^51^, ^52^, ^53^, ^54^, ^55^, ^56^, ^57^, ^58^, ^59^, ^60^, ^61^ ^]^ However, the basis for the observed diastereoselectivity was not known. Based on prior work,^[^ ^62^, ^63^, ^64^, ^65^, ^66^, ^67^, ^68^, ^69^, ^70^, ^71^, ^72^ ^]^ a chelate may form between the Lewis basic oxygen and the Lewis acidic boron (Scheme 6a). This allows for rigidification of the enolate and therefore allows for approach of the large APhos–Pd complex from the face opposite the Ph group (intermediate **49**).^[^ ^83^ ^]^ It is important to note that a rapid reductive elimination is likely necessary, which is facilitated by the large ligand, so as to avoid epimerization via a Pd–enolate complex. A data point that supports the proposed chelate is illustrated in Scheme 6b. Reaction of the related cyano‐derived substrate **50** led to formation of product **52** in low dr. Here, the proposed chelate is not geometrically feasible and thus low selectivity was observed. However, the potential for reaction via C‐bound Cu–enolate complicates full interpretation of these results.

**Scheme 6:**
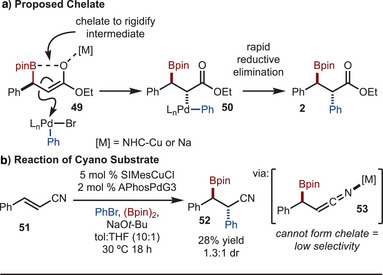
Mechanism studies.

In conclusion, a Cu/Pd‐catalyzed method for the arylboration of electron‐deficient alkenes is presented. The reaction allowed for control of stereochemistry for both the C─B and C─Ar bonds. Enantioselective variants of the reaction are presented, along with demonstrated utility of the products. Finally, the methodology provides a solution to the challenges experienced in controlling selectivity in α‐arylation reactions.

## Conflict of Interests

The authors declare no conflict of interest.

## Supporting information



Supporting Information

## Data Availability

The data that support the findings of this study are available in the Supporting Information of this article.
