# Supplementary material for: Approach Toward Stereoselective α‐Arylation by Pd/Cu‐Catalyzed Arylboration of Electron Deficient Alkenes
Source: Angew Chem Int Ed Engl. 2025 Mar 27;64(22):e202424073. doi: 10.1002/anie.202424073 (PMC12105705; doi:10.1002/anie.202424073)
Supplement: Supplementary file 1 — Supporting Information [file ANIE-64-e202424073-s001.pdf]

Approach Towards Stereoselective α-Arylation by Pd/Cu-Catalyzed Arylboration of Electron Deficient Alkenes

Suman Das, Maeve Reilly, Stanna K. Dorn, Alison M. Pearson, and M. Kevin Brown*

† Department of Chemistry, Indiana University, 800 E. Kirkwood Ave, Bloomington, IN 47405, United States

Table of Contents

- General information S-2
- Reagents S-2
- Synthesis of alkene substrates and chiral Cu-catalyst S-4
- General procedures for Cu/Pd-catalyzed Arylboration reaction S-7
- Optimization of the reaction S-10
- Experimental characterization data S-12
- Control experiments S-36
- Scale-up synthesis and further functionalization S-38
- Determination of relative and absolute configuration of product S-43
- NMR data S-46
- HPLC data S-121
- Reference S-145

1. General Information:

Infrared (IR) spectra were recorded on Bruker Tensor II FT-IR Spectrometer, n_{max} in cm-1. Bands are characterized as broad (br), strong (s) for >70% transmittance, medium (m) for 40-70% transmittance, and weak (w) for <40% transmittance. ^{1}H NMR spectra were recorded at room temperature on a Varian I400 (400 MHz), Varian VXR400 (400 MHz), Varian I500 (500 MHz), a Varian I600 (600 MHz) spectrometer, and/or a Bruker Ascend™ 500 MHz (equipped with cryoprobe). Chemical shifts are reported in ppm from tetramethyl silane with the solvent resonance as the internal standard ($CHCl_3$: λ 7.26 ppm). Data are reported as follows: chemical shift, multiplicity (s = singlet, d = doublet, t = triplet, q = quartet, br = broad, m = multiplet, app. = apparent), coupling constants (Hz), and integration. ^{13}C NMR spectra were recorded on a Varian I400 (100 MHz), Varian I500 (125 MHz), and/or a Bruker Ascend™ 500 MHz (125 MHz, equipped with cryoprobe) spectrometer with complete proton decoupling. Chemical shifts are reported in ppm from tetramethyl silane with the solvent resonance as the internal standard ($CDCl_3$: δ77.16 ppm). Unless otherwise noted, all reactions have been carried out with distilled and degassed solvents under an atmosphere of dry N_2 in oven- (135 °C) and flame-dried glassware with standard vacuum-line techniques. Tetrahydrofuran (THF) was purified under a positive pressure of dry argon by passage through two columns of activated alumina. Toluene was purified under a positive pressure of dry argon by passage through columns of activated alumina and Q5 (Grubbs apparatus). All work-up and purification procedures were carried out with reagent grade solvents (purchased from Sigma-Aldrich) in air. Standard column chromatography techniques were carried out using ZEO prep 60/40-63 µm silica gel. For samples that were unstable on silica, purification was done using neutral aluminum oxide (activated, Brockmann I 58 Å pore size, Oakwood). For difficult separations, medium-pressure liquid chromatography (MPLC) was performed using a Teledyne ISCO Combi Flash Rf 150 instrument. Optical rotations were measured on a PerkinElmer 241 polarimeter at 589 nm wavelength (sodium D-line) using a standard 10 cm cell (1 mL). Specific rotations, $[\alpha]_D^{20}$, are reported in degree mL/(g·dm) at the specific temperature. Concentrations (c) are given in grams per 100 mL of the specific solvent. Chiral HPLC analysis was performed on an Agilent 1220 Infinity LC system.

2. Reagents:

***(E)*-Ethyl cinnamate** was purchased from Sigma Aldrich and used as received.
Methyl cinnamate was purchased from Sigma Aldrich and used as received.
Chalcone was purchased from Sigma Aldrich and used as received.
APhos was purchased from Strem and used as received.
3-Phenylacrylonitrile was was purchased from Ambeed and purified by neat filtration through a 2 cm (about 0.79 in) pad of dry silica in a 5.75-inch pipette onto activated 4Å MS prior to use.
APhosPdG$_3$ was prepared according to the literature.[1]
CPhos was purchased from Strem and used as received.
tBuXPhos was purchased from Strem and used as received.

RuPhos was purchased from Sigma Aldrich and used as received.
CyAPhos was purchased from Sigma Aldrich and used as received.
XPhos was purchased from Sigma Aldrich and used as received.
CyJohnPhos was purchased from Sigma Aldrich and used as received.
PCy_3 was purchased from Strem and used as received.
SPhos was purchased from Sigma Aldrich and used as received.
BrettPhos was purchased from Sigma Aldrich and used as received.
Bis(pinacolato)diboron (B_2pin_2) was purchased from Oakwood and purified by recrystallization in pentane prior to use.
Bromobenzene was purchased from Sigma Aldrich and purified by neat filtration through a 2 cm (about 0.79 in) pad of dry silica in a 5.75-inch pipette onto activated 4Å MS prior to use.
6-bromoindole was purchased from CombiBlocks and used as received.
5-bromo-2,2-difluorobenzodioxole was purchased from Sigma Aldrich and purified by neat filtration through a 2 cm (about 0.79 in) pad of dry silica in a 5.75-inch pipette onto activated 4Å MS prior to use.
2-bromonapthalene was purchased from ambeed and purified by neat filtration through a 2 cm (about 0.79 in) pad of dry silica in a 5.75-inch pipette onto activated 4Å MS prior to use.
4-bromo-2-methoxypyridine was purchased from TCI and purified by neat filtration through a 2 cm (about 0.79 in) pad of dry silica in a 5.75-inch pipette onto activated 4Å MS prior to use.
Vinyl bromide was purchased from Sigma Aldrich and used as received.
2-bromo-propene was purchased from Oakwood and used as received.
Methyl trans cinnamate was purchased from CombiBlocks and purified by neat filtration through a 2 cm (about 0.79 in) pad of dry silica in a 5.75-inch pipette onto activated 4Å MS prior to use.
E-Chalcone was purchased from ambeed and used as received.
5-bromo-1-methyl-1H-pyrrolo[2,3-b] pyridine was prepared according to the literature.[2]
N-Bromosuccinimide was purchased from Oakwood and was purified by recrystallization in H_2O prior to use.
Copper (I) chloride was purchased from STREM and purified by trituration twice with aqueous HCl, twice with EtOH, and twice with Et_2O in that order before drying under vacuum overnight.
Furan was purchased from Sigma Aldrich and used as received.
30% H_2O_2 in H_2O was purchased from Macron and used as received.
SIMes-CuCl was prepared according to the literature.[2]
McQuade-CuCl was prepared according to the literature.[3]
tBu-McQuade-CuCl was prepared according to the literature.[4]
Iodine was purchased from Alfa Aesar and used as received.
Methanol Anhydrous was purchased from Neta Scientific and used as used as received.
n-butyl lithium solution (2.5 M in hexanes) was purchased from Sigma Aldrich and used as received.
PCy_3-PdG$_3$ was prepared according to the literature.[5]
Potassium carbonate was purchased from EMD and dried in an oven at 80 oC overnight before use.
RuPhos-PdG$_3$ was prepared according to the literature.[1]
Sodium tert-butoxide was purchased from Strem and used as received.
tert-butyllithium solution (1.7M in pentane) was purchased from Sigma Aldrich and used as received.

Tricyclohexylphosphine (PCy_3) was purchased from Strem and used as received.
Triethylamine was purchased from EMD and purified by distillation over calcium hydride before use.
XPhos-PdG3 was prepared according to the literature. [5]
$MeNH_2$ (2.0 M in MeOH) was purchased from TCI and used as received.
Palladium (II) Acetate was purchased from Sigma Aldrich and used as received.
DavePhos was purchased from alfa aesar and used as received.
P*t*Bu3 was purchased from Sigma-Aldrich and used as received.
PAd2*n*Bu was purchased from Sigma-Aldrich and used as received.

3. **General Procedure for the synthesis of alkene substrates:**

The substrates were synthesized according to the literature reported procedure with some modifications:

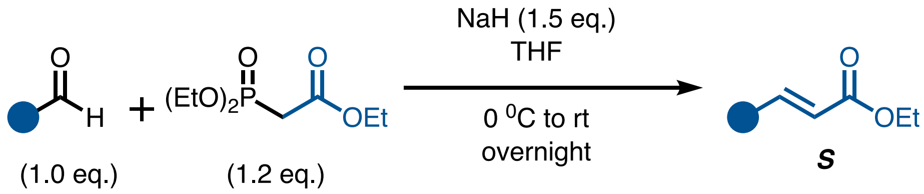

Sodium hydride (360 mg, 1.5 eq., 15 mmol) was dissolved in THF (47 mL), and triethyl phosphonoacetate (1.34 g, 1.2 mL, 1.2 eq., 6.0 mmol) was added dropwise to the suspension at 0 °C. The mixture was stirred until gas evolution had ceased. Then, the aldehyde or ketone (1.0 eq., 10 mmol) in THF (3.0 mL) was added by syringe. The reaction was stirred at room temperature and monitored by TLC. The reaction mixture was quenched with saturated aqueous NH_4Cl solution. The organic phase was separated, and the aqueous layer was extracted with EtOAc. The combined organic phases were washed with saturated aqueous NaCl solution, dried over anhydrous sodium sulfate, and concentrated under vacuum pressure. Purification by silica gel chromatography (Hexane: EtOAC=40:1 to 10:1) gave the alkene as an oil or solid. The product NMR were matched with following literature data

S-1& S-2[6]; S-3[7]; S-4[8]; S-5[9]; S-6[10], S-7[11], S-8 &S-12[12], S-9[13], S-10[14], S-11[15], S-13[16], S-14[17], S-15[18]

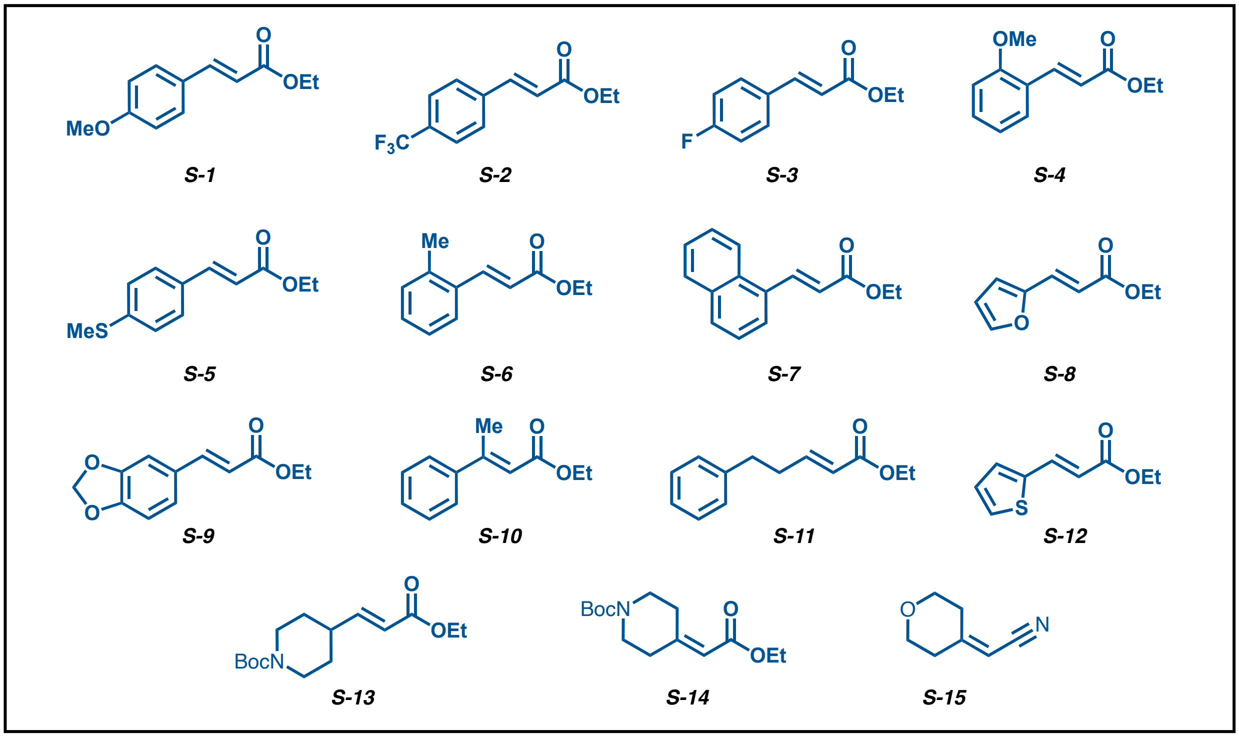

Aryl bromide from Commercial source:

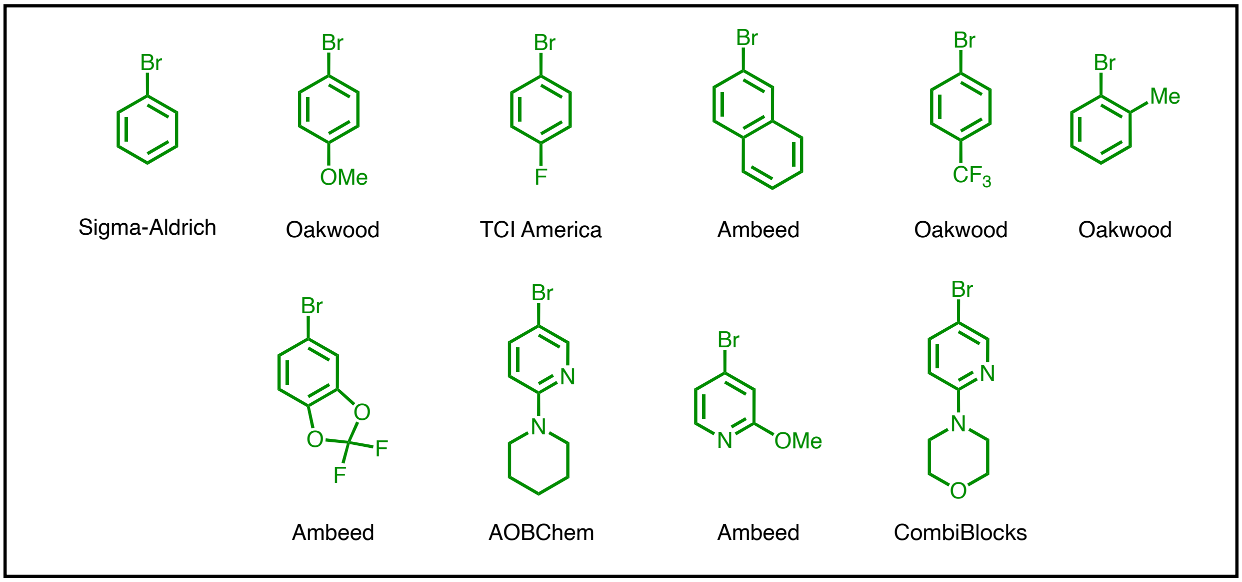

6-bromo-1-methyl-1H-indole synthesized and characterized based on literature report[19]

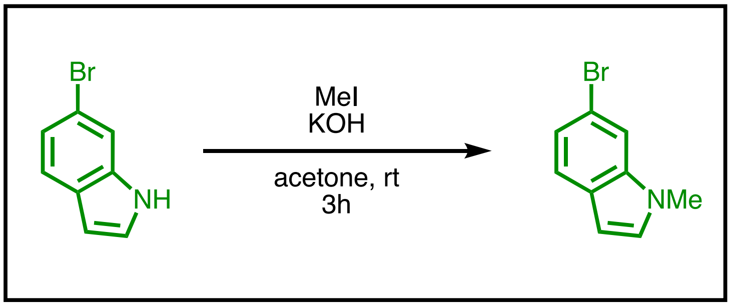

Synthesis of Chiral Cu-McQuade complex

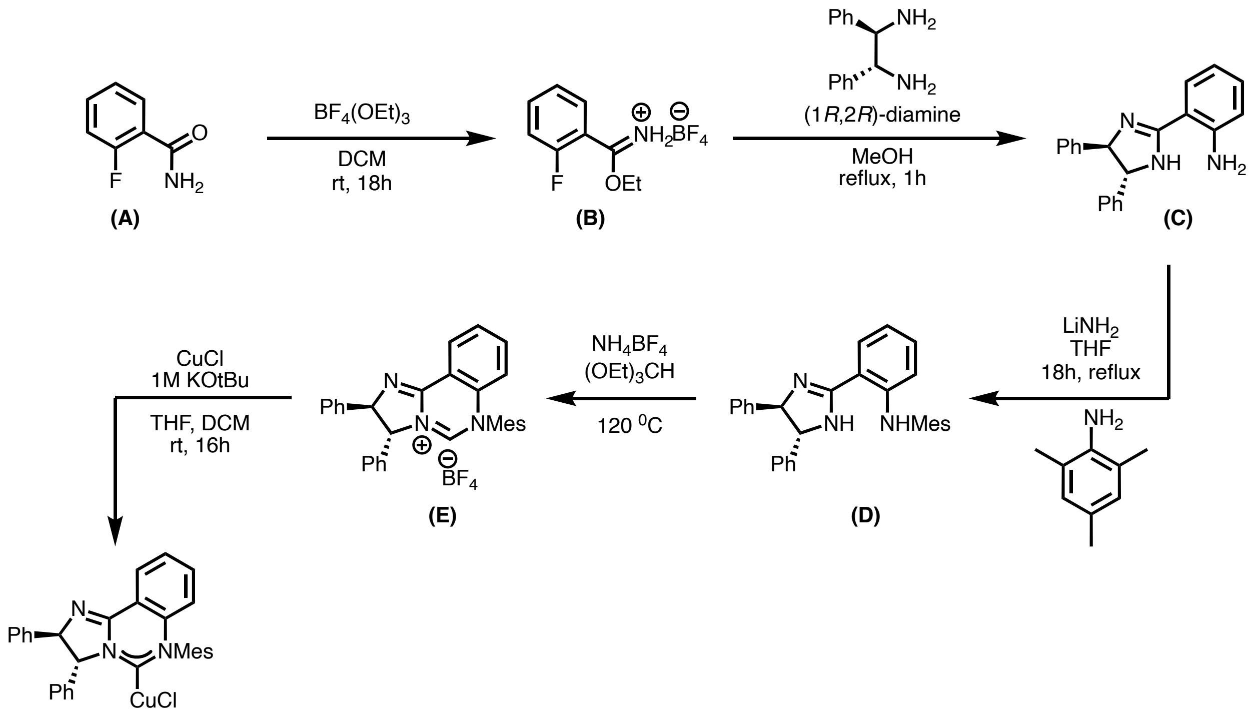

Chiral Cu-McQuade complex made via literature producer: Compound **(B)** and **(C)** made using literature procedure using (*R,R*)-1,2-diphenylethane-1,2-diamine[20]
Compound **(D)** and **(E)** made using literature procedure[21]
Compound **(30)** made using literature procedure[22]
All characterization data matched with the corresponding reported literature data.

4(a). General Procedure A: The Arylboration of electron-deficient alkenes

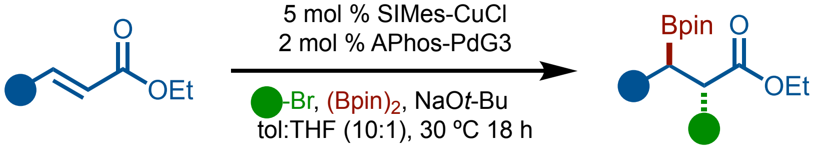

In an N_2-filled glovebox, to a flame-dried 16 × 100 mm screw cap vial with a magnetic stir bar, was added APhosPdG3 (3.2 mg, 5.0 µmol, 2.0 mol%), SIMesCuCl (5.1 mg, 13 µmol, 5.0 mol%), B_2pin_2 (95 mg, 0.38 mmol, 1.5 eq.), NaOtBu (31 mg, 0.33 mmol, 1.3 eq.) was added, ***(Note: if your alkene and aryl bromide are solid then both of them were taken inside glovebox; aryl bromide (0.38 mmol, 1.5 eq. if it is a solid) and alkene (0.25 mmol, 1.0 eq.)*** in that order. Then the vial was sealed with a rubber septum and lined with Teflon tape, removed from the glove box, and placed under a positive pressure of N_2. To the vial was added 1.5 mL of toluene: THF (10:1), the alkene (0.25 mmol, 1.0 eq.), and the aryl bromide (0.38 mmol, 1.5 eq. if it is a liquid) before rinsing the sides of the vial with another 1.0 mL of toluene: THF (10:1). *(Note: it is important that the vial is stirring vigorously while the liquid reagents are added to prohibit aggregation of the solid reagents, which typically results in lower yields)*. The septum was then quickly replaced with a Teflon lined screw cap and the reaction was stirred at 30°C for 18 hours. After 18 hours, the reaction was quenched with aqueous water (2.5 mL), the two phases were separated, and the aqueous phase was back extracted with EtOAc (2 × 3 mL). The combined organic phases were dried with anhydrous Sodium sulfate and concentrated in-vacuo. The organic residue further purified by column chromatography.

4(b). General Procedure B: The Arylboration of electron-deficient alkenes without glovebox setup

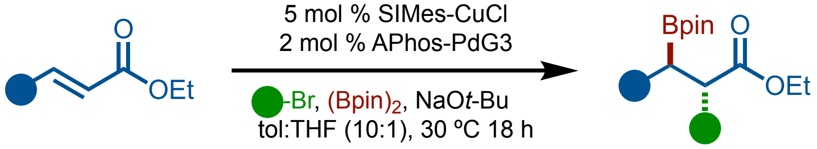

In a flame-dried 16 × 100 mm screw cap vial with a magnetic stir bar, was added APhosPdG3 (3.2 mg, 5.0 µmol, 2.0 mol%), SIMesCuCl (5.1 mg, 13 µmol, 5.0 mol%), B_2pin_2 (95 mg, 0.38 mmol, 1.5 eq.), NaOtBu (31 mg, 0.33 mmol, 1.3 eq.) were added ***(Note: if your alkene and aryl bromide are solid then both of them was taken along with these reagents; aryl bromide (0.380 mmol, 1.5 eq, if it is a solid) and alkene (0.25 mmol, 1.0 eq***. Then the vial was put under a N_2 atmosphere via standard Schlenk technique evacuated and backfilled with N_2 three times. The vial was removed from the Schlenk line and quickly capped with a septum and a N_2 line. 1.5 mL of Toluene:

THF (10:1) solution was added via micro syringe, aryl bromide (0.38 mmol, 1.5 eq.) was immediately added via syringe followed by alkene (0.25 mmol, 1.0 eq.) via micro syringe. *(Note: it is important that the vial is stirring vigorously while the liquid reagents are added to prohibit aggregation of the solid reagents, which typically results in lower yields).* The septum was then quickly replaced with a Teflon lined screw cap and the reaction was stirred at 30°C for 18 hours. The mixture was allowed to stir for 18 h at 30 ^{0}C. After 18 hours, the reaction was quenched with aqueous water (2.5 mL), the two phases were separated, and the aqueous phase was back extracted with EtOAc (2 × 3 mL). The combined organic phases were dried with anhydrous Sodium sulfate and concentrated in-vacuo. The organic residue further purified by column chromatography.

4(c). General Procedure C: Enantioselective Arylboration of electron-deficient alkenes using Chiral Cu-McQuade catalyst and (*R,R,R,S*)-B_2(pai)$_2$

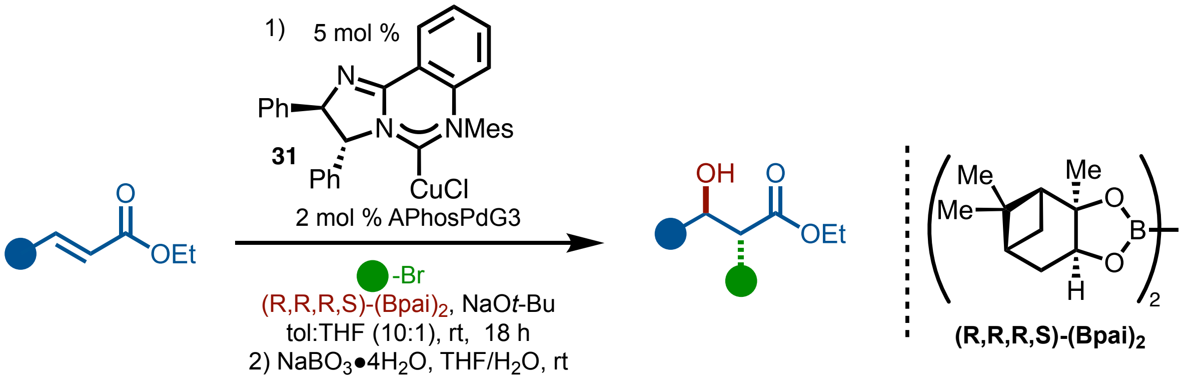

In an N_2-filled glovebox, to a flame-dried 13 × 100 mm screw cap vial with a magnetic stir bar, was added APhosPdG3 (1.27 mg, 2.0 µmol, 2.0 mol%), McQuade-CuCl (2.7 mg, 5.0 µmol, 5.0 mol%), (*R,R,R,S*)-B_2(pai)$_2$ (56 mg, 0.15 mmol, 1.5 eq.), NaOtBu (12.5 mg, 0.13 mmol, 1.3 eq.) was added, ***(Note: if your alkene and aryl bromide are solid then both of them were taken inside glovebox; aryl bromide (0.380 mmol, 1.5 eq. if it is a solid) and alkene (0.25 mmol, 1.0 eq.)*** in that order. Then the vial was sealed with a rubber septum and lined with Teflon tape, removed from the glove box, and placed under a positive pressure of N_2. To the vial was added 0.5 mL toluene: THF (10:1), the alkene (0.1 mmol, 1.0 eq.), and the aryl bromide (0.15 mmol, 1.50 eq., if it is a liquid) before rinsing the sides of the vial with another 0.5 mL toluene: THF (10:1). *(Note: it is important that the vial is stirring vigorously while the liquid reagents are added to prohibit aggregation of the solid reagents, which typically results in lower yields).* The septum was then quickly replaced with a Teflon lined screw cap and the reaction was stirred at room temperature for 18 hours. After 18 hours, the reaction was quenched with aqueous water (1.0 mL), the two phases were separated, and the aqueous phase was back extracted with EtOAc (1.0 × 3 mL). The combined organic phases were dried with anhydrous Sodium sulfate and concentrated in-vacuo. The organic residue was further used for checking NMR yield and dr. After that crude directly subjected to oxidation of C-B bond.

The arylboration product (0.2 mmol) was taken in a 10 mL round-bottom flask, dissolved in THF (1.0 mL) and $NaBO_3 \cdot 4H_2O$ (90.0 mg, 0.58 mmol, 3.0 eq.) was added, followed by addition of

water (1.0 mL). The reaction was allowed to stir at room temperature overnight. Then the reaction mixture was quenched upon the addition of aqueous saturated $Na_2S_2O_3$ (3 mL) and extracted with EtOAc (3 x 2 mL). The combined organic layers were washed with 2 M HCl (3 x 3 mL), dried over anhydrous sodium sulfate, and concentrated in vacuo. The residue was purified via silica gel column chromatography

4(d). General Procedure D: The Arylboration of electron-deficient alkenes

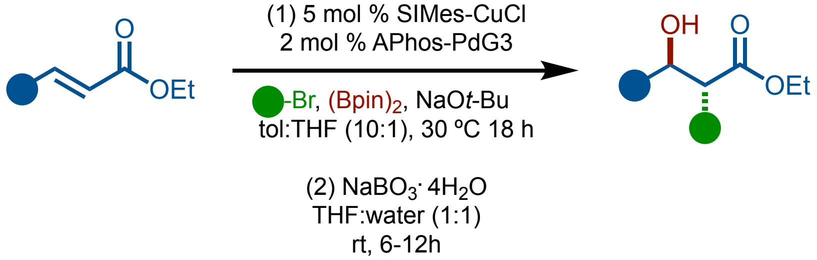

In an N_2-filled glovebox, to a flame-dried 16 × 100 mm screw cap vial with a magnetic stir bar, was added APhosPdG3 (3.2 mg, 5.0 µmol, 2.0 mol%), SIMesCuCl (5.1 mg, 13 µmol, 5.0 mol%), B_2pin_2 (95 mg, 0.38 mmol, 1.5 eq.), NaOtBu (31 mg, 0.33 mmol, 1.3 eq.) was added, ***(Note: if your alkene and aryl bromide are solid then both of them were taken inside glovebox; aryl bromide (0.38 mmol, 1.5 eq. if it is a solid) and alkene (0.25 mmol, 1.0 eq.)*** in that order. Then the vial was sealed with a rubber septum and lined with Teflon tape, removed from the glove box, and placed under a positive pressure of N_2. To the vial was added 1.5 mL of toluene: THF (10:1), the alkene (0.25 mmol, 1.0 eq.), and the aryl bromide (0.38 mmol, 1.5 eq. if it is a liquid) before rinsing the sides of the vial with another 1.0 mL of toluene: THF (10:1). *(Note: it is important that the vial is stirring vigorously while the liquid reagents are added to prohibit aggregation of the solid reagents, which typically results in lower yields).* The septum was then quickly replaced with a Teflon lined screw cap and the reaction was stirred at 30°C for 18 hours. After 18 hours, the reaction was quenched with aqueous water (2.5 mL), the two phases were separated, and the aqueous phase was back extracted with EtOAc (2 × 3 mL). The combined organic phases were dried with anhydrous Sodium sulfate and concentrated in-vacuo. The organic residue then taken in a 16 × 100 mm screw cap vial was dissolved in THF (1.0 mL) and NaBO3·4H2O (90 mg, 0.58 mmol, 3.0 eq.) was added, followed by addition of H_2O (1.0 mL). The reaction was allowed to stir at room temperature overnight until complete conversion was observed by TLC analysis then was quenched upon the addition of aqueous saturated $Na_2S_2O_3$ (3.0 mL) and extracted with Et_2O (3 x 2 mL). The combined organic layers were washed with 2.0 M HCl (3 x 3 mL), dried over Na_2SO_4, gravity filtered, and concentrated in vacuo. The residue was purified via silica gel chromatography (hexanes/EtOAc) to afford the desired product.

5. (a) Optimization of the reaction conditions: Racemic reactions

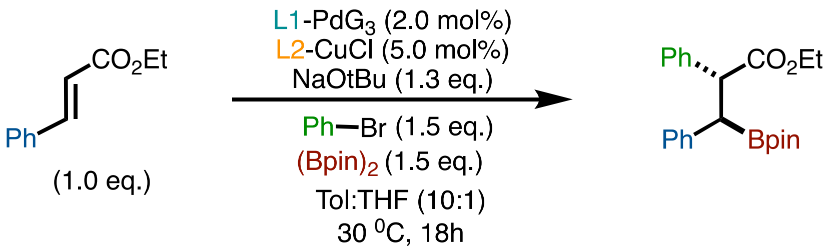

entry	L_1	L_2	yield[a]	dr[b]
1	**APhos**	**SIMes**	**94%**	**64:1**
2	CPhos	SIMes	31%	4:1
3	tBuXPhos	SIMes	83%	18:1
4	RuPhos	SIMes	13%	9:1
5	CyAPhos	SIMes	<5%	-
6	XPhos	SIMes	35%	2:1
7	CyJohnPhos	SIMes	39%	4:1
8	PCy_3	SIMes	<5%	-
9	SPhos	SIMes	82%	5:1
10	BrettPhos	SIMes	46%	8:1
11	$Pt\mathrm{Bu}_3$	SIMes	73%	14:1
12	$PAd_2n\mathrm{Bu}$	SIMes	20%	9:1
13	APhos	SIPr	64%	9:1
14	APhos	IPr	79%	11.5:1
15	APhos	IMes	91%	18.5:1
16	APhos	Mes-Pyr	82%	8.5:1
17	APhos	XantPhos	18%	3:1
18[c]	APhos	PCy_3	<5%	-
19[c]	APhos	(R)-SegPhos	<5%	-
20	APhos	$P(O\text{-tol})_3$	52%	7.5:1

[a] yields were determined by analyzing the ^{1}HNMR of crude reaction mixture using CH_2Br_2 as internal standard.
[b] dr were determined by analyzing the ^{1}HNMR of crude reaction mixture using CH_2Br_2 as internal standard.
[c] protoboration was the major product.

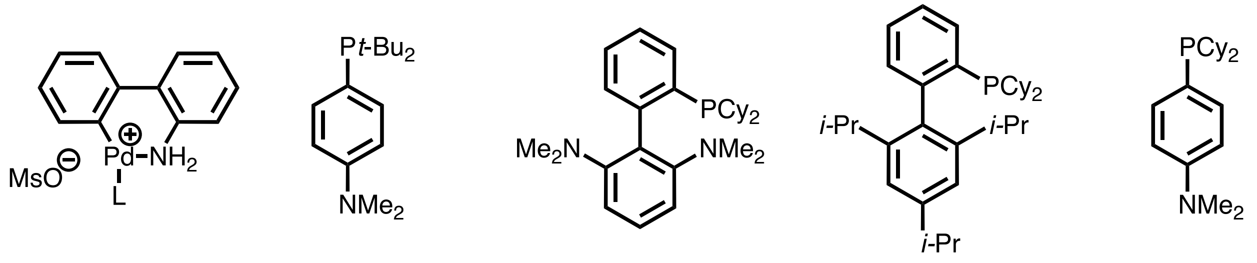

L-PdG3 APhos CPhos XPhos CyAPhos

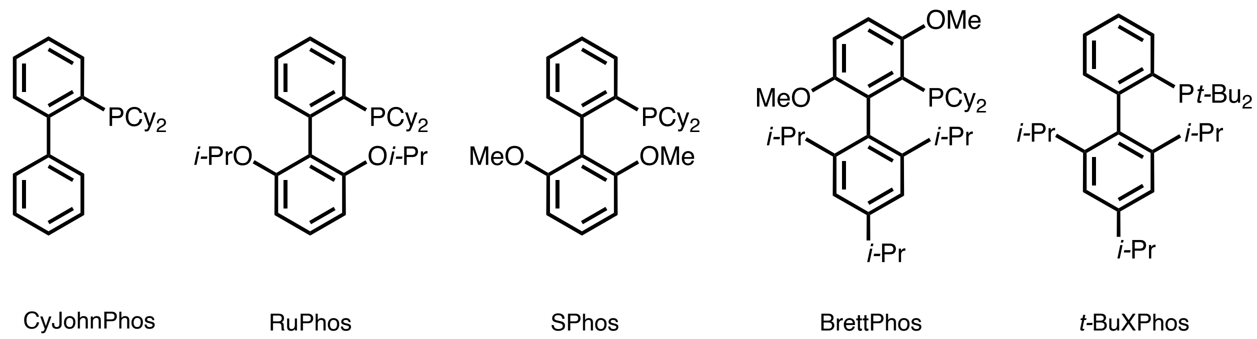

(b) Optimization of the reaction conditions: enantioselective variants

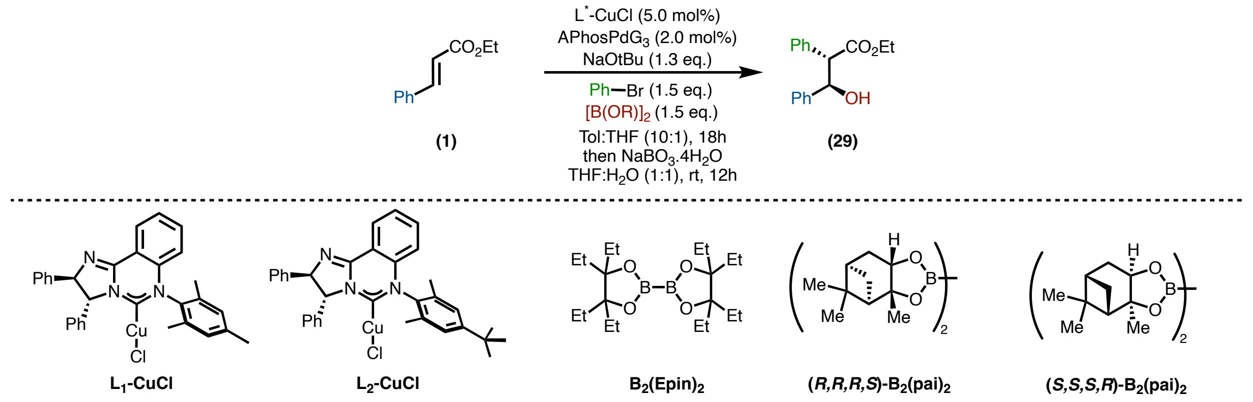

entry	temp.	L	$[(RO)_2B\text{-}B(OR)_2]$	NMR yield[a]	dr[b]	er[c]
1	30 ^{0}C	L_1	$B_2(pin)_2$	83%	13:1	77:23
2	4 ^{0}C	L_1	$B_2(pin)_2$	99%	14:1	78:22
3	-20 ^{0}C	L_1	$B_2(pin)_2$	92%	>20:1	80:20
4	30 ^{0}C	L_2	$B_2(pin)_2$	88%	8:1	76:24
5	30 ^{0}C	L_1	$B_2(Epin)_2$	89%	10:1	77.5:22.5
6	30 ^{0}C	L_1	(*R,R,R,S*)-$B_2(pai)_2$	84%	12:1	90:10
7	30 ^{0}C	L_1	(*S,S,S,R*)-$B_2(pai)_2$	58%	10:1	75:25
8[d]	30 ^{0}C	L_1	(*R,R,R,S*)-$B_2(pai)_2$	65%	15:1	63:37

[a]NMR yields were determined by ^{1}HNMR analysis of crude mixture using CH_2Br_2 as internal standard
[b]dr were determined by ^{1}HNMR analysis of crude reaction mixture
[c]enantiomeric ratio were determined by HPLC analysis using chiral columns
[d]reaction with (*Z*)-ethylcinnamate

6. **Experimental characterization Data:**

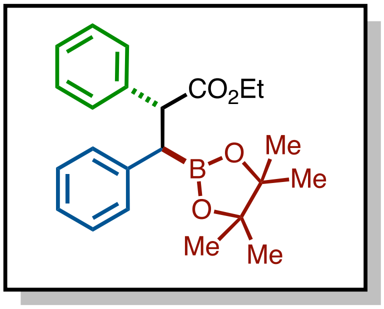

ethyl 2,3-diphenyl-3-(4,4,5,5-tetramethyl-1,3,2-dioxaborolan-2-yl) propanoate **(2)**

The title compound was prepared according to General Procedure A. Purification by silica-gel flash column chromatography (Gradient: Hexane to 3-5% EtOAc:Hexanes) yields with 88% (average of two runs) and >20:1 of dr as colorless oil.

^{1}H NMR (500 MHz, $CDCl_3$) δ 7.18 – 7.05 (m, 5H), 7.00 (ddd, *J* = 17.7, 6.8, 1.6 Hz, 5H), 4.21 (dq, *J* = 10.5, 7.1 Hz, 1H), 4.06 (dq, *J* = 10.9, 7.4 Hz, 1H), 4.0 (d, *J* = 10.3, 7.4 Hz, 1H), 2.96 (d, *J* = 12.0 Hz, 1H), 1.26 (s, 6H), 1.21 (t, *J* = 7.1 Hz, 3H), 1.17 (s, 6H).

^{13}C NMR (126 MHz, $CDCl_3$) δ 174.7, 139.2, 138.8, 128.2, 128.2, 128.1, 126.8, 125.6, 83.7, 61.0, 54.8, 24.6, 24.6, 14.3

^{11}B NMR (160 MHz, $CDCl_3$) δ 33.30

HRMS (APCI+): Calculated for $C_{23}H_{29}O_4BH$ $[M+H]^+$: 381.2235, Found: 381.2232.

IR (Neat): 2972, 1729, 1505, 1288, 1213 cm^{-1}

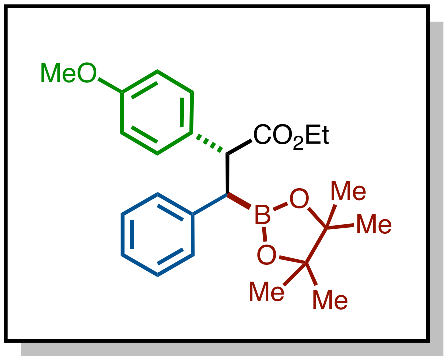

ethyl-2-(4-methoxyphenyl)-3-phenyl-3-(4,4,5,5-tetramethyl-1,3,2-dioxaborolan-2-yl) propanoate **(3)**

The title compound was prepared according to General Procedure A. Purification by silica-gel flash column chromatography (Gradient: Hexane to 16-17% EtOAc:Hexanes) yields with 88% (average of two runs) with 20:1 dr as white sticky solid.

^{1}H NMR (500 MHz, $CDCl_3$δ 7.09 (dd, *J* = 8.1, 6.8 Hz, 2H), 7.06 – 6.97 (m, 3H), 6.97 – 6.91 (m, 2H), 6.70 – 6.63 (m, 2H), 4.20 (dq, *J* = 10.8, 7.1 Hz, 1H), 4.05 (dq, *J* = 10.8, 7.1 Hz, 1H), 3.95 (d, *J* = 12.4 Hz, 1H), 3.70 (s, 3H), 2.93 (d, *J* = 12.4 Hz, 1H), 1.25 (s, 6H), 1.21 (t, *J* = 7.1 Hz, 3H), 1.16 (s, 6H).

^{13}C NMR (126 MHz, CDCl$_3$) δ 174.9, 158.4, 139.4, 131.0, 129.2, 129.1, 128.3, 125.6, 113.7, 83.7, 60.94, 5.19, 53.9, 24.7, 24.6, 14.3
^{11}B NMR (160 MHz, CDCl$_3$) δ 32.85
HRMS (ESI+): Calculated for $C_{24}H_{31}O_5BNa$ $[M+Na]^+$: 433.2156, Found: 434.2155
IR (Neat): 2976, 1724, 1435, 1329, 1214, 1139 cm^{-1}

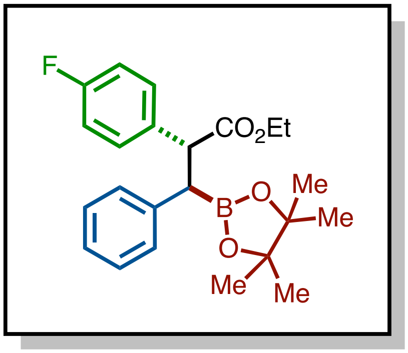

***ethyl-2-(4-fluorophenyl)-3-phenyl-3-(4,4,5,5-tetramethyl-1,3,2-dioxaborolan-2-yl) propanoate* (4)**
The title compound was prepared according to General Procedure A. Purification by silica-gel flash column chromatography (Gradient: Hexane to 4-5% EtOAc:Hexanes) yields with 75% (average of two runs) with 11:1 dr as white sticky solid.
^{1}H NMR (500 MHz, CDCl$_3$ δ 7.09 (td, J = 7.3, 1.4 Hz, 2H), 7.06 – 6.99 (m, 1H), 6.99 – 6.94 (m, 4H), 6.85 – 6.77 (m, 2H), 4.20 (dq, J = 10.8, 7.1 Hz, 1H), 4.07 (dq, J = 10.7, 7.0 Hz, 1H), 3.99 (d, J = 12.4 Hz, 1H), 2.91 (d, J = 12.4 Hz, 1H), 1.25 (s, 6H), 1.24 – 1.20 (m, 3H), 1.17 (s, 6H).
^{13}C NMR (126 MHz, CDCl$_3$) δ 174.5, 161.8 (d, J = 244.8 Hz), 141.5, 139.0, 134.6 (d, J = 3.2 Hz), 129.6 (d, J = 8.1 Hz), 128.4, 125.8, 115.2 (d, J = 21.5 Hz), 83.8, 61.1, 54.0, 37.5, 24.6, 24.6, 14.3
^{19}F NMR (376 MHz, CDCl$_3$) δ -116.15
^{11}B NMR (160 MHz, CDCl$_3$) δ 33.32
HRMS (EI+): Calculated for $C_{23}H_{28}O_4FB$ $[M]^+$: 398.2071, Found: 398.2071
IR (Neat): 2975, 1725, 1335, 1239, 1140 cm^{-1}

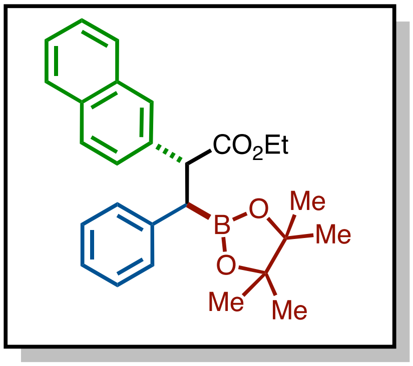

***ethyl-2-(naphthalen-2-yl)-3-phenyl-3-(4,4,5,5-tetramethyl-1,3,2-dioxaborolan-2-yl) propanoate* (5)**

The title compound was prepared according to General Procedure A. Purification by silica-gel flash column chromatography (Gradient: Hexane to 10-12% EtOAc:Hexanes) yields with 77% (average of two runs) with >20:1 dr as colorless oil.

^{1}H NMR (500 MHz, CDCl$_3$): δ 7.78 – 7.69 (m, 1H), 7.67 (dd, J = 13.9, 8.9 Hz, 2H), 7.48 (s, 1H), 7.43 – 7.36 (m, 2H), 7.22 (dd, J = 8.5, 1.8 Hz, 1H), 7.09 – 6.95 (m, 5H), 4.29 – 4.17 (m, 2H), 4.12 – 4.02 (m, 1H), 3.11 (d, J = 12.3 Hz, 1H), 1.29 (s, 6H), 1.25 – 1.18 (m, 9H).

^{13}C NMR (126 MHz, CDCl$_3$): δ 174.6, 139.0, 136.3, 133.3, 132.4, 129.0, 128.2, 127.9, 127.8, 127.5, 127.2, 126.0, 125.8, 125.6, 125.5, 83.7, 61.0, 54.8, 24.7, 24.6, 14.2

^{11}B NMR (160 MHz, CDCl$_3$) δ 33.32

HRMS (APCI+): Calculated for $C_{27}H_{32}O_4B$ $[M+H]^+$: 431.2388, Found: 431.2391.

IR (Neat): 2976, 1727, 1499, 1359, 1230, 1141 cm^{-1}

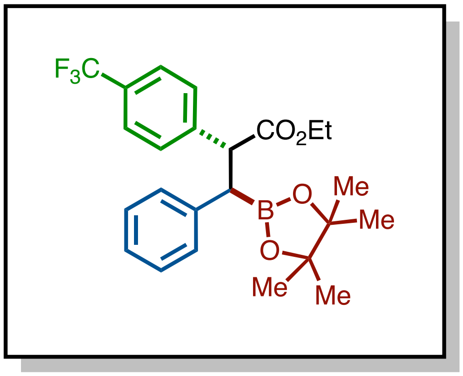

***ethyl-3-phenyl-3-(4,4,5,5-tetramethyl-1,3,2-dioxaborolan-2-yl)-2-(4-(trifluoromethyl) phenyl) propanoate* (6)**

The title compound was prepared according to General Procedure A. Purification by silica-gel flash column chromatography (Gradient: Hexane to 3-4% EtOAc:Hexanes) yields with 79% (average of two runs) with 10:1 dr as colorless oil.

^{1}H NMR (500 MHz, CDCl$_3$) δ 7.38 (d, J = 8.0 Hz, 2H), 7.23 (dd, J = 8.0, 6.3 Hz, 1H), 7.14 – 7.07 (m, 3H), 7.05 – 7.01 (m, 1H), 7.00 – 6.94 (m, 2H), 4.21 (dd, J = 10.8, 7.1 Hz, 1H), 4.16 – 4.02 (m, 2H), 2.95 (d, J = 12.4 Hz, 1H), 1.26 (s, 6H), 1.25 – 1.18 (m, 3H), 1.17 (s, 6H).

^{13}C NMR (126 MHz, CDCl$_3$) δ 174.0, 143.0, 141.5, 138.6, 129.0, 128.6 (d, J = 35.5 Hz), 125.9, 125.3 (q, J = 3.5 Hz),123.0 (d, J = 272.9 Hz), 83.9, 61.3, 54.6, 37.5, 24.6, 24.6, 14.3

^{19}F NMR (376 MHz, CDCl$_3$) δ -62.55

HRMS (EI+): Calculated for $C_{24}H_{28}O_4BF_3$ $[M]^+$: 448.2043, Found: 448.2043

IR (Neat): 2975, 1727, 1435, 1327, 1214, 1140 cm^{-1}

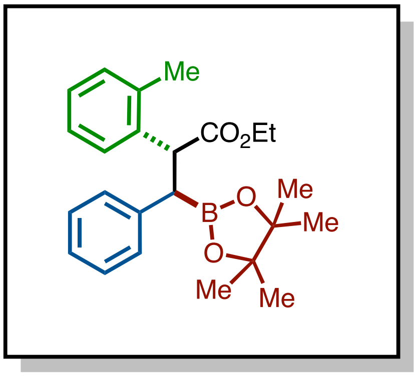

ethyl-3-phenyl-3-(4,4,5,5-tetramethyl-1,3,2-dioxaborolan-2-yl)-2-(o-tolyl) propanoate **(7)**

The title compound was prepared according to General Procedure A. Purification by silica-gel flash column chromatography (Gradient: Hexane to 4-6% EtOAc:Hexanes) yields with 62% (average of two runs) with 14:1 dr as colorless oil.

^{1}H NMR (500 MHz, $CDCl_3$) δ 7.25 (m, 1H), 7.15 – 6.95 (m, 8H), 6.93 – 6.85 (m, 1H), 4.29 (d, *J* = 12.3 Hz, 1H), 4.19 (dq, *J* = 10.7, 7.1 Hz, 1H), 4.02 (dq, *J* = 10.8, 7.1 Hz, 1H), 2.98 (d, *J* = 12.2 Hz, 1H), 1.98 (s, 3H), 1.29 (s, 6H), 1.24 – 1.16 (m, 9H).

^{13}C NMR (126 MHz, $CDCl_3$) δ 175.1, 141.5, 139.2, 137.6, 136.8, 130.1, 129.3, 128.1, 126.6, 126.1, 125.7, 83.7, 60.9, 50.2, 37.5, 24.7, 24.7, 19.8, 14.3

^{11}B NMR (160 MHz, $CDCl_3$) δ 33.11

HRMS (ESI+): Calculated for $C_{24}H_{31}O_4BNa$ $[M+Na]^+$: 417.2207, Found: 417.2203

IR (Neat): 2978, 1724, 1495, 1241, 1140 cm^{-1}

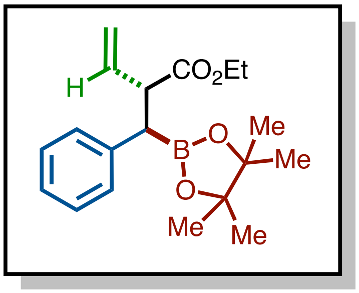

ethyl-2-(phenyl(4,4,5,5-tetramethyl-1,3,2-dioxaborolan-2-yl) methyl) but-3-enoate **(8)**

The title compound was prepared according to General Procedure A. Purification by silica-gel flash column chromatography (Gradient: Hexane to 8-9% EtOAc:Hexanes) yields with 75% (average of two runs) with 5.5:1 dr as colorless oil.

^{1}H NMR (500 MHz, $CDCl_3$) δ 7.29-7.25 (m,1H), 7.24 – 7.19 (m, 1H), 7.17 – 7.10 (m, 3H), 5.59 (ddd, *J* = 17.0, 10.2, 8.4 Hz, 1H), 4.97 – 4.88 (m, 2H), 4.26 – 4.06 (m, 2H), 3.54 (dd, *J* = 11.6, 8.4 Hz, 1H), 2.69 (d, *J* = 11.6 Hz, 1H), 1.29 (t, *J* = 7.1 Hz, 3H), 1.20 (s, 6H), 1.13 (s, 6H).

^{13}C NMR (126 MHz, $CDCl_3$) δ 174.3, 139.5, 135.6, 129.3, 128.6, 128.4, 128.3, 125.8, 118.0, 83.7, 60.9, 52.5, 24.7, 24.6, 24.6, 14.4

^{11}B NMR (160 MHz, $CDCl_3$) δ 32.73

HRMS (APCI+): Calculated for $C_{19}H_{27}O_4BNa$ $[M+Na]^+$: 353.1895, Found: 353.1896

IR (Neat): 2979, 1728, 1452, 1346, 1284, 1141 cm^{-1}

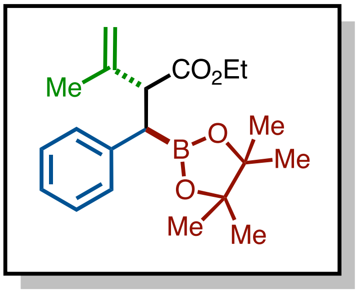

ethyl-3-methyl-2-(phenyl(4,4,5,5-tetramethyl-1,3,2-dioxaborolan-2-yl) methyl) but-3-enoate **(9)**

The title compound was prepared according to General Procedure A. Purification by silica-gel flash column chromatography (Gradient: Hexane to 6-8% EtOAc:Hexanes) yields with 90% (average of two runs) with 11:1 dr as colorless oil.

^{1}H NMR (500 MHz, CDCl$_3$) δ 7.22 (t, J = 7.6 Hz, 2H), 7.18 – 7.09 (m, 3H), 4.75 – 4.62 (m, 2H), 4.21 (dq, J = 10.8, 7.1 Hz, 1H), 4.14 (dq, J = 10.8, 7.1 Hz, 1H), 3.64 (d, J = 12.5 Hz, 1H), 2.79 (d, J = 12.5 Hz, 1H), 1.55 (s, 3H), 1.28 (t, J = 7.1 Hz, 3H), 1.20 (s, 6H), 1.12 (s, 6H).

^{13}C NMR (126 MHz, CDCl$_3$) δ 174.4, 141.4, 139.6, 128.9, 128.4, 125.7, 115.4, 83.6, 60.9, 55.9, 24.6, 19.7, 14.4

^{11}B NMR (160 MHz, CDCl$_3$) δ 33.16

HRMS (APCI+): Calculated for $C_{20}H_{29}O_4BNa$ $[M+Na]^+$: 367.2051, Found: 367.2052

IR (Neat): 2978, 1725, 1451, 1360, 1345, 1140 cm^{-1}

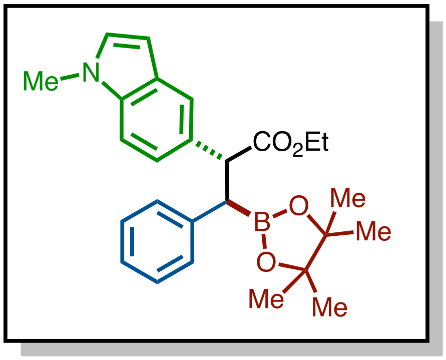

ethyl-2-(1-methyl-1H-indol-5-yl)-3-phenyl-3-(4,4,5,5-tetramethyl-1,3,2-dioxaborolan-2-yl) propanoate **(10)**

The title compound was prepared according to General Procedure A. Purification by silica-gel flash column chromatography (Gradient: Hexane to 16-17% EtOAc:Hexanes) yields with 74% (average of two runs) with >20:1 dr as colorless oil.

^{1}H NMR (500 MHz, CDCl$_3$) δ 7.29 (s, 1H), 7.12 – 6.99 (m, 5H), 6.99 – 6.91 (m, 3H), 6.32 (d, J = 3.0 Hz, 1H), 4.21 (dqd, J = 11.0, 7.1, 1.1 Hz, 1H), 4.10 (d, J = 12.3 Hz, 1H), 4.06 – 3.96 (m, 1H), 3.69 (s, 3H), 3.07 (d, J = 12.3 Hz, 1H), 1.26 (s, 6H), 1.22 – 1.18 (m, 3H), 1.17 (s, 6H).

^{13}C NMR (126 MHz, CDCl$_3$) δ 175.5, 139.7, 135.9, 129.7, 129.2, 128.9, 128.5, 128.2, 125.4, 121.9, 120.6, 109.0, 100.9, 83.6, 60.8, 54.7, 32.9, 29.8, 24.7, 24.7, 14.4

^{11}B NMR (160 MHz, $CDCl_3$) δ 33.42
HRMS (APCI+): Calculated for $C_{26}H_{33}O_4NB$ $[M+H]^+$: 434.2497, Found: 434.2502.
IR (Neat): 2975, 1727, 1445, 1350, 1246, 1138 cm^{-1}

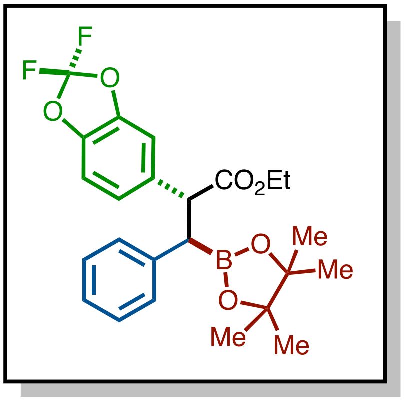

ethyl-2-(2,2-difluorobenzo[d] [1,3] dioxol-5-yl)-3-phenyl-3-(4,4,5,5-tetramethyl-1,3,2-dioxaborolan-2-yl)propanoate **(11)**

The title compound was prepared according to General Procedure A. Purification by silica-gel flash column chromatography (Gradient: Hexane to 10-11% EtOAc:Hexanes) yields with 81% (average of two runs) with >20:1 dr as colorless oil.

^{1}H NMR (500 MHz, $CDCl_3$): δ 7.14 (t, J = 7.4 Hz, 2H), 7.10 – 7.04 (m, 1H), 7.04 – 6.98 (m, 2H), 6.84 – 6.79 (m, 2H), 6.73 (dd, J = 8.3, 1.7 Hz, 1H), 4.24 (dq, J = 10.9, 7.1 Hz, 1H), 4.16 – 4.05 (m, 1H), 4.02 (d, J = 12.4 Hz, 1H), 2.93 (d, J = 12.4 Hz, 1H), 1.32 – 1.21 (m, 9H), 1.18 (s, 6H).
^{13}C NMR (126 MHz, $CDCl_3$): δ 174.0, 143.7, 142.5, 138.6, 134.9, 128.9, 128.5, 128.4, 125.8, 123.5, 109.1, 109.0, 83.8, 61.2, 54.3, 24.5, 24.5, 14.2
^{11}B NMR (160 MHz, $CDCl_3$) δ 34.10
HRMS (APCI+): Calculated for $C_{24}H_{28}O_6F_2B$ $[M+H]^+$: 461.1942, Found: 461.1946.
IR (Neat): 2975, 1726, 1487, 1356, 1239, 1141 cm^{-1}

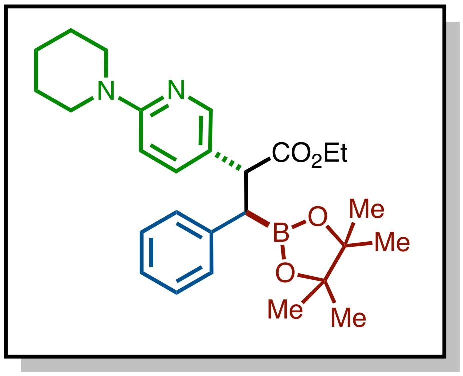

ethyl-3-phenyl-2-(5-(piperidin-1-yl) pyridin-2-yl)-3-(4,4,5,5-tetramethyl-1,3,2-dioxaborolan-2-yl) propanoate **(12)**

The title compound was prepared according to General Procedure A. Purification by silica-gel flash column chromatography (Gradient: Hexane to 12-13% EtOAc:Hexanes) yields with 74% (average of two runs) with >20:1 dr as colorless oil.

^{1}H NMR (400 MHz, CDCl$_3$): δ 7.80 (d, *J* = 2.5 Hz, 1H), 7.17 (dd, *J* = 8.8, 2.5 Hz, 1H), 7.13 – 7.05 (m, 2H), 7.01 (d, *J* = 7.3 Hz, 3H), 6.44 (d, *J* = 8.9 Hz, 1H), 4.17 (dq, *J* = 10.7, 7.1 Hz, 1H), 4.02 (dq, *J* = 10.8, 7.1 Hz, 1H), 3.90 (d, *J* = 12.4 Hz, 1H), 3.39 (d, *J* = 5.3 Hz, 4H), 2.92 (d, *J* = 12.4 Hz, 1H), 1.56 (m, 6H), 1.30 – 1.16 (m, 9H), 1.13 (s, 6H).

^{13}C NMR (126 MHz, CDCl$_3$): δ 174.6, 147.7, 138.9, 136.5, 129.0, 128.3, 125.6, 122.4, 106.8, 83.6, 60.9, 51.0, 46.3, 25.5, 24.6, 24.5, 24.5, 14.2

^{11}B NMR (160 MHz, CDCl$_3$) δ 33.97

HRMS (APCI+): Calculated for $C_{27}H_{38}O_4N_2B$ $[M+H]^+$: 465.2919, Found: 465.2923.

IR (Neat): 2978, 1724, 1489, 1324, 1141 cm^{-1}

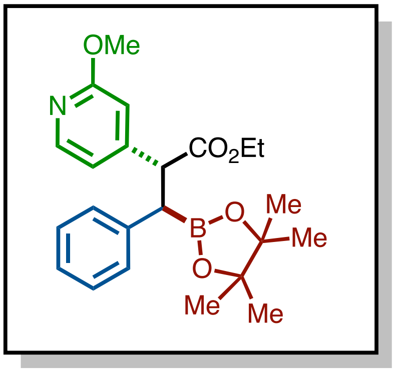

***ethyl-2-(2-methoxypyridin-4-yl)-3-phenyl-3-(4,4,5,5-tetramethyl-1,3,2-dioxaborolan-2-yl) propanoate* (13)**

The title compound was prepared according to General Procedure A. Purification by silica-gel flash column chromatography (Gradient: Hexane to 15-18% EtOAc:Hexanes) yields with 56% (average of two runs) with >20:1 dr as colorless oil.

^{1}H NMR (500 MHz, CDCl$_3$): δ 7.94 (d, *J* = 5.3 Hz, 1H), 7.14 (t, *J* = 7.4 Hz, 2H), 7.10 – 6.98 (m, 3H), 6.59 (dd, *J* = 5.3, 1.5 Hz, 1H), 6.42 (s, 1H), 4.23 (dq, *J* = 10.9, 7.2 Hz, 1H), 4.09 (dq, *J* = 10.9, 7.1 Hz, 1H), 4.04 – 3.97 (m, 1H), 3.85 (s, 3H), 2.95 (d, *J* = 12.4 Hz, 1H), 1.25 (d, *J* = 10.4 Hz, 9H), 1.17 (s, 6H).

^{13}C NMR (126 MHz, CDCl$_3$): δ 173.3, 164.2, 150.3, 147.7, 146.6, 138.3, 128.8, 128.4, 125.9, 116.6, 114.9, 110.4, 108.7, 83.8, 61.3, 54.0, 53.3, 24.5, 24.5, 14.1

^{11}B NMR (160 MHz, CDCl$_3$) δ 32.49

HRMS (APCI+): Calculated for $C_{23}H_{31}O_5NB$ $[M+H]^+$: 412.2290, Found: 412.2294.

IR (Neat): 2980, 1725, 1369, 1351, 1243, 1144 cm^{-1}

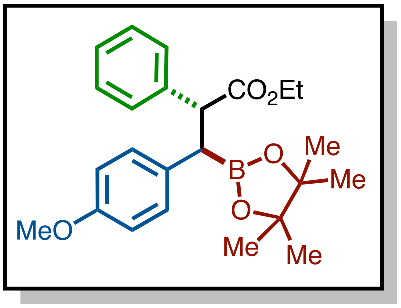

***ethyl-3-(4-methoxyphenyl)-2-phenyl-3-(4,4,5,5-tetramethyl-1,3,2-dioxaborolan-2-yl) propanoate* (14)**

The title compound was prepared according to General Procedure A. Purification by silica-gel flash column chromatography (Gradient: Hexane to 14-15% EtOAc: Hexanes) yields with 78% (average of two runs) and >20:1 of dr as colorless oil.

^{1}H NMR (400 MHz, $CDCl_3$) δ 7.18 – 7.05 (m, 3H), 7.04 – 6.97 (m, 2H), 6.92 – 6.84 (m, 2H), 6.67 – 6.59 (m, 2H), 4.20 (dq, J = 10.8, 7.1 Hz, 1H), 4.05 (dq, J = 10.8, 7.1 Hz, 1H), 3.94 (d, J = 10.4, 7.5 Hz, 1H), 3.69 (s, 3H), 2.89 (d, J = 12.3 Hz, 1H), 1.26 (s, 6H), 1.21 (t, J = 7.1 Hz, 3H), 1.17 (s, 6H);

^{13}C NMR (126 MHz, $CDCl_3$) δ 174.7, 157.6, 139.0, 131.1, 130.0, 128.3, 128.2, 126.8, 113.7, 83.6, 60.9, 55.1, 55.0, 24.6, 24.6, 14.3

^{11}B NMR (160 MHz, $CDCl_3$) δ 33.17

HRMS (APCI+): Calculated for $C_{24}H_{31}O_5BNa$ $[M+Na]^+$: 433.2157, Found: 433.2154

IR (Neat): 2977, 1724, 1510, 1347, 1247 cm^{-1}

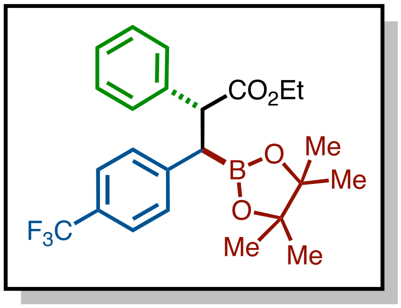

***ethyl-2-phenyl-3-(4,4,5,5-tetramethyl-1,3,2-dioxaborolan-2-yl)-3-(4-(trifluoromethyl) phenyl) propanoate* (15)**

The title compound was prepared according to General Procedure A. Purification by silica-gel flash column chromatography (Gradient: Hexane to 4-5% EtOAc:Hexanes) yields with 46% (average of two runs) and 14:1 of dr as colorless oil

^{1}H NMR (500 MHz, $CDCl_3$) δ 7.34 (d, J = 8.0 Hz, 2H), 7.12 (dt, J = 16.5, 7.1 Hz, 5H), 7.01 (dd, J = 8.0, 1.7 Hz, 2H), 4.21 (dq, J = 10.7, 7.1 Hz, 1H), 4.10 – 4.04 (m, 1H), 4.02 (d, J = 12.3 Hz, 1H), 3.06 (d, J = 12.3 Hz, 1H), 1.26 (s, 6H), 1.21 (t, J = 7.1 Hz, 3H), 1.17 (s, 6H).

^{13}C NMR (126 MHz, $CDCl_3$) δ 174.3, 143.7, 138.3, 129.3, 128.6, 128.5, 128.0, 127.1, 125.2 (q, J = 3.8 Hz), 123.3(q, J = 271.6 Hz), 84.0, 61.2, 54.5, 24.6, 24.6, 14.3

^{19}F NMR (376 MHz, CDCl$_3$) δ -62.39
^{11}B NMR (160 MHz, CDCl$_3$) δ 33.54
HRMS (APCI+): Calculated for $C_{24}H_{28}O_4BF_4Na$ [M+Na]$^+$: 471.1925, Found: 471.1920
IR (Neat): 2979, 1725, 1324, 1286, 1139 cm^{-1}

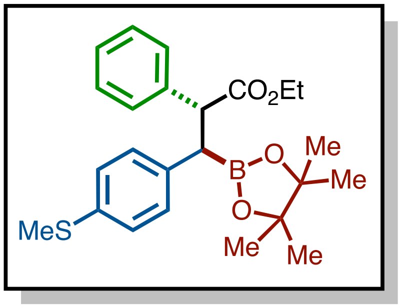

***ethyl-3-(4-(methylthio) phenyl)-2-phenyl-3-(4,4,5,5-tetramethyl-1,3,2-dioxaborolan-2-yl) propanoate* (16)**

The title compound was prepared according to General Procedure A. Purification by silica-gel flash column chromatography (Gradient: Hexane to 4-5% EtOAc:Hexanes) yields with 88% (average of two runs) and 9:1 of dr as colorless oil

^{1}H NMR (500 MHz, CDCl$_3$) δ 7.13 (dtd, J = 11.5, 6.6, 2.0 Hz, 3H), 7.05 – 6.95 (m, 4H), 6.93 – 6.87 (m, 2H), 4.19 (ddd, J = 13.9, 7.9, 5.5 Hz, 1H), 4.10 – 4.00 (m, 1H), 3.96 (d, J = 12.4 Hz, 1H), 2.93 (d, J = 12.4 Hz, 1H), 2.38 (s, 3H), 1.25 (s, 6H), 1.20 (t, J = 7.1 Hz, 3H), 1.17 (s, 6H).
^{13}C NMR (126 MHz, CDCl$_3$) δ 174.6, 138.7, 136.2, 135.0, 129.6, 128.4, 128.2, 126.9, 126.8, 83.8, 61.02, 54.7, 24.7, 24.6, 16.0, 14.3
^{11}B NMR (160 MHz, CDCl$_3$) δ 34.12
HRMS (APCI+): Calculated for $C_{24}H_{31}O_4BSNa$ [M+Na]$^+$:449.1928, Found: 449.1925
IR (Neat): 2978, 1724, 1361, 1323, 1261, 1139 cm^{-1}

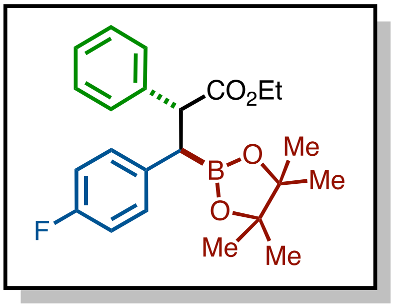

***ethyl 3-(4-fluorophenyl)-2-phenyl-3-(4,4,5,5-tetramethyl-1,3,2-dioxaborolan-2-yl) propanoate* (17)**

The title compound was prepared according to General Procedure A. Purification by silica-gel flash column chromatography (Gradient: Hexane to 4-5% EtOAc:Hexanes) yields with 73% (average of two runs) and 9:1 of dr as colorless oil

^{1}H NMR (400 MHz, CDCl$_3$) δ 7.17 – 7.09 (m, 3H), 7.03 – 6.96 (m, 2H), 6.96 – 6.89 (m, 2H), 6.82 – 6.72 (m, 2H), 4.21 (dq, J = 10.8, 7.1 Hz, 1H), 4.05 (dq, J = 10.8, 7.1 Hz, 1H), 3.94 (d, J = 12.4 Hz, 1H), 2.93 (d, J = 12.4 Hz, 1H), 1.26 (s, 6H), 1.21 (t, J = 7.1 Hz, 3H), 1.17 (s, 6H).
^{13}C NMR (126 MHz, CDCl$_3$) δ 174.5, 161.2 (d, J = 243.3 Hz), 138.7, 134.9(d, J = 3.1 Hz), 130.4(d, J = 7.8 Hz), 128.4, 128.1, 127.0, 115.2 (d, J = 21.1 Hz), 83.8, 55.0, 24.7, 24.6, 14.3
^{19}F NMR (376 MHz, CDCl$_3$) δ -117.93
^{11}B NMR (160 MHz, CDCl$_3$) δ 33.34
HRMS (APCI+): Calculated for $C_{23}H_{28}O_4BFNa$ $[M+Na]^+$:421.1957, Found: 421.1955
IR (Neat): 2978, 1725, 1361, 1359, 1289, 1139 cm^{-1}

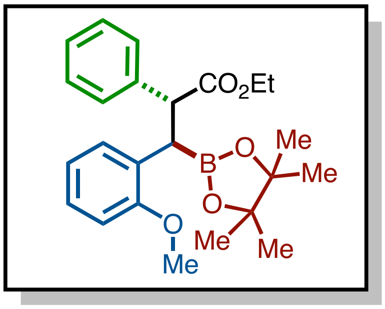

ethyl -3-(2-methoxyphenyl)-2-phenyl-3-(4,4,5,5-tetramethyl-1,3,2-dioxaborolan-2-yl) propanoate **(18)**

The title compound was prepared according to General Procedure A. Purification by silica-gel flash column chromatography (Gradient: Hexane to 4-5% EtOAc:Hexanes) yields with 76% (average of two runs) and 6:1 of dr as colorless oil
^{1}H NMR (500 MHz, CDCl$_3$) δ 7.11 – 6.92 (m, 6H), 6.84 (dd, J = 7.7, 1.8 Hz, 1H), 6.63 (ddd, J = 7.5, 3.5, 2.3 Hz, 2H), 4.23 (dq, J = 10.8, 7.1 Hz, 1H), 4.06 (ddd, J = 14.2, 11.6, 6.8 Hz, 2H), 3.65 (s, 3H), 3.24 (d, J = 12.2 Hz, 1H), 1.27 (s, 6H), 1.24 (t, J = 7.2 Hz, 3H), 1.19 (s, 6H).
^{13}C NMR (126 MHz, CDCl$_3$) δ 174.7, 157.3, 139.2, 131.4, 128.2, 128.2, 127.9, 126.9, 126.6, 120.4, 110.0, 83.5, 60.8, 55.0, 52.8, 24.8, 24.7, 14.3
^{11}B NMR (160 MHz, CDCl$_3$) δ 34.04
HRMS (APCI+): Calculated for $C_{24}H_{31}O_5BNa$ $[M+Na]^+$:433.2157, Found: 433.2155
IR (Neat): 2978, 1727, 1494, 1354, 1243, 1140 cm^{-1}

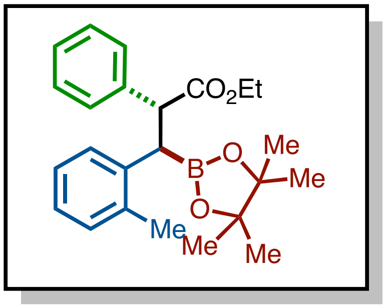

ethyl -2-phenyl-3-(4,4,5,5-tetramethyl-1,3,2-dioxaborolan-2-yl)-3-(o-tolyl) propanoate **(19)**

The title compound was prepared according to General Procedure A. Purification by silica-gel flash column chromatography (Gradient: Hexane to 4-5% EtOAc:Hexanes) yields with 79% (average of two runs) and 7:1 of dr as colorless oil

^{1}H NMR (500 MHz, CDCl$_3$) δ 7.28 (d, *J* = 7.8 Hz, 1H), 7.14 – 7.02 (m, 4H), 6.98 (dd, *J* = 6.7, 3.0 Hz, 2H), 6.96 – 6.86 (m, 2H), 4.24 (dq, *J* = 10.8, 7.1 Hz, 1H), 4.15 – 4.02 (m, 2H), 3.24 (d, *J* = 12.3 Hz, 1H), 1.92 (s, 3H), 1.24 (s, 6H), 1.23 (t, *J* = 7.2 Hz, 3H), 1.15 (s, 6H).

^{13}C NMR (126 MHz, CDCl$_3$) δ 174.9, 139.0, 137.6, 137.2, 130.3, 128.1, 128.0, 126.8, 125.7, 125.4, 83.6, 61.0, 54.9, 24.6, 24.6, 20.1, 14.3

^{11}B NMR (160 MHz, CDCl$_3$) δ 33.06

HRMS (APCI+): Calculated for $C_{24}H_{31}O_4BNa$ $[M+Na]^+$:417.2208, Found: 417.2207

IR (Neat): 2978, 1725, 1346, 1323, 1237, 1140 cm^{-1}

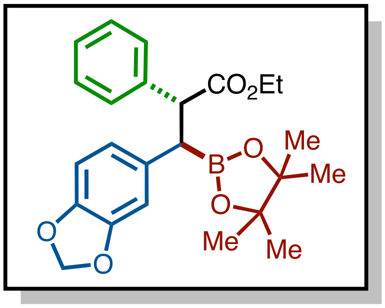

***ethyl 3-(1,3-dihydroisobenzofuran-5-yl)-2-phenyl-3-(4,4,5,5-tetramethyl-1,3,2-dioxaborolan-2-yl) propanoate* (20)**

The title compound was prepared according to General Procedure A. Purification by silica-gel flash column chromatography (Gradient: Hexane to 4-5% EtOAc:Hexanes) yields with 96% (average of two runs) and >20:1 of dr as colorless oil

^{1}H NMR (500 MHz, CDCl$_3$) δ 7.19 – 7.07 (m, 3H), 7.07 – 7.00 (m, 2H), 6.55 – 6.49 (m, 2H), 6.41 (dd, *J* = 8.0, 1.8 Hz, 1H), 5.82 (q, *J* = 1.5 Hz, 2H), 4.19 (dq, *J* = 10.7, 7.1 Hz, 1H), 4.04 (dq, *J* = 10.8, 7.1 Hz, 1H), 3.92 (d, *J* = 12.3 Hz, 1H), 2.87 (d, *J* = 12.3 Hz, 1H), 1.26 (s, 6H), 1.19 (t, *J* = 7.2 Hz, 3H), 1.18 (s, 6H).

^{13}C NMR (126 MHz, CDCl$_3$) δ 174.6, 147.5, 145.5, 138.8, 132.9, 128.4, 126.9, 122.2, 109.4, 108.2, 100.7, 83.7, 61.0, 55.0, 24.7, 24.6, 14.3

^{11}B NMR (160 MHz, CDCl$_3$) δ 33.18

HRMS (APCI+): Calculated for $C_{24}H_{29}O_6BNa$ $[M+Na]^+$:447.1949, Found: 447.1945

IR (Neat): 2978, 1727, 1492, 1356, 1243, 1140 cm^{-1}

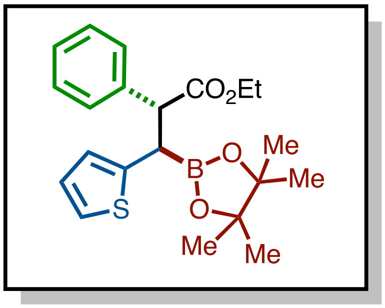

***ethyl -2-phenyl-3-(4,4,5,5-tetramethyl-1,3,2-dioxaborolan-2-yl)-3-(thiophen-2-yl) propanoate* (21)**

The title compound was prepared according to General Procedure A. Purification by silica-gel flash column chromatography (Gradient: Hexane to 4-5% EtOAc:Hexanes) yields with 78% (average of two runs) and 5.5:1 of dr as colorless oil

^{1}H NMR (500 MHz, $CDCl_3$) δ 7.23 – 7.12 (m, 3H), 7.12 – 7.05 (m, 2H), 6.95 (dd, *J* = 5.0, 1.2 Hz, 1H), 6.73 (dd, *J* = 5.2, 3.5 Hz, 1H), 6.56 – 6.52 (m, 1H), 4.20 (dq, *J* = 10.8, 7.2 Hz, 1H), 4.05 (dq, *J* = 10.8, 7.2 Hz, 1H), 3.95 (d, *J* = 12.2 Hz, 1H), 3.27 (d, *J* = 12.2 Hz, 1H), 1.29 (s, 6H), 1.25 – 1.17 (m, 9H).

^{13}C NMR (126 MHz, $CDCl_3$) δ 174.3, 141.7, 138.7, 128.4, 128.2, 127.1, 126.7, 125.5, 123.5, 84.0, 61.1, 55.7, 24.7, 24.6, 14.4

^{11}B NMR (160 MHz, $CDCl_3$) δ 32.60

HRMS (APCI+): Calculated for $C_{21}H_{27}O_4BSNa$ $[M+Na]^+$:409.1615, Found: 409.1614

IR (Neat): 2978, 1724, 1454, 1369, 1272, 1140 cm^{-1}

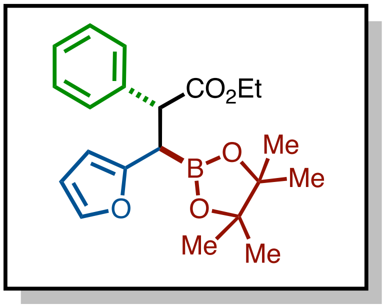

***ethyl -3-(furan-2-yl)-2-phenyl-3-(4,4,5,5-tetramethyl-1,3,2-dioxaborolan-2-yl) propanoate* (22)**

The title compound was prepared according to General Procedure A. Purification by silica-gel flash column chromatography (Gradient: Hexane to 4-5% EtOAc:Hexanes) yields with 84% (average of two runs) and 13:1 of dr as colorless oil

^{1}H NMR (500 MHz, $CDCl_3$) δ 7.25 – 7.12 (m, 4H), 7.12 – 7.06 (m, 2H), 6.08 (dd, *J* = 3.2, 1.9 Hz, 1H), 5.69 (d, *J* = 3.1 Hz, 1H), 4.20 (dq, *J* = 10.7, 7.1 Hz, 1H), 4.10 – 3.99 (m, 2H), 3.14 (d, *J* = 12.2 Hz, 1H), 1.28 (s, 6H), 1.25 – 1.15 (m, 9H).

^{13}C NMR (126 MHz, $CDCl_3$) δ 174.2, 153.0, 141.2, 139.0, 128.4, 128.0, 127.1, 110.2, 106.8, 84.1, 61.1, 52.6, 24.8, 24.5, 14.3

^{11}B NMR (160 MHz, CDCl$_3$) δ 32.07.
HRMS (APCI+): Calculated for $C_{21}H_{27}O_5BNa$ [M+Na]$^+$:393.1793, Found: 393.1791
IR (Neat): 2980, 1726, 1454(w), 1371, 1273, 1143 cm^{-1}

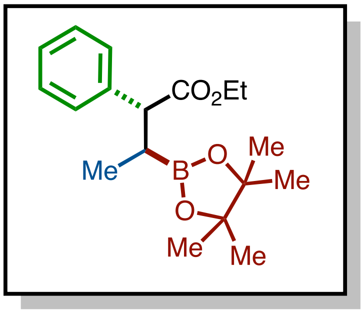

***ethyl 2-phenyl-3-(4,4,5,5-tetramethyl-1,3,2-dioxaborolan-2-yl) butanoate* (23)**

The title compound was prepared according to General Procedure A. Purification by silica-gel flash column chromatography (Gradient: Hexane to 4-5% EtOAc:Hexanes) yields with 74% (average of two runs) and 5:1 dr as colorless oil.

^{1}H NMR (500 MHz, CDCl$_3$) δ 7.31 – 7.18 (m, 5H), 4.13 (dq, J = 10.8, 7.0 Hz, 1H), 4.00 (dq, J = 10.9, 7.1 Hz, 1H), 3.48 (d, J = 11.8 Hz, 1H), 1.68 (dt, J = 11.8, 7.5 Hz, 1H), 1.27 (s, 6H), 1.23 (s, 6H), 1.16 (t, J = 7.1 Hz, 3H), 0.75 (d, J = 7.5 Hz, 3H).
^{13}C NMR (126 MHz, CDCl$_3$) δ 174.9, 139.4, 128.6, 128.5, 127.0, 83.3, 60.7, 54.7, 29.8, 24.9, 24.6, 14.3, 12.9
^{11}B NMR (160 MHz, CDCl$_3$) δ 34.74
HRMS (APCI): Calculated for $C_{18}H_{27}O_4BNa$ [M+Na]$^+$:341.1895, Found: 341.1895
IR (Neat): 2977, 1727, 1455, 1368, 1319, 1142 cm^{-1}

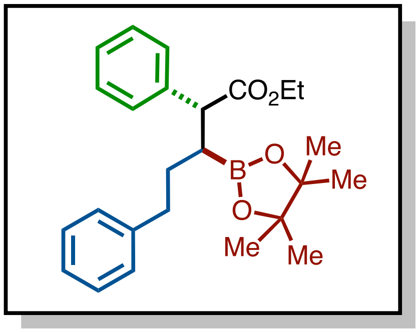

***ethyl-2,5-diphenyl-3-(4,4,5,5-tetramethyl-1,3,2-dioxaborolan-2-yl) pentanoate* (24)**

The title compound was prepared according to General Procedure A. Purification by silica-gel flash column chromatography (Gradient: Hexane to 3-5% EtOAc:Hexanes) yields with 69% (average of two runs) and 7:1 of dr as colorless oil

^{1}H NMR (500 MHz, CDCl$_3$) δ 7.31 – 7.26 (m, 2H), 7.25 – 7.16 (m, 5H), 7.11 (t, J = 7.3 Hz, 1H), 7.00 (d, J = 7.3 Hz, 2H), 4.13 (dq, J = 10.9, 7.1 Hz, 1H), 4.00 (dq, J = 10.9, 7.1 Hz, 1H), 3.64 (d,

J = 12.1 Hz, 1H), 2.66 (ddd, J = 13.5, 10.1, 6.3 Hz, 1H), 2.50 – 2.35 (m, 1H), 1.80 (ddd, J = 12.7, 8.5, 5.1 Hz, 1H), 1.52 – 1.44 (m, 2H), 1.32 (s, 6H), 1.28 (s, 6H), 1.16 (t, J = 7.0 Hz, 3H).
^{13}C NMR (126 MHz, $CDCl_3$) δ 174.8, 142.6, 139.2, 128.66, 128.5, 128.4, 128.3, 127.2, 125.7, 83.5, 60.8, 53.1, 34.9, 30.4, 25.2, 24.8, 14.3
^{11}B NMR (160 MHz, $CDCl_3$) δ 34.32
HRMS (APCI+): Calculated for $C_{25}H_{33}O_4BNa$ [M+Na]$^+$:431.2364, Found: 431.2363
IR (Neat): 2978, 1727, 1453, 1380, 1245, 1142 cm^{-1}

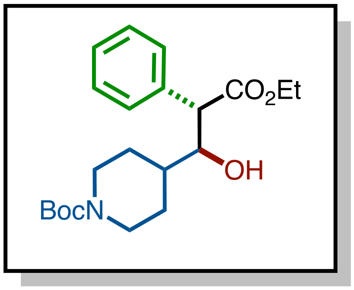

tert-butyl 4-(-3-ethoxy-3-oxo-2-phenyl-1-(4,4,5,5-tetramethyl-1,3,2-dioxaborolan-2-yl) propyl) piperidine-1-carboxylate **(25)**

The title compound was prepared according to General Procedure D. Purification by silica-gel flash column chromatography (Gradient: Hexane to 20-30% EtOAc:Hexanes) yields with 58% after two steps (average of two runs) and 20:1 of dr as colorless oil.
^{1}H NMR (500 MHz, $CDCl_3$) δ 7.36 – 7.24 (m, 5H), 4.23 – 3.98 (m, 5H), 3.75 (d, J = 8.7 Hz, 1H), 2.87 (s, 1H), 2.49 (p, J = 14.7 Hz, 2H), 1.65 (dt, J = 13.0, 2.8 Hz, 1H), 1.59 – 1.48 (m, 1H), 1.43 (s, 12H), 1.33 – 1.22 (m, 2H), 1.19 (t, J = 7.1 Hz, 3H).
^{13}C NMR (126 MHz, $CDCl_3$) δ 173.8, 154.8, 136.2, 129.0, 128.3, 127.8, 79.4, 76.4, 61.3, 55.0, 38.2, 29.3, 28.6, 25.0, 14.1
HRMS (ESI): Calculated for $C_{21}H_{31}O_5NNa$ [M+Na]$^+$:400.2094, Found: 400.2096
IR (Neat): 3470 (br), 2977, 1731, 1709, 1691, 1280 cm^{-1}

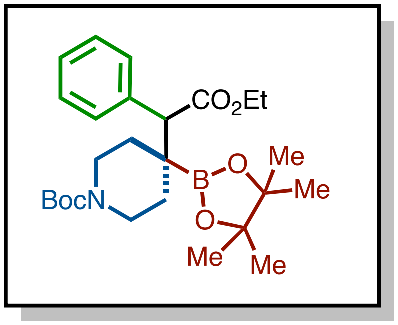

tert-butyl-4-(2-ethoxy-2-oxo-1-phenylethyl)-4-(4,4,5,5-tetramethyl-1,3,2-dioxaborolan-2-yl) piperidine-1-carboxylate **(26)**

The title compound was prepared according to General Procedure A. Purification by silica-gel flash column chromatography (Gradient: Hexane to 7-9% EtOAc:Hexanes) yields 68% (average of two runs) as colorless oil.
^{1}H NMR (500 MHz, CDCl$_3$) δ 7.26 (m, 5H), 4.13 (ddd, *J* = 12.9, 7.2, 4.7 Hz, 1H), 4.06 (dq, *J* = 10.8, 7.0 Hz, 1H), 4.01 – 3.85 (m, 2H), 3.59 (s, 1H), 2.86 (s, 2H), 1.80 (ddd, *J* = 13.4, 11.1, 2.6 Hz, 2H), 1.39 (s, 9H), 1.27 (s, 6H), 1.25 (m, 2H), 1.23 (s, 6H), 1.18 (t, *J* = 7.1 Hz, 3H).
^{13}C NMR (126 MHz, CDCl$_3$) δ 173.8, 155.0, 135.6, 130.5, 127.9, 127.2, 83.6, 79.2, 60.7, 60.2, 33.0, 30.9, 28.5, 25.2, 25.2, 25.0, 21.2, 14.3
^{11}B NMR (160 MHz, CDCl$_3$) δ 34.63
HRMS (ESI): Calculated for $C_{26}H_{40}O_5NBNa$ [M+Na]$^+$:496.2841, Found: 496.2848
IR (Neat): 2976, 1726, 1692, 1423, 1666, 1250, cm^{-1}

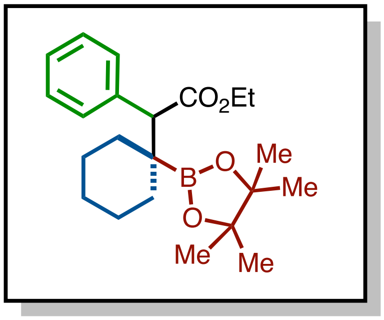

***ethyl-2-phenyl-2-(1-(4,4,5,5-tetramethyl-1,3,2-dioxaborolan-2-yl) cyclohexyl) acetate* (27)**
The title compound was prepared according to General Procedure A. Purification by silica-gel flash column chromatography (Gradient: Hexane to 3-4% EtOAc:Hexanes) yields 60% (average of two runs) as colorless oil.
^{1}H NMR (500 MHz, CDCl$_3$) δ 7.34 – 7.20 (m, 5H), 4.14 (dq, *J* = 11.3, 7.3 Hz, 1H), 4.06 (dq, *J* = 10.9, 7.0 Hz, 1H), 3.55 (s, 1H), 1.92 – 1.79 (m, 2H), 1.64 – 1.52 (m, 3H), 1.48 – 1.34 (m, 2H), 1.28 (s, 6H), 1.24 (s, 6H), 1.19 (t, *J* = 7.1 Hz, 3H), 1.11 (td, *J* = 12.9, 3.7 Hz, 1H), 0.97 (tq, *J* = 12.6, 4.3 Hz, 2H).
^{13}C NMR (126 MHz, CDCl$_3$) δ 174.0, 130.6, 127.6, 127.0, 83.3, 61.1, 60.1, 34.6, 32.3, 26.3, 25.2, 25.2, 25.0, 24.9, 24.7, 14.4
^{11}B NMR (160 MHz, CDCl$_3$) δ 34.79
HRMS (APCI): Calculated for $C_{22}H_{33}O_4BNa$ [M+Na]$^+$:395.2364, Found: 395.2362
IR (Neat): 2925, 1727, 1452, 1306, 1237, 1139 cm^{-1}

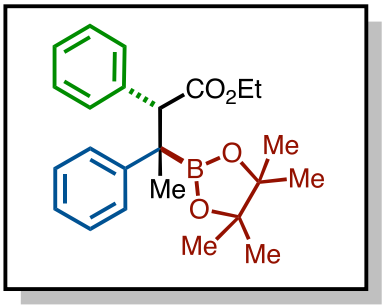

ethyl -2,3-diphenyl-3-(4,4,5,5-tetramethyl-1,3,2-dioxaborolan-2-yl) butanoate **(28)**
The title compound was prepared according to General Procedure A. Purification by silica-gel flash column chromatography (Gradient: Hexane to 2-3% EtOAc:Hexanes) yields with 91% (average of two runs) and 3:1 of dr as colorless oil

^{1}H NMR (500 MHz, CDCl$_3$) δ ***major dr*** 7.25 – 7.20 (m, 4H), 7.17 – 7.08 (m, 2H), 7.05 (t, *J* = 7.6 Hz, 2H), 6.57 (dd, *J* = 7.1, 1.8 Hz, 2H), 4.36 (s, 1H), 4.23 (dq, *J* = 10.8, 7.1 Hz, 1H), 4.13 (dq, *J* = 10.7, 7.1 Hz, 1H), 1.38 (s, 3H), 1.27 (s, 6H), 1.23 (t, *J* = 7.1 Hz, 3H), 1.17 (s, 6H). ***minor dr*** 7.44 (d, *J* = 7.5 Hz, 2H), 7.39 – 7.31 (m, 2H), 7.21 (d, *J* = 7.9 Hz, 4H), 7.12 – 7.09 (m, 2H), 4.44 (s, 1H), 3.87 (dq, *J* = 10.8, 7.1 Hz, 1H), 3.79 (dq, *J* = 10.8, 7.1 Hz, 1H), 1.50 (s, 3H), 1.17 (s, 6H), 1.12 (s, 6H), 0.92 (t, *J* = 7.1 Hz, 3H).

^{13}C NMR (126 MHz, CDCl$_3$) δ ***major dr*** 174.8, 143.7, 135.7, 129.5, 128.0, 127.9, 127.4, 126.6, 125.7, 83.7, 60.7, 59.5, 24.8, 24.6, 15.3, 14.4 ***minor dr*** 173.0, 143.4, 136.8, 130.5, 128.2, 127.7, 127.6, 127.1, 125.8, 83.8, 60.1, 58.2, 24.8, 24.5, 17.4, 14.0

HRMS (APCI+): Calculated for $C_{24}H_{31}O_4BNa$ $[M+Na]^+$:417.2208, Found: 417.2203

IR (Neat): 2977, 1726, 1450, 1318, 1212, 1145 cm^{-1}

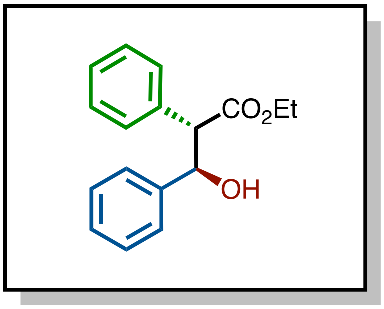

ethyl (2S,3R)-3-hydroxy-2,3-diphenylpropanoate (29)
The title compound was prepared according to General Procedure C. Purification by silica-gel flash column chromatography (Gradient: Hexane to 15-18% EtOAc:Hexanes) yields with 86% (average of two runs) and 11:1 of dr as colorless oil

^{1}H NMR (500 MHz, CDCl$_3$) δ 7.18 (dt, *J* = 6.1, 2.7 Hz, 6H), 7.10 (td, *J* = 6.0, 3.1 Hz, 4H), 5.17 (dd, *J* = 9.2, 3.5 Hz, 1H), 4.24 (dq, *J* = 10.6, 7.1 Hz, 1H), 4.16 (dq, *J* = 10.6, 7.1 Hz, 1H), 3.87 (d, *J* = 9.1 Hz, 1H), 3.22 (d, *J* = 4.1 Hz, 1H), 1.22 (t, *J* = 7.1 Hz, 3H).

^{13}C NMR (126 MHz, CDCl$_3$) δ 173.6, 140.9, 135.5, 128.7, 128.6, 128.2, 127.9, 127.6, 126.7, 76.8, 61.3, 60.1, 14.3

HRMS (APCI+): Calculated for $C_{17}H_{18}O_3Na$ $[M+Na]^+$: 293.1148, Found: 293.1148

IR (Neat): 3350 (br), 2976, 1726, 1615, 1240 cm^{-1}

HPLC: The enantiomeric ratio was **90:10** of the corresponding compound, was determined by HPLC analysis using Phenomenex Cellulose-2 column (95:5 *n*-Hexane/IPA, 1 mL/min, 20 °C, 220 nm, *t*-major = 15.955 min *t*-minor =60.044 min)

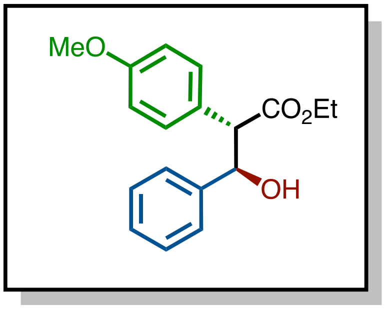

ethyl (2S,3R)-3-hydroxy-2-(4-methoxyphenyl)-3-phenylpropanoate (31)

The title compound was prepared according to General Procedure C. Purification by silica-gel flash column chromatography (Gradient: Hexane to 15-18% EtOAc:Hexanes) yields with 65% (average of two runs) and >20:1 of dr as colorless oil

^{1}H NMR (500 MHz, CDCl$_3$) δ 7.23 – 7.15 (m, 3H), 7.15 – 7.08 (m, 2H), 7.04 – 6.97 (m, 2H), 6.75 – 6.68 (m, 2H), 5.13 (dd, *J* = 9.2, 2.9 Hz, 1H), 4.30 – 4.19 (m, 1H), 4.15 (dq, *J* = 10.8, 7.1 Hz, 1H), 3.81 (d, *J* = 9.2 Hz, 1H), 3.73 (s, 3H), 3.12 (d, *J* = 4.1 Hz, 1H), 1.22 (t, *J* = 7.1 Hz, 3H).

^{13}C NMR (126 MHz, CDCl$_3$) δ 173.8, 158.9, 141.1, 129.7, 128.2, 127.8, 127.6, 126.8, 113.9, 76.8, 61.3, 59.2, 55.3, 14.2

HRMS (ESI): Calculated for $C_{18}H_{20}O_3Na$ $[M+Na]^+$: 307.1304, Found: 307.1301

IR (Neat): 3480 (br), 3033, 2930, 1728, 1611, 1584, 1424 cm^{-1}

HPLC: The enantiomeric ratio was **90.5:9.5** of the corresponding compound, was determined by HPLC analysis using Phenomenex Cellulose-1 column (90:10 *n*-Hexane/IPA, 1 mL/min, 20 °C, 220 nm, *t*-minor =10.475 min *t*-major = 12.381 min)

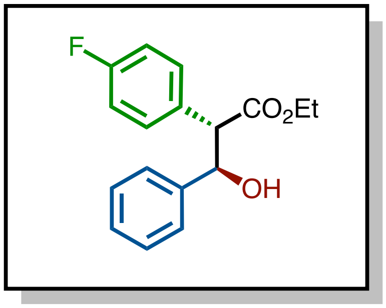

ethyl (2S,3R)-2-(4-fluorophenyl)-3-hydroxy-3-phenylpropanoate (32)

The title compound was prepared according to General Procedure C. Purification by silica-gel flash column chromatography (Gradient: Hexane to 15-18% EtOAc:Hexanes) yields with 65% (average of two runs) and 15:1 of dr as colorless oil

^{1}H NMR (500 MHz, CDCl$_3$) δ 7.19 (qd, *J* = 4.5, 1.5 Hz, 3H), 7.16 – 7.02 (m, 4H), 6.91 – 6.82 (m, 2H), 5.13 (d, *J* = 9.2 Hz, 1H), 4.28 – 4.20 (m, 1H), 4.20 – 4.13 (m, 1H), 3.85 (d, *J* = 9.3 Hz, 1H), 3.10 (s, 1H), 1.23 (t, *J* = 7.2 Hz, 3H).
^{13}C NMR (126 MHz, CDCl$_3$) δ 173.4, 162.2 (d, *J* = 246.1 Hz), 140.7, 131.3 (d, *J* = 3.2 Hz), 130.3 (d, *J* = 8.2 Hz), 128.3, 128.0, 126.7, 115.5 (d, *J* = 21.3 Hz), 76.8, 61.5, 59.3, 14.2
^{19}F NMR (471 MHz, CDCl$_3$) δ -114.76 (td, *J* = 9.0, 4.8 Hz)
HRMS (APCI+): Calculated for $C_{17}H_{17}FO_3Na$ $[M+Na]^+$: 311.1053, Found: 311.1048
IR (Neat): 3466 (br), 3031, 2922, 1730, 1605, 1511, 1455 cm^{-1}
HPLC: The enantiomeric ratio was **89:11** of the corresponding compound, was determined by HPLC analysis using Phenomenex Cellulose-1 column (90:10 *n*-Hexane/IPA, 1 mL/min, 20 °C, 220 nm, *t*-minor = 8.344 min *t*-major =11.386 min)

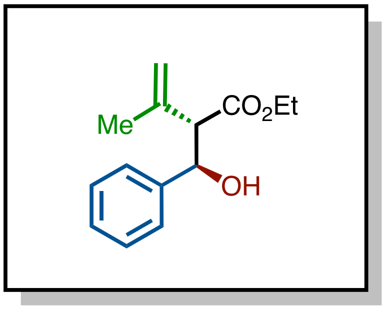

ethyl (S)-2-((R)-hydroxy(phenyl)methyl)-3-methylbut-3-enoate (33)
The title compound was prepared according to General Procedure C. Purification by silica-gel flash column chromatography (Gradient: Hexane to 12-17% EtOAc:Hexanes) yields with 60% (average of two runs) and 10:1 of dr as colorless oil
^{1}H NMR (500 MHz, CDCl$_3$) δ 7.38 – 7.24 (m, 5H), 5.03 (dd, *J* = 8.8, 4.3 Hz, 1H), 4.84 (dd, *J* = 4.0, 2.5 Hz, 2H), 4.20 (qd, *J* = 7.1, 1.6 Hz, 2H), 3.37 (d, *J* = 8.8 Hz, 1H), 3.11 (d, *J* = 4.5 Hz, 1H), 1.58 (t, *J* = 1.2 Hz, 3H), 1.25 (t, *J* = 7.1 Hz, 3H).
^{13}C NMR (126 MHz, CDCl$_3$) δ 173.4, 141.2, 139.7, 128.4, 128.1, 126.9, 115.8, 74.7, 61.2, 60.5, 22.1, 14.2
HRMS (APCI+): Calculated for $C_{14}H_{18}O_3Na$ $[M+Na]^+$: 257.1148, Found: 257.1143
IR (Neat): 3460 (br), 3063, 2923, 2853,1732, 1645, 1454 cm^{-1}
HPLC: The enantiomeric ratio was **87.5:12.5** of the corresponding compound, was determined by HPLC analysis using Chiralpak IA-3 column (95:5 *n*-Hexane/IPA, 1 mL/min, 20 °C, 220 nm, *t*-minor = 13.248 min *t*-major =14.391 min)

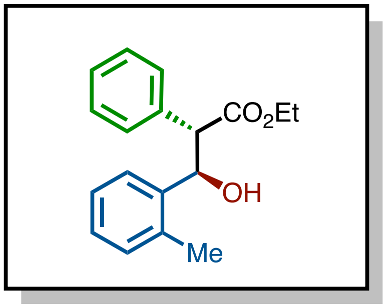

ethyl (2S,3R)-3-hydroxy-2-phenyl-3-(o-tolyl) propanoate (34)

The title compound was prepared according to General Procedure C. Purification by silica-gel flash column chromatography (Gradient: Hexane to 10-14% EtOAc:Hexanes) yields with 53% (average of two runs) and 6:1 of dr as colorless oil

^{1}H NMR (500 MHz, CDCl$_3$) δ 7.49 (dd, J = 7.7, 1.4 Hz, 1H), 7.22 – 7.13 (m, 4H), 7.12 – 7.03 (m, 3H), 6.92 (dd, J = 7.4, 1.4 Hz, 1H), 5.43 (dd, J = 9.1, 4.1 Hz, 1H), 4.26 (dq, J = 10.8, 7.1 Hz, 1H), 4.18 (dq, J = 10.8, 7.1 Hz, 1H), 3.96 (d, J = 9.1 Hz, 1H), 3.17 (d, J = 4.3 Hz, 1H), 1.90 (s, 3H), 1.23 (t, J = 7.2 Hz, 3H).

^{13}C NMR (126 MHz, CDCl$_3$) δ 173.8, 139.2, 135.7, 135.1, 130.2, 128.6, 128.5, 127.7, 127.6, 126.5, 126.3, 72.5, 61.4, 59.5, 19.1, 14.2

HRMS (ESI): Calculated for $C_{18}H_{20}O_3Na$ [M+Na]$^+$: 307.1304, Found: 307.1301

IR (Neat): 3495 (br), 3029, 2923, 2853,1730, 1603, 1455 cm^{-1}

HPLC: The enantiomeric ratio was **94:6 (major dr)** and **90.5:9.5 (minor dr)** of the corresponding compound determined by HPLC analysis using Phenomenex Cellulose-2 column (95:5 *n*-Hexane/IPA, 1 mL/min, 20 °C, 220 nm, *t*-major = 7.946 min *t*-minor =27.420 min).

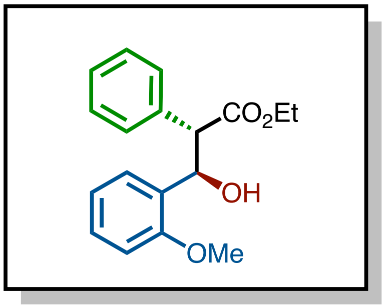

ethyl (2S,3R)-3-hydroxy-3-(2-methoxyphenyl)-2-phenylpropanoate (35)

The title compound was prepared according to General Procedure C. Purification by silica-gel flash column chromatography (Gradient: Hexane to 6-10% EtOAc:Hexanes) yields with 70% (average of two runs) and 7:1 of dr as colorless oil

^{1}H NMR (500 MHz, CDCl$_3$) δ 7.22 – 7.13 (m, 6H), 7.10 (dd, J = 7.5, 1.7 Hz, 1H), 6.84 – 6.74 (m, 2H), 5.37 (t, J = 8.2 Hz, 1H), 4.32 – 4.03 (m, 3H), 3.75 (s, 3H), 3.70 (d, J = 8.2 Hz, 1H), 1.21 (t, J = 7.1 Hz, 3H).

^{13}C NMR (126 MHz, CDCl$_3$) δ 173.4, 156.6, 136.1, 129.0, 128.8, 128.5, 128.4, 128.3, 127.4, 120.7, 110.5, 73.8, 61.1, 58.1, 55.3, 14.2

HRMS (ESI): Calculated for $C_{18}H_{20}O_4Na$ [M+Na]$^+$: 323.1253, Found: 323.1241

IR (Neat): 3490 (br), 3056, 2925, 2832,1735, 1625, 1450 cm^{-1}

HPLC: The enantiomeric ratio was **90:10** of the corresponding compound determined by HPLC analysis using Chiralpak IA-3 column (80:20 *n*-Hexane/IPA, 1 mL/min, 20 °C, 220 nm, *t*-major = 11.095 min *t*-minor =14.314 min).

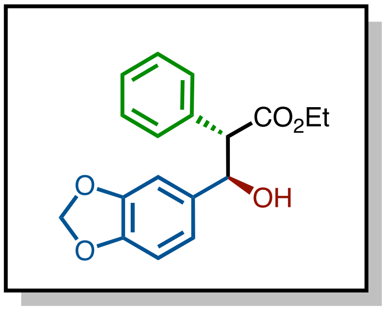

ethyl (2S,3R)-3-(benzo[d][1,3] dioxol-5-yl)-3-hydroxy-2-phenylpropanoate (36)

The title compound was prepared according to General Procedure C. Purification by silica-gel flash column chromatography (Gradient: Hexane to 15-20% EtOAc:Hexanes) yields with 73% (average of two runs) and >20:1 of dr as colorless oil

^{1}H NMR (500 MHz, CDCl$_3$) δ 7.24 – 7.15 (m, 3H), 7.14 – 7.07 (m, 2H), 6.71 (d, *J* = 1.6 Hz, 1H), 6.58 (d, *J* = 7.9 Hz, 1H), 6.48 (dd, *J* = 8.0, 1.8 Hz, 1H), 5.91 – 5.85 (m, 2H), 5.09 (dd, *J* = 9.4, 3.8 Hz, 1H), 4.24 (dq, *J* = 10.8, 7.1 Hz, 1H), 4.15 (dq, *J* = 10.9, 7.1 Hz, 1H), 3.82 (d, *J* = 9.3 Hz, 1H), 3.10 (d, *J* = 4.0 Hz, 1H), 1.22 (t, *J* = 7.1 Hz, 3H).

^{13}C NMR (126 MHz, CDCl$_3$) δ 173.6, 147.6, 147.1, 135.5, 134.9, 128.6, 128.6, 127.6, 120.5, 107.9, 107.0, 101.1, 76.6, 61.4, 60.1, 14.2

HRMS (ESI+): Calculated for $C_{18}H_{18}O_5Na$ $[M+Na]^+$: 337.1046, Found: 337.1038

IR (Neat): 3437 (br), 2977, 2856,1732, 1688, 1477 cm^{-1}

HPLC: The enantiomeric ratio was **90:10** of the corresponding compound, was determined by HPLC analysis using Chiralpak IA-3 column (85:15 *n*-Hexane/IPA, 1 mL/min, 20 °C, 220 nm, *t*-major = 12.861 min *t*-minor =17.953 min)

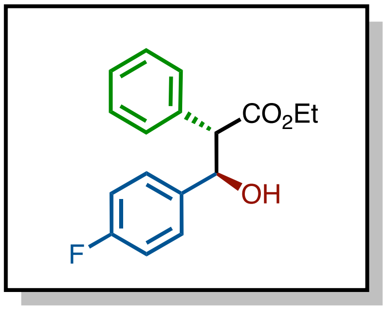

ethyl (2S,3R)-3-(4-fluorophenyl)-3-hydroxy-2-phenylpropanoate (37)

The title compound was prepared according to General Procedure C. Purification by silica-gel flash column chromatography (Gradient: Hexane to 10-14% EtOAc:Hexanes) yields with 65% (average of two runs) and 15:1 of dr as colorless oil

^{1}H NMR (500 MHz, CDCl$_3$) δ 7.23 – 7.15 (m, 3H), 7.05 (ddd, *J* = 7.8, 5.4, 2.3 Hz, 4H), 6.91 – 6.82 (m, 2H), 5.16 (dd, *J* = 9.3, 3.8 Hz, 1H), 4.24 (dq, *J* = 10.8, 7.1 Hz, 1H), 4.16 (dq, *J* = 10.8, 7.1 Hz, 1H), 3.80 (d, *J* = 9.3 Hz, 1H), 3.21 (d, *J* = 4.0 Hz, 1H), 1.22 (t, *J* = 7.1 Hz, 3H).

^{13}C NMR (126 MHz, CDCl$_3$) δ 173.6, 162.3 (d, *J* = 245.9 Hz), 136.70, 136.71, 135.3, 128.7 (d, *J* = 6.6 Hz), 128.4 (d, *J* = 8.2 Hz), 127.7, 115.1 (d, *J* = 21.5 Hz), 76.1, 61.5, 60.3, 14.2

^{19}F NMR (471 MHz, CDCl$_3$) δ -114.56 (dt, *J* = 14.2, 7.5 Hz).

HRMS (ESI+): Calculated for $C_{17}H_{17}FO_3Na$ $[M+Na]^+$: 311.1053, Found: 311.1048

IR (Neat): 3464 (br), 3032, 2983, 2926,1728, 1605, 1411 cm^{-1}
HPLC: The enantiomeric ratio was **89:11** of the corresponding compound, was determined by HPLC analysis using Chiralpak IA-3 column (90:10 *n*-Hexane/IPA, 1 mL/min, 20 °C, 220 nm, *t*-minor =11.499 min *t*-major = 13.001 min)

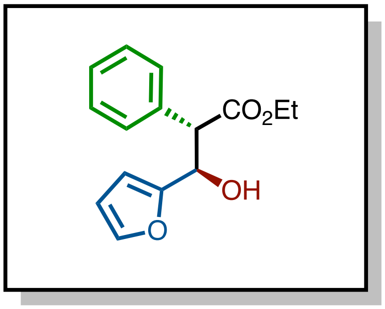

ethyl (2S,3R)-3-(furan-2-yl)-3-hydroxy-2-phenylpropanoate (38)
The title compound was prepared according to General Procedure C. Purification by silica-gel flash column chromatography (Gradient: Hexane to 10-14% EtOAc:Hexanes) yields with 81% (average of two runs) and 8:1 of dr as colorless oil
^{1}H NMR (500 MHz, $CDCl_3$) δ 7.33 – 7.29 (m, 1H), 7.28 – 7.18 (m, 5H), 6.18 (dd, *J* = 3.3, 1.8 Hz, 1H), 6.05 (d, *J* = 3.2 Hz, 1H), 5.19 (dd, *J* = 8.8, 5.9 Hz, 1H), 4.30 – 4.20 (m, 1H), 4.16 (ddt, *J* = 10.8, 7.1, 3.2 Hz, 2H), 3.27 (d, *J* = 5.9 Hz, 1H), 1.22 (t, *J* = 7.1 Hz, 3H).
^{13}C NMR (126 MHz, $CDCl_3$) δ 173.2, 153.4, 142.3, 135.3, 128.7, 128.3, 127.8, 110.4, 108.1, 70.2, 61.1, 56.7, 14.3
HRMS (ESI): Calculated for $C_{15}H_{16}O_4Na$ $[M+Na]^+$: 283.0940, Found: 283.0935
IR (Neat): 3468 (br), 3032, 2925,1730, 1603, 1498, 1455 cm^{-1}
HPLC: The enantiomeric ratio was **87.5:12.5** of the corresponding compound, was determined by HPLC analysis using Chiralpak IA column (90:10 *n*-Hexane/IPA, 1 mL/min, 20 °C, 220 nm, *t*-minor =12.398 min *t*-major = 15.580 min)

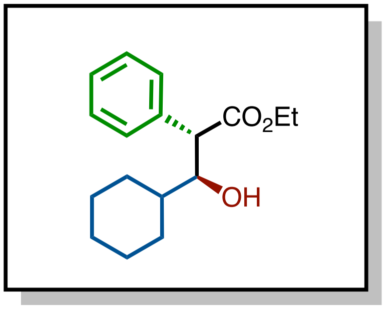

ethyl (2S,3S)-3-cyclohexyl-3-hydroxy-2-phenylpropanoate (39)
The title compound was prepared according to General Procedure C. Later a H_2O_2/NaOH oxidation performed instead of perborate oxidation. Purification by silica-gel flash column chromatography (Gradient: Hexane to 10-12% EtOAc:Hexanes) yields with 65% (average of two runs) and 9:1 of dr as colorless oil
^{1}H NMR (500 MHz, $CDCl_3$) δ 7.35 – 7.26 (m, 5H), 4.19 (dq, *J* = 10.8, 7.1 Hz, 1H), 4.09 (dq, *J* = 10.8, 7.1 Hz, 1H), 3.97 (ddd, *J* = 8.8, 5.9, 2.9 Hz, 1H), 3.78 (d, *J* = 8.7 Hz, 1H), 2.66 (d, *J* = 6.0

Hz, 1H), 1.78 – 1.64 (m, 3H), 1.62 – 1.57 (m, 1H), 1.54 – 1.45 (m, 1H), 1.41 – 1.28 (m, 1H), 1.20 (t, J = 7.1 Hz, 3H), 1.17 – 0.96 (m, 5H).
^{13}C NMR (126 MHz, CDCl$_3$) δ 174.1, 136.6, 128.9, 128.5, 127.6, 61.2, 55.1, 39.7, 30.6, 26.5, 26.2, 25.6, 14.2
HRMS (ESI): Calculated for $C_{17}H_{24}O_3Na$ [M+Na]$^+$:299.1617, Found: 299.1609
IR (Neat): 3455 (br), 2960, 1728, 1665, 1611, 1330 cm^{-1}
HPLC: The enantiomeric ratio was **90.5:9.5** of the corresponding compound, was determined by HPLC analysis using Chiralpak IA-3 column (90:10 *n*-Hexane/IPA, 1 mL/min, 20 °C, 220 nm, *t*-major = 6.831 min *t*-minor =9.715 min)

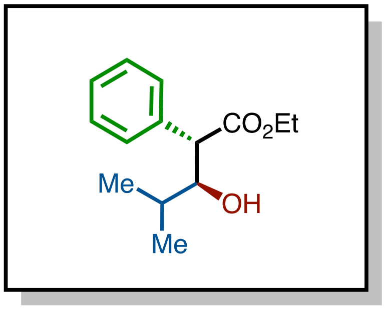

ethyl (2S,3S)-3-hydroxy-4-methyl-2-phenylpentanoate (40)

The title compound was prepared according to General Procedure C. Later a H_2O_2/NaOH oxidation performed instead of perborate oxidation. Purification by silica-gel flash column chromatography (Gradient: Hexane to 10-12% EtOAc:Hexanes) yields with 69% (average of two runs) and 4:1 of dr as colorless oil
^{1}H NMR (500 MHz, CDCl$_3$) δ 7.36 – 7.26 (m, 5H), 4.19 (dq, J = 10.9, 7.1 Hz, 1H), 4.13 – 4.07 (m, 1H), 4.07 – 4.00 (m, 1H), 3.71 (d, J = 9.2 Hz, 1H), 2.61 (d, J = 5.3 Hz, 1H), 1.46 (m, 1H), 1.20 (t, J = 7.2 Hz, 3H), 0.94 (d, J = 6.9 Hz, 3H), 0.88 (d, J = 6.8 Hz, 3H).
^{13}C NMR (126 MHz, CDCl$_3$) δ 174.0, 136.5, 128.9, 128.5, 127.7, 77.6, 61.2, 56.0, 29.4, 20.4, 14.7, 14.2
HRMS (ESI): Calculated for $C_{14}H_{20}O_3Na$ [M+Na]$^+$:, 259.1304, Found: 259.1296
IR (Neat): 3455 (br), 2986, 1735, 1745, 1666, 1350 cm^{-1}
HPLC: The enantiomeric ratio was **90.5:9.5** of the corresponding compound, was determined by HPLC analysis using Chiralpak IA column (95:5 *n*-Hexane/IPA, 0.5 mL/min, 20 °C, 220 nm, *t*-major = 16.514 min *t*-minor =18.636 min).

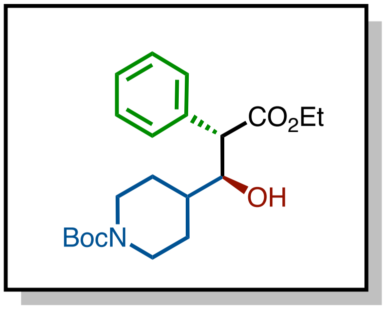

tert-butyl 4-((1S,2S)-3-ethoxy-1-hydroxy-3-oxo-2-phenylpropyl) piperidine-1-carboxylate (41)

The title compound was prepared according to General Procedure C. Purification by silica-gel flash column chromatography (Gradient: Hexane to 20-30% EtOAc:Hexanes) yields with 59% (average of two runs) and 20:1 of dr as colorless oil

^{1}H NMR (500 MHz, $CDCl_3$) δ 7.40 – 7.27 (m, 5H), 4.24 – 3.98 (m, 5H), 3.75 (d, *J* = 8.7 Hz, 1H), 2.80 (d, *J* = 5.9 Hz, 1H), 2.65 – 2.35 (m, 2H), 1.66 (dt, *J* = 12.9, 2.9 Hz, 1H), 1.53 (td, *J* = 12.5, 4.5 Hz, 1H), 1.43 (s, 11H), 1.30 – 1.25 (m, 1H), 1.19 (t, *J* = 7.1 Hz, 3H).

^{13}C NMR (126 MHz, $CDCl_3$) δ 173.9, 154.9, 136.2, 129.1, 128.4, 127.9, 79.4, 76.5, 61.3, 55.0, 38.2, 29.4, 28.6, 25.0, 14.2

HRMS (ESI): Calculated for $C_{21}H_{31}O_5NNa$ $[M+Na]^+$:400.2094, Found: 400.2096

IR (Neat): 3470 (br), 2977, 1731, 1709, 1691, 1280 cm^{-1}

HPLC: The enantiomeric ratio was **92:8** of the corresponding compound, was determined by HPLC analysis using Phenomenex Cellulose-2 column (90:10 *n*-Hexane/IPA, 1 mL/min, 20 °C, 220 nm, *t*-minor = 13.335 min *t*-major =17.372 min).

7. Unsuccessful substrates:

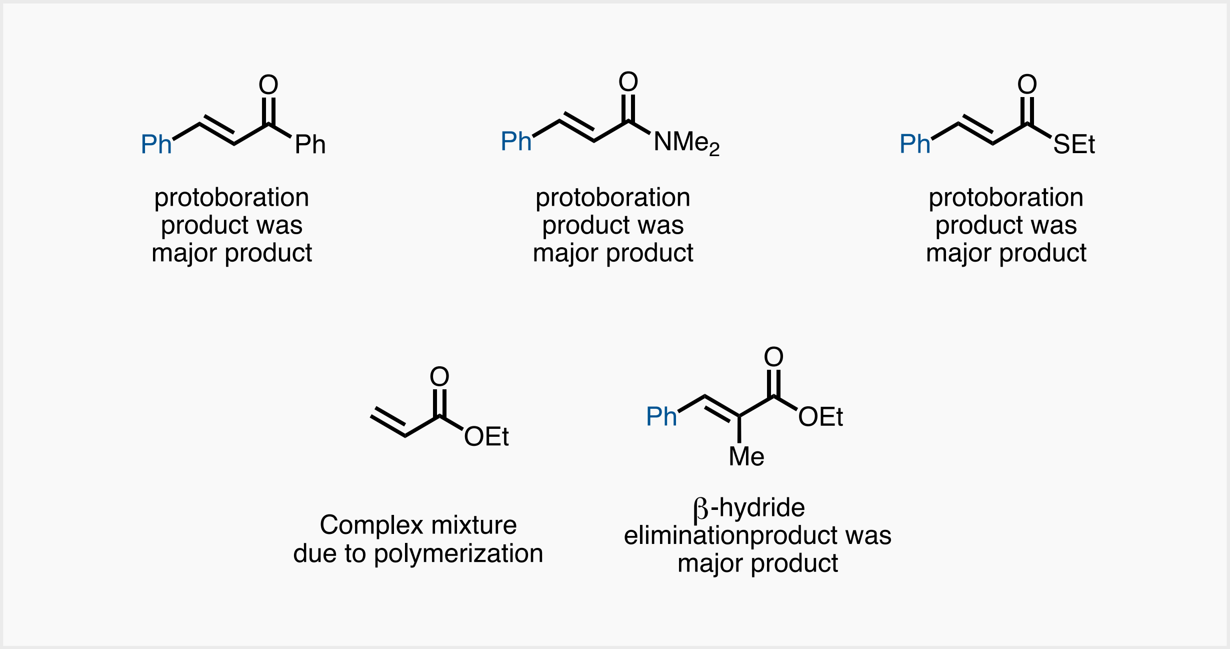

8. Control experiments:

^{11}BNMR to detect the chelate intermediate:

To explain the diastereoselectivity of the reaction we are proposing a chelate intermediate that might be the actual reason for higher dr. To investigate the hypothesis for the chelate formation we have conducted couple of control experiments.

(A) **Stoichiometric reaction to make alkyl-CuL complex:**

In flame dried 13x100 mm screw cap vial with a magnetic stir bar, was taken into a N_2 filled glovebox, was added SIMesCuCl (31.75 mg, 50.0 µmol, 1.0 eq.), B_2pin_2 (19.05 mg, 75.0 µmol, 1.5 eq.) and NaO*t*-Bu (6.25 mg, 65.0 µmol, 1.3 eq.) in this sequence. Further, the vial was capped with a septum and sealed using Teflon and taken out from the glovebox. Then (E)-ethyl cinnamate (8.5 µL, 50.0 µmol, 1.0 eq.) was added flowed by 0.5 mL of toluene-d_8 solvent. Then the reaction mixture was allowed to stir at room temperature for 30 min. Then after 30 min half of reaction mixture carefully taken out via syringe and added in an NMR tube under N_2 atmosphere and checked the ^{11}BNMR.

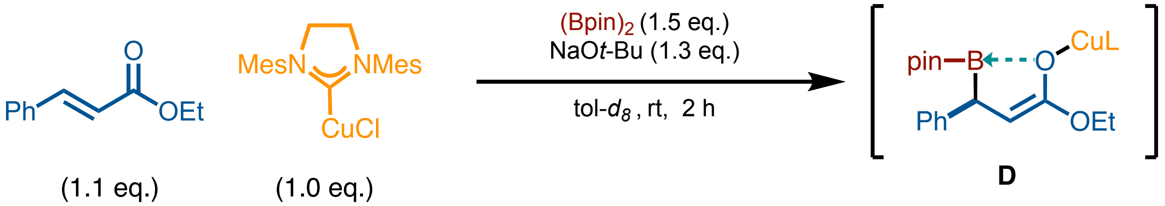

^{11}BNMR analysis suggest that this chelate is a short lived intermediate and shows small peak in the range between δ 4-11 ppm (see figure below). It is possible the peak at δ 4.65 ppm or δ 10.93 ppm might be associated with the chelate (D).

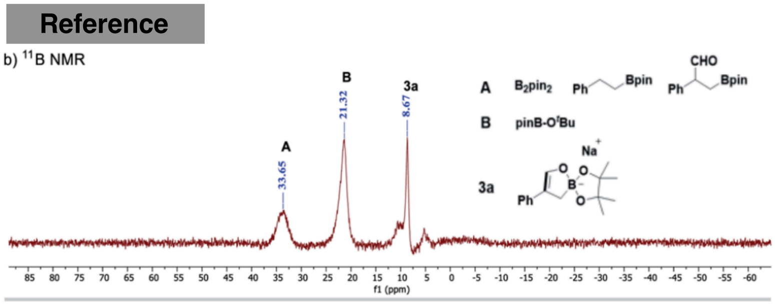

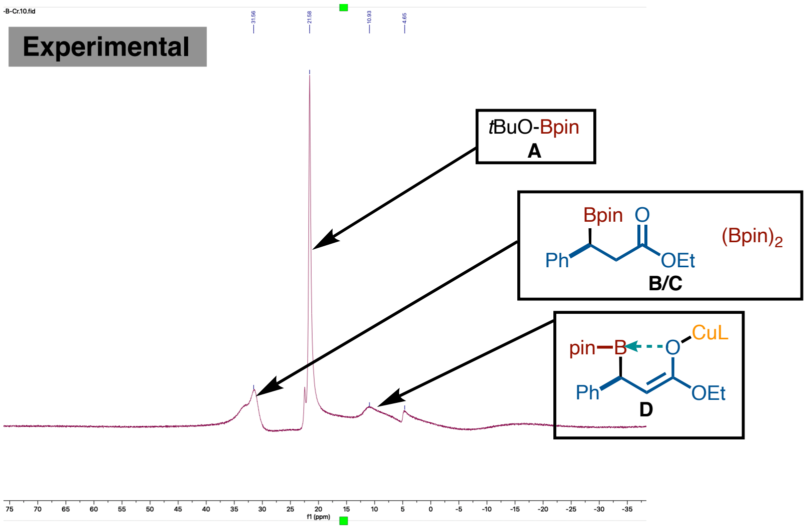

(B) **Reaction with -CN substrate**

In flame dried 13x100 mm screw cap vial with a magnetic stir bar, was taken into a N_2 filled glovebox, was added APhosPdG3 (3.2 mg, 5.0 µmol, 2.0 mol%), SIMesCuCl (5.1 mg, 13 µmol, 5.0 mol%), B_2pin_2 (95 mg, 0.38 mmol, 1.5 eq.), NaOtBu (31 mg, 0.33 mmol, 1.3 eq.) was added, in that order. Then the vial was sealed with a rubber septum and lined with Teflon tape, removed from the glove box, and placed under a positive pressure of N_2. To the vial was added 1.5 mL of toluene: THF (10:1), (E)-3-phenylacrylonitrile (0.25 mmol, 1.0 eq.), and the bromobenzene (0.38 mmol, 1.5 eq.) before rinsing the sides of the vial with another 1.0 mL of toluene: THF (10:1). The septum was then quickly replaced with a Teflon lined screw cap and the reaction was stirred at 30°C for 18 hours. After 18 hours, the reaction was quenched with aqueous water (2.5 mL), the two phases were separated, and the aqueous phase was back extracted with EtOAc (2 × 3 mL). The combined organic phases were dried with anhydrous Sodium sulfate and concentrated in-vacuo.

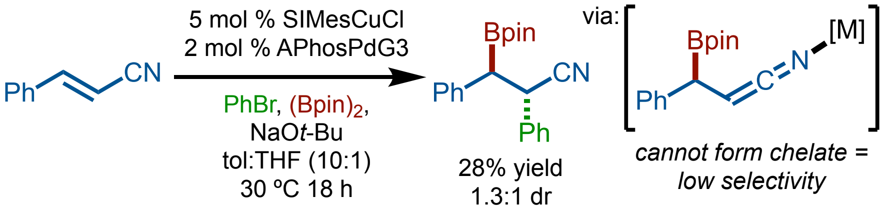

From crude NMR and GCMS analysis found that arylboration product formed with 28% NMR yield and 1.3:1 dr which suggest that probably due to lack of formation of stable chelate the selectivity is lower with -CN substrate.

9. (a) Gram scale synthesis: Glovebox free setup

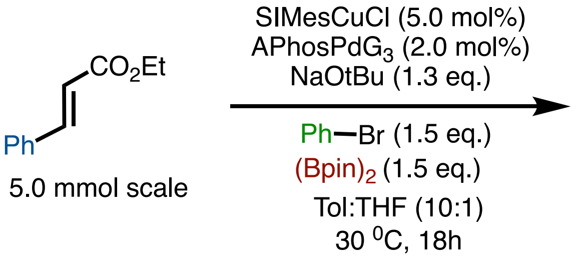

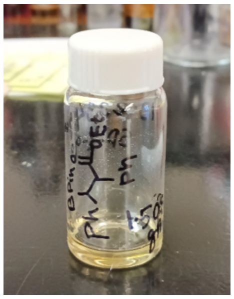

The title compound was prepared according to General Procedure B (Glovebox free condition). In a flame-dried 100 mL round-bottom flask with a magnetic stir bar, was added APhosPdG3 (63.5 mg, 100 µmol, 2.0 mol%), SIMesCuCl (102 mg, 250 µmol, 5.0 mol%), B_2pin_2 (1.9 g, 7.5 mmol, 1.5 eq.), NaOtBu (625 mg, 6.5 mmol, 1.30 eq.) were added. The round-bottom flask was put under a N_2 atmosphere via standard Schlenk technique evacuated and backfilled with N_2 three times. The round-bottom flask was removed from the Schlenk line and quickly capped with a septum and a N_2 line. 30 mL of toluene:THF (10:1) solution added via syringe, bromobenzene (0.79mL, 7.5 mmol, 1.5 eq.) was immediately added via syringe followed by ethyl cinnamate (0.839 mL, 5.00 mmol, 1.0 eq.) via syringe. *(Note: it is important that the round bottom flask is stirring vigorously while the liquid reagents are added to prohibit aggregation of the solid reagents, which typically results in lower yields).* The N_2 line was removed, and two pieces of electrical tape were used to cover the puncture sites of the septum. The mixture stirred for 18 hours at 30 ^{0}C. Then the reaction was quenched with the addition of 50 mL water. The mixture was extracted with EtOAc (3 x 30 mL). The combined organic layers were collected dried over anhydrous Sodium sulfate and concentrated in vacuo. Purification by silica gel column chromatography (R_f= 0.45 in 10% EtOAc:Hexane) 5-7% EtOAc in hexanes gave 1.5 g **(2)** product as a colorless oil which further solidified overnight in the freezer with 82% yield (avg. yield of two runs) and >20:1 dr

Further functionalization:

(b) Oxidation of C-B bond:

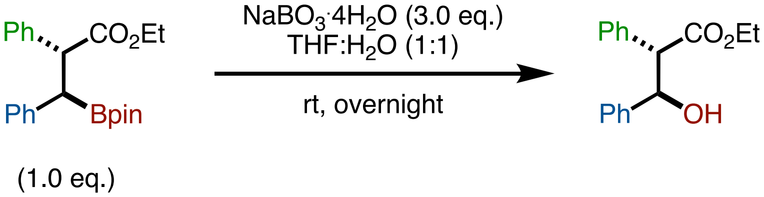

ethyl-3-hydroxy-2,3-diphenylpropanoate (rac-29)

The arylboration product ethyl-2,3-diphenyl-3-(4,4,5,5-tetramethyl-1,3,2-dioxaborolan-2-yl) propanoate (0.2 mmol) was taken in a 10 mL round-bottom flask, dissolved in THF (1.0 mL) and $NaBO_3·4H_2O$ (90.0 mg, 0.58 mmol, 3.0 eq.) was added, followed by addition of water (1.0 mL). The reaction was allowed to stir at room temperature overnight. Then the reaction mixture was

quenched upon the addition of aqueous sat. $Na_2S_2O_3$ (3 mL) and extracted with EtOAc (3 x 2 mL). The combined organic layers were washed with 2 M HCl (3 x 3 mL), dried over anhydrous sodium sulfate, and concentrated in vacuo. The residue was purified via silica gel column chromatography (R_f= 0.35 in 20% EtOAc:Hexane) 16%-20% EtOAc in hexanes to afford the desired product with 65% yield and >20:1 dr.

^{1}H NMR (500 MHz, $CDCl_3$) δ 7.18 (dt, *J* = 6.1, 2.7 Hz, 6H), 7.10 (td, *J* = 6.0, 3.1 Hz, 4H), 5.17 (dd, *J* = 9.2, 3.5 Hz, 1H), 4.24 (dq, *J* = 10.6, 7.1 Hz, 1H), 4.16 (dq, *J* = 10.6, 7.1 Hz, 1H), 3.87 (d, *J* = 9.1 Hz, 1H), 3.22 (d, *J* = 4.1 Hz, 1H), 1.22 (t, *J* = 7.1 Hz, 3H).

^{13}C NMR (126 MHz, $CDCl_3$) δ 173.6, 140.9, 135.5, 128.7, 128.6, 128.2, 127.9, 127.6, 126.7, 76.8, 61.3, 60.1, 14.3

HRMS (APCI+): Calculated for $C_{17}H_{18}O_3Na$ $[M+Na]^+$: 293.1148, Found: 293.1148

IR (Neat): 3350 (br), 2976, 1726, 1615, 1240 cm^{-1}

(c) Homologation of C-B bond:

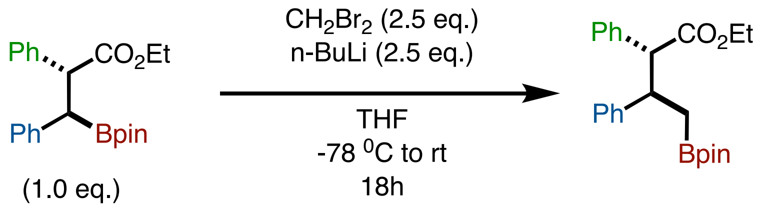

ethyl-2,3-diphenyl-4-(4,4,5,5-tetramethyl-1,3,2-dioxaborolan-2-yl) butanoate **(45)**

The product was synthesized according to the following modified literature procedure.[23] In a flame-dried 25-mL round-bottom flask equipped with a stir bar was charged with ethyl-2,3-diphenyl-3-(4,4,5,5-tetramethyl-1,3,2-dioxaborolan-2-yl) propanoate (190 mg, 0.5 mmol, 1.0 eq.) and via standard Schlenk technique evacuated and backfilled with N_2 x3. Then THF (5.0 mL) was added and CH_2Br_2 (87.2 µL, 1.25 mmol, 2.5 eq.) were added sequentially via syringe and the mixture was cooled to -78 ^{0}C in a dry ice/acetone bath. *n*-BuLi (0.45 mL, 2.25 M in hexanes, 1.1 mmol, 2.2 eq.) was added dropwise via syringe over 5 minutes. The reaction was stirred at -78 °C for one hour and then warmed to room temperature and stirred for 18 hours. The reaction was quenched with addition of 5 mL water, the organic layers were separated, the aqueous layer was extracted with ethyl acetate (5x3 mL). The combined organic layers were dried over anhydrous Sodium sulfate, concentrated in vacuo. The residue was purified via silica gel column chromatography (R_f= 0.5 in 10% EtOAc:Hexane) 4%-8% EtOAc in hexanes to afford the desired product with 63% yield and >20:1 dr.

^{1}H NMR (500 MHz, $CDCl_3$) δ 7.17-7.11 (m, 2H), 7.10 – 7.03 (m, 5H), 7.01 – 6.94 (m, 3H), 4.21 (dq, *J* = 10.9, 7.1 Hz, 1H), 4.10 (dq, *J* = 10.9, 7.1 Hz, 1H), 3.69 (d, *J* = 10.9 Hz, 1H), 3.56 (td, *J* = 10.6, 5.8 Hz, 1H), 1.37 – 1.29 (m, 2H), 1.24 (t, *J* = 7.1 Hz, 3H), 0.98 (s, 6H), 0.94 (s, 6H).

^{13}C NMR (126 MHz, $CDCl_3$) δ 173.7, 143.1, 137.8, 128.8, 128.4, 128.0, 127.8, 126.9, 126.1, 83.1, 60.8, 60.8, 45.5, 24.7, 24.5, 14.3

^{11}B NMR (160 MHz, $CDCl_3$) δ 33.95

HRMS (APCI+): Calculated for $C_{24}H_{31}O_4BNa$ $[M+Na]^+$: 417.2208, Found: 417.2207
IR (Neat): 2978, 1730, 1368, 1329, 1245, 1145 cm^{-1}

(d) Zweifel olefination:

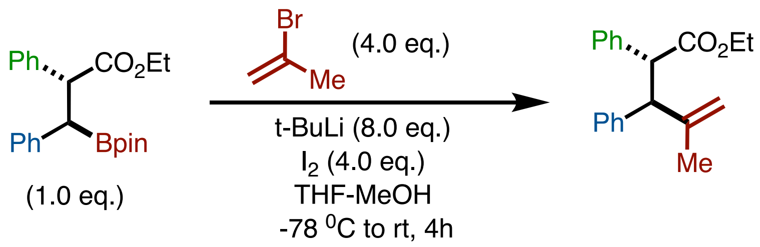

***ethyl 4-methyl-2,3-diphenylpent-4-enoate* (46)**

The product was synthesized according to the following modified literature procedure.[24] In a flame-dried 2-dram vial under N_2-atmosphere was charged with 2-bromoprop-1-ene (36 µL, 0.4 mmol, 4.0 eq.) was added in 2.0 mL of THF and the solution was cooled to -78 °C in a dry ice/acetone bath. *t*-BuLi (0.53 mL, 1.5 M in pentane, 0.8 mmol, 8.0 eq.) was added dropwise and the solution stirred at -78 °C for 1 hour. A solution of ethyl 2,3-diphenyl-3-(4,4,5,5-tetramethyl-1,3,2-dioxaborolan-2-yl) propanoate (38.0 mg, 0.10 mmol, 1.0 eq.) in THF (0.5 mL) was added dropwise at -78 °C and the mixture was allowed to stir at -78 °C for 3 h. A solution of Iodine (I_2) (102 mg, 0.402 mmol, 4.0 eq.) in MeOH (0.4 mL) was added dropwise down the side of the flask and the reaction was stirred at -78 °C for 30 minutes, then allowed to warm to room temperature and stirred for 1 h. The reaction was quenched upon the addition of $Na_2S_2O_3$ (sat. solution in H_2O, 3.0 mL) and stirred for another 1 hour. The organic layer was separated, and the aqueous phase extracted with EtOAc (3 x 5.0 mL). The combined organic layers were washed with brine (10 mL), dried over anhydrous Sodium sulfate, and concentrated under reduced pressure. The residue was purified via silica gel column chromatography (R_f= 0.5 in 10% EtOAc:Hexane) 5%-6% EtOAc in hexanes to afford the desired product with 65% yield and >20:1 dr.

^{1}H NMR (500 MHz, $CDCl_3$) δ 7.16 – 7.00 (m, 8H), 7.01 – 6.95 (m, 2H), 5.15 (s, 1H), 4.94 (t, *J* = 1.4 Hz, 1H), 4.20 (dq, *J* = 10.8, 7.1 Hz, 1H), 4.13 – 4.00 (m, 2H), 3.92 (d, *J* = 12.2 Hz, 1H), 1.70 (s, 3H), 1.22 (t, *J* = 7.1 Hz, 3H).

^{13}C NMR (126 MHz, $CDCl_3$) δ 173.3, 147.4, 139.9, 137.2, 129.1, 128.6, 128.3, 128.0, 127.2, 126.5, 110.1, 60.9, 56.0, 56.0, 22.9, 14.2

HRMS (EI+): Calculated for $C_{20}H_{22}O_2$ $[M]^+$: 294.1615, Found: 294.1614
IR (Neat): 2978, 1731, 1452, 1369, 1152, 698 cm^{-1}

(e) Metal-free cross-coupling reaction:

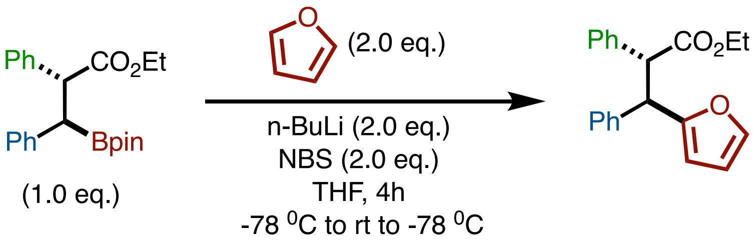

***ethyl 3-(furan-2-yl)-2,3-diphenylpropanoate* (44)**

The title compound was synthesized in accordance with the following modified literature procedure.[21] In a flame-dried round bottom flask equipped with a stir bar under N_2 atmosphere was charged with furan (29 µL, 0.40 mmol, 2.0 eq.) and 1.0 mL of THF was added sequentially via syringe. The mixture was cooled to -78 ^{0}C in a dry ice/acetone bath, and then *n*-BuLi (0.16 mL, 2.5 M in hexanes, 0.4 mmol, 2.0 eq.) was added dropwise via syringe. The mixture was warmed to room temperature and stirred for 1 h, before being cooled to -78 ^{0}C. A solution of ethyl 2,3-diphenyl-3-(4,4,5,5-tetramethyl-1,3,2-dioxaborolan-2-yl) propanoate (76 mg, 0.2 mmol, 1.0 eq.) in THF (1.0 mL) was then added dropwise via syringe to the solution of lithiated furan at -78 ^{0}C, and stirred for 1 hour at this temperature. The reaction mixture was then added dropwise via syringe to a solution of NBS (71 mg, 0.4 mmol, 2.0 eq.) in 1.0 mL THF was added at -78 ^{0}C and stirred for 1 hour at this temperature. After the reaction was quenched with 20% aq. NaS_2O_2 (3 mL) and warmed to room temperature. The layers were separated, and the aqueous layer was extracted with ethyl acetate (3 x 3 mL), the combined organic layers were dried over anhhydrous Sodium sulfate and concentrated in vacuo. The residue was purified via silica gel column chromatography (R_f= 0.4 in 10% EtOAc:Hexane) 7%-10% EtOAc in hexanes to afford the desired product with 46% yield and >20:1 dr.

^{1}H NMR (500 MHz, $CDCl_3$) δ 7.33 (d, *J* = 1.8 Hz, 1H), 7.24 – 7.18 (m, 2H), 7.18 – 7.11 (m, 3H), 7.11 – 7.06 (m, 2H), 7.03 (td, *J* = 7.3, 1.8 Hz, 3H), 6.30 (dd, *J* = 3.2, 1.9 Hz, 1H), 6.24 (d, *J* = 3.2 Hz, 1H), 4.73 (s, 1H), 4.25 (d, *J* = 12.0 Hz, 1H), 4.13 (dq, *J* = 10.9, 7.1 Hz, 1H), 4.02 (dq, *J* = 10.8, 7.1 Hz, 1H), 1.14 (t, *J* = 7.1 Hz, 3H).

^{13}C NMR (126 MHz, $CDCl_3$) δ 172.8, 155.9, 141.7, 139.3, 136.4, 128.8, 128.6, 128.4, 128.2, 127.5, 126.7, 110.3, 106.0, 61.0, 56.5, 48.9, 14.2

HRMS (APCI+): Calculated for $C_{21}H_{20}O_3Na$ $[M+Na]^+$: 343.1305, Found: 343.1305

IR (Neat): 2928, 1731, 1454, 1356, 1154, cm^{-1}

(f) Amidation reaction:

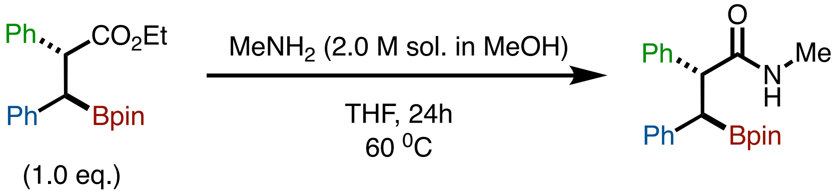

N-methyl-2,3-diphenyl-3-(4,4,5,5-tetramethyl-1,3,2-dioxaborolan-2-yl) propenamide **(47)**

In a flame dried reaction vial under N_2 atmosphere a solution of ethyl 2,3-diphenyl-3-(4,4,5,5-tetramethyl-1,3,2-dioxaborolan-2-yl) propanoate (114 mg, 0.3 mmol, 1.0 eq.) in THF (1.5 mL) was added $MeNH_2$ (2.0 M in MeOH, 200 µL, 0.6 mmol, 2.0 eq.) at 60 ˚C. After being stirred at 60 ^{0}C for 24 hours, the solvent was evaporated under reduced pressure. The residue was purified via silica gel column chromatography (R_f= 0.4 in 5% methanol/DCM) 3% MeOH in DCM to afford the desired product with 52% yield and 5:1 rotamer.

^{1}H NMR (500 MHz, $CDCl_3$) ***major rotamer*** δ 7.25 – 7.10 (m, 4H), 7.06 (t, *J* = 4.3 Hz, 6H), 5.32 (s, 1H), 4.05 (d, *J* = 12.4 Hz, 1H), 2.87 (d, *J* = 12.5 Hz, 1H), 2.77 (d, *J* = 4.9 Hz, 3H), 1.18 (s, 6H), 1.16 (s, 6H); ***minor rotamer*** δ 7.50 – 7.46 (m, 2H), 7.38 (d, *J* = 1.4 Hz, 2H), 7.29 (dd, *J* = 8.4, 6.9 Hz, 2H), 6.98 (h, *J* = 4.2 Hz, 4H), 5.15 (dd, *J* = 8.9, 4.9 Hz, 1H), 3.70 (d, *J* = 12.0 Hz, 1H), 3.32 (d, *J* = 12.0 Hz, 1H), 2.49 (dd, *J* = 4.9, 1.4 Hz, 3H), 0.91 (s, 6H), 0.89 (s, 6H).

^{13}C NMR (126 MHz, $CDCl_3$) ***major rotamer*** δ 176.3, 140.4, 139.2, 128.9, 128.8, 128.7, 127.9, 127.3, 125.1, 82.7, 56.5, 27.1, 24.8, 24.7; ***minor rotamer*** δ 173.2, 140.5, 139.8, 129.1, 128.8, 128.7, 128.5, 128.4, 126.0, 125.8, 83.4, 57.0, 26.4, 24.5, 24.2

^{11}B NMR (160 MHz, $CDCl_3$) δ 29.22

HRMS (APCI+): Calculated for $C_{22}H_{28}O_3BNNa$ $[M+Na]^+$: 388.2054, Found: 388.2053

IR (Neat): 2925, 1699, 1647, 1362, 1141 cm^{-1}

10. Determination of relative configuration:

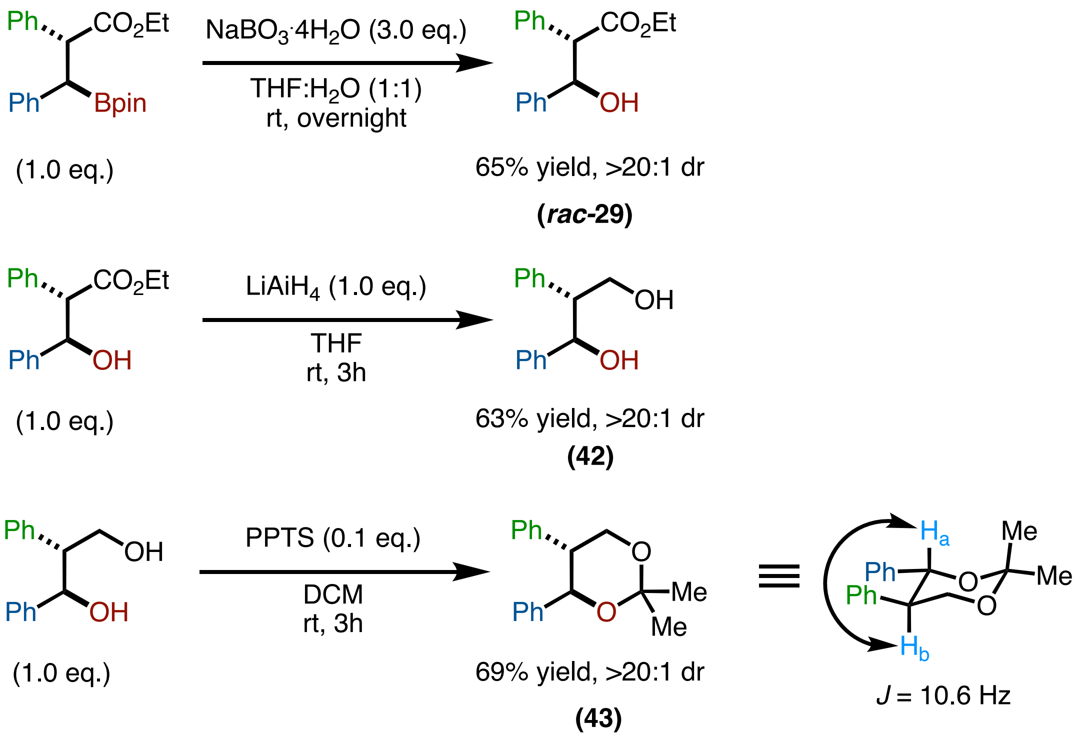

All data were matched with literature data reported.[25]

The arylboration product ethyl-2,3-diphenyl-3-(4,4,5,5-tetramethyl-1,3,2-dioxaborolan-2-yl) propanoate (0.2 mmol) was taken in a 10 mL round-bottom flask, dissolved in THF (1.0 mL) and $NaBO_3 \cdot 4H_2O$ (90 mg, 0.58 mmol, 3.0 eq.) was added, followed by addition of water (1.0 mL). The reaction was allowed to stir at room temperature overnight. Then the reaction mixture was quenched upon the addition of aqueous sat. $Na_2S_2O_3$ (3.0 mL) and extracted with EtOAc (3 x 2 mL). The combined organic layers were washed with 2 M HCl (3 x 3 mL), dried over sodium sulfate, and concentrated in vacuo. The residue was purified via silica gel column chromatography (R_f= 0.35 in 20% EtOAc:Hexane) 16%-20% EtOAc in hexanes to afford the desired product **(rac-29)** with 65% yield and >20:1 dr.

^{1}H NMR (500 MHz, $CDCl_3$) δ 7.18 (dt, J = 6.1, 2.7 Hz, 6H), 7.10 (td, J = 6.0, 3.1 Hz, 4H), 5.17 (dd, J = 9.2, 3.5 Hz, 1H), 4.24 (dq, J = 10.6, 7.1 Hz, 1H), 4.16 (dq, J = 10.6, 7.1 Hz, 1H), 3.87 (d, J = 9.1 Hz, 1H), 3.22 (d, J = 4.1 Hz, 1H), 1.22 (t, J = 7.1 Hz, 3H).

^{13}C NMR (126 MHz, $CDCl_3$) δ 173.6, 140.9, 135.5, 128.7, 128.6, 128.2, 127.9, 127.6, 126.7, 76.8, 61.3, 60.1, 14.3

Then the compound ethyl 3-hydroxy-2,3-diphenylpropanoate (34 mg, 0.13 mmol, 1.0 eq.) was taken in a 5.0 mL round bottom flask under N_2 atmosphere containing $LiAlH_4$ (4.8 mg, 0.13 mmol, 1.0 eq.) in 2.0 mL of THF and stirred for 3h. After stirring for 3 h, H_2O (2 mL/mmol of substrate)

and 1 N HCl (2 mL/mmol of substrate) solution at 0 ^{0}C were added and the solutions were extracted with DCM (3 x 3 mL). The extracts were dried over anhydrous Na_2SO_4 and concentrated in vacuo. The residue was purified via silica gel column chromatography (R_f= 0.3 in 40% EtOAc:Hexane) 45%-50% EtOAc in hexanes to afford the desired product **(42)** with 63% yield and >20:1 dr.

^{1}H NMR (500 MHz, $CDCl_3$) δ 7.24 – 7.11 (m, 8H), 7.06 – 6.98 (m, 2H), 5.04 (dd, *J* = 8.4, 4.5 Hz, 1H), 4.21 (ddd, *J* = 11.4, 7.9, 4.6 Hz, 1H), 3.99 (dt, *J* = 11.1, 4.6 Hz, 1H), 3.17 (tt, *J* = 8.3, 4.3 Hz, 1H), 3.04 – 2.68 (brs, 2H).

^{13}C NMR (126 MHz, $CDCl_3$) δ 142.8, 139.3, 128.6, 128.6, 128.3, 127.8, 127.0, 126.7, 79.8, 66.54, 55.0

In a flame dried 10 ml round bottom flask under N_2 atmosphere a solution of 1,2-diphenylpropane-1,3-diol (18 mg, 79 µmol, 1.0 eq.) in DCM (2 mL) were added 2,2- dimethoxypropane (48 µL, 0.39 mmol, 5.0 eq.) and PPTS (2.0 mg, 7.9 µmol, 0.1 eq.) at 0 °C, and the mixture was stirred at room temperature for 3 h. After completion the reaction mixture was added saturated NH_4Cl solution at 0 °C, and the aqueous layer was extracted with EtOAc (3 x 3 ml). The organic layer was washed with brine (5.0 mL). The combined organic layer was dried over anhydrous Na_2SO_4 and concentrated in vacuo. The residue was purified via silica gel column chromatography (R_f= 0.5 in 10% EtOAc:Hexane) 3%-4% EtOAc in hexanes to afford the desired product **()** with 69% yield and >20:1 dr.

^{1}H NMR (500 MHz, $CDCl_3$) δ 7.23 – 7.09 (m, 8H), 7.06 – 6.99 (m, 2H), 5.02 (d, *J* = 10.5 Hz, 1H), 4.23 (t, *J* = 11.7 Hz, 1H), 3.98 (dd, *J* = 11.8, 5.2 Hz, 1H), 3.06 (td, *J* = 11.0, 5.1 Hz, 1H), 1.72 (s, 3H), 1.58 (s, 3H).

^{13}C NMR (126 MHz, $CDCl_3$) δ 140.1, 138.1, 128.6, 128.6, 128.1, 127.8, 127.2, 127.2, 99.0, 65.48, 49.3, 30.0, 19.5

The coupling constant (J = 10.6 Hz) between Ha and Hb on ^{1}HNMR spectra of **(43)** indicated that the relative stereochemistry of the substituents on the 1,3-dioxane ring of **(43)** was anti-orientation

Establishments of Absolute configuration:

The absolute configuration of the C-B bond forming in the products **(29, 31-41)** for the enantioselective reaction using chiral Cu-McQuade catalyst is believed to has the *(R)-Configuration.* The absolute configuration determines based on the previous literature report by McQuade and co-workers[3]. In this report the opposite enantiomer of the catalyst was used to obtain the hydroborated product from same (E)-ethyl cinnamate. The product was established to have *(S)-Configuration* by comparison of the optical rotation value with literature report[26]

McQuade and co-workers:

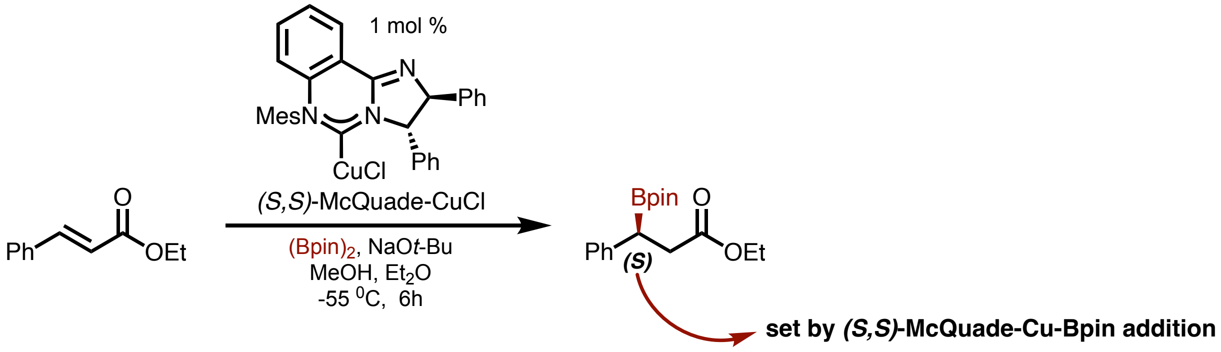

This Study:

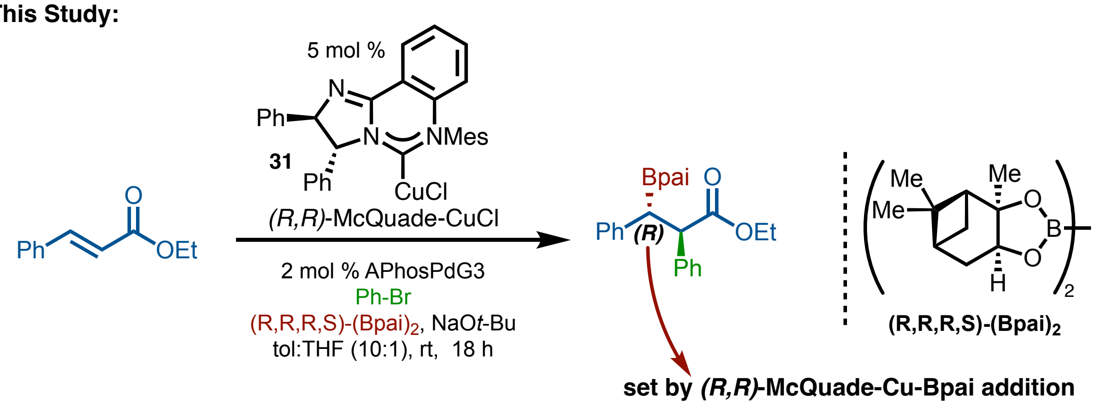

11.NMR Spectra

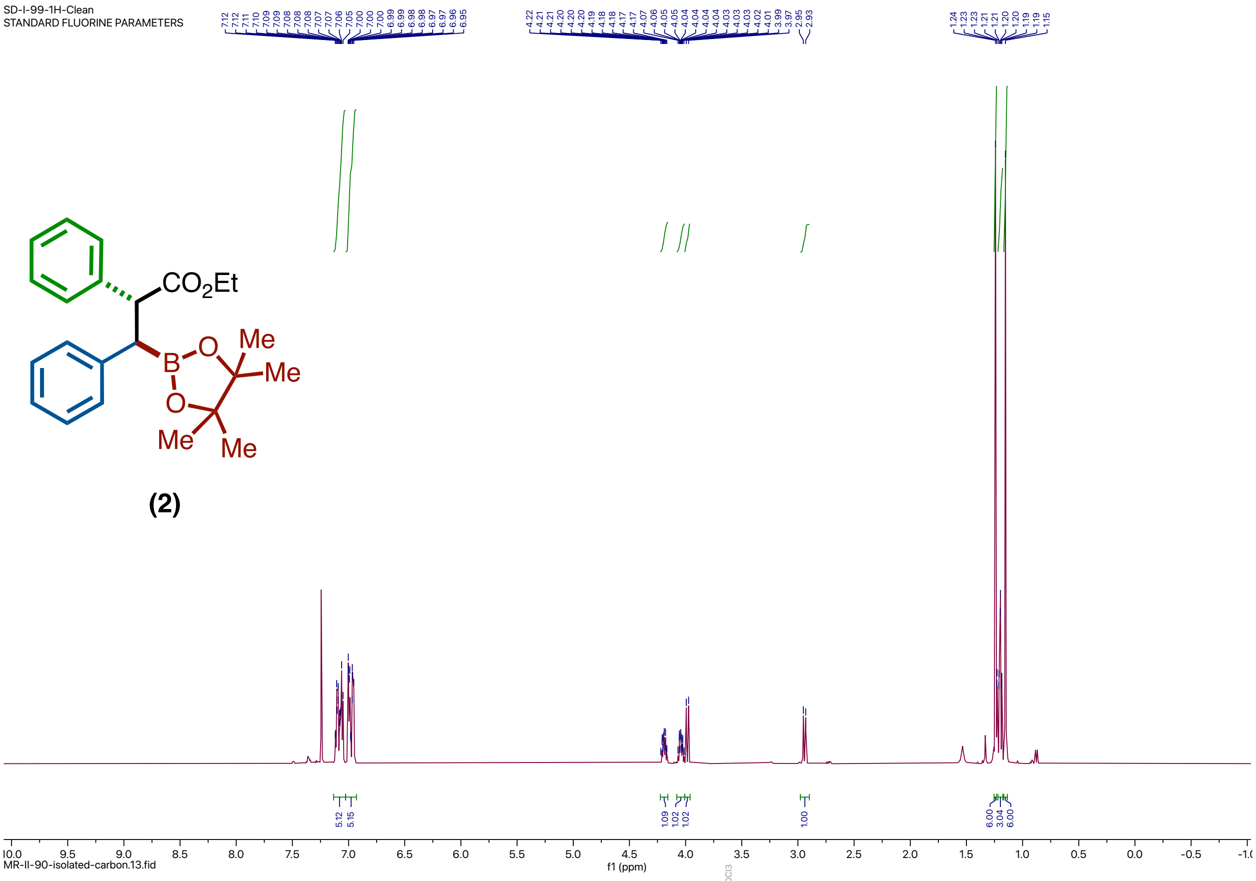
SD-I-99-1H-Clean
STANDARD FLUORINE PARAMETERS
CO2Et
B
O
O
Me
Me
Me
Me
f1 (ppm)

(2)

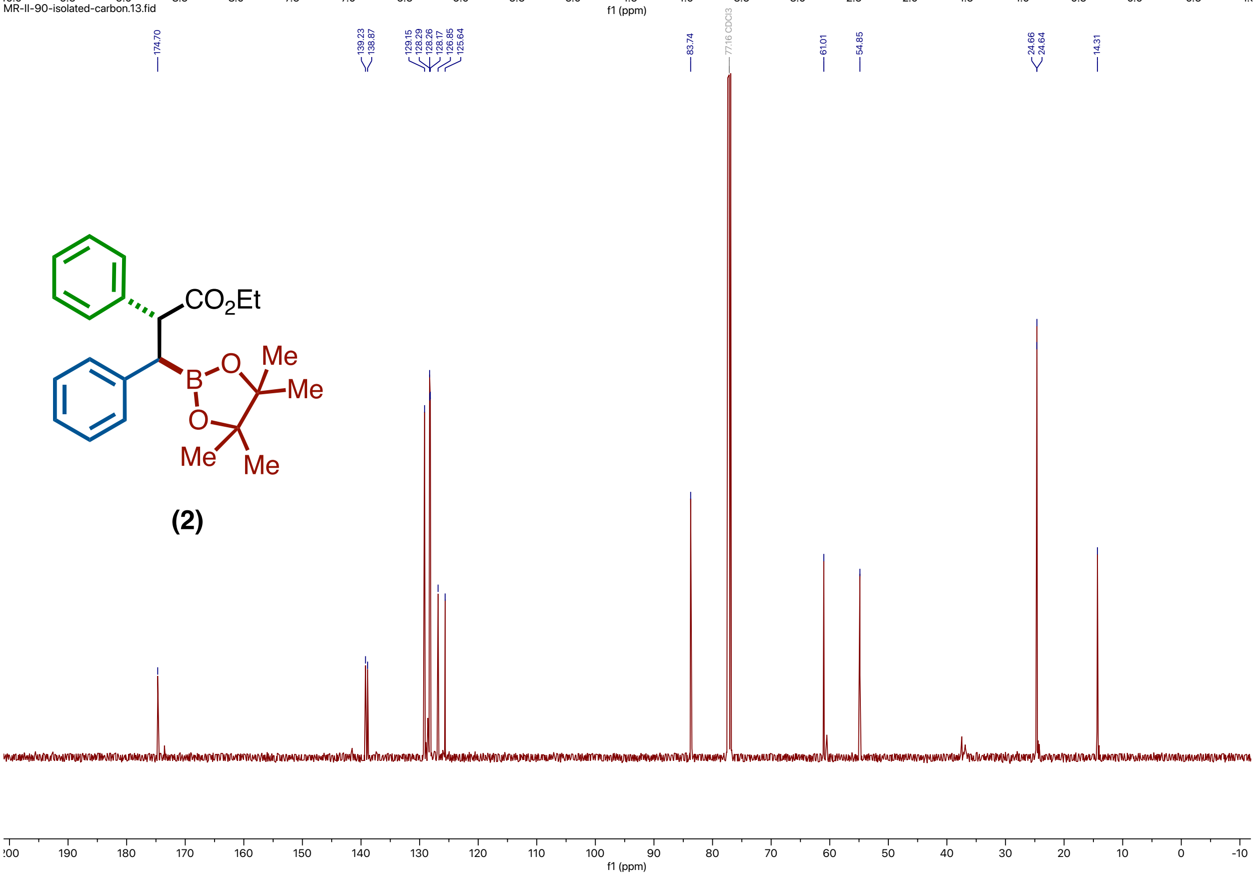
MR-II-90-isolated-carbon.13.fid
174.70
139.23
138.87
129.15
128.29
128.26
128.17
126.85
125.64
83.74
77.16 CDCl3
61.01
54.85
24.66
24.64
14.31
CO2Et
B
O
O
Me
Me
Me
Me
f1 (ppm)

(2)

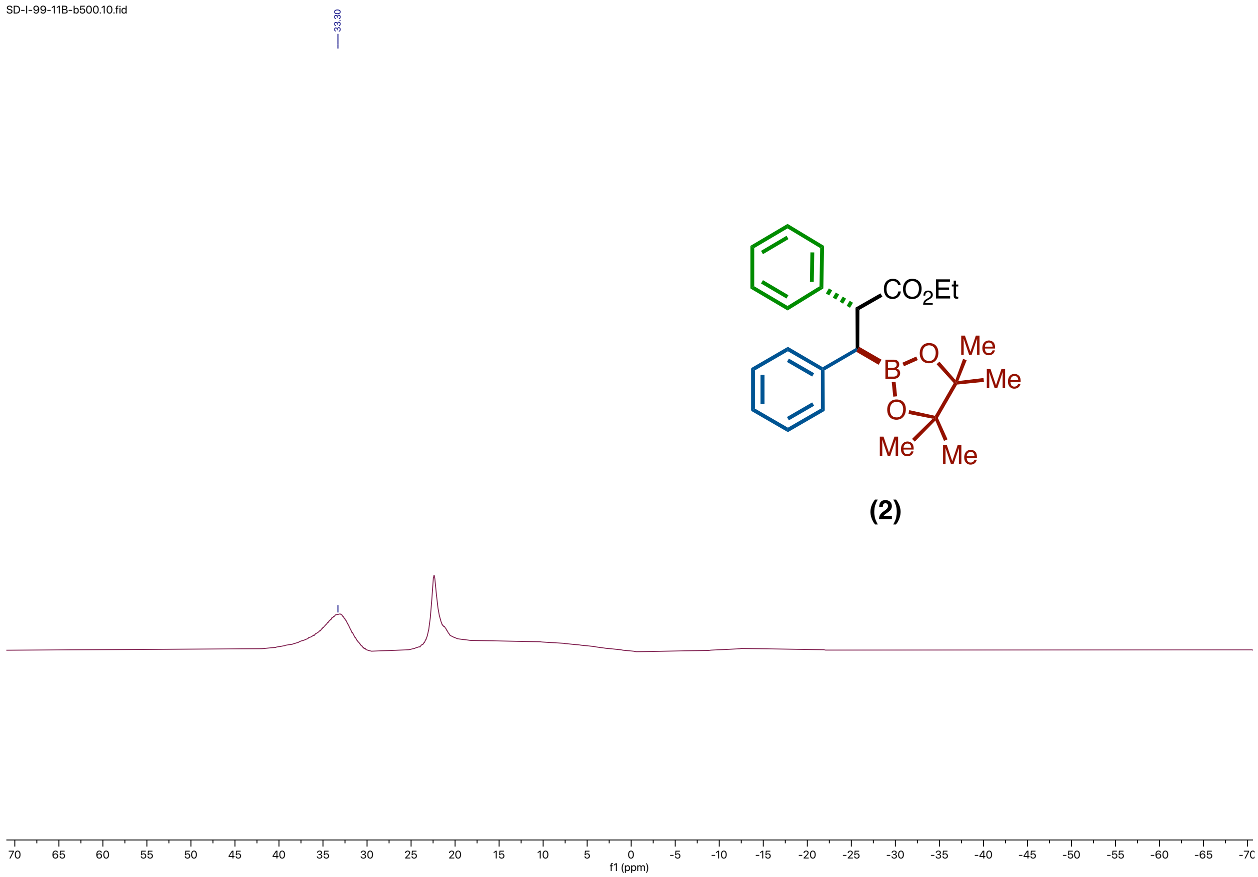
SD-I-99-11B-b500.10.fid
33.30
CO2Et
B
O
O
Me
Me
Me
Me
(2)
70 65 60 55 50 45 40 35 30 25 20 15 10 5 0 -5 -10 -15 -20 -25 -30 -35 -40 -45 -50 -55 -60 -65 -70
f1 (ppm)

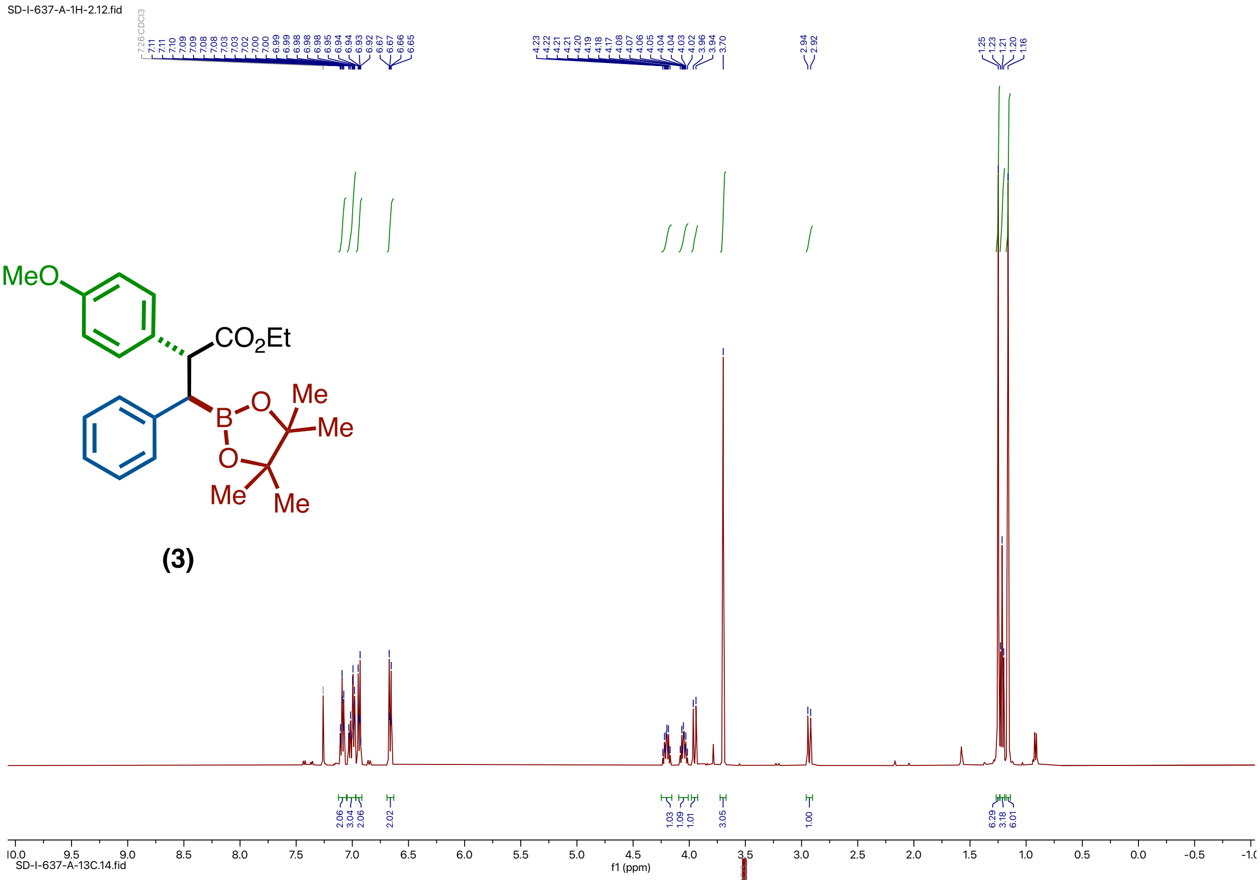
SD-I-637-A-1H-2.12.fid
7.26 CDCl3
7.11 7.10 7.09 7.09 7.08 7.08 7.03 7.03 7.02 7.00 7.00 6.99 6.99 6.98 6.98 6.98 6.95 6.94 6.94 6.93 6.92 6.67 6.67 6.66 6.65
4.23 4.22 4.21 4.21 4.20 4.19 4.18 4.17 4.08 4.07 4.06 4.05 4.04 4.04 4.03 4.02 3.96 3.94 3.70
2.94 2.92
1.25 1.23 1.21 1.20 1.16
MeO
CO2Et
B
O
O
Me
Me
Me
Me
(3)
2.06 3.04 2.06 2.02 1.03 1.09 1.01 3.05 1.00 6.29 3.18 6.01
f1 (ppm)

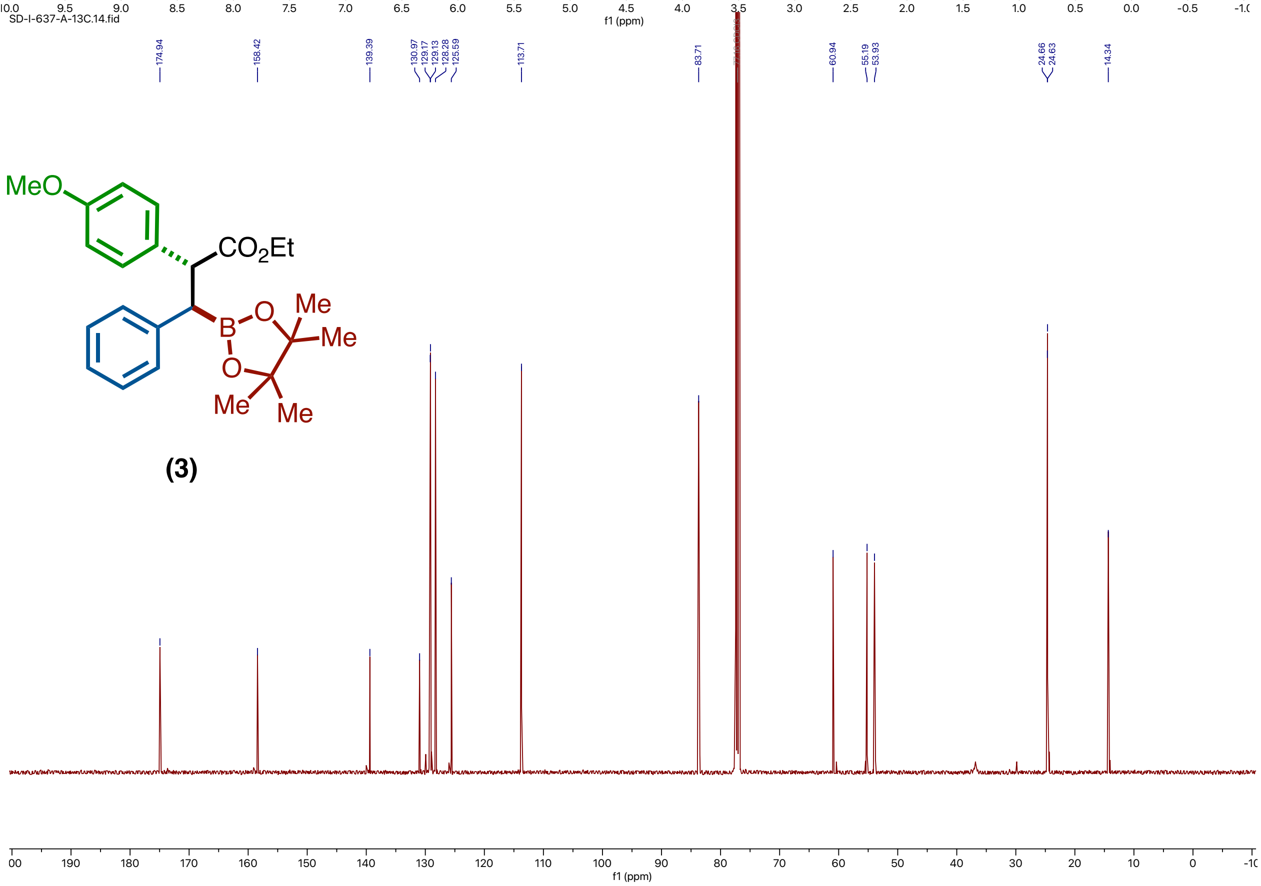
SD-I-637-A-13C.14.fid
174.94
158.42
139.39
130.97 129.17 129.13 128.28 125.59
113.71
83.71
60.94
55.19 53.93
24.66 24.63
14.34
MeO
CO2Et
B
O
O
Me
Me
Me
Me
(3)
f1 (ppm)

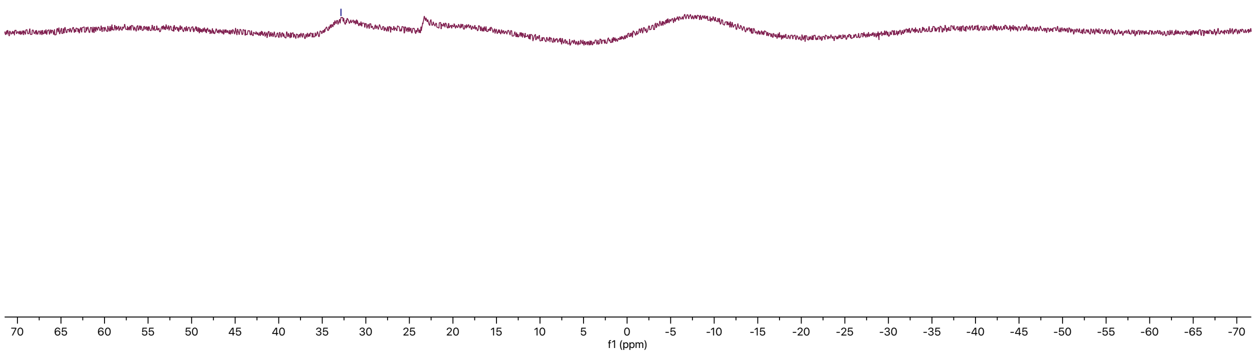
SD-I-637-11B
STANDARD BORON PARAMETERS
32.85
70 65 60 55 50 45 40 35 30 25 20 15 10 5 0 -5 -10 -15 -20 -25 -30 -35 -40 -45 -50 -55 -60 -65 -70
f1 (ppm)

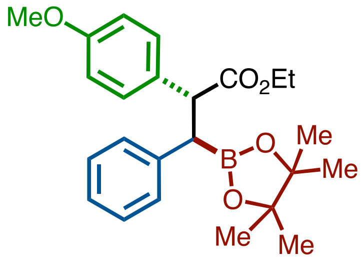
MeO
CO2Et
B
O
O
Me
Me
Me
Me

(3)

SD-I-634-1H-010101.10.fid

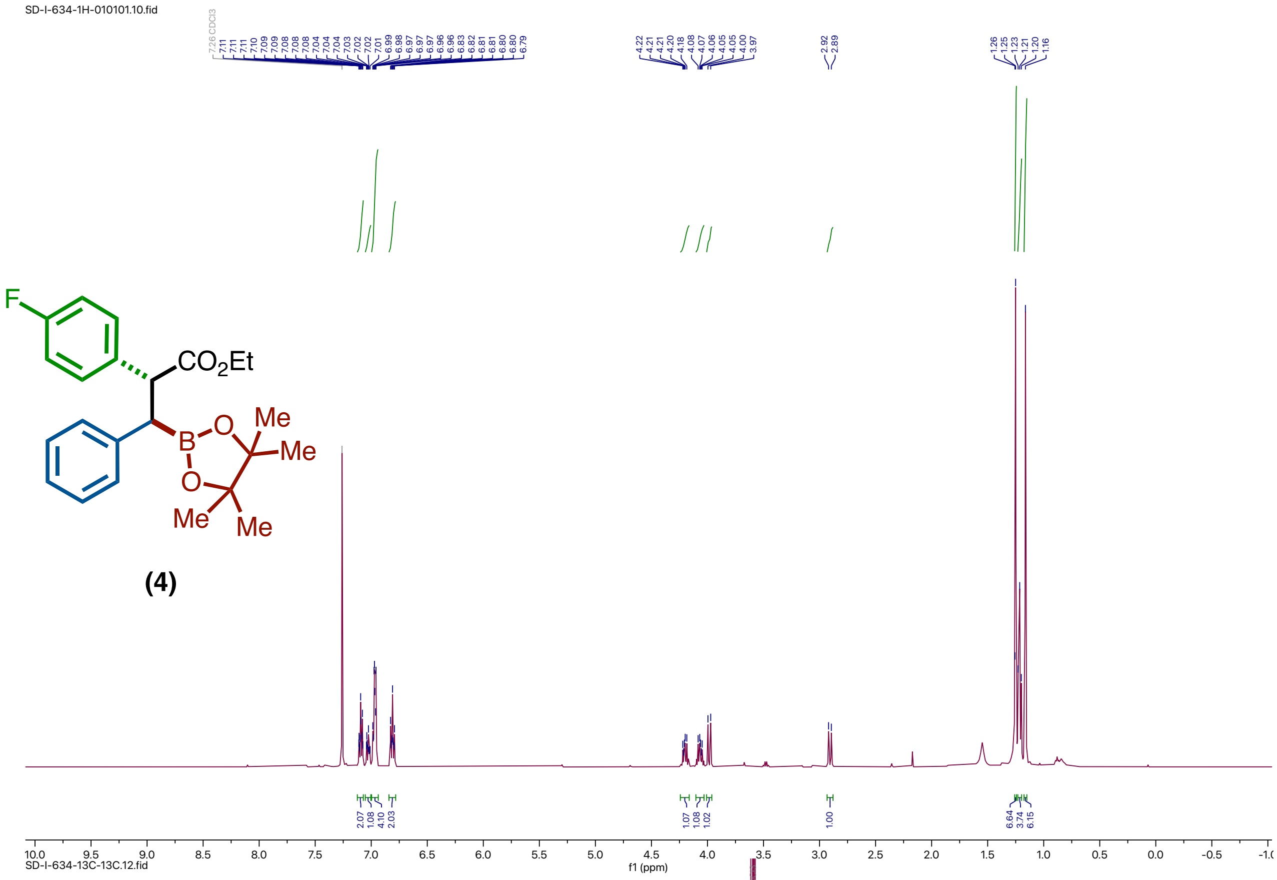

(4)

SD-I-634-13C-13C.12.fid

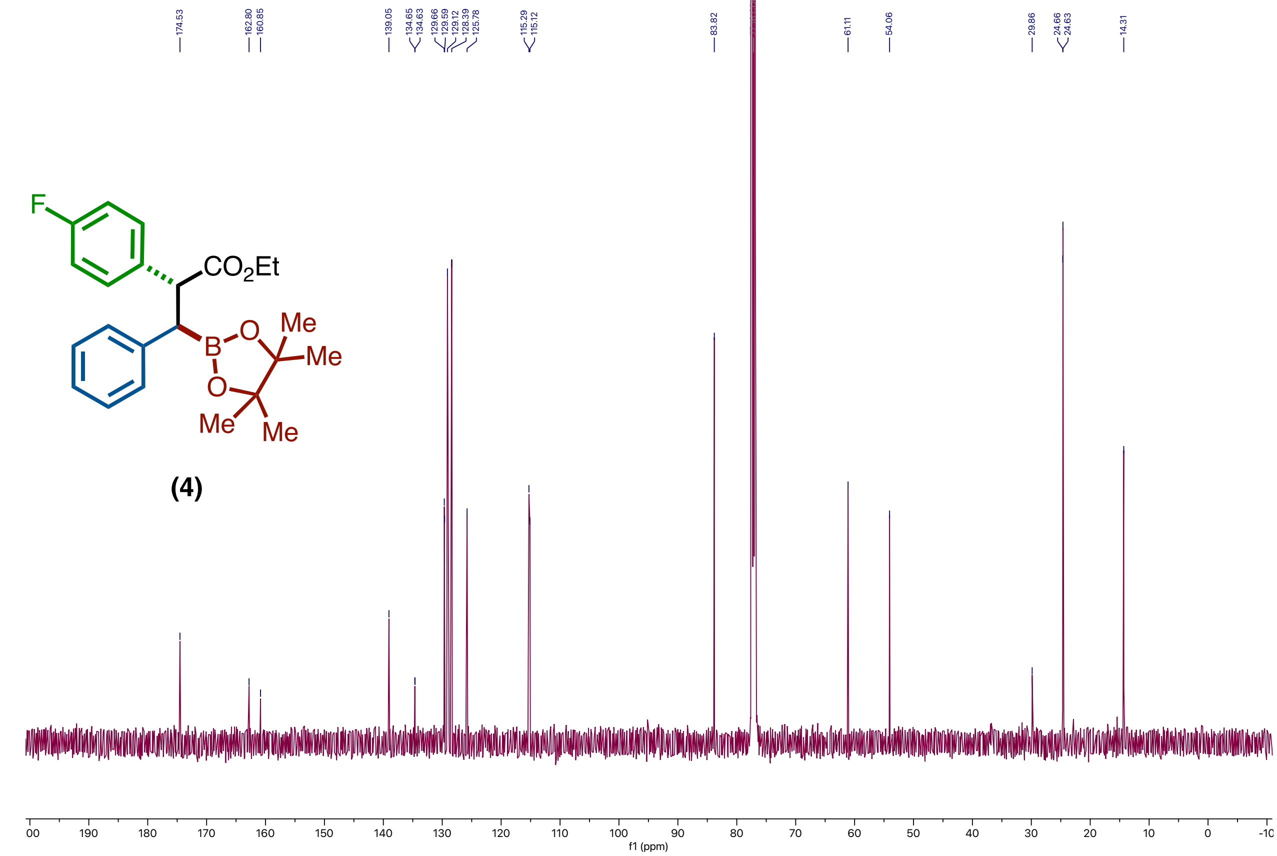

(4)

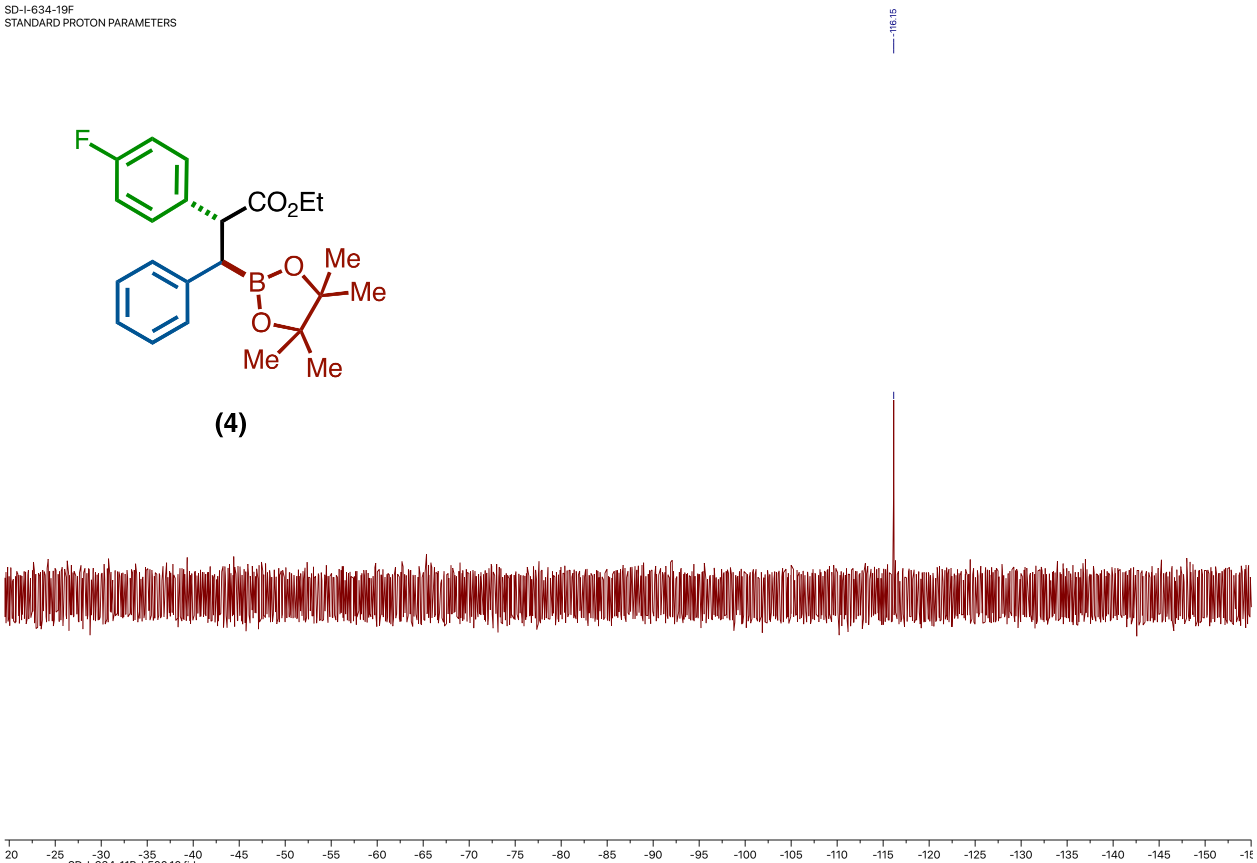
SD-I-634-19F
STANDARD PROTON PARAMETERS
-116.15
F
CO_2Et
B
O
O
Me
Me
Me
Me
(4)
f1 (ppm)

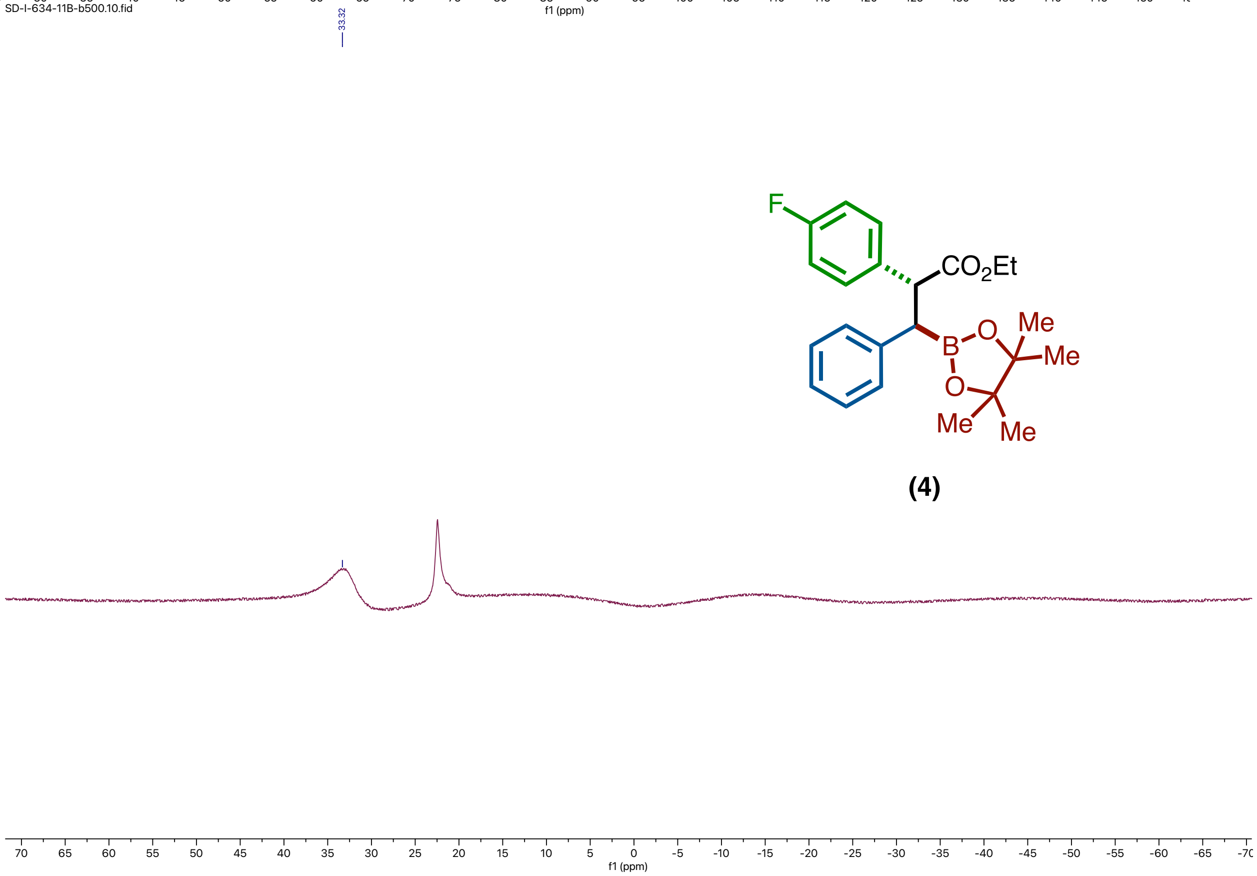
SD-I-634-11B-b500.10.fid
33.32
F
CO_2Et
B
O
O
Me
Me
Me
Me
(4)
f1 (ppm)

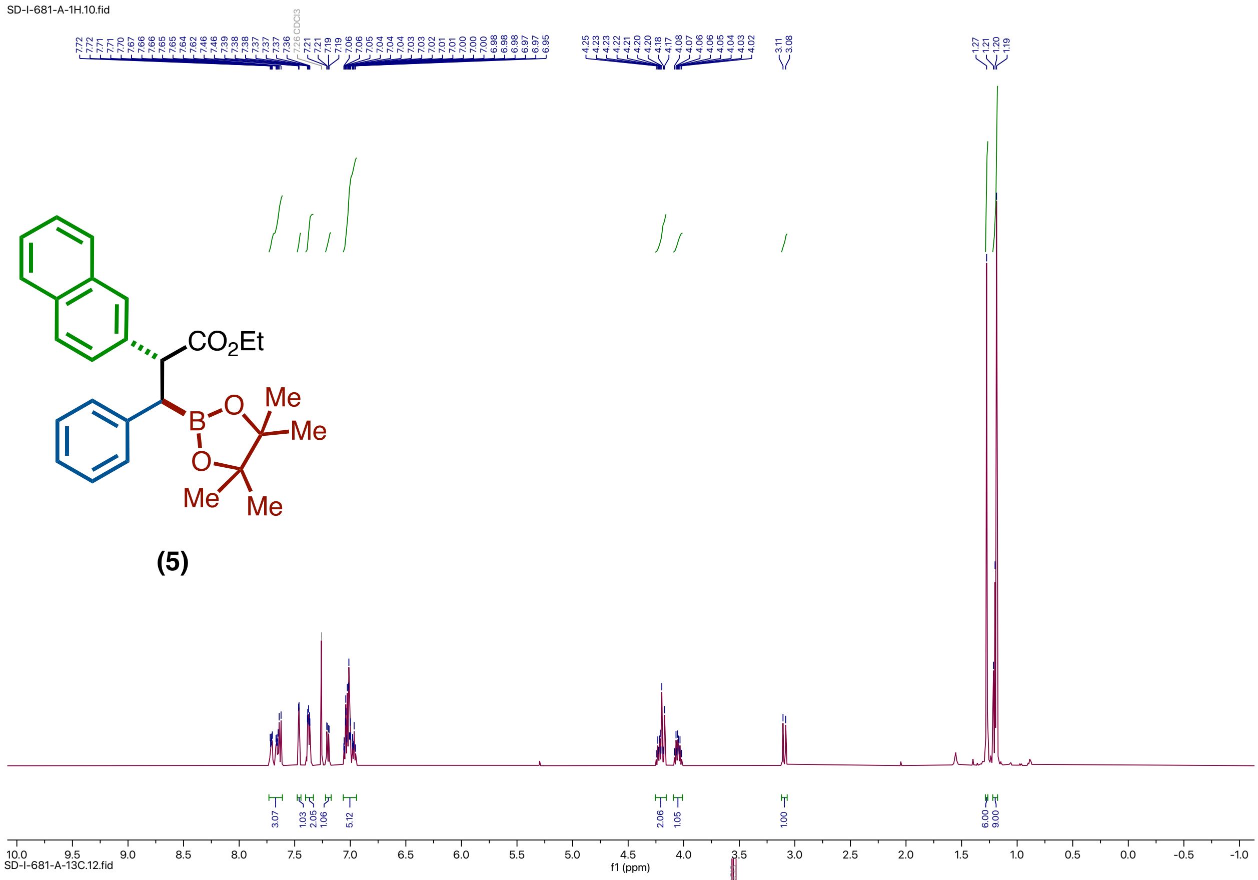

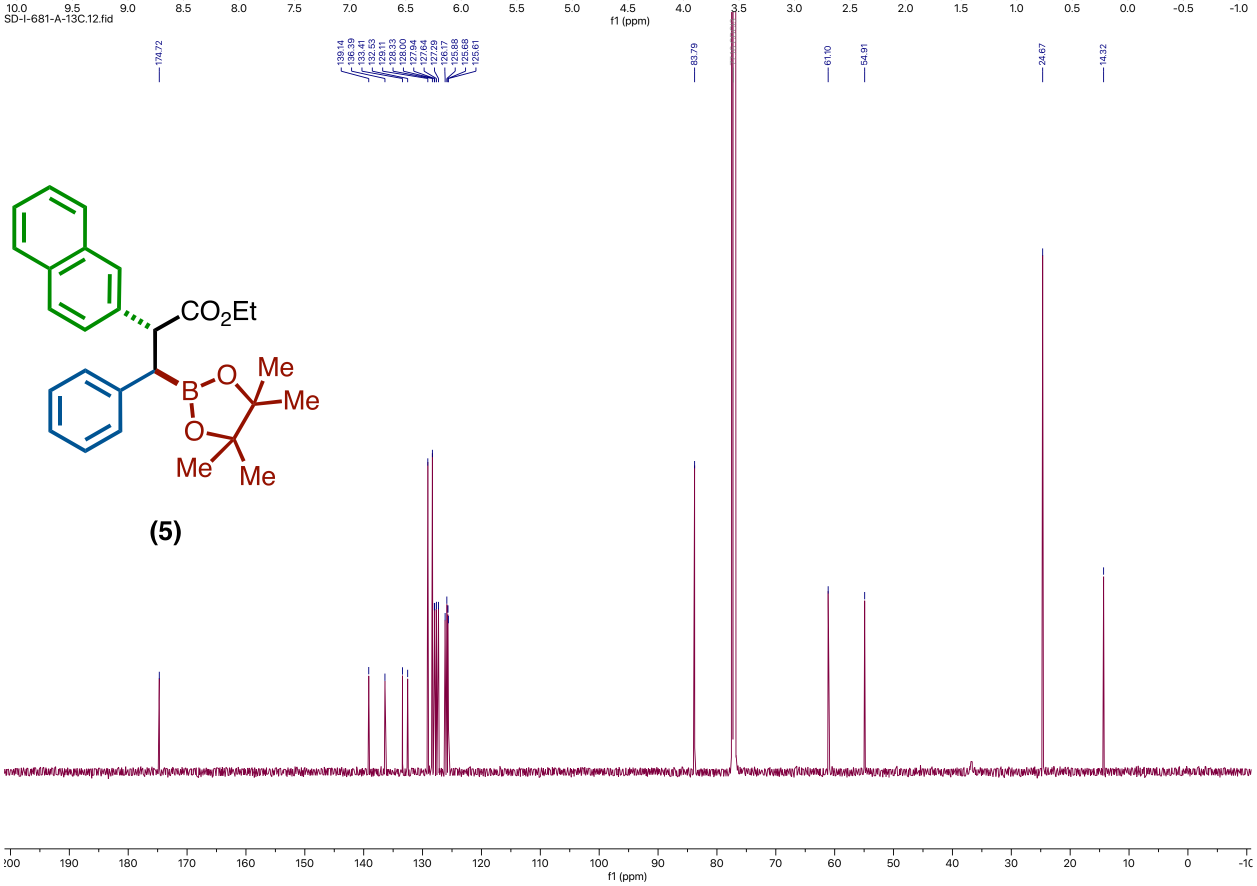

SD-I-681-A-11B-b500.10.fid

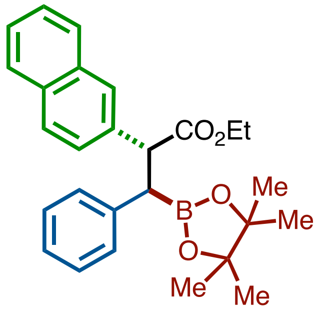

(5)

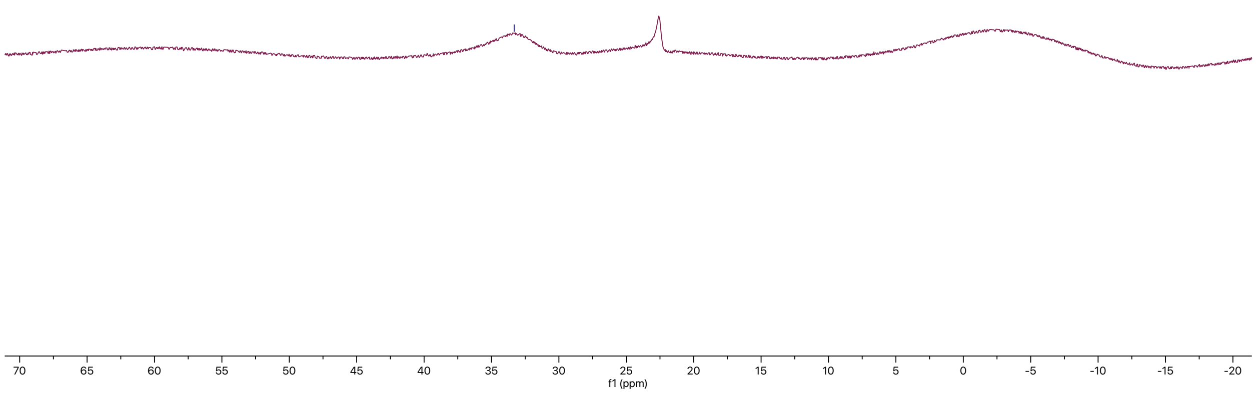

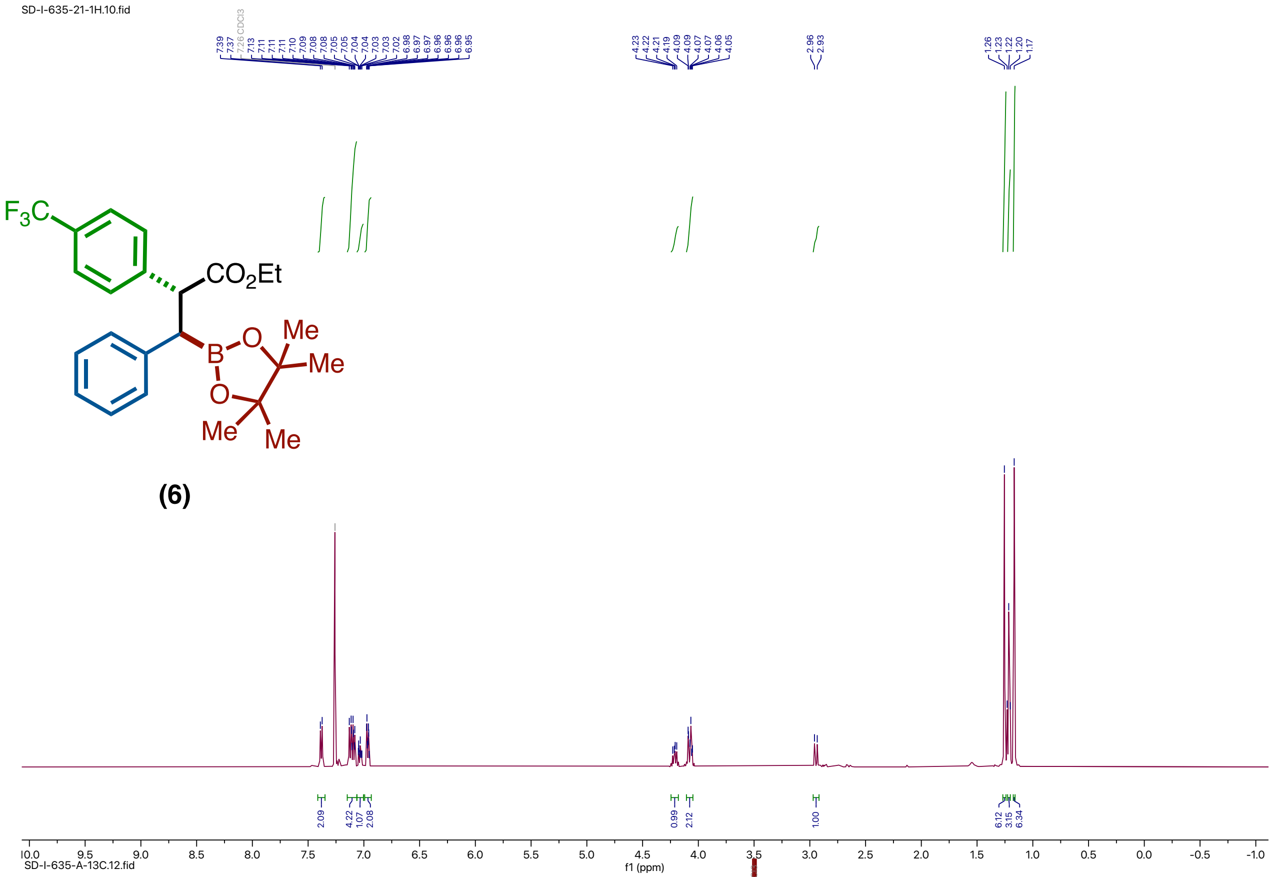
SD-I-635-21-1H.10.fid
7.39
7.37
7.26 CDCl3
7.13
7.11
7.11
7.11
7.10
7.09
7.08
7.08
7.05
7.05
7.04
7.04
7.03
7.03
7.02
6.98
6.97
6.97
6.96
6.96
6.96
6.95
4.23
4.22
4.21
4.19
4.09
4.09
4.07
4.07
4.06
4.05
2.96
2.93
1.26
1.23
1.22
1.20
1.17
F_3C
CO_2Et
B
O
O
Me
Me
Me
Me
(6)
2.09
4.22
1.07
2.08
0.99
2.12
1.00
6.12
3.15
6.34
f1 (ppm)

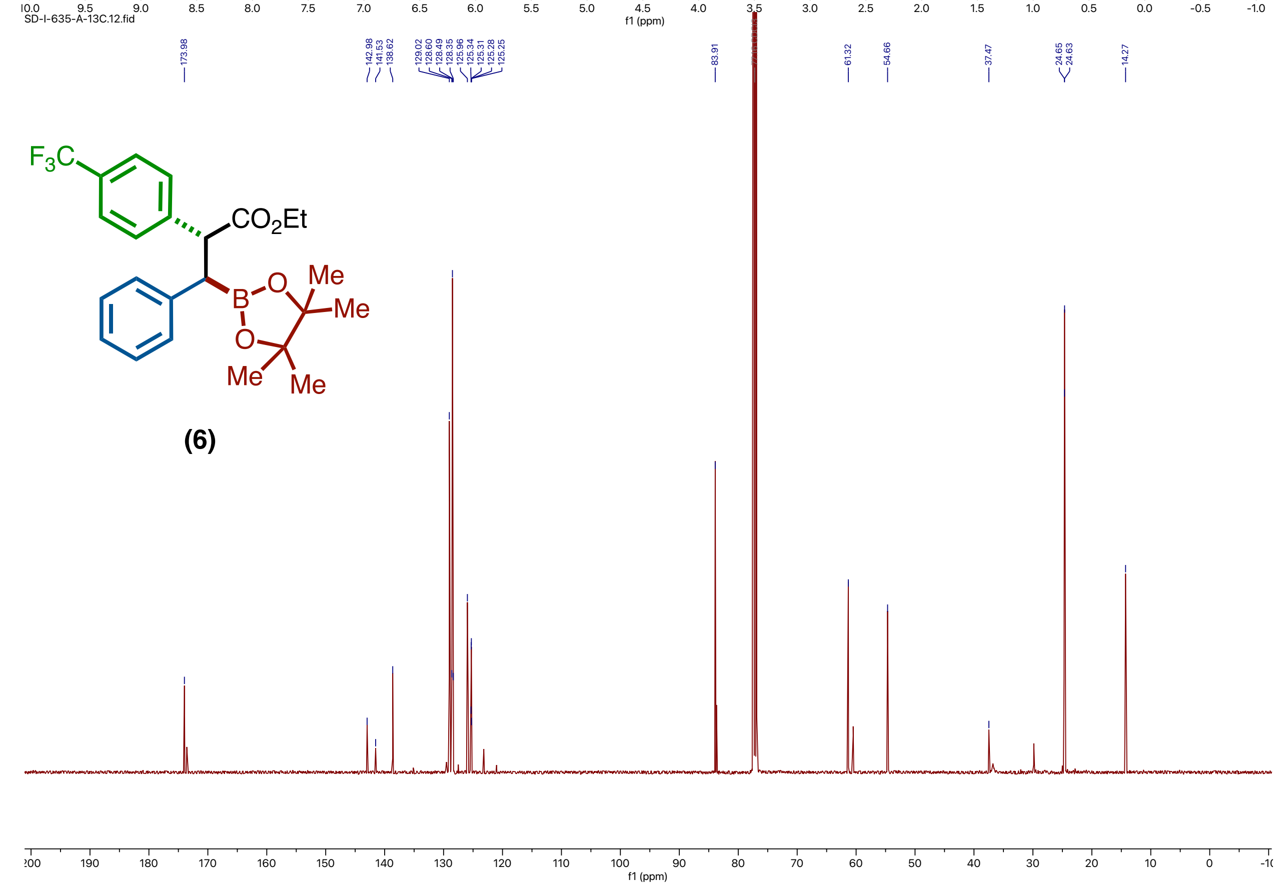
SD-I-635-A-13C.12.fid
173.98
142.98
141.53
138.62
129.02
128.60
128.49
128.35
125.96
125.34
125.31
125.28
125.25
83.91
61.32
54.66
37.47
24.65
24.63
14.27
F_3C
CO_2Et
B
O
O
Me
Me
Me
Me
(6)
f1 (ppm)

SD-I-635-19F
STANDARD PROTON PARAMETERS

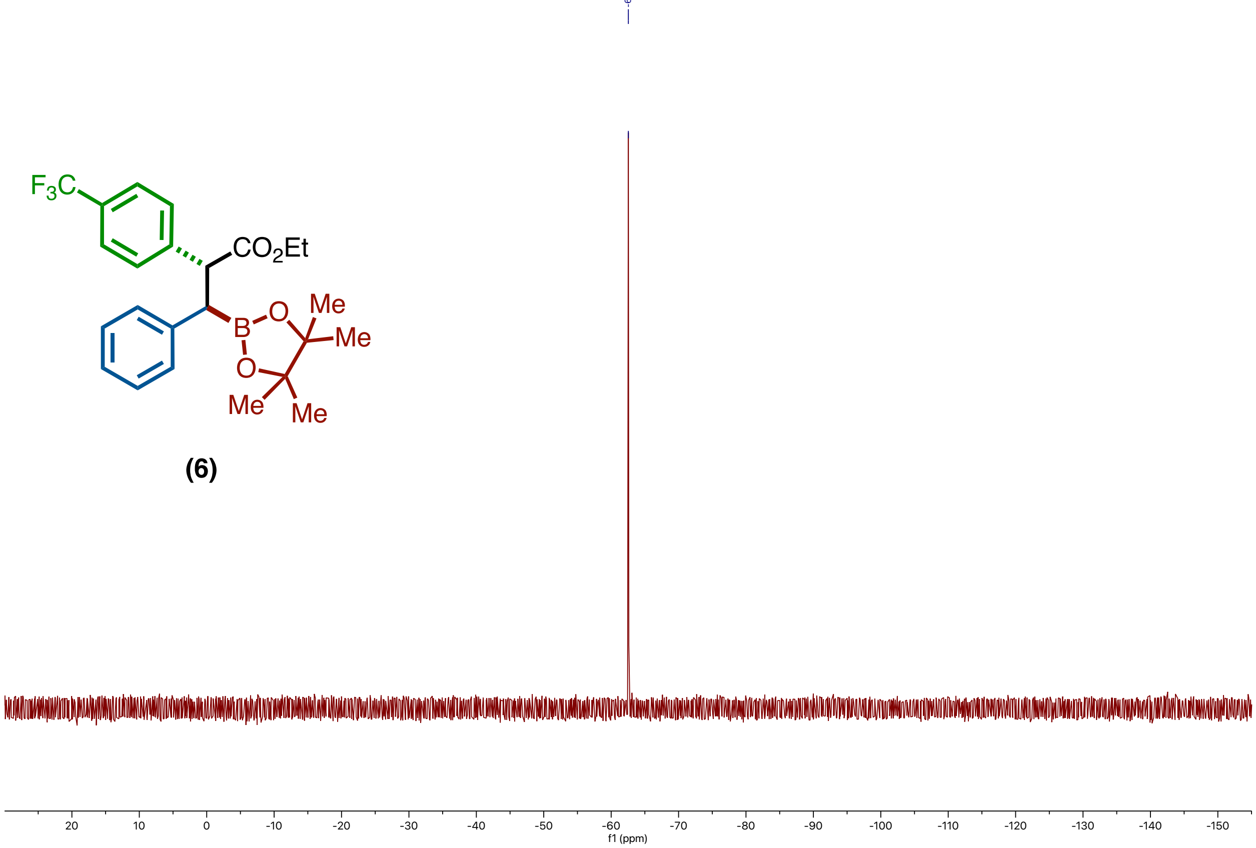

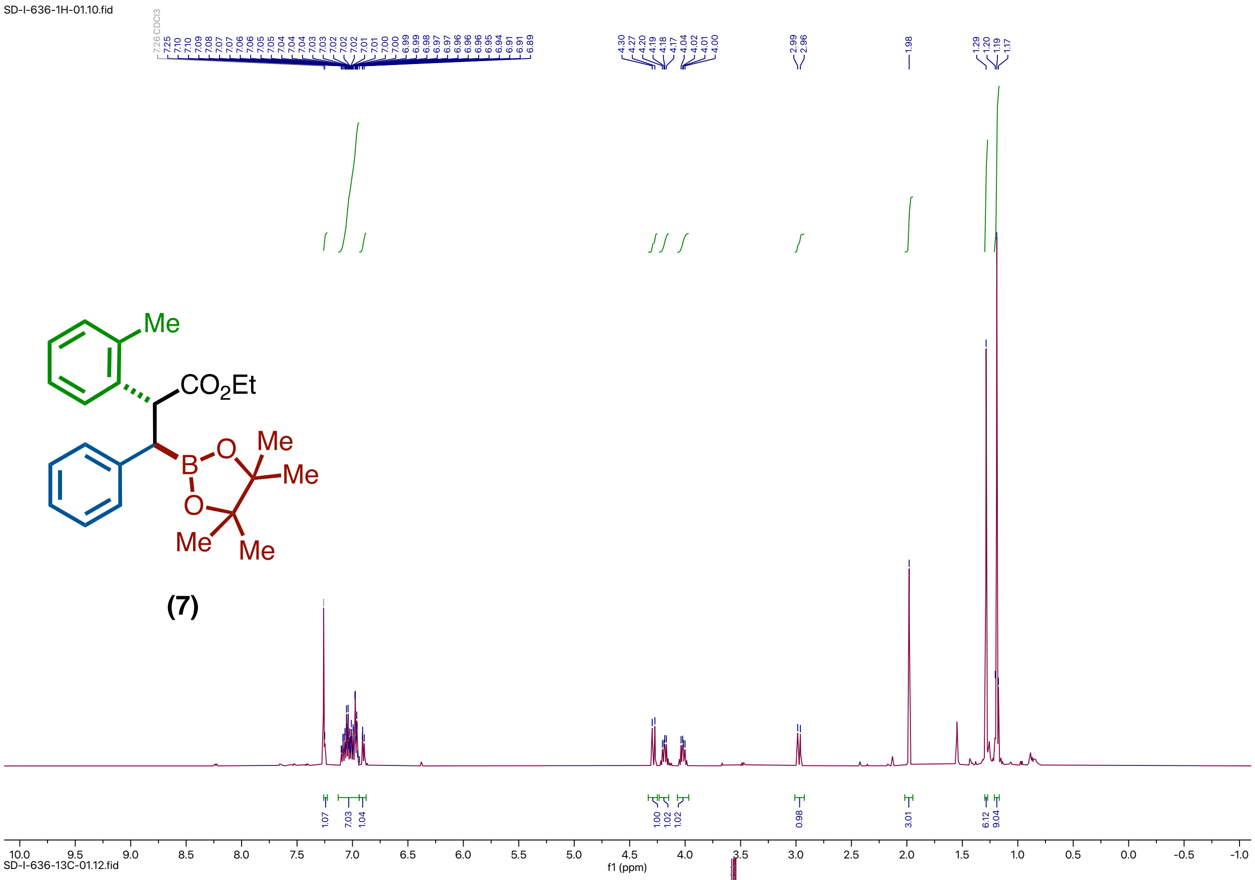
SD-I-636-1H-01.10.fid
7.26 CDCl3
7.25 7.10 7.09 7.08 7.07 7.07 7.06 7.06 7.05 7.05 7.04 7.04 7.04 7.03 7.03 7.02 7.02 7.02 7.01 7.01 7.00 7.00 6.99 6.99 6.98 6.97 6.97 6.96 6.96 6.96 6.95 6.94 6.91 6.91 6.89
4.30 4.27 4.20 4.19 4.18 4.17 4.04 4.02 4.01 4.00
2.99 2.96
1.98
1.29 1.20 1.19 1.17
Me
CO2Et
B
O
O
Me
Me
Me
Me
(7)
1.07 7.03 1.04
1.00 1.02 1.02
0.98
3.01
6.12 9.04
10.0 9.5 9.0 8.5 8.0 7.5 7.0 6.5 6.0 5.5 5.0 4.5 4.0 3.5 3.0 2.5 2.0 1.5 1.0 0.5 0.0 -0.5 -1.0
f1 (ppm)

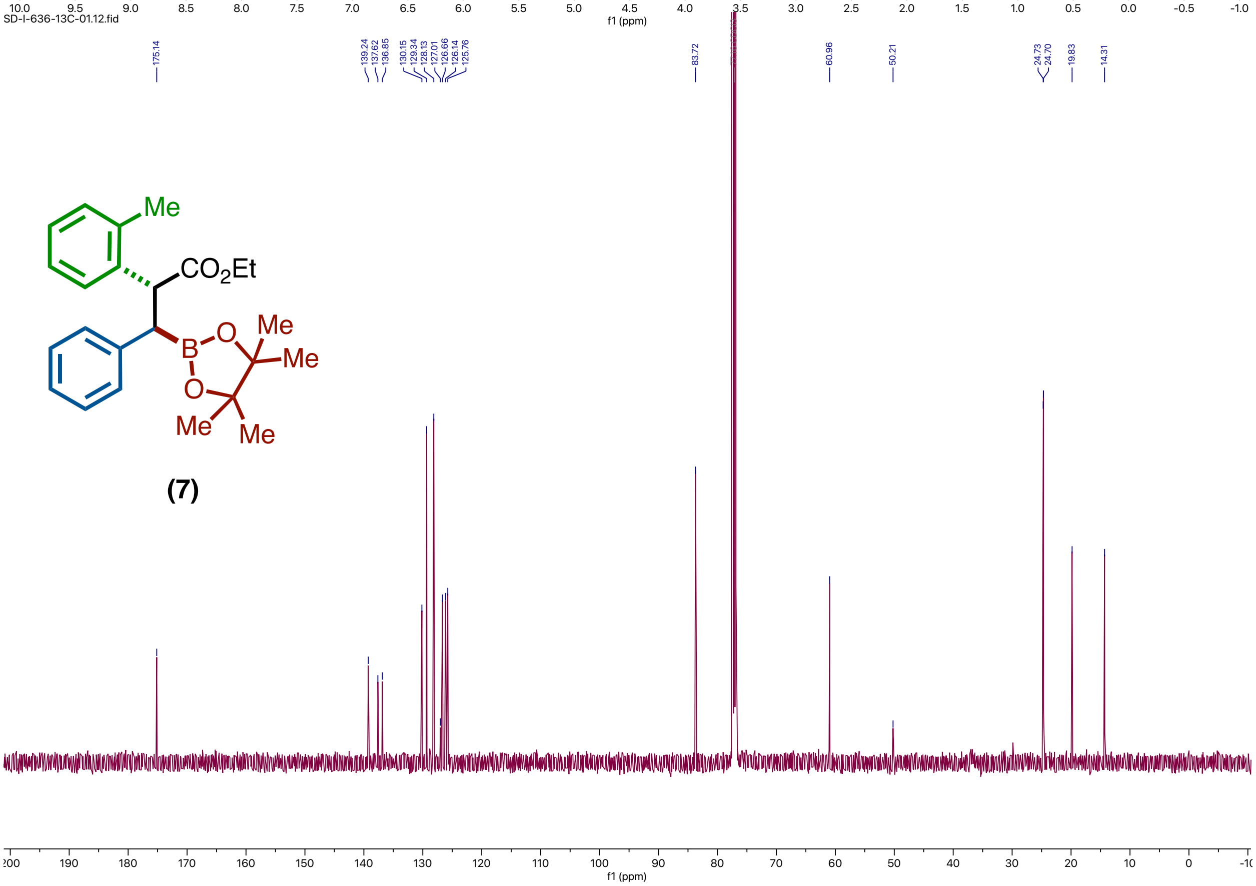
SD-I-636-13C-01.12.fid
175.14
139.24 137.62 136.85 130.15 129.34 128.13 127.01 126.66 126.14 125.76
83.72
60.96
50.21
24.73 24.70
19.83
14.31
Me
CO2Et
B
O
O
Me
Me
Me
Me
(7)
200 190 180 170 160 150 140 130 120 110 100 90 80 70 60 50 40 30 20 10 0 -10
f1 (ppm)

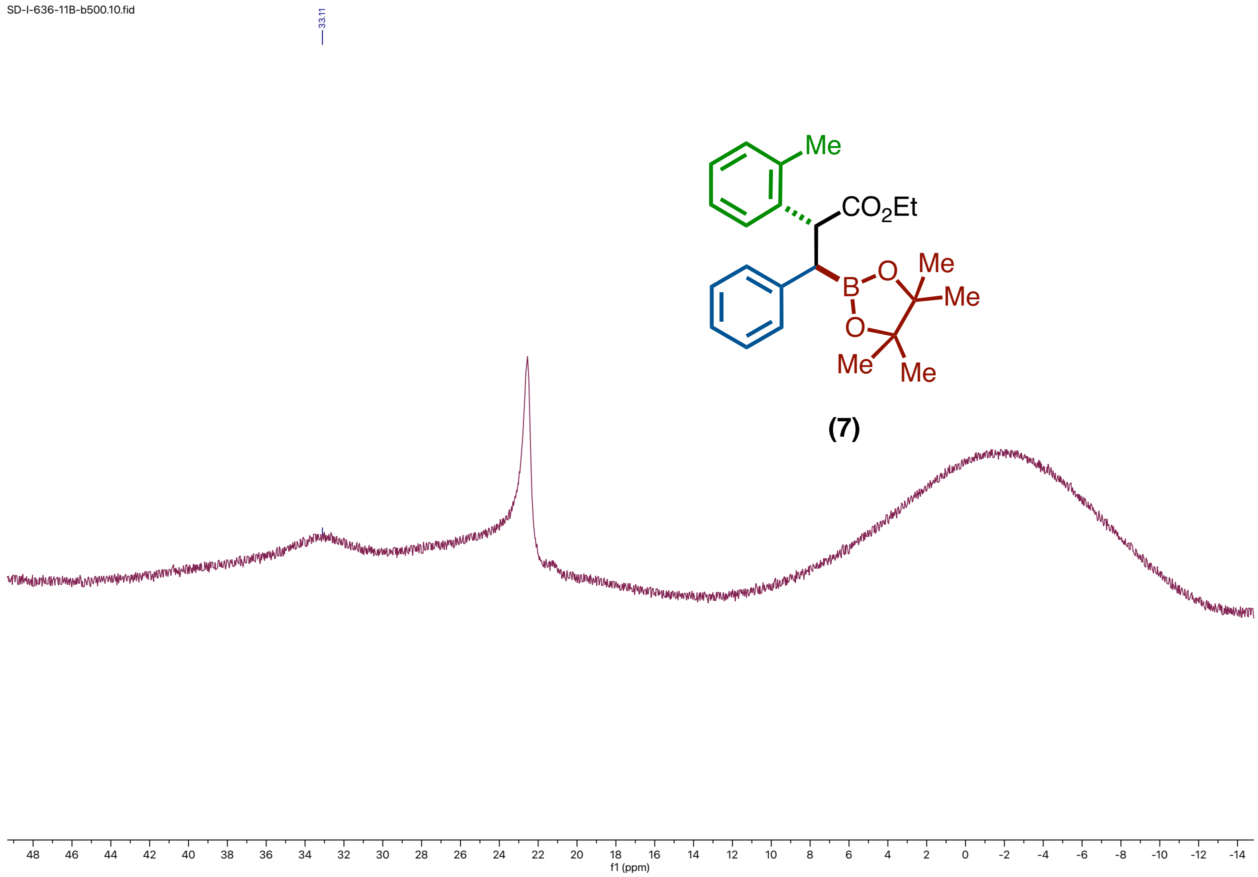
SD-I-636-11B-b500.10.fid
33.11
Me
CO2Et
B
O
O
Me
Me
Me
Me
(7)
f1 (ppm)

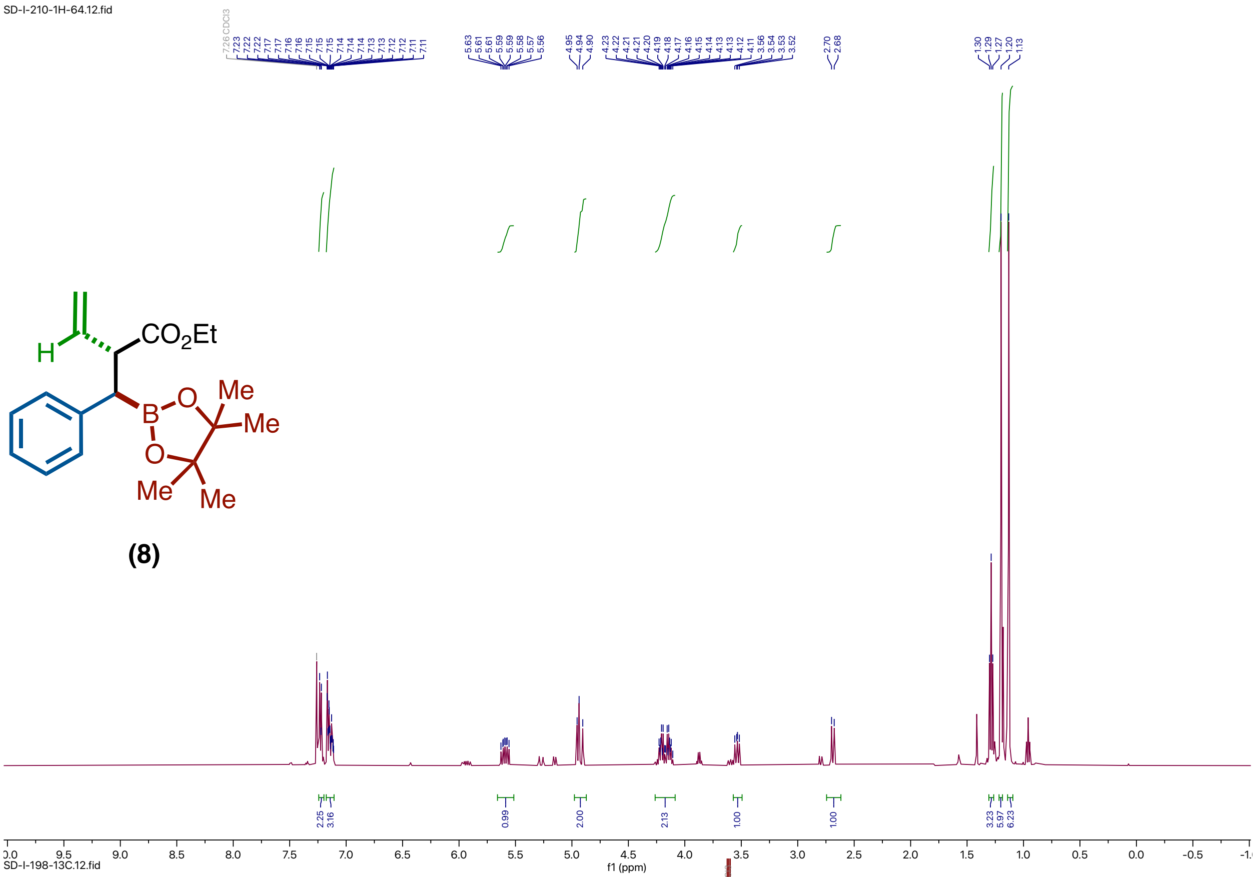
SD-I-210-1H-64.12.fid
7.26 CDCl3
7.23 7.22 7.22 7.17 7.17 7.16 7.16 7.15 7.15 7.15 7.14 7.14 7.14 7.13 7.13 7.13 7.12 7.12 7.11 7.11
5.63 5.61 5.61 5.59 5.59 5.58 5.57 5.56
4.95 4.94 4.90
4.23 4.22 4.21 4.21 4.20 4.19 4.18 4.18 4.17 4.16 4.15 4.14 4.13 4.13 4.12 4.11
3.56 3.54 3.53 3.52
2.70 2.68
1.30 1.29 1.27 1.20 1.13
CO2Et
H
B
O
Me
(8)
2.25 3.16 0.99 2.00 2.13 1.00 1.00 3.23 5.97 6.23
f1 (ppm)

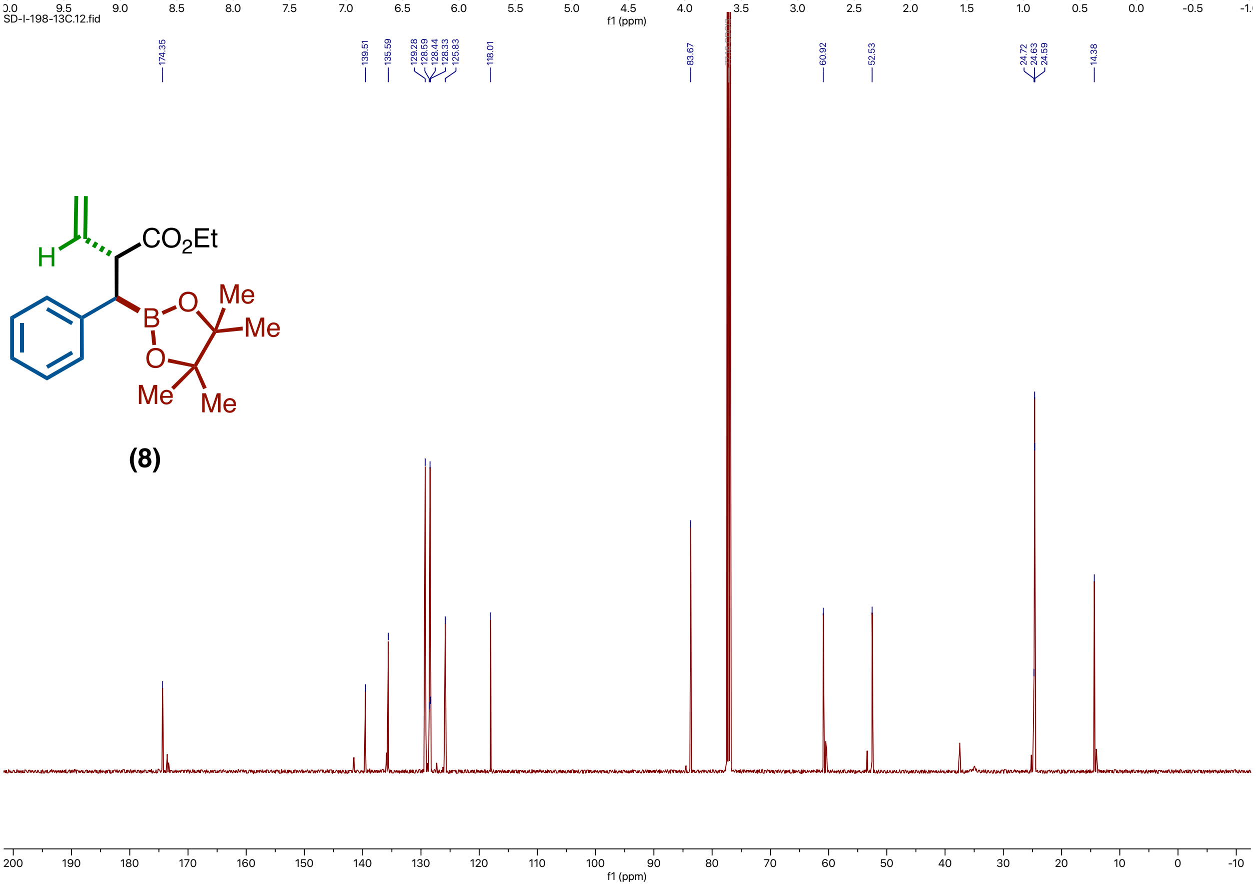
SD-I-198-13C.12.fid
174.35
139.51
135.59
129.28 128.59 128.44 128.33 125.83
118.01
83.67
60.92
52.53
24.72 24.63 24.59
14.38
CO2Et
H
B
O
Me
(8)
f1 (ppm)

SD-I-216-210-11B-b500.10.fid

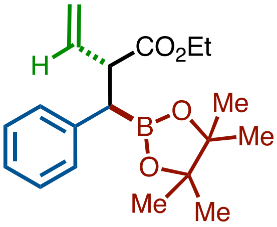

(8)

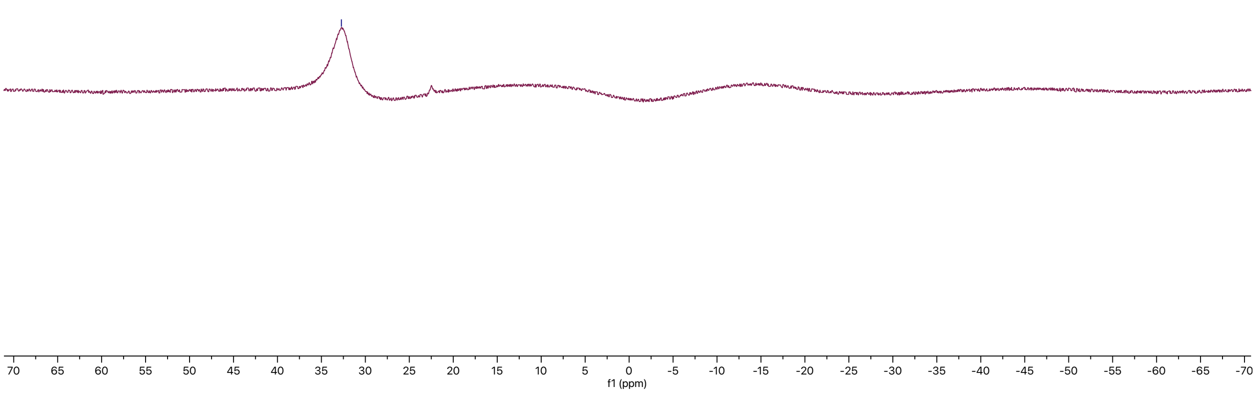

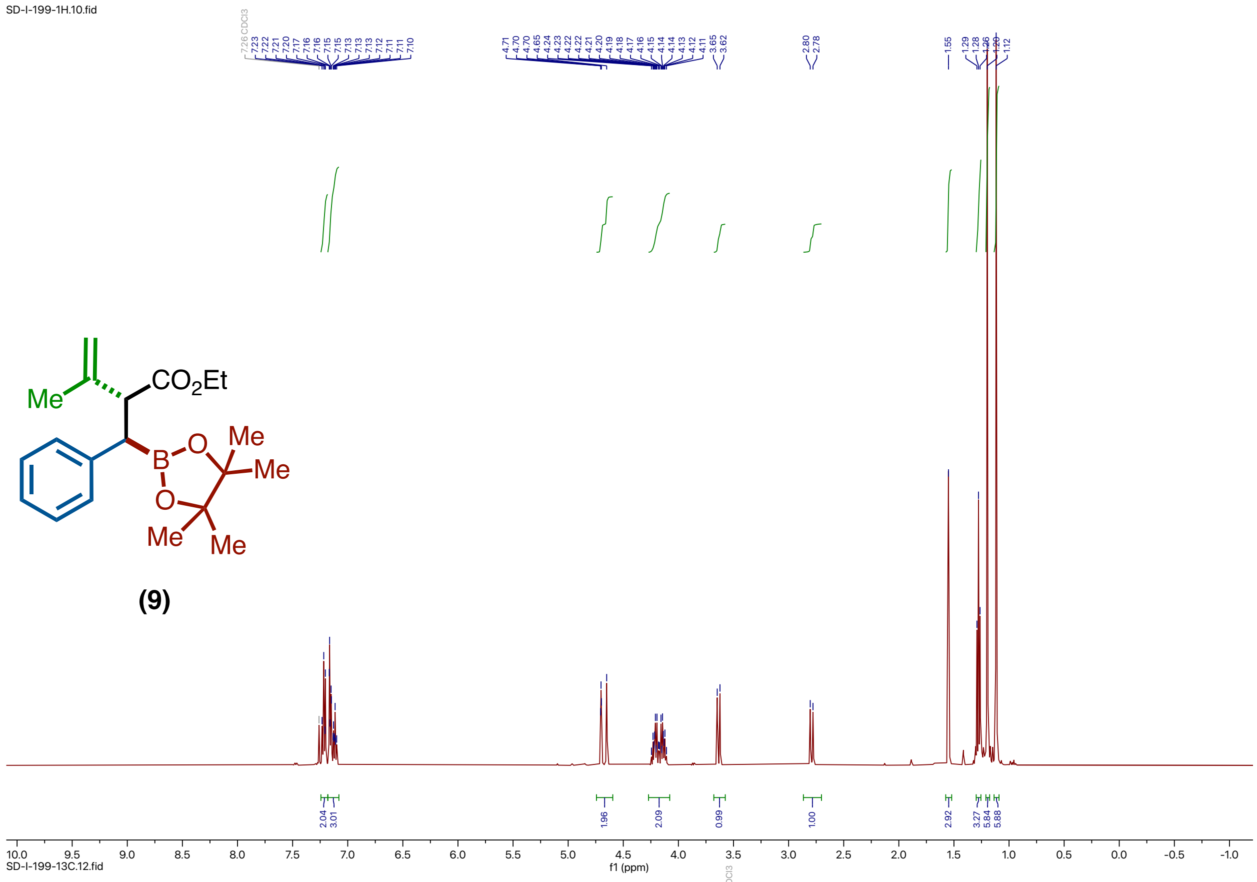
SD-I-199-1H.10.fid
CO2Et
Me
B
O
Me
Me
Me
Me
(9)
f1 (ppm)

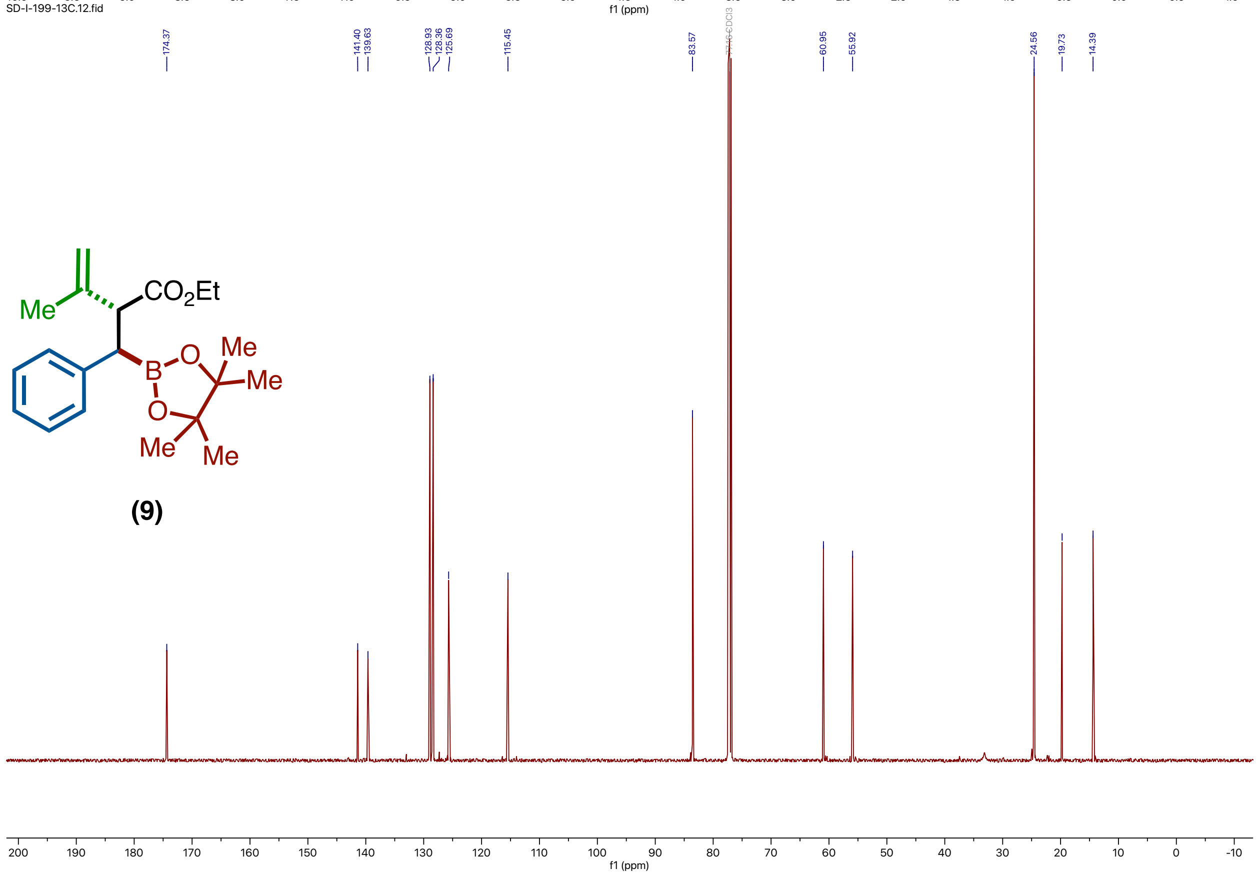
SD-I-199-13C.12.fid
174.37
141.40
139.63
128.93
128.36
125.69
115.45
83.57
60.95
55.92
24.56
19.73
14.39
CO2Et
Me
B
O
Me
Me
Me
Me
(9)
f1 (ppm)

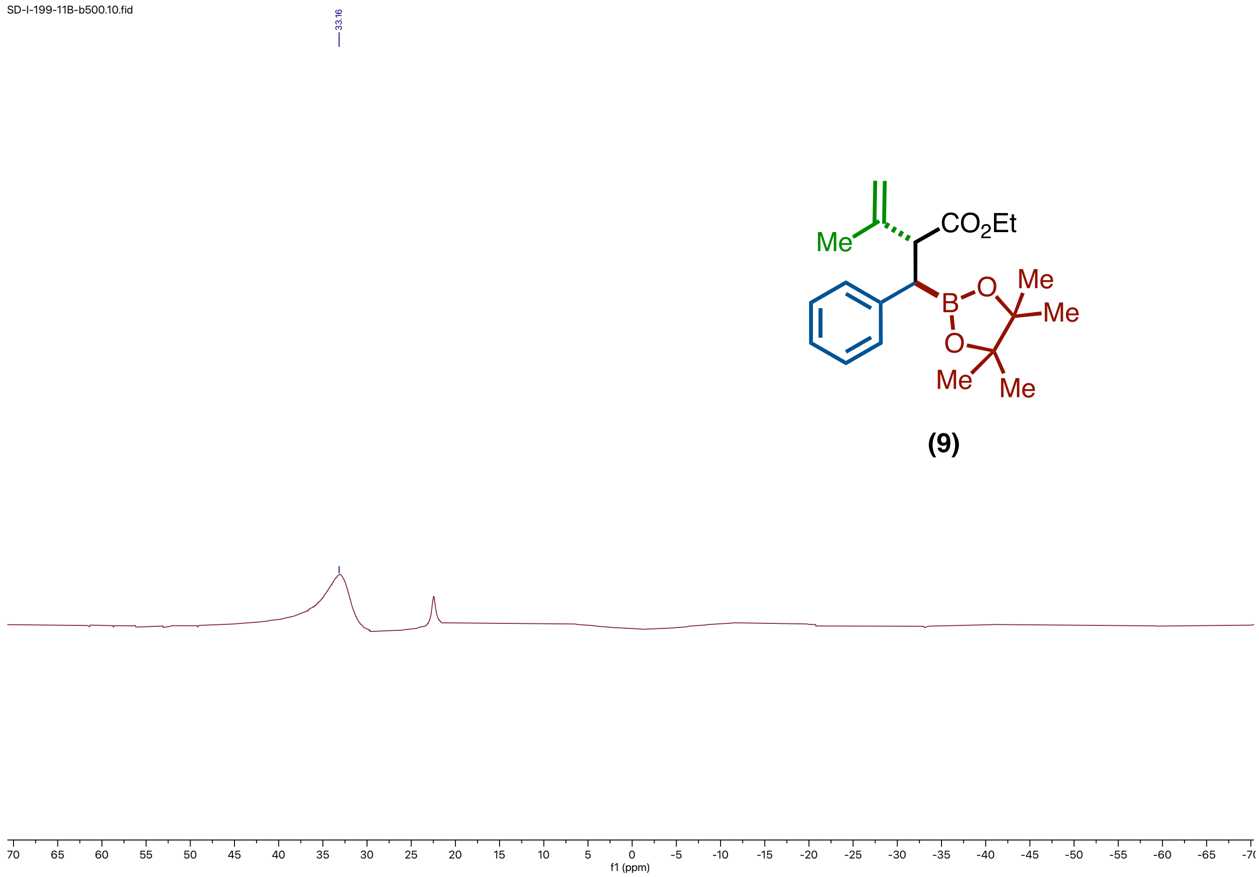
SD-I-199-11B-b500.10.fid
33.16
70 65 60 55 50 45 40 35 30 25 20 15 10 5 0 -5 -10 -15 -20 -25 -30 -35 -40 -45 -50 -55 -60 -65
f1 (ppm)
CO2Et
Me
B
O
O
Me
Me
Me
Me

(9)

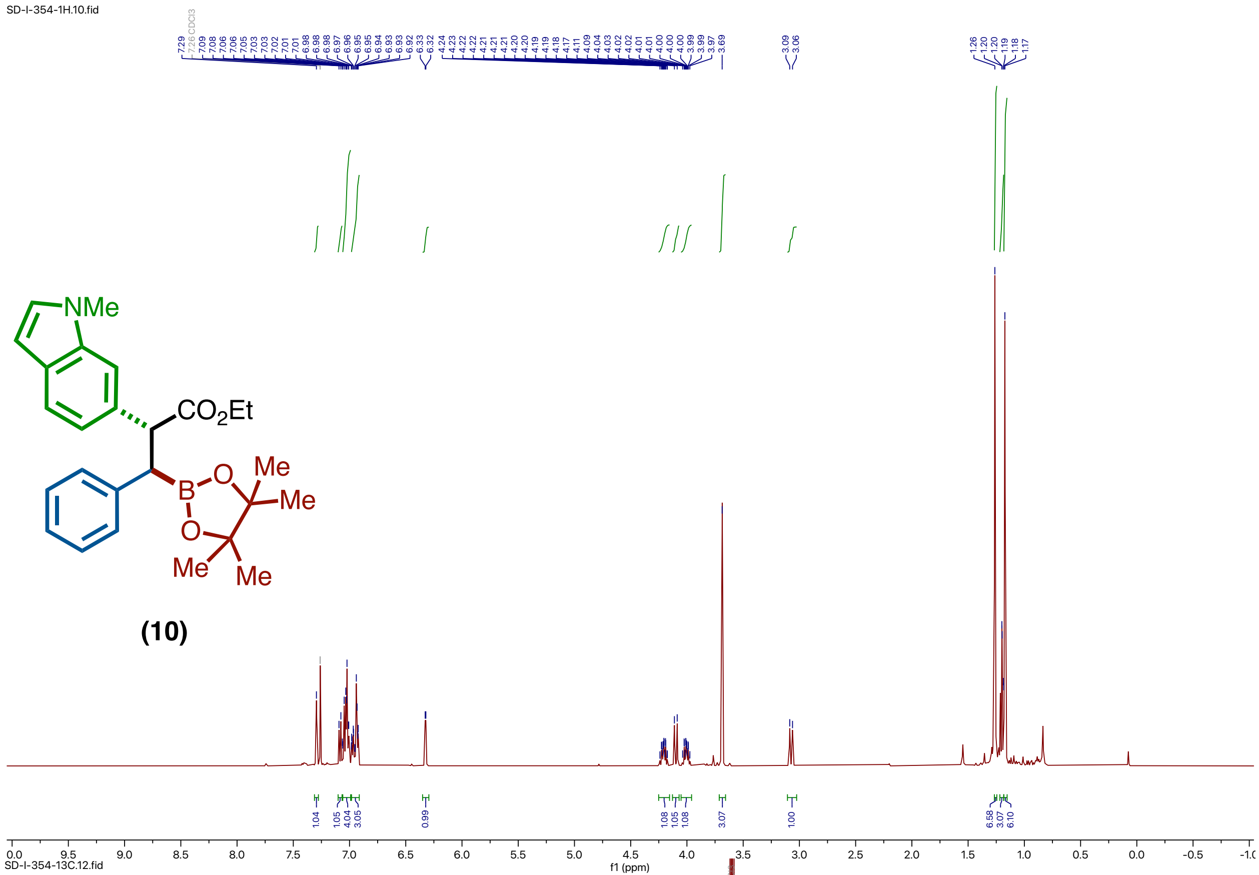
SD-I-354-1H.10.fid
NMe
CO2Et
Me
Me
Me
Me
(10)
f1 (ppm)

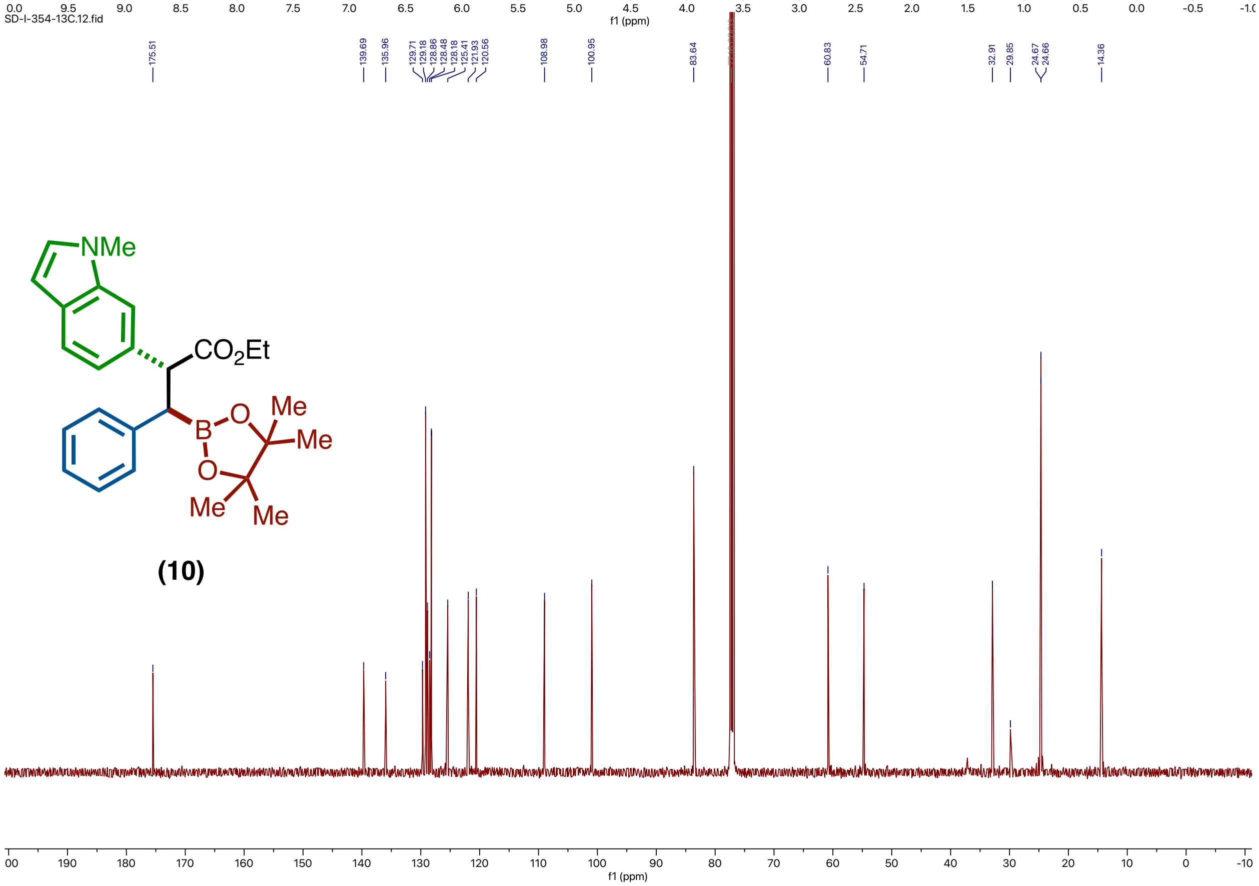
SD-I-354-13C.12.fid
175.51
139.69
135.96
129.71
129.18
128.86
128.48
128.18
125.41
121.93
120.56
108.98
100.95
83.64
60.83
54.71
32.91
29.85
24.67
24.66
14.36
NMe
CO2Et
Me
Me
Me
Me
(10)
f1 (ppm)

SD-I-354-11B-New.10.fid

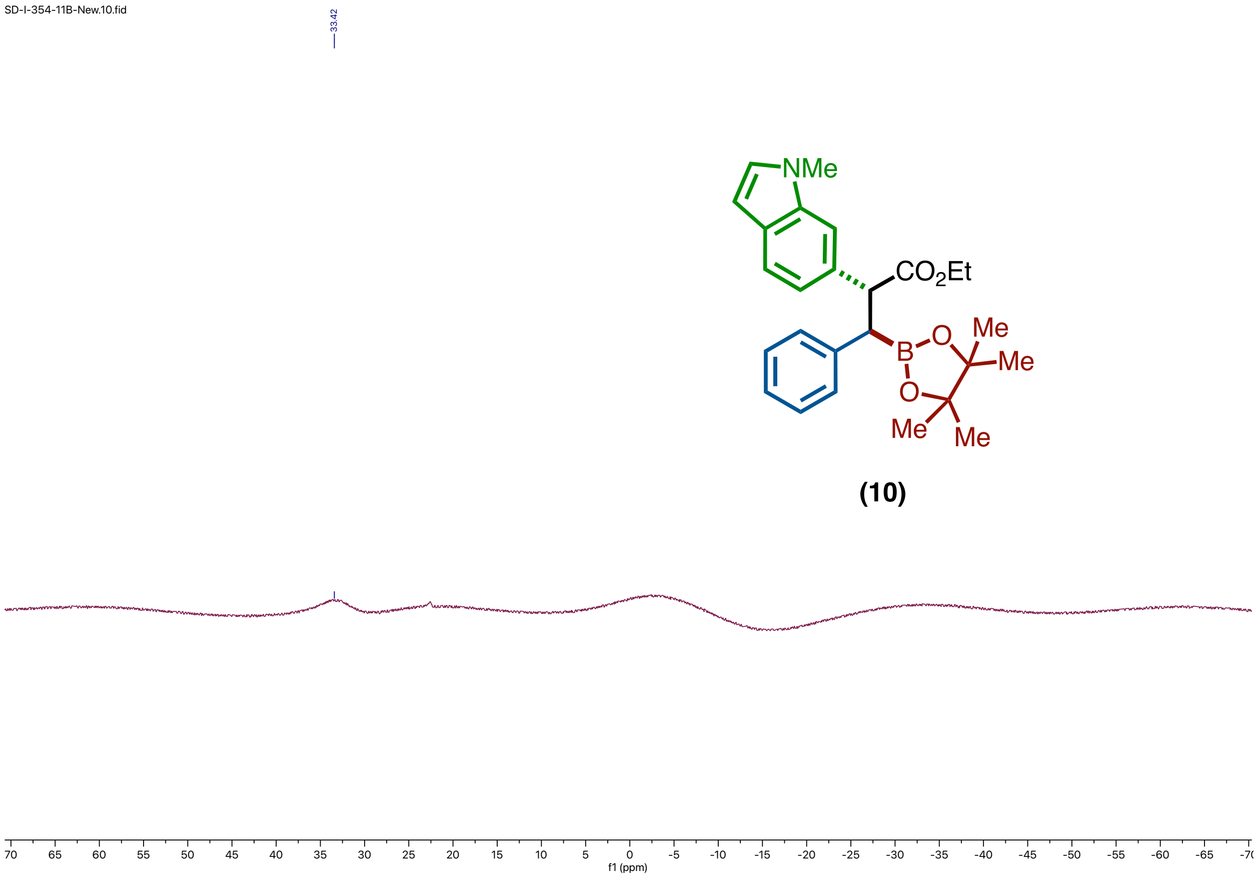

(10)

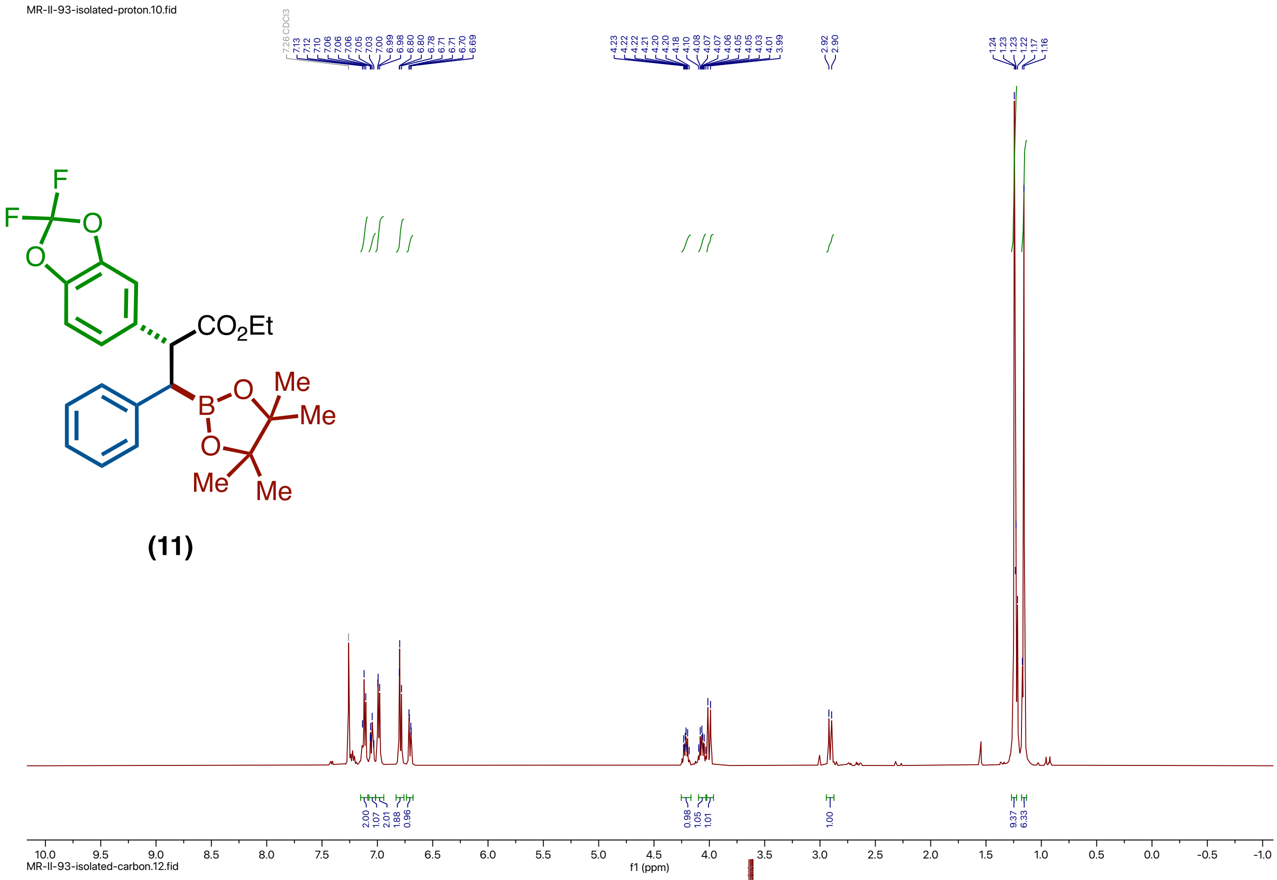
MR-II-93-isolated-proton.10.fid
f1 (ppm)
(11)

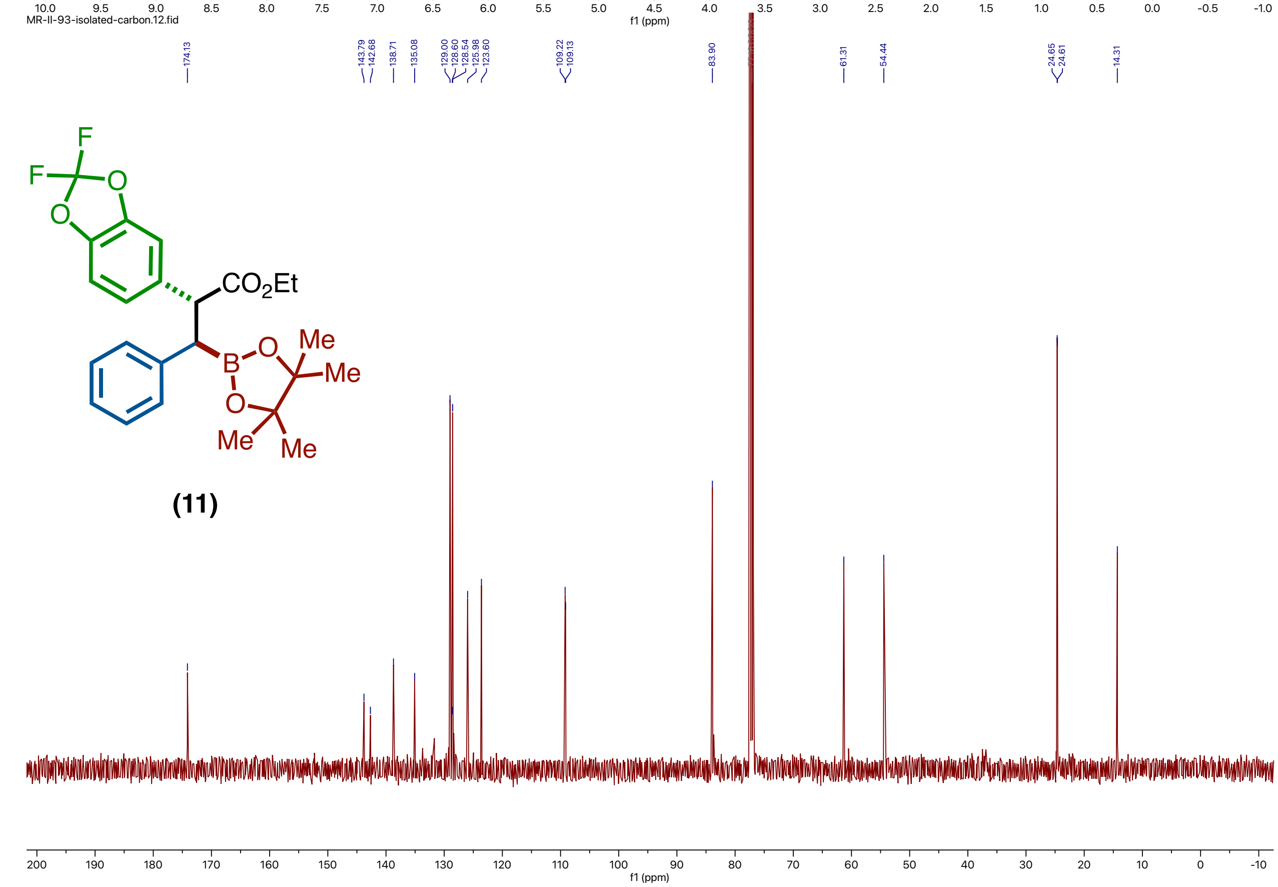
MR-II-93-isolated-carbon.12.fid
f1 (ppm)
(11)

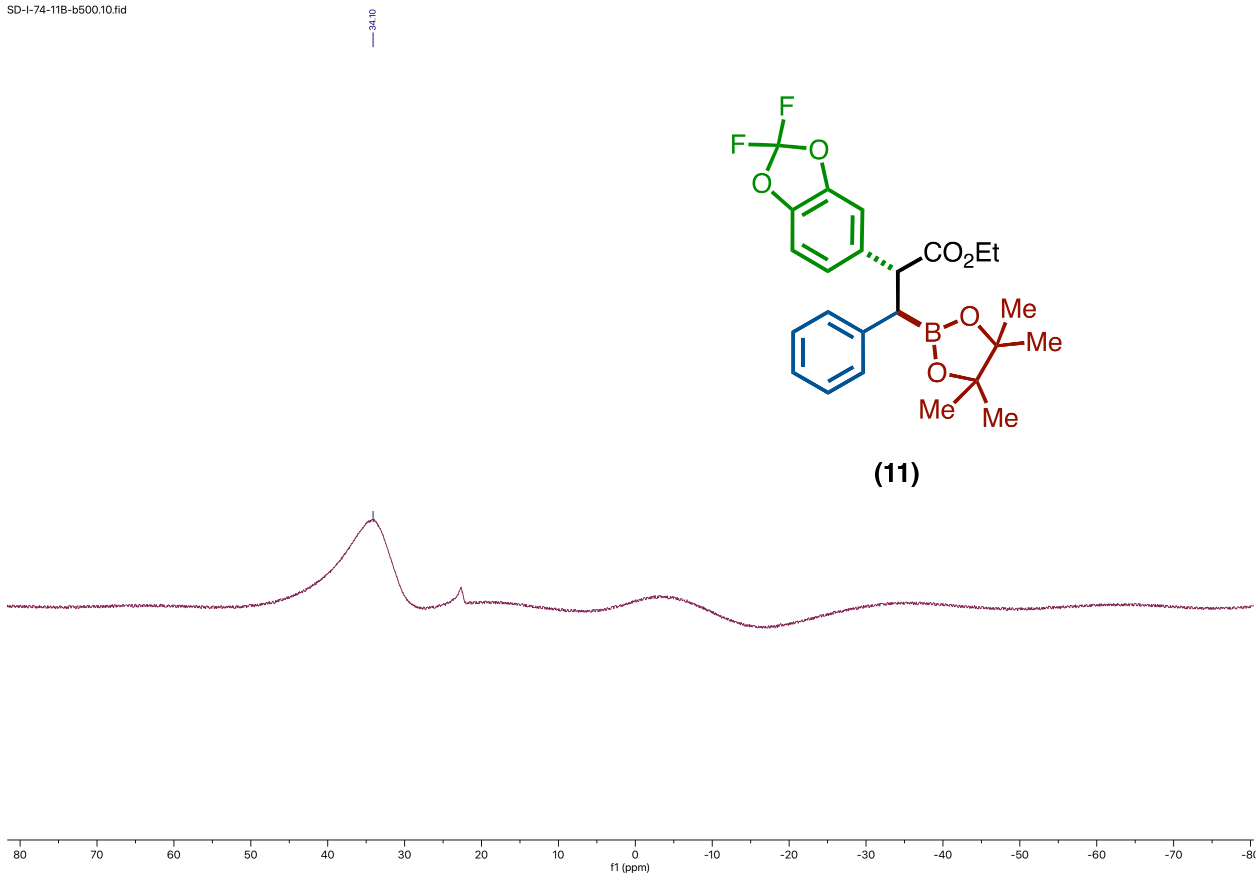
SD-I-74-11B-b500.10.fid
34.10
F
F
O
O
CO2Et
B
O
O
Me
Me
Me
Me
(11)
80 70 60 50 40 30 20 10 0 -10 -20 -30 -40 -50 -60 -70 -80
f1 (ppm)

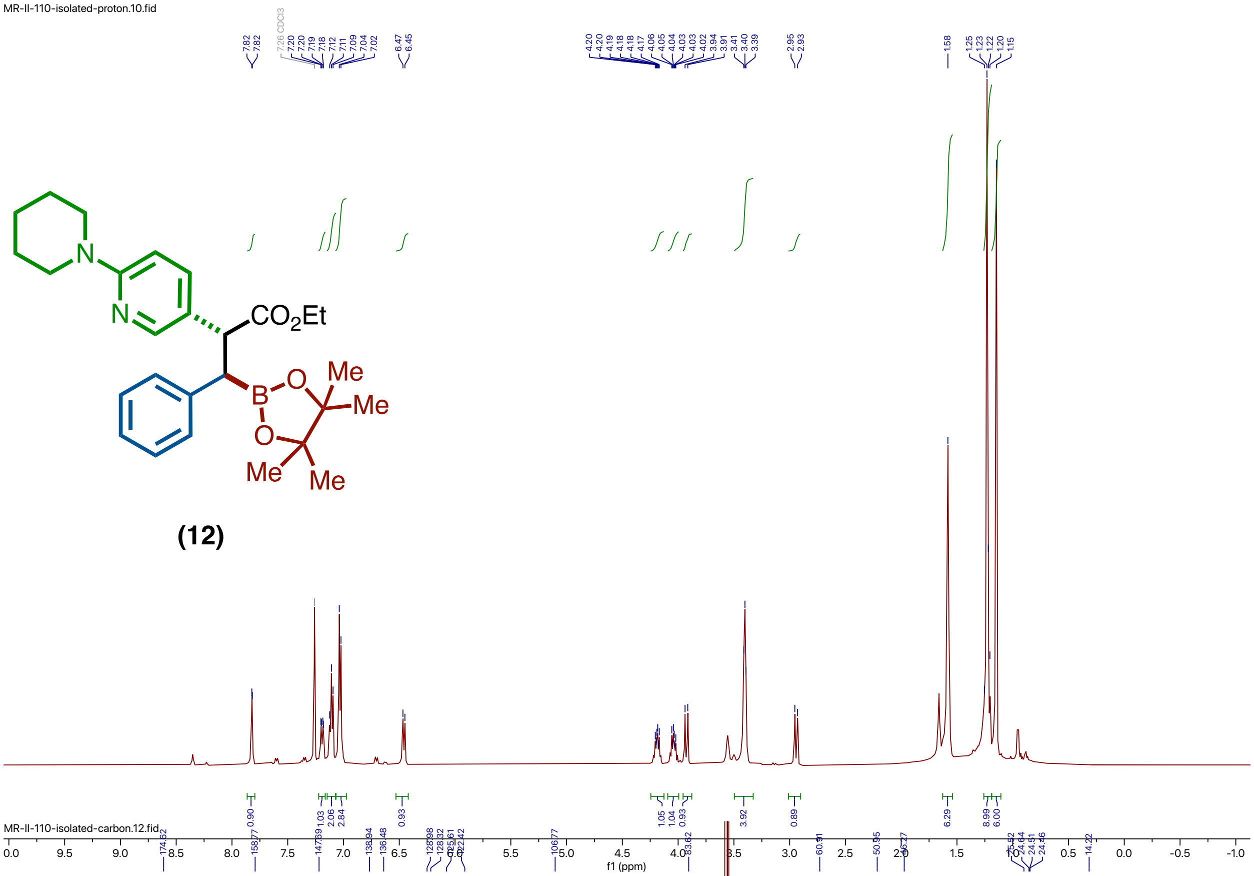
MR-II-110-isolated-proton.10.fid
7.26 CDCl3
CO_2Et
(12)
f1 (ppm)

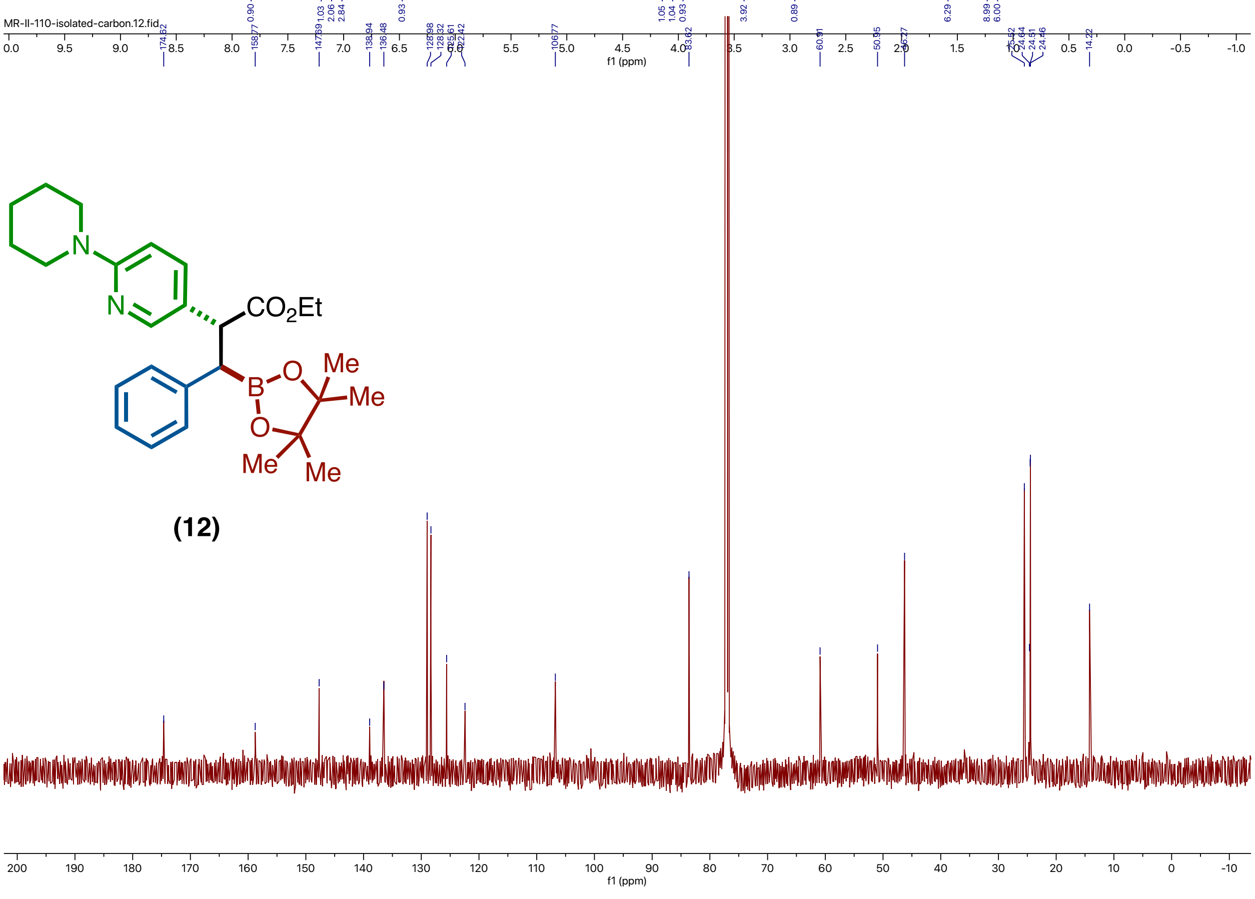
MR-II-110-isolated-carbon.12.fid
CO_2Et
(12)
f1 (ppm)

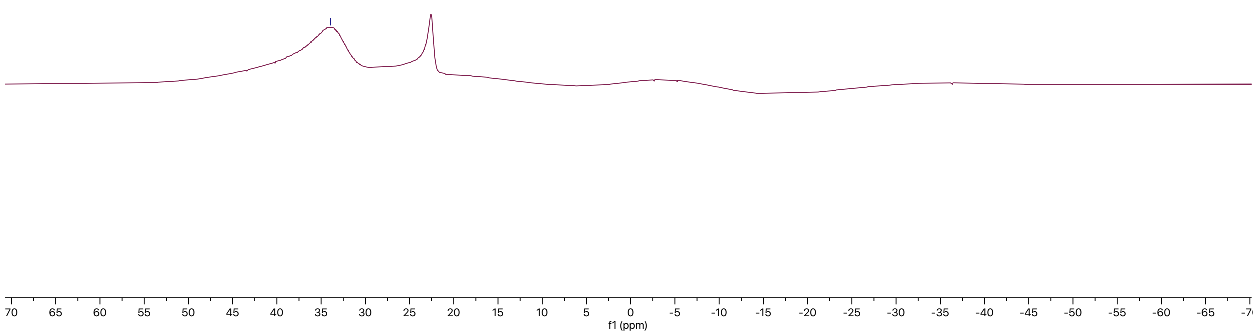
SD-I-60-11B-b500.10.fid
33.97
70 65 60 55 50 45 40 35 30 25 20 15 10 5 0 -5 -10 -15 -20 -25 -30 -35 -40 -45 -50 -55 -60 -65 -70
f1 (ppm)

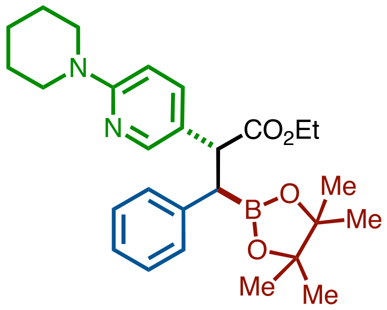
N
N
CO2Et
B
O
O
Me
Me
Me
Me

(12)

SD-I-681-B-1H-12.10.fid

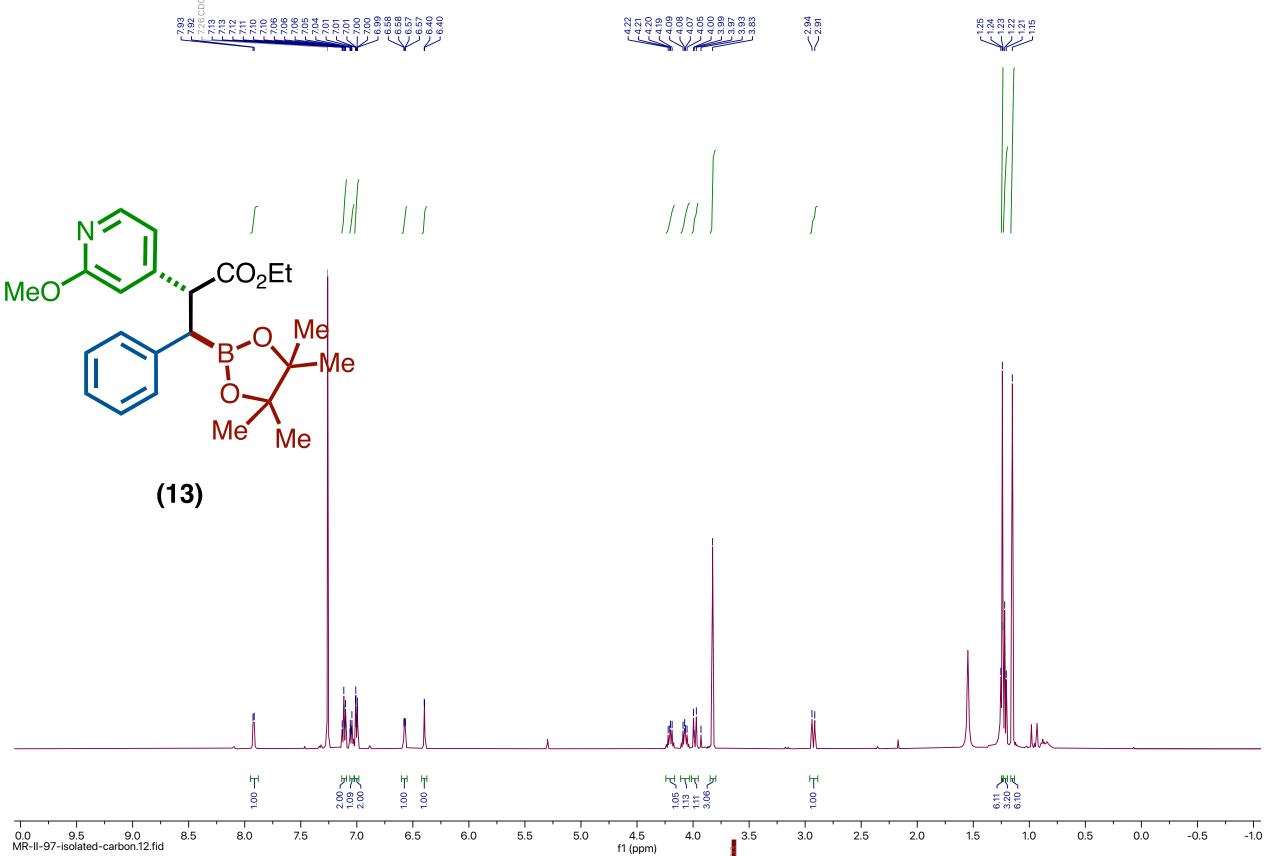

MR-II-97-isolated-carbon.12.fid

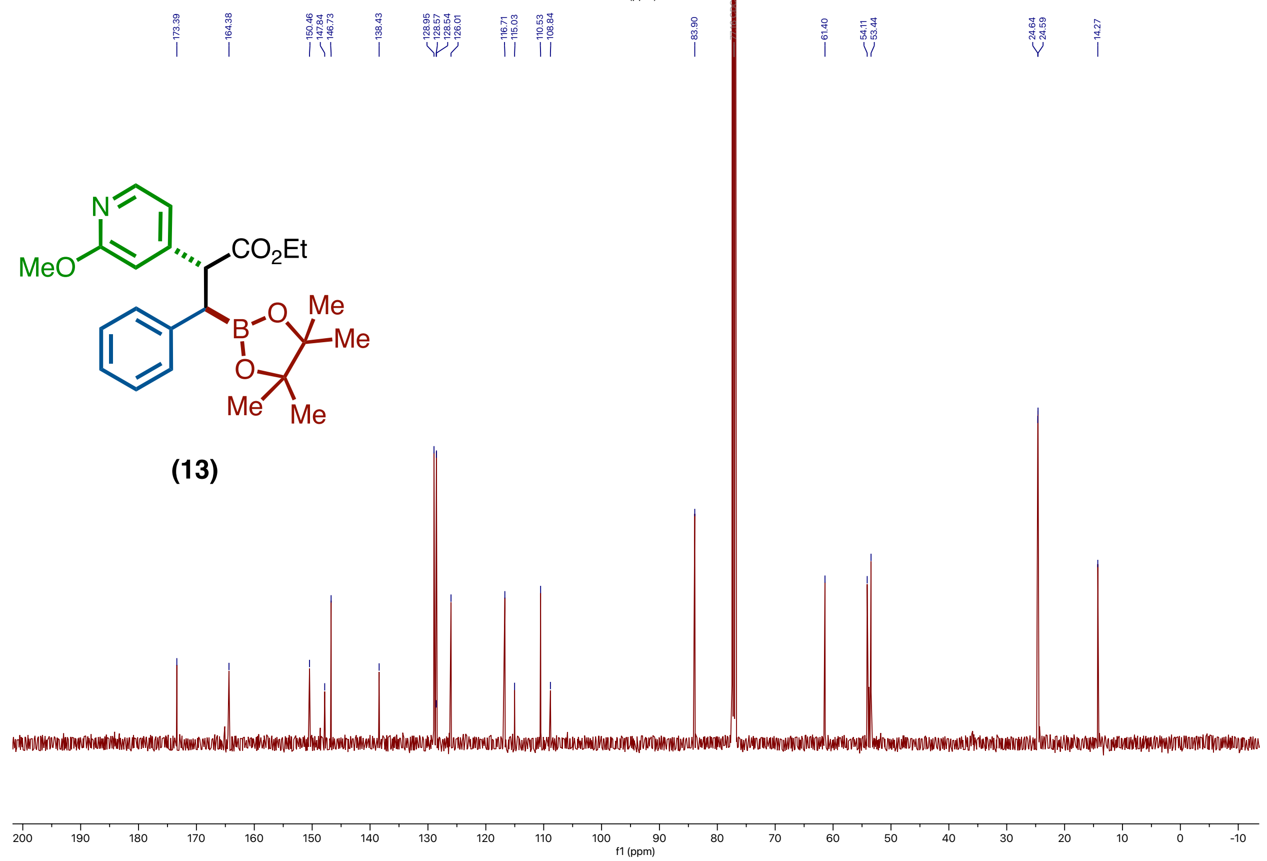

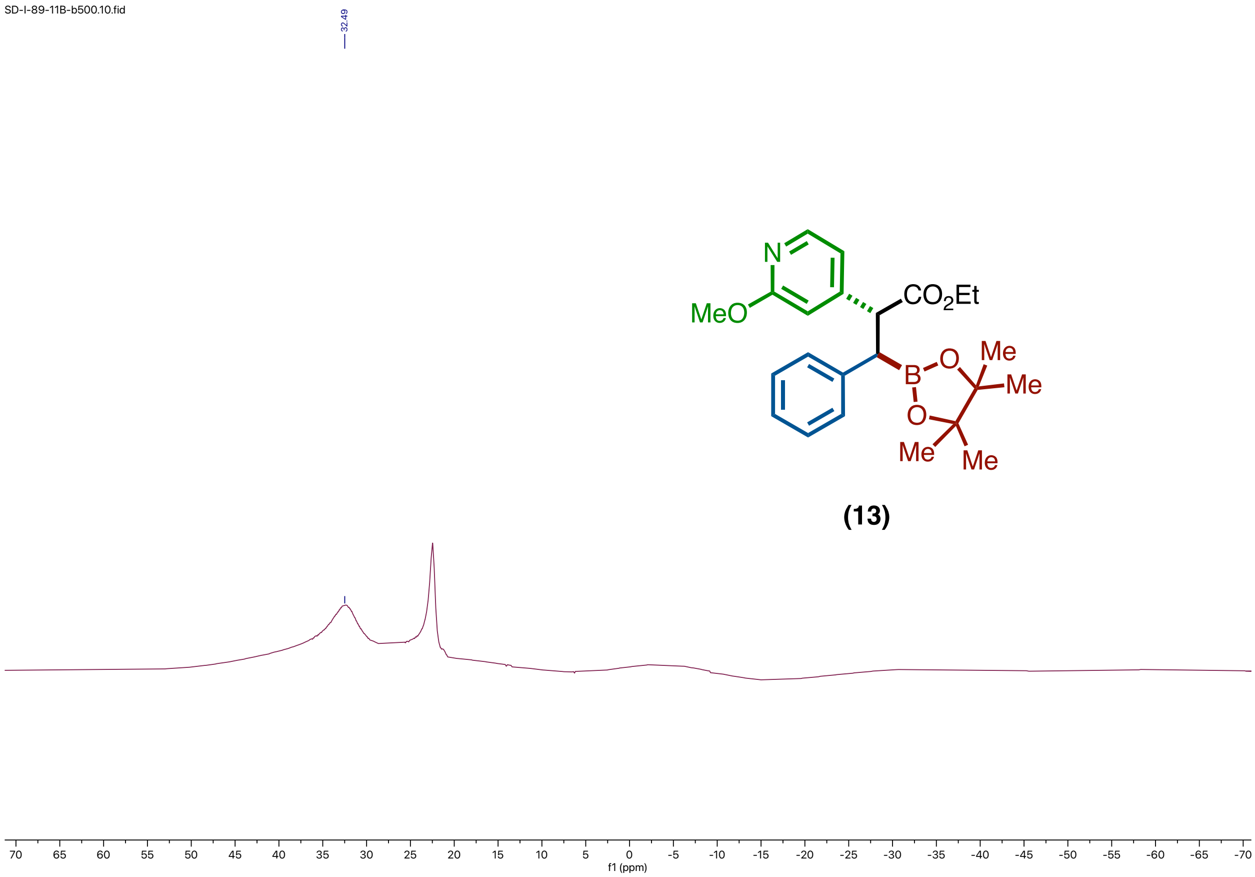
SD-I-89-11B-b500.10.fid
32.49
MeO
N
CO2Et
B
O
O
Me
Me
Me
Me
(13)
70 65 60 55 50 45 40 35 30 25 20 15 10 5 0 -5 -10 -15 -20 -25 -30 -35 -40 -45 -50 -55 -60 -65 -70
f1 (ppm)

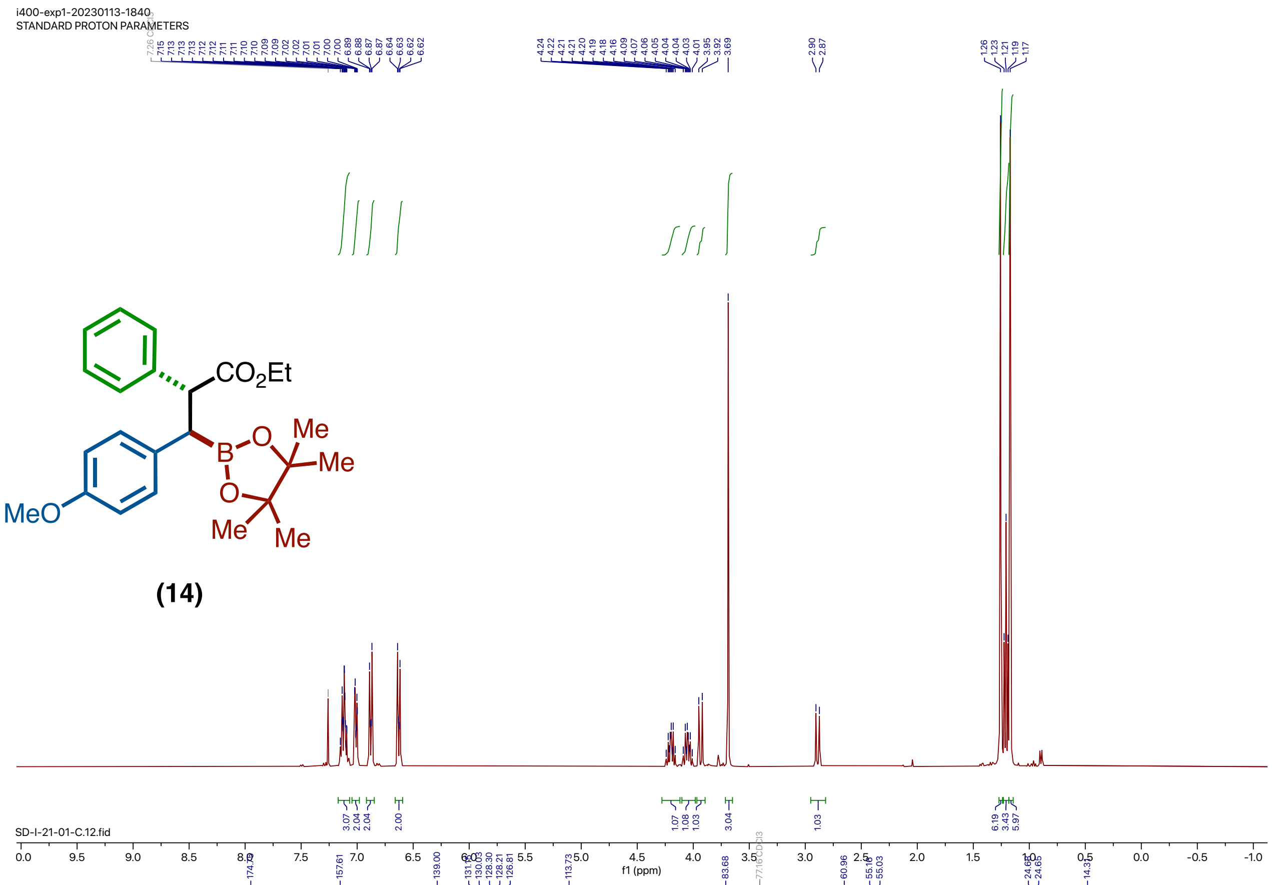
i400-exp1-20230113-1840
STANDARD PROTON PARAMETERS
SD-I-21-01-C.12.fid
f1 (ppm)
CO2Et
MeO
Me
(14)

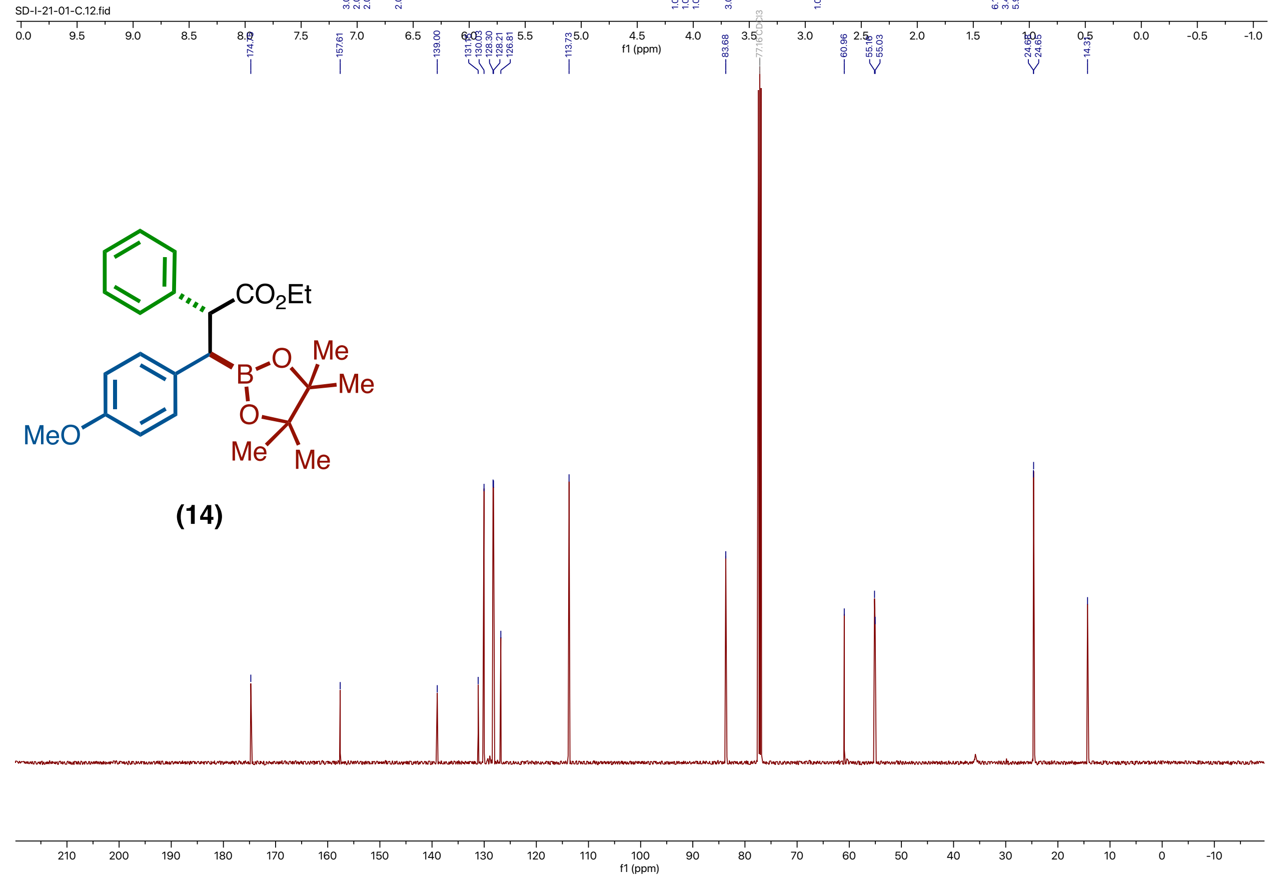
f1 (ppm)
77.16 CDCl3
CO2Et
MeO
Me
(14)

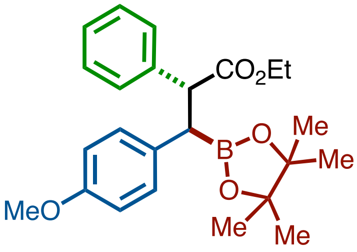
CO2Et
B
O
O
Me
Me
Me
Me
MeO

(14)

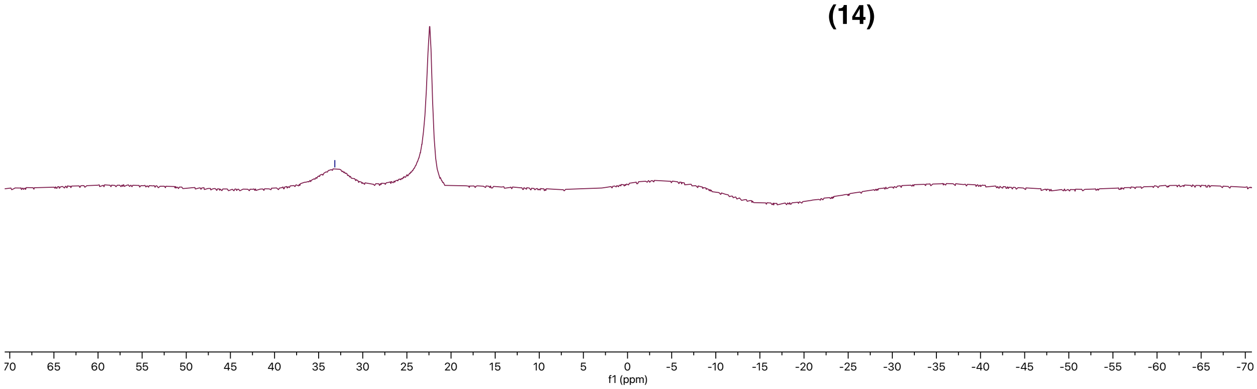
SD-I-18-11B-b500.10.fid
33.17
70
65
60
55
50
45
40
35
30
25
20
15
10
5
0
-5
-10
-15
-20
-25
-30
-35
-40
-45
-50
-55
-60
-65
-70
f1 (ppm)

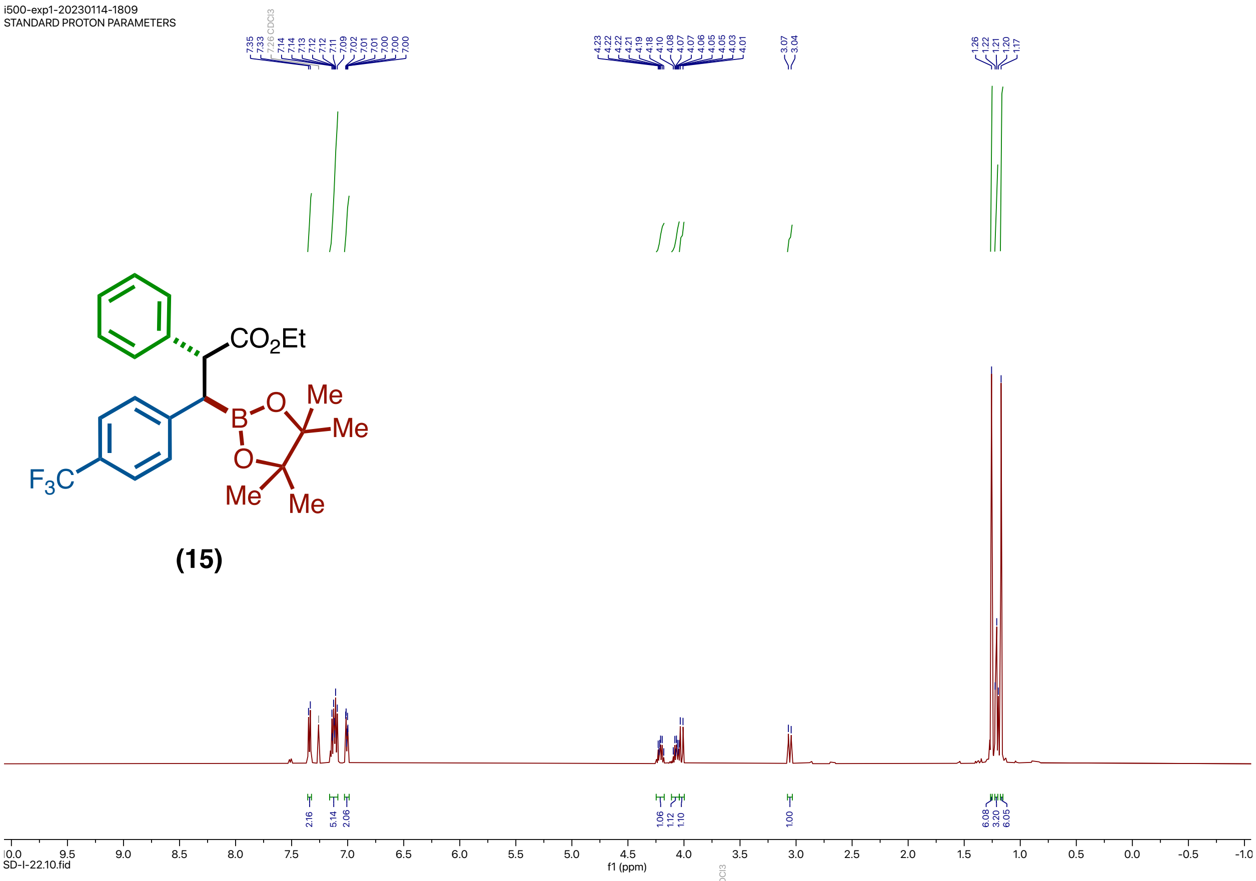
i500-exp1-20230114-1809
STANDARD PROTON PARAMETERS
7.35 7.33 7.26 CDCl3 7.14 7.14 7.13 7.12 7.12 7.11 7.09 7.02 7.01 7.01 7.00 7.00 7.00
4.23 4.22 4.22 4.21 4.19 4.18 4.10 4.08 4.07 4.07 4.06 4.05 4.05 4.03 4.01
3.07 3.04
1.26 1.22 1.21 1.20 1.17
CO_2Et
B O O Me Me Me Me
F_3C
(15)
2.16 5.14 2.06 1.06 1.12 1.10 1.00 6.08 3.20 6.05
f1 (ppm)

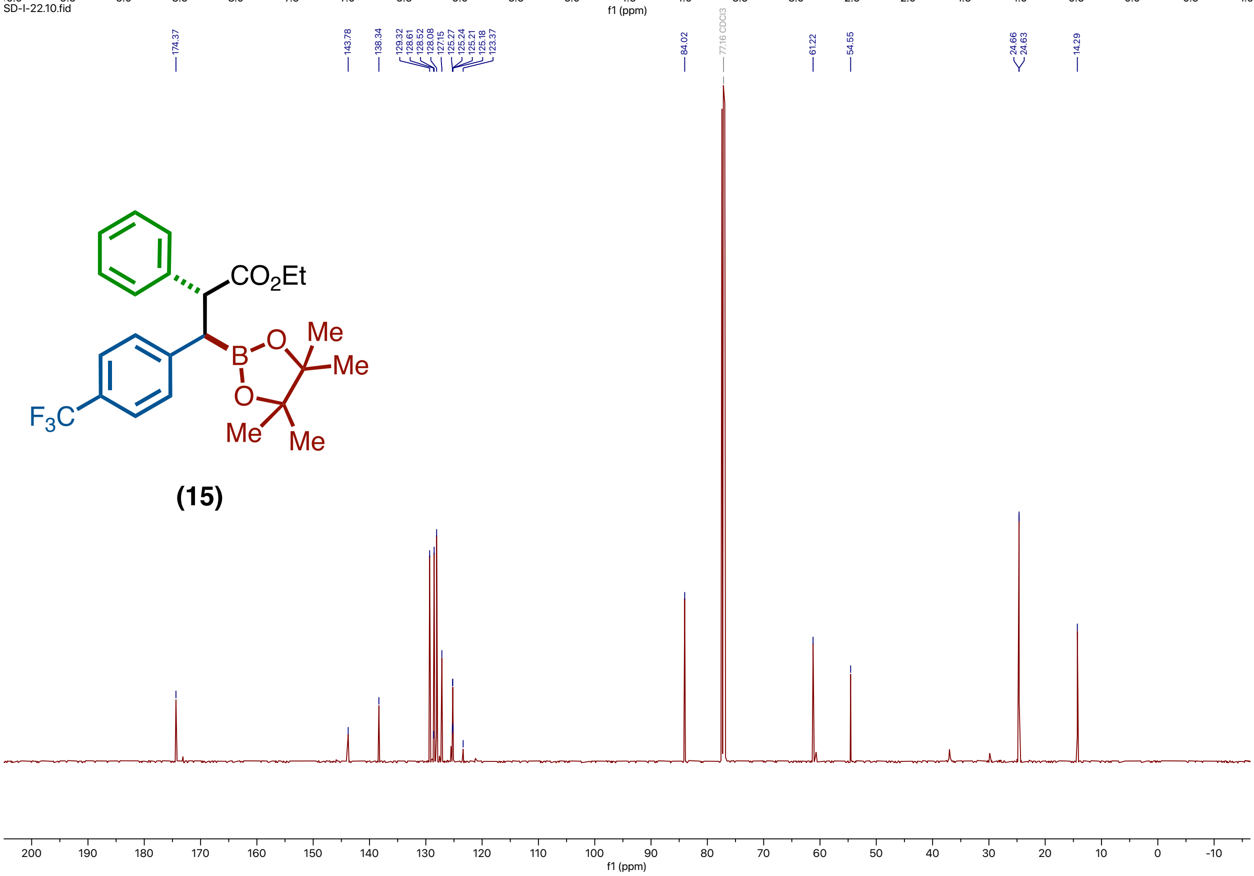
SD-I-22.10.fid
174.37 143.78 138.34 129.32 128.61 128.52 128.08 127.15 125.27 125.24 125.21 125.18 123.37 84.02 77.16 CDCl3 61.22 54.55 24.66 24.63 14.29
CO_2Et
B O O Me Me Me Me
F_3C
(15)
f1 (ppm)

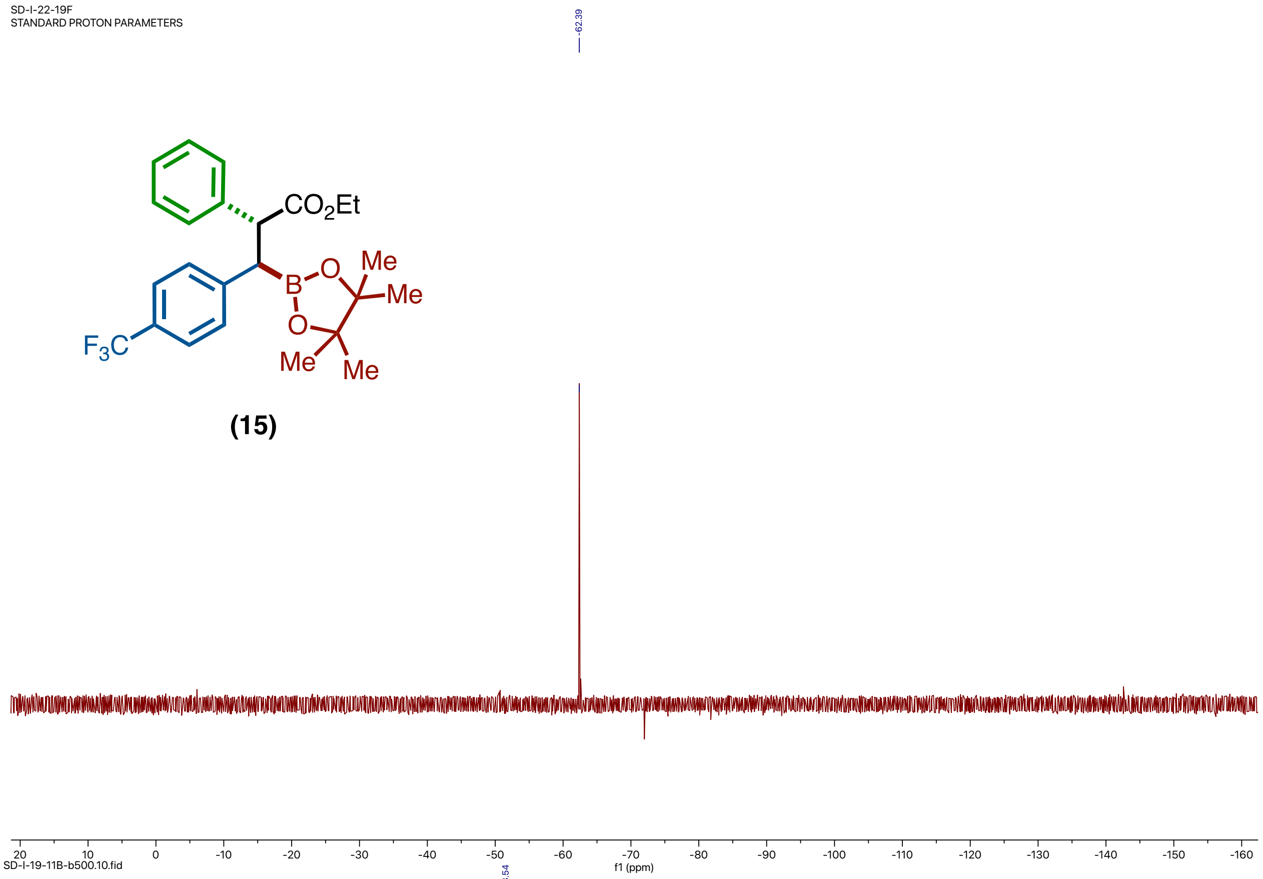
SD-I-22-19F
STANDARD PROTON PARAMETERS
-62.39
CO2Et
B
O
O
Me
Me
Me
Me
F3C
20 10 0 -10 -20 -30 -40 -50 -60 -70 -80 -90 -100 -110 -120 -130 -140 -150 -160
f1 (ppm)

(15)

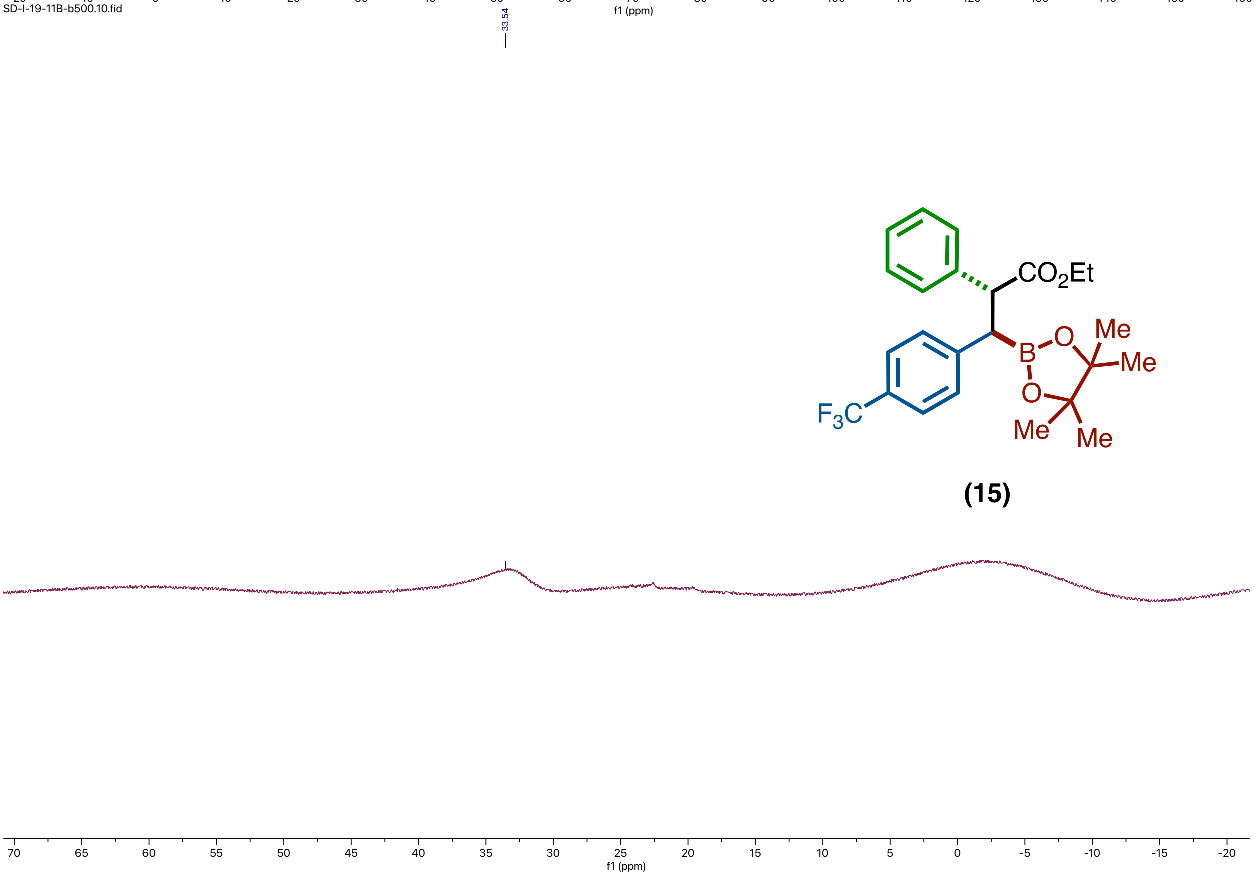
SD-I-19-11B-b500.10.fid
33.54
CO2Et
B
O
O
Me
Me
Me
Me
F3C
70 65 60 55 50 45 40 35 30 25 20 15 10 5 0 -5 -10 -15 -20
f1 (ppm)

(15)

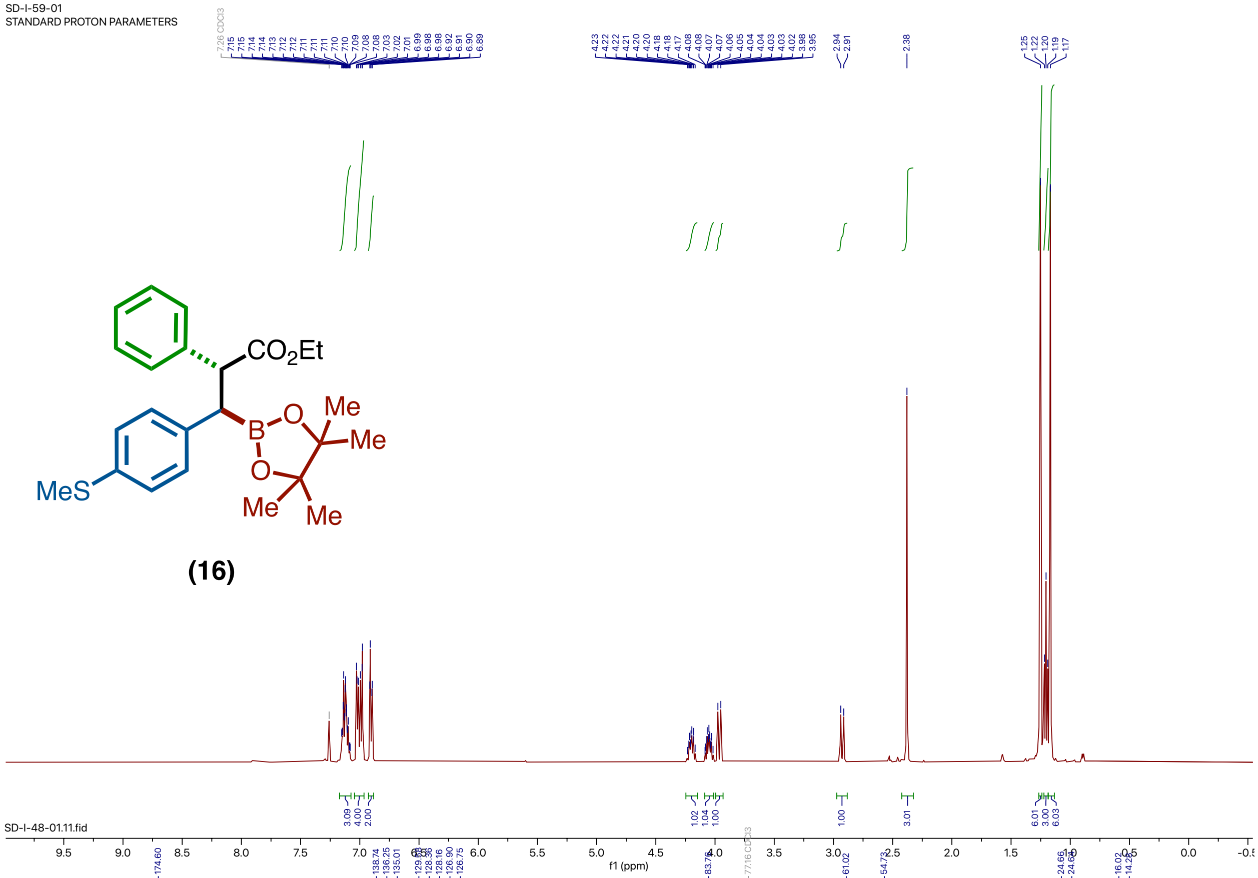
SD-I-59-01
STANDARD PROTON PARAMETERS
MeS
CO2Et
B
O
O
Me
Me
Me
Me
(16)
3.09
4.00
2.00
1.02
1.04
1.00
1.00
3.01
6.01
3.00
6.03
2.94
2.91
2.38
f1 (ppm)

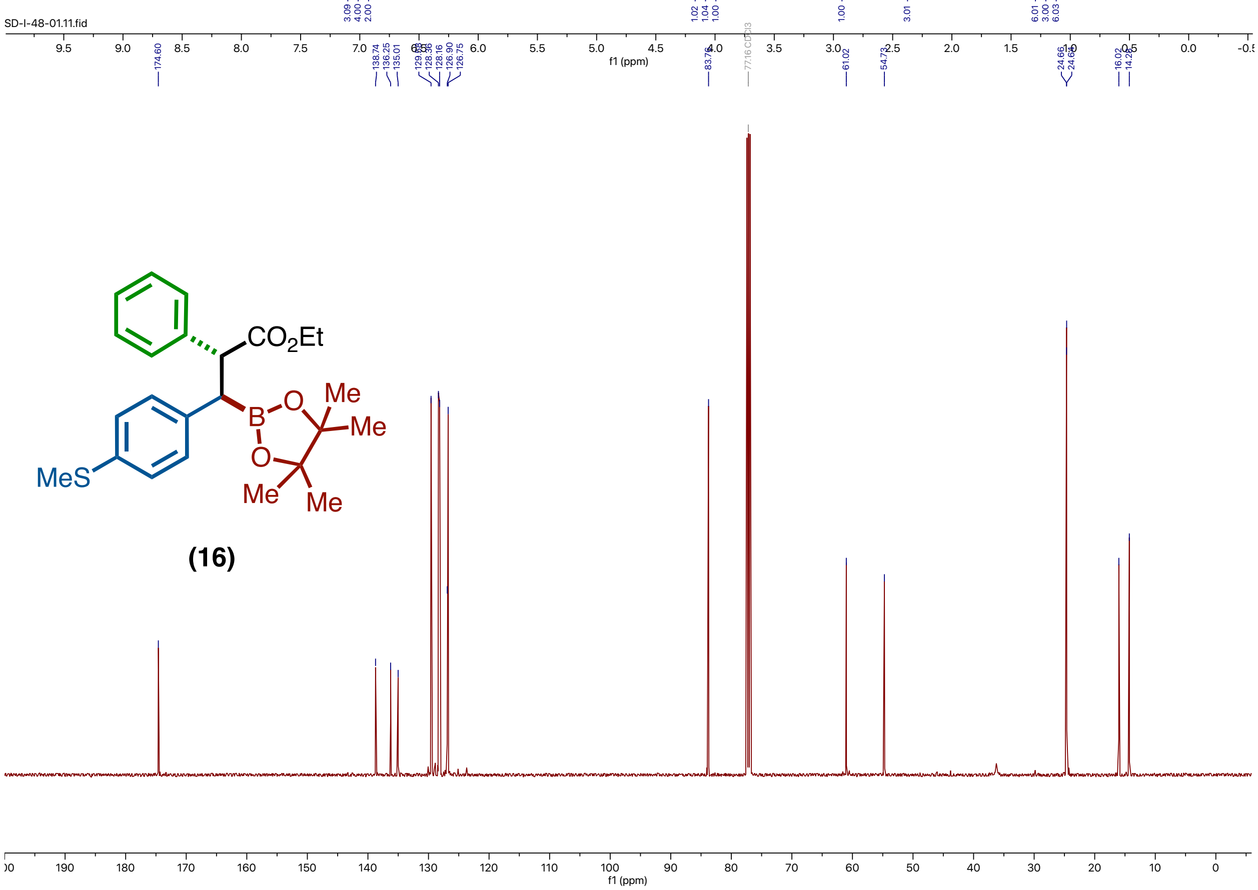
SD-I-48-01.11.fid
174.60
138.74
136.25
135.01
83.76
77.16 CDCl3
61.02
54.73
16.02
14.26
MeS
CO2Et
B
O
O
Me
Me
Me
Me
(16)
f1 (ppm)

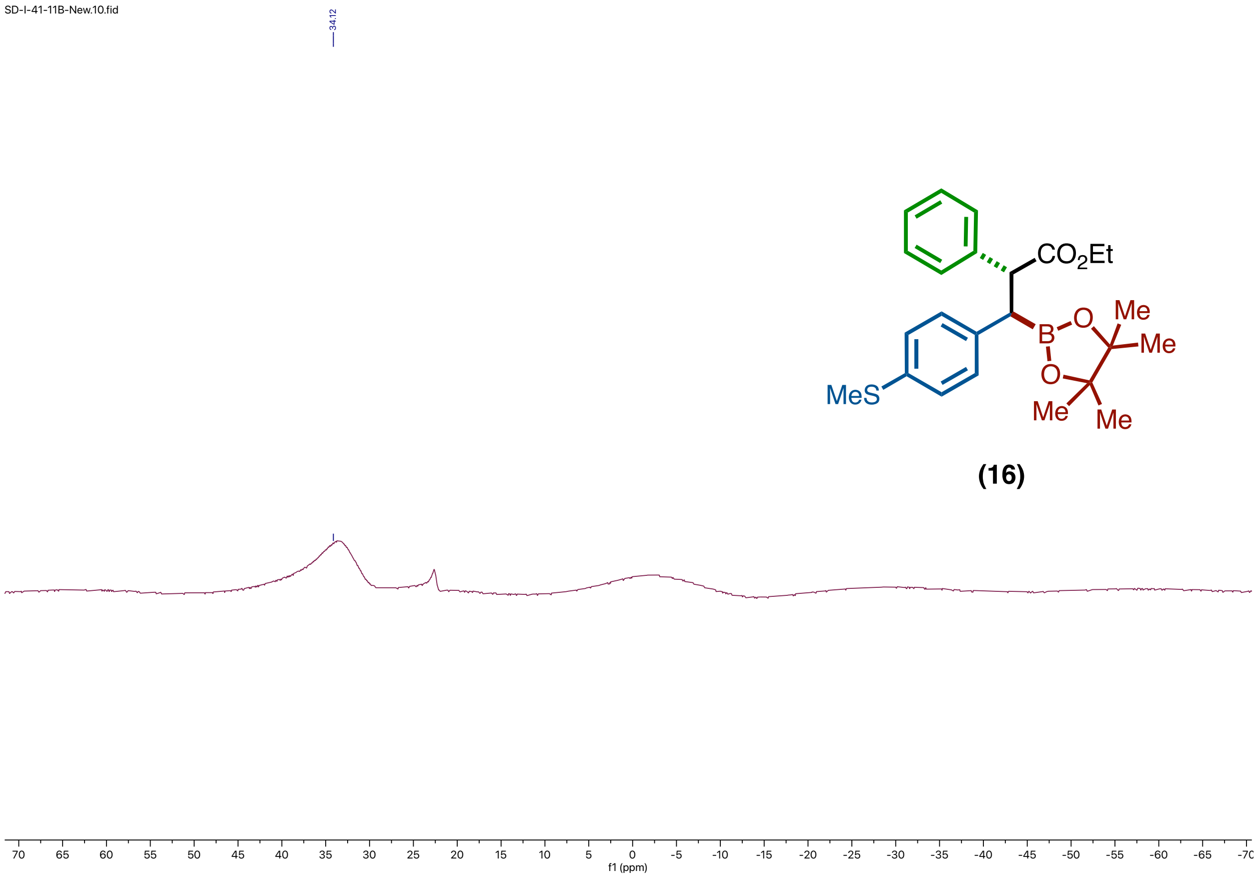
SD-I-41-11B-New.10.fid
34.12
CO2Et
B
O
O
Me
Me
Me
Me
MeS
(16)
70 65 60 55 50 45 40 35 30 25 20 15 10 5 0 -5 -10 -15 -20 -25 -30 -35 -40 -45 -50 -55 -60 -65 -70
f1 (ppm)

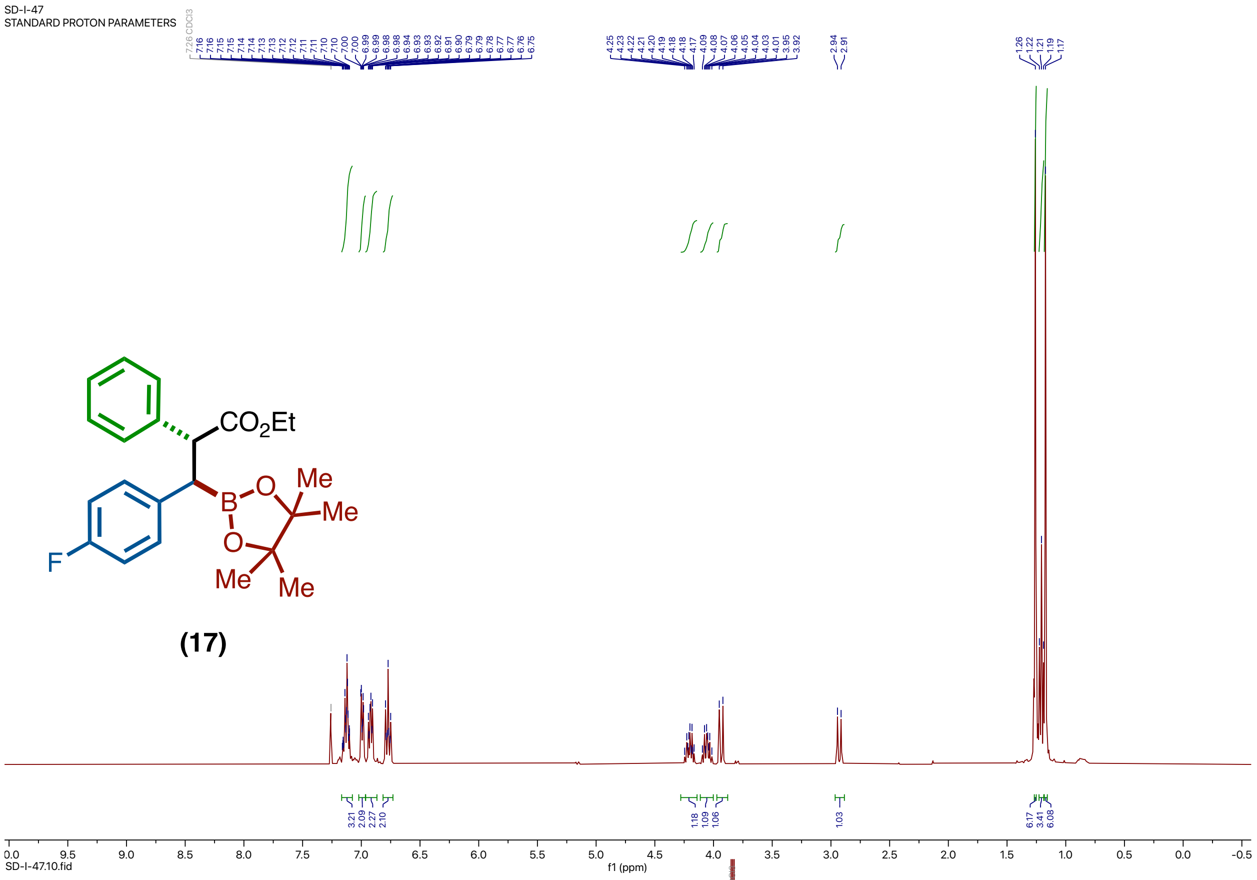

SD-I-47
STANDARD PROTON PARAMETERS
7.26 CDCl3
7.16 7.16 7.15 7.14 7.14 7.13 7.12 7.12 7.11 7.11 7.10 7.10 7.00 6.99 6.99 6.98 6.98 6.94 6.93 6.93 6.92 6.91 6.90 6.79 6.79 6.78 6.77 6.77 6.76 6.75
4.25 4.23 4.22 4.21 4.20 4.19 4.18 4.18 4.17 4.09 4.08 4.07 4.06 4.05 4.04 4.03 4.01 3.95 3.92
2.94 2.91
1.26 1.22 1.21 1.19 1.17
CO2Et
B
O
O
Me
Me
Me
Me
F
(17)
3.21 2.09 2.27 2.10
1.18 1.09 1.06
1.03
6.17 3.41 6.08
f1 (ppm)

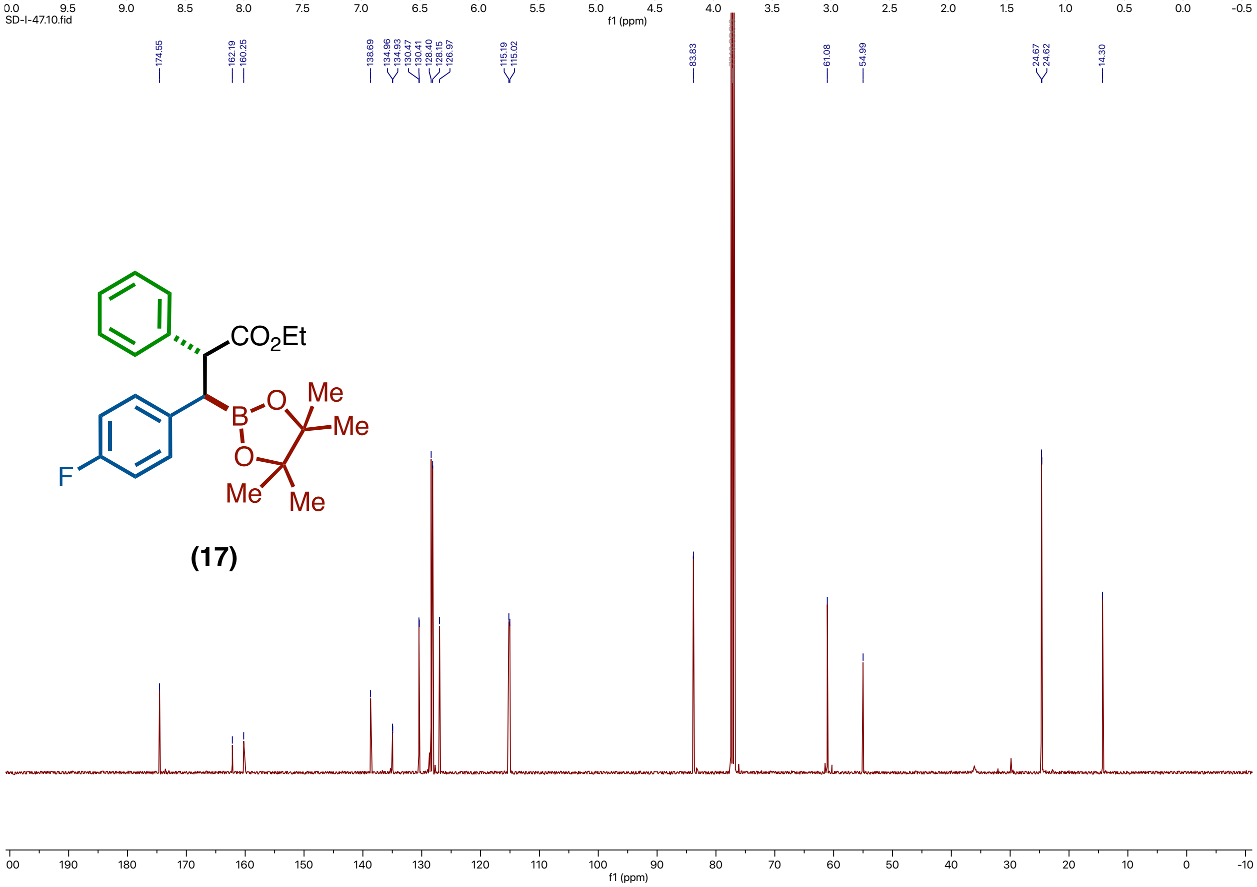

SD-I-47.10.fid
174.55
162.19 160.25
138.69 134.96 134.93 130.47 130.41 128.40 128.15 126.97
115.19 115.02
83.83
61.08 54.99
24.67 24.62
14.30
CO2Et
B
O
O
Me
Me
Me
Me
F
(17)
f1 (ppm)

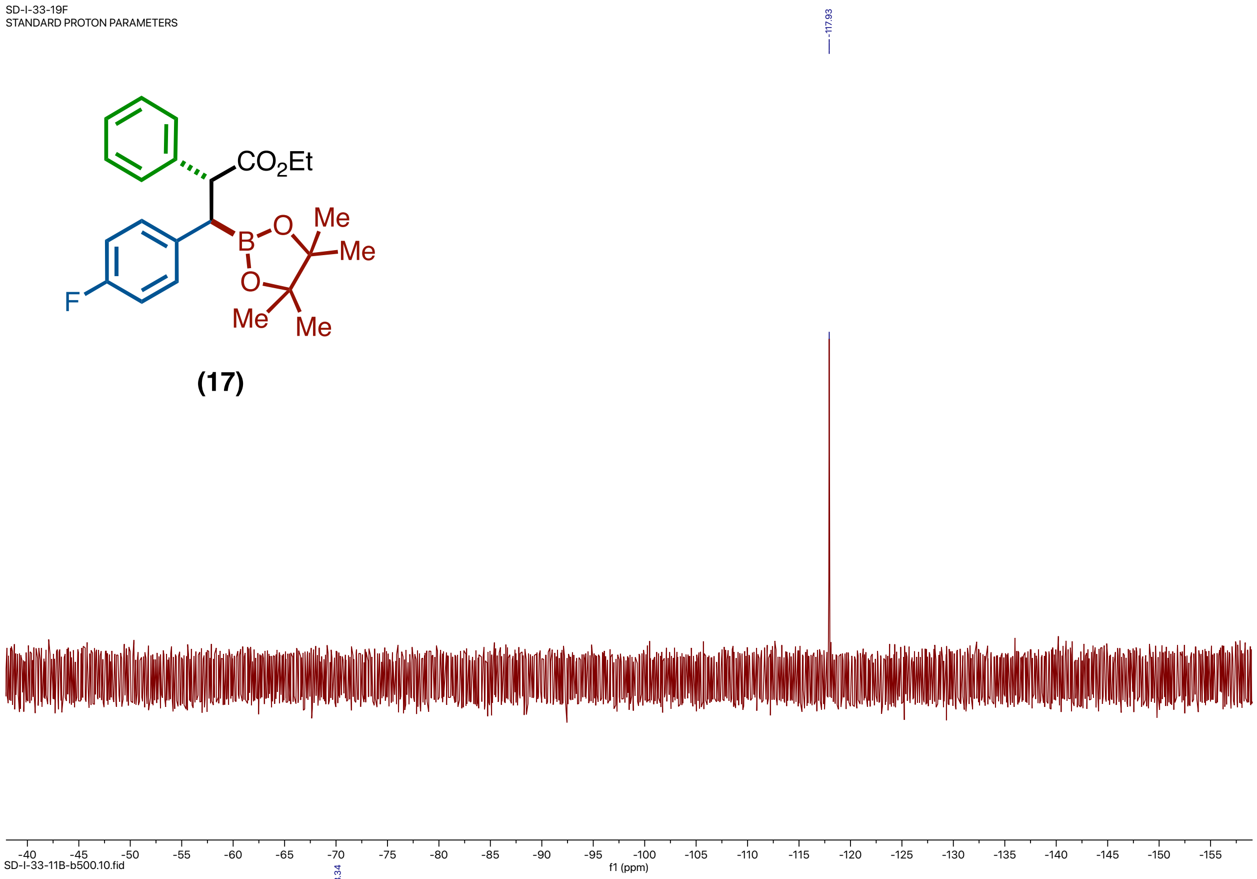
SD-I-33-19F
STANDARD PROTON PARAMETERS
-117.93
CO2Et
Me
Me
Me
Me
B
O
O
F
(17)
-40 -45 -50 -55 -60 -65 -70 -75 -80 -85 -90 -95 -100 -105 -110 -115 -120 -125 -130 -135 -140 -145 -150 -155
f1 (ppm)

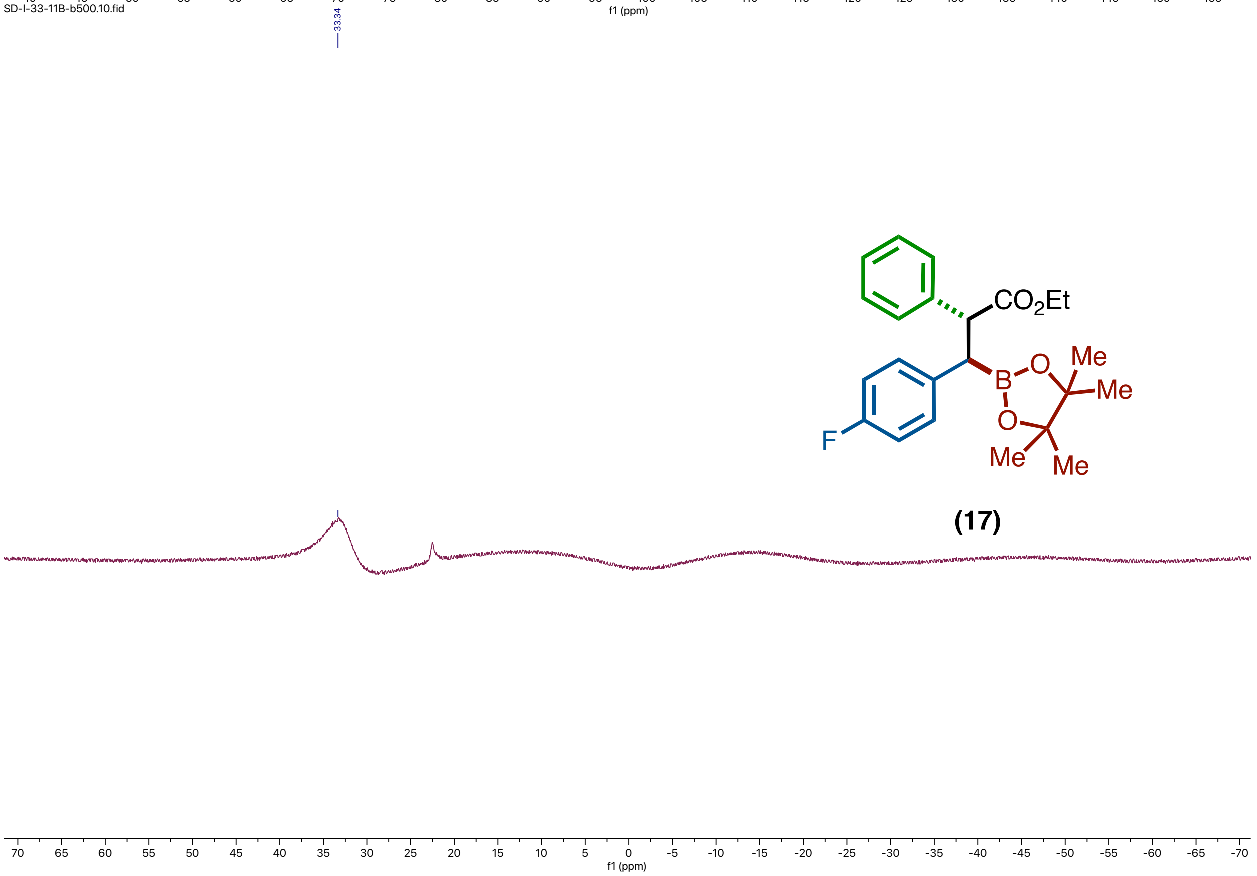
SD-I-33-11B-b500.10.fid
33.34
CO2Et
Me
Me
Me
Me
B
O
O
F
(17)
70 65 60 55 50 45 40 35 30 25 20 15 10 5 0 -5 -10 -15 -20 -25 -30 -35 -40 -45 -50 -55 -60 -65 -70
f1 (ppm)

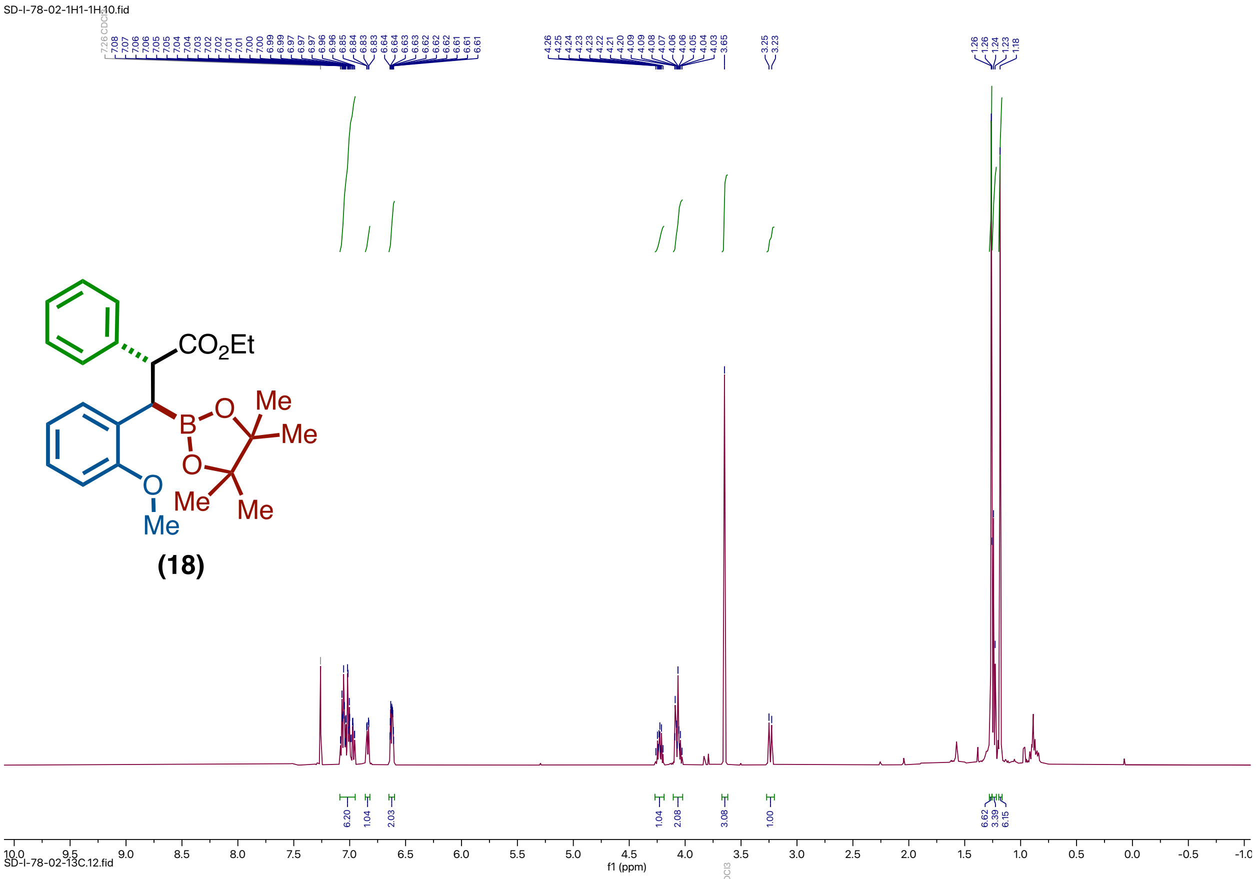

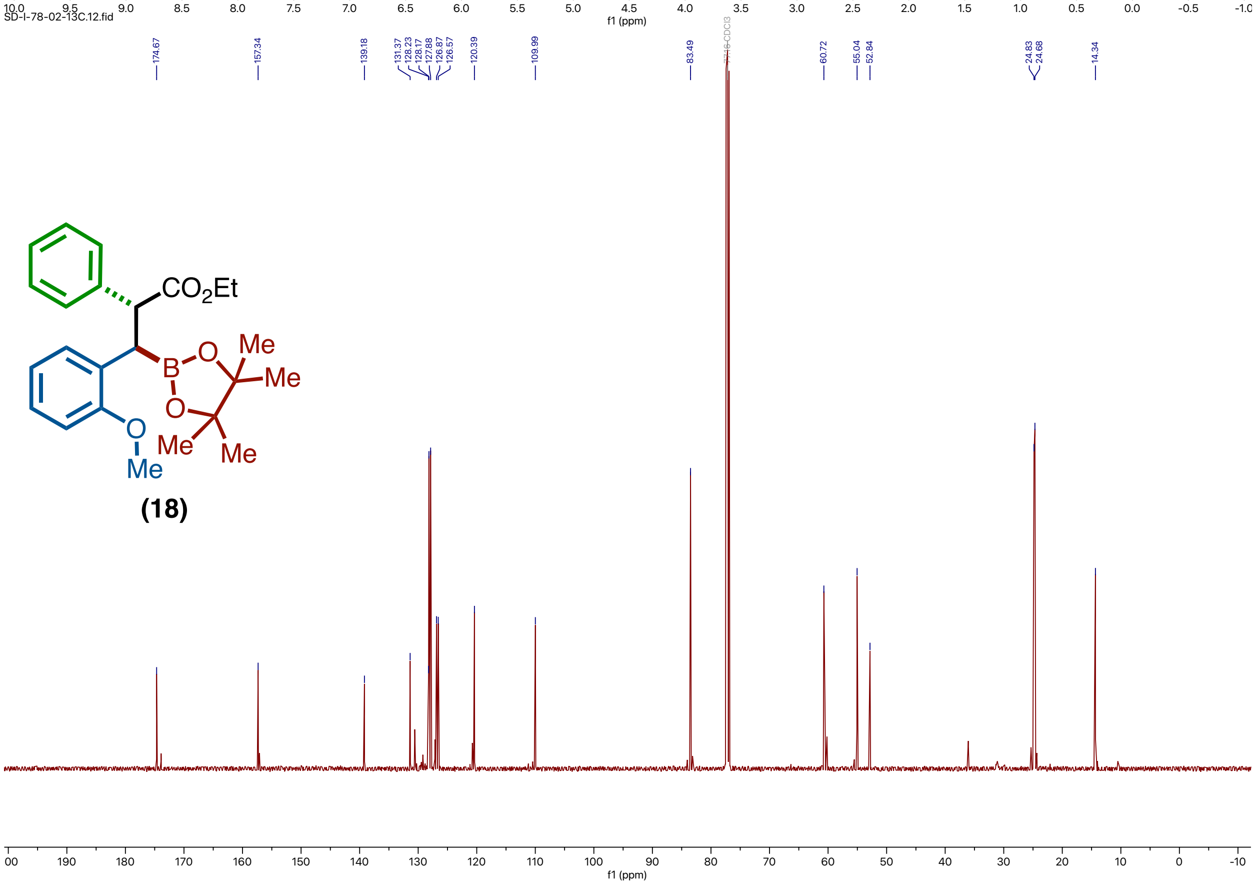

SD-I-78-11B-b500.10.fid

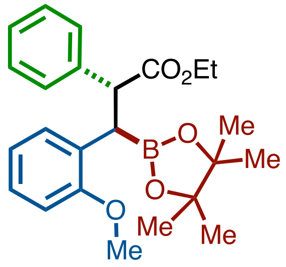

(18)

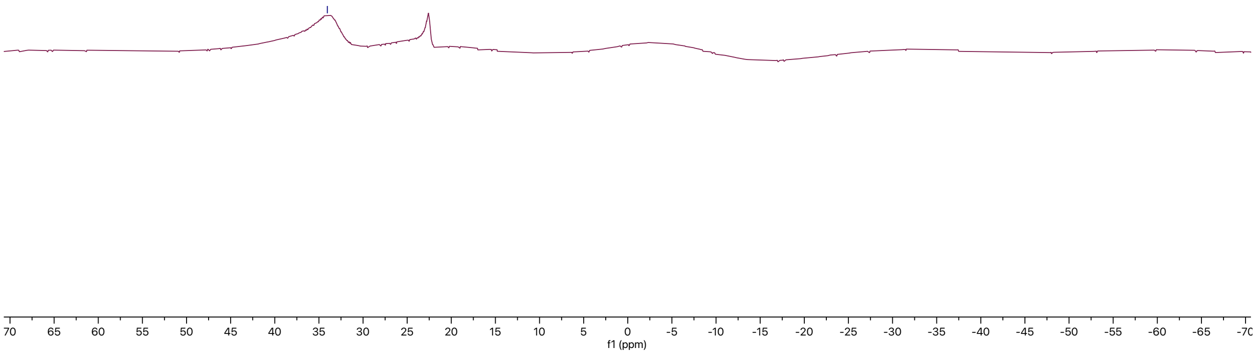

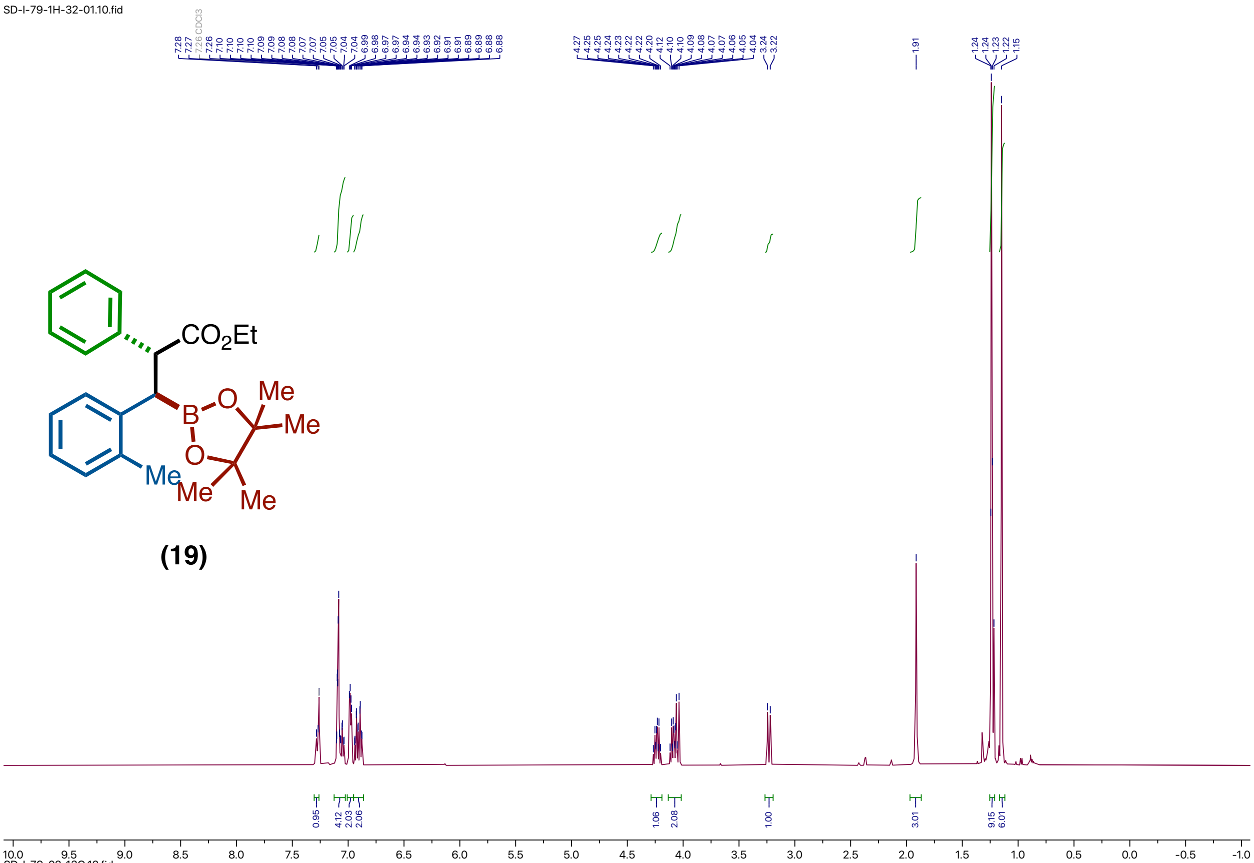

SD-I-79-1H-32-01.10.fid
7.28 7.27 7.26 CDCl3 7.26 7.10 7.10 7.10 7.10 7.09 7.09 7.08 7.08 7.08 7.07 7.07 7.05 7.05 7.04 7.04 6.99 6.98 6.97 6.97 6.94 6.94 6.93 6.93 6.92 6.91 6.91 6.89 6.89 6.88 6.88
4.27 4.25 4.25 4.24 4.23 4.22 4.22 4.20 4.12 4.10 4.10 4.09 4.08 4.07 4.07 4.06 4.05 4.04 3.24 3.22
1.91
1.24 1.24 1.23 1.22 1.15
CO2Et
B
O
Me
(19)
0.95 4.12 2.03 2.06 1.06 2.08 1.00 3.01 9.15 6.01
f1 (ppm)

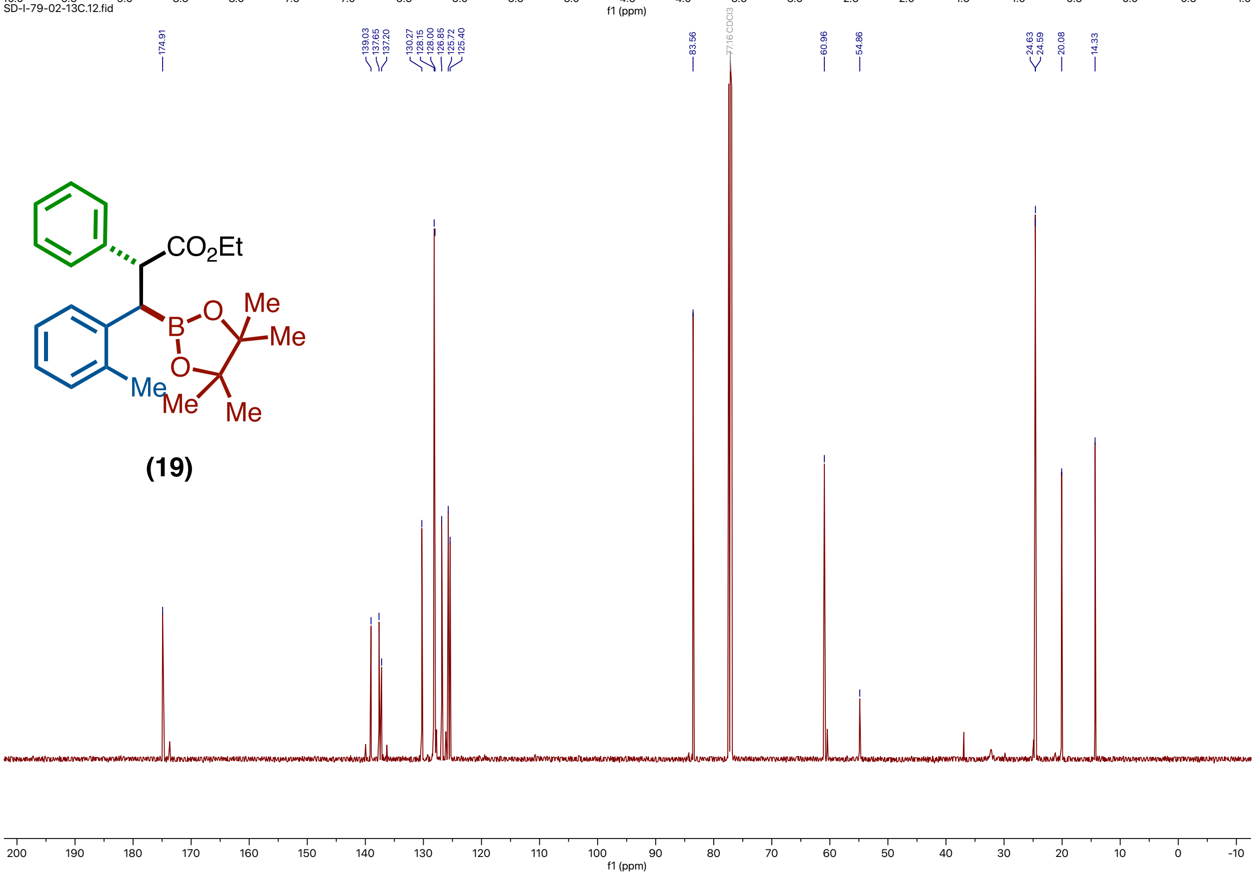

SD-I-79-02-13C.12.fid
174.91
139.03 137.65 137.20 130.27 128.15 128.00 126.85 125.72 125.40
83.56
77.16 CDCl3
60.96
54.86
24.63 24.59
20.08
14.33
CO2Et
B
O
Me
(19)
f1 (ppm)

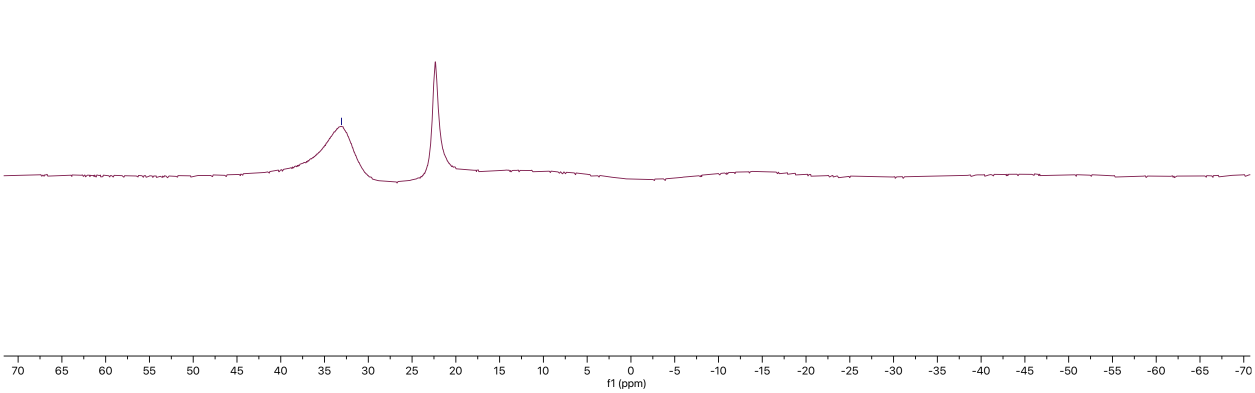
SD-I-95-11B-b500.10.fid
33.06
70
65
60
55
50
45
40
35
30
25
20
15
10
5
0
-5
-10
-15
-20
-25
-30
-35
-40
-45
-50
-55
-60
-65
-70
f1 (ppm)

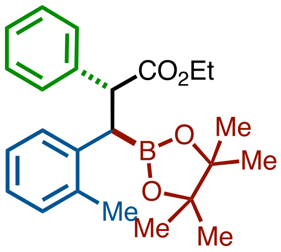
CO2Et
B
O
O
Me
Me
Me
Me
Me

(19)

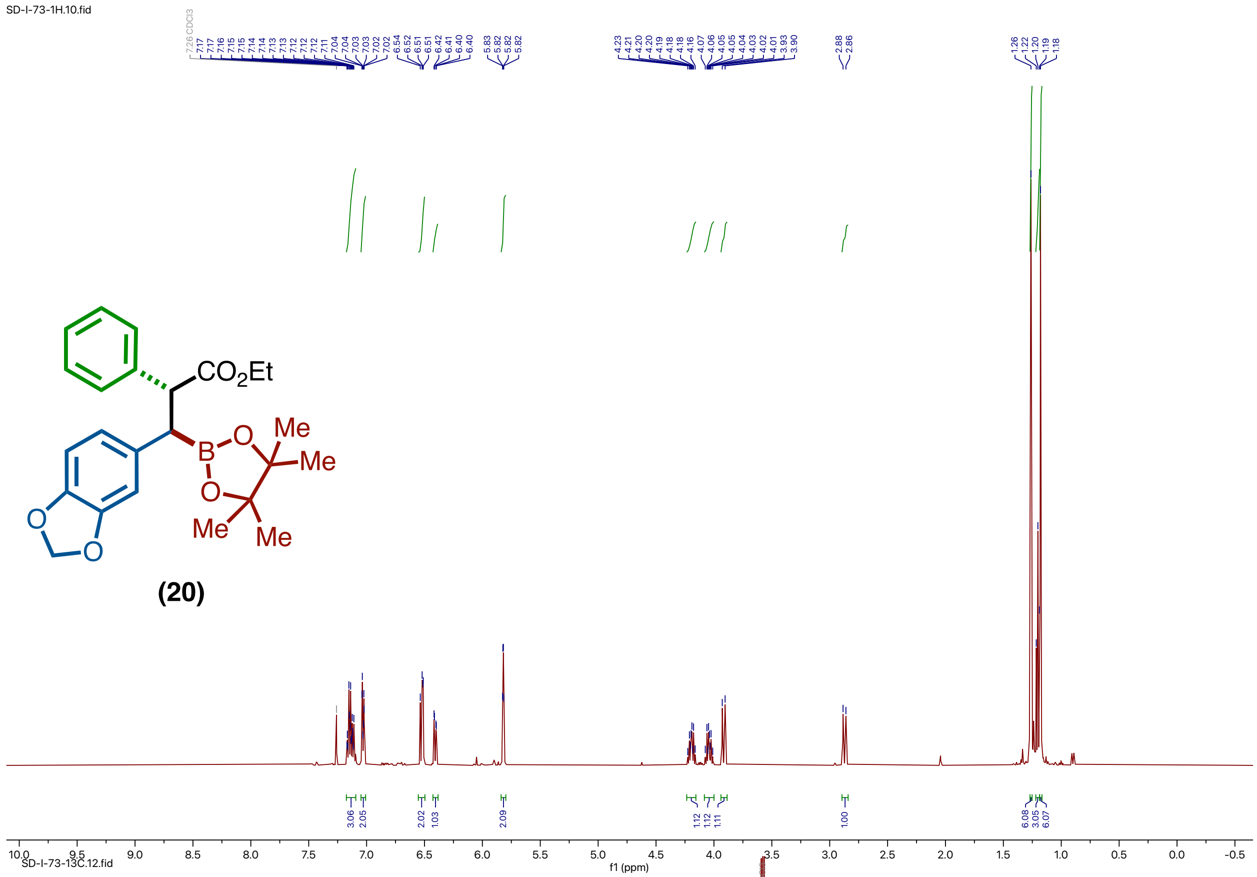
SD-I-73-1H.10.fid
7.26 CDCl3
7.17 7.16 7.15 7.15 7.14 7.14 7.13 7.13 7.12 7.12 7.12 7.11 7.04 7.04 7.03 7.03 7.02 7.02 6.54 6.52 6.51 6.51 6.42 6.41 6.40 6.40 5.83 5.82 5.82 5.82 5.82
4.23 4.21 4.20 4.20 4.19 4.18 4.18 4.16 4.07 4.06 4.05 4.05 4.04 4.03 4.02 4.01 3.93 3.90
2.88 2.86
1.26 1.22 1.20 1.19 1.18
CO2Et
B
O
O
Me
Me
Me
Me
O
O
(20)
3.06 2.05 2.02 1.03 2.09 1.12 1.12 1.11 1.00 6.08 3.05 6.07
10.0 9.5 9.0 8.5 8.0 7.5 7.0 6.5 6.0 5.5 5.0 4.5 4.0 3.5 3.0 2.5 2.0 1.5 1.0 0.5 0.0 -0.5
f1 (ppm)

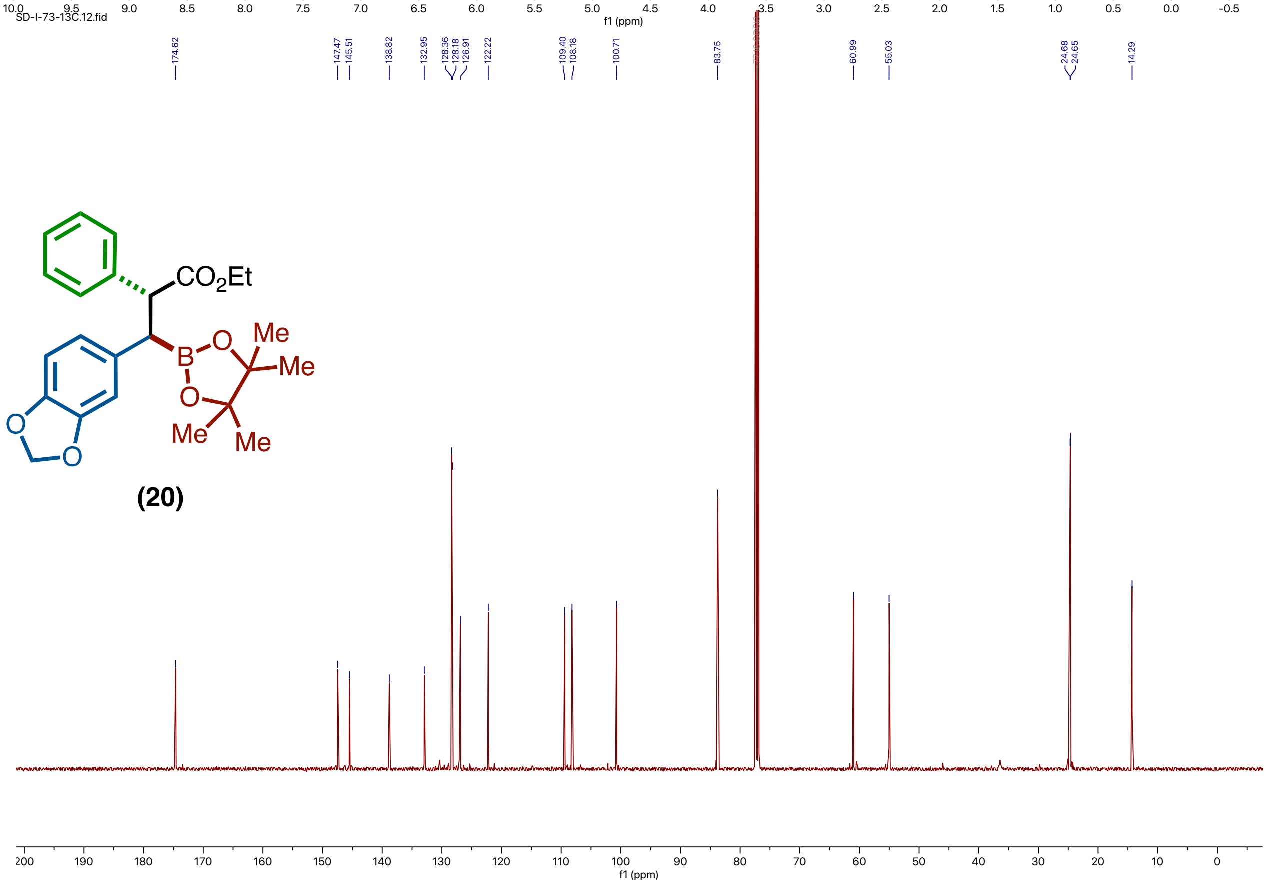
SD-I-73-13C.12.fid
174.62
147.47 145.51 138.82 132.95 128.36 128.18 126.91 122.22
109.40 108.18 100.71 83.75
60.99 55.03 24.68 24.65 14.29
CO2Et
B
O
O
Me
Me
Me
Me
O
O
(20)
200 190 180 170 160 150 140 130 120 110 100 90 80 70 60 50 40 30 20 10 0
f1 (ppm)

SD-I-73-11B-New.10.fid

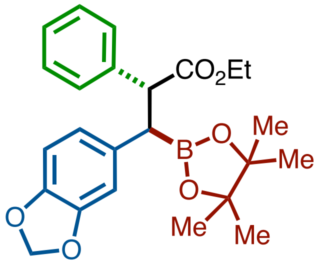

(20)

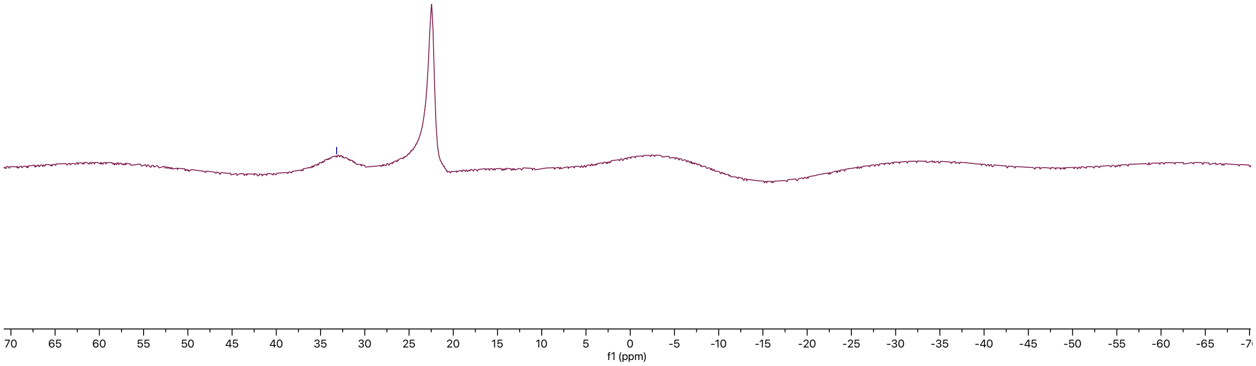

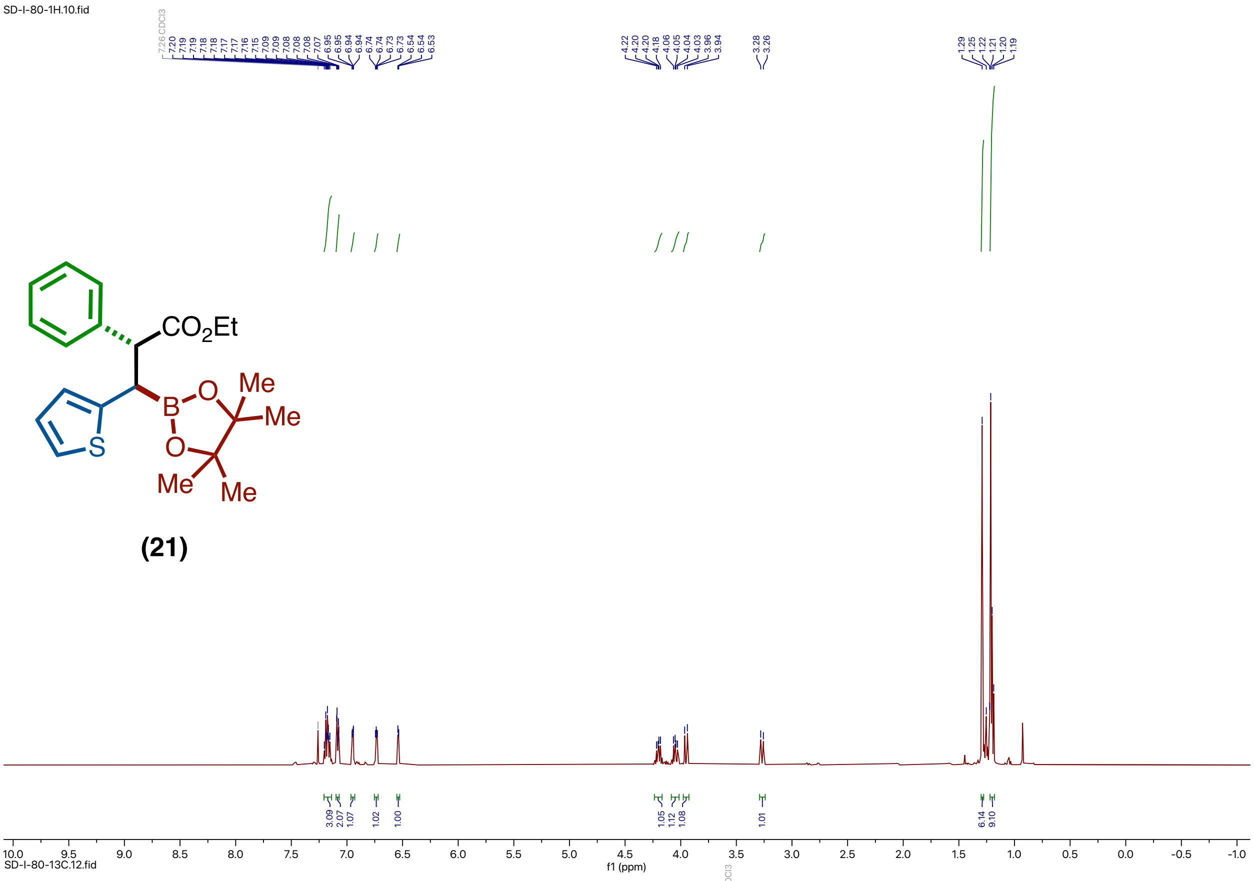
SD-I-80-1H.10.fid
CO2Et
B
O
O
Me
Me
Me
Me
S
(21)
f1 (ppm)

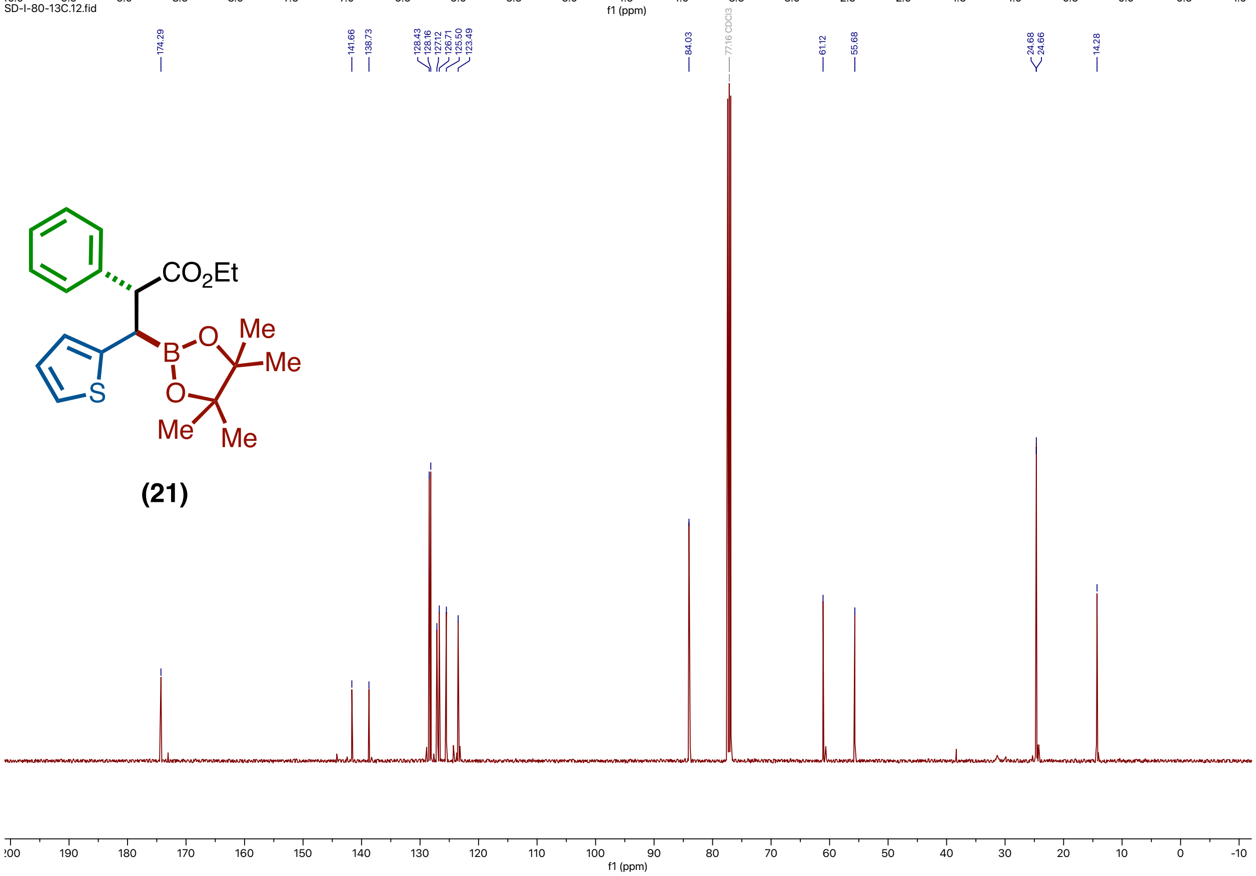
SD-I-80-13C.12.fid
174.29
141.66
138.73
128.43
128.16
127.12
126.71
125.50
123.49
84.03
77.16 CDCl3
61.12
55.68
24.68
24.66
14.28
CO2Et
B
O
O
Me
Me
Me
Me
S
(21)
f1 (ppm)

SD-I-80-11B-New.10.fid

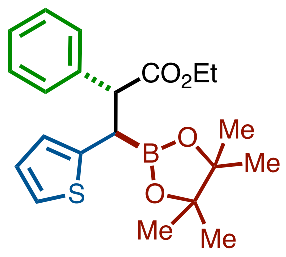

(21)

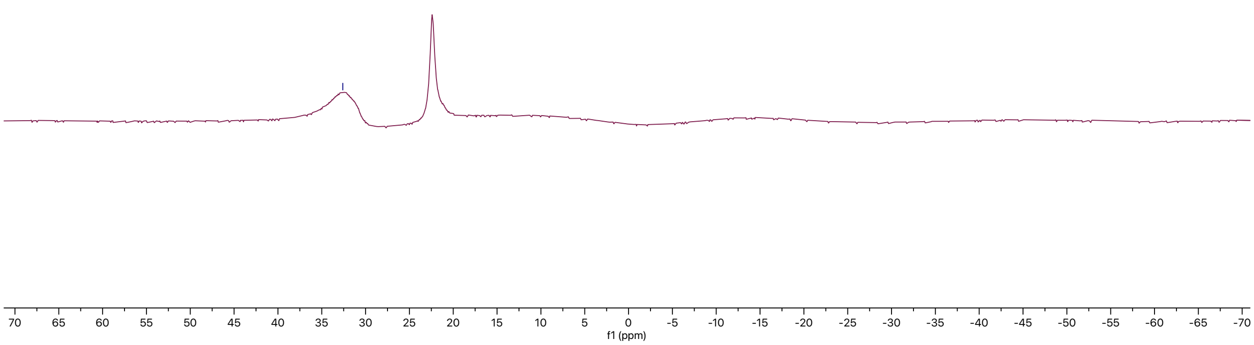

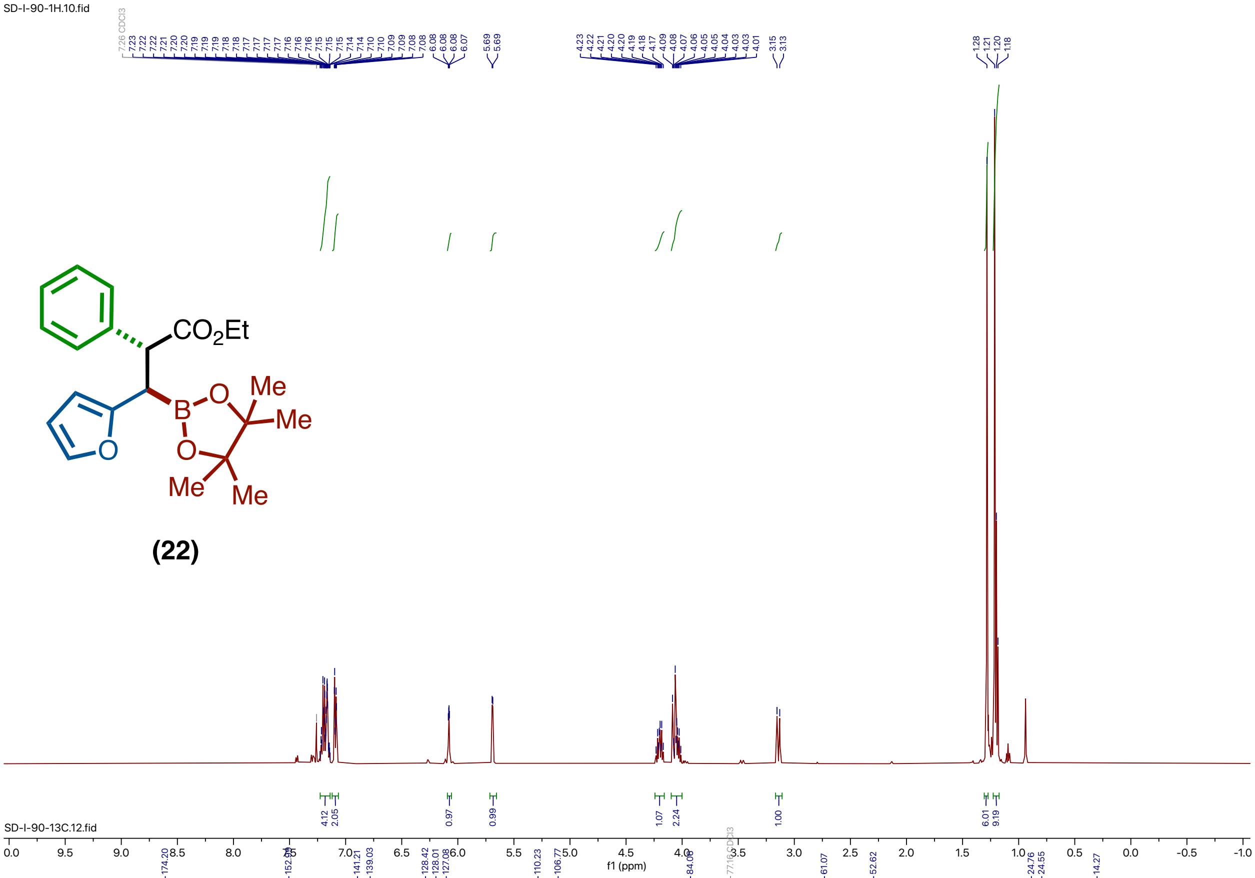
SD-I-90-1H.10.fid
CO2Et
Me
Me
Me
Me
(22)
f1 (ppm)

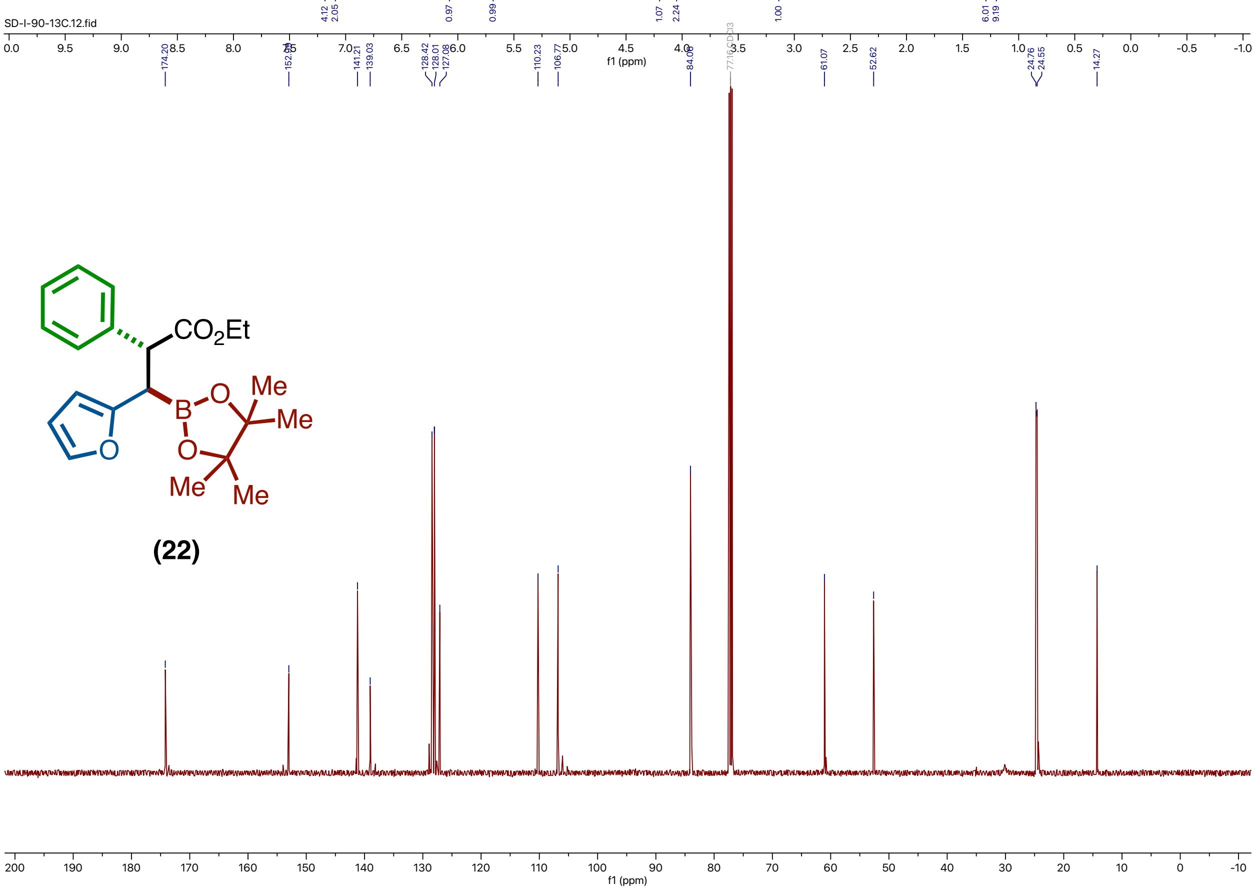
SD-I-90-13C.12.fid
CO2Et
Me
Me
Me
Me
(22)
f1 (ppm)

SD-I-81-11B
STANDARD BORON PARAMETERS

32.07

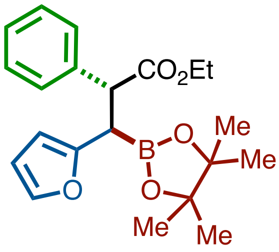

(22)

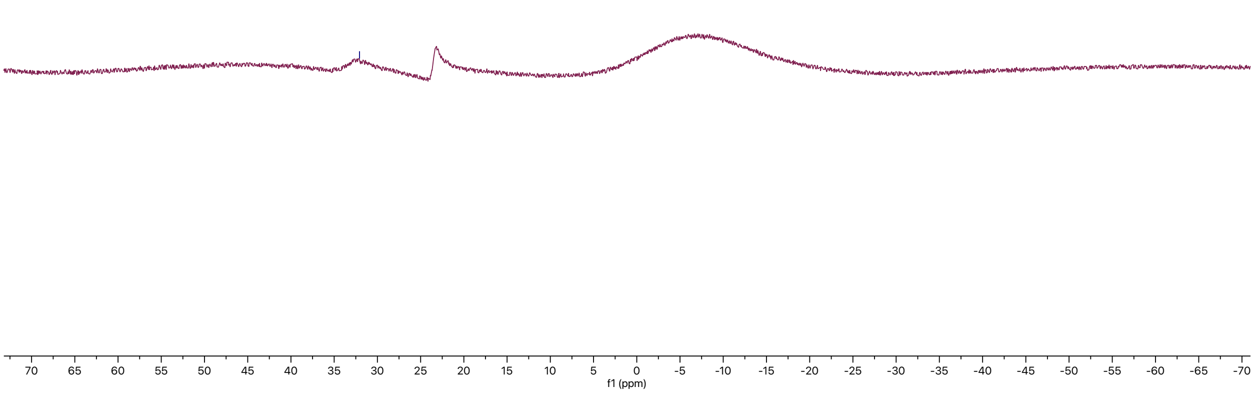

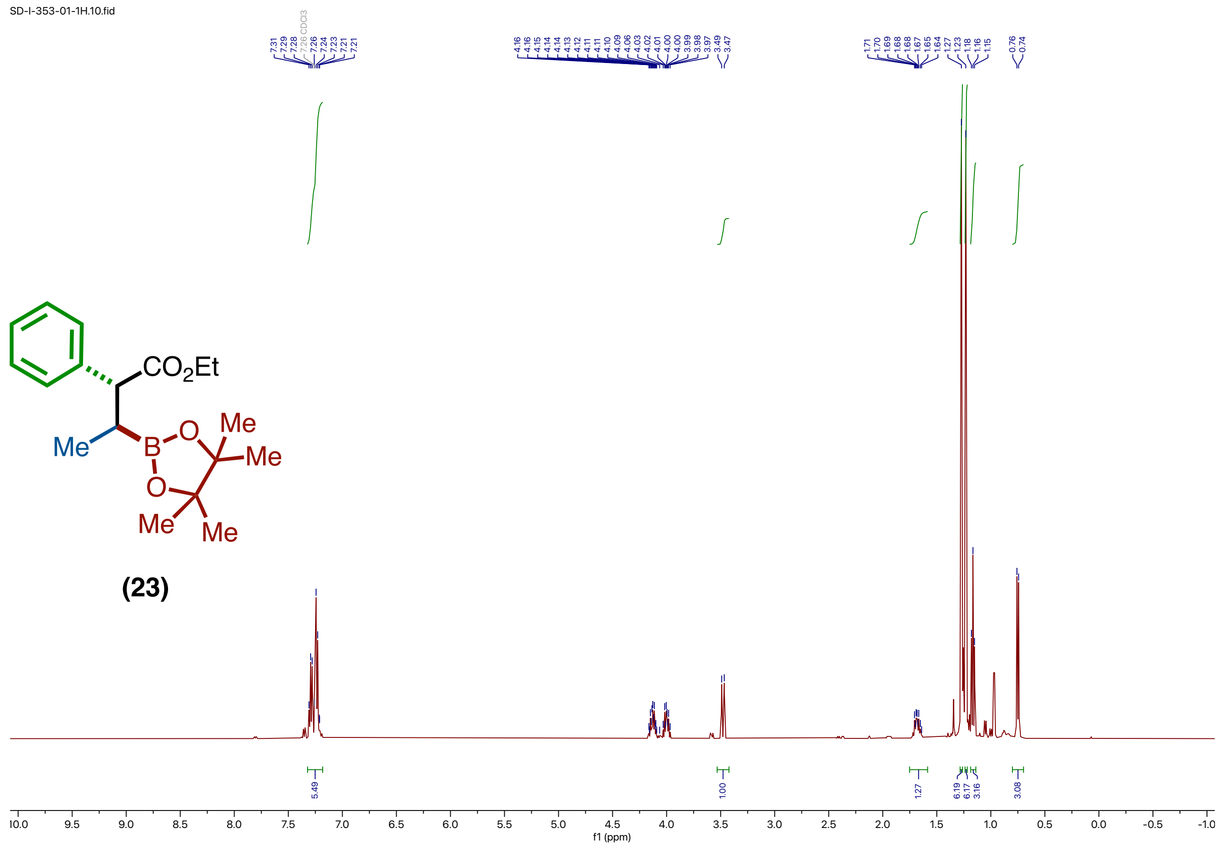
SD-I-353-01-1H.10.fid
CO2Et
Me
B
O
Me
(23)
5.49
1.00
1.27
6.19
6.17
3.16
3.08
f1 (ppm)

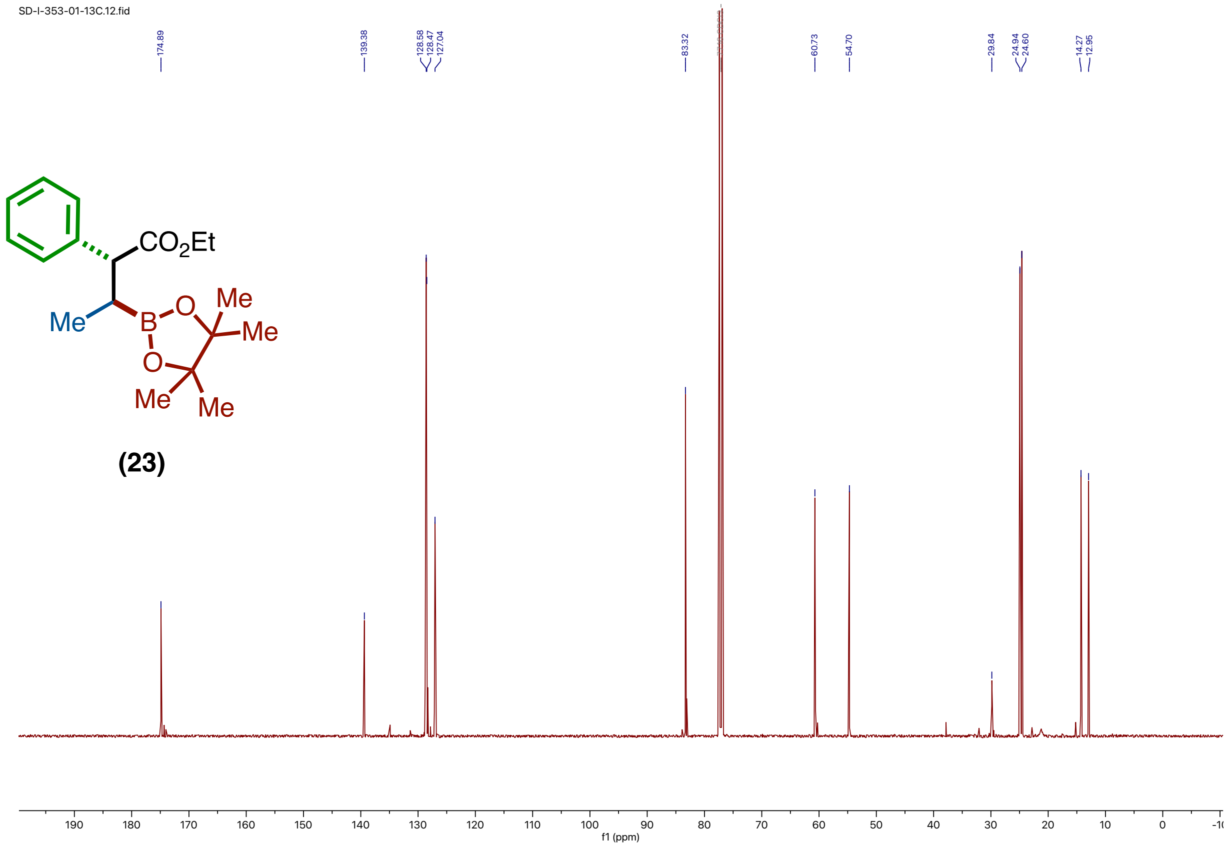
SD-I-353-01-13C.12.fid
174.89
139.38
128.58
128.47
127.04
83.32
60.73
54.70
29.84
24.94
24.60
14.27
12.95
CO2Et
Me
B
O
Me
(23)
f1 (ppm)

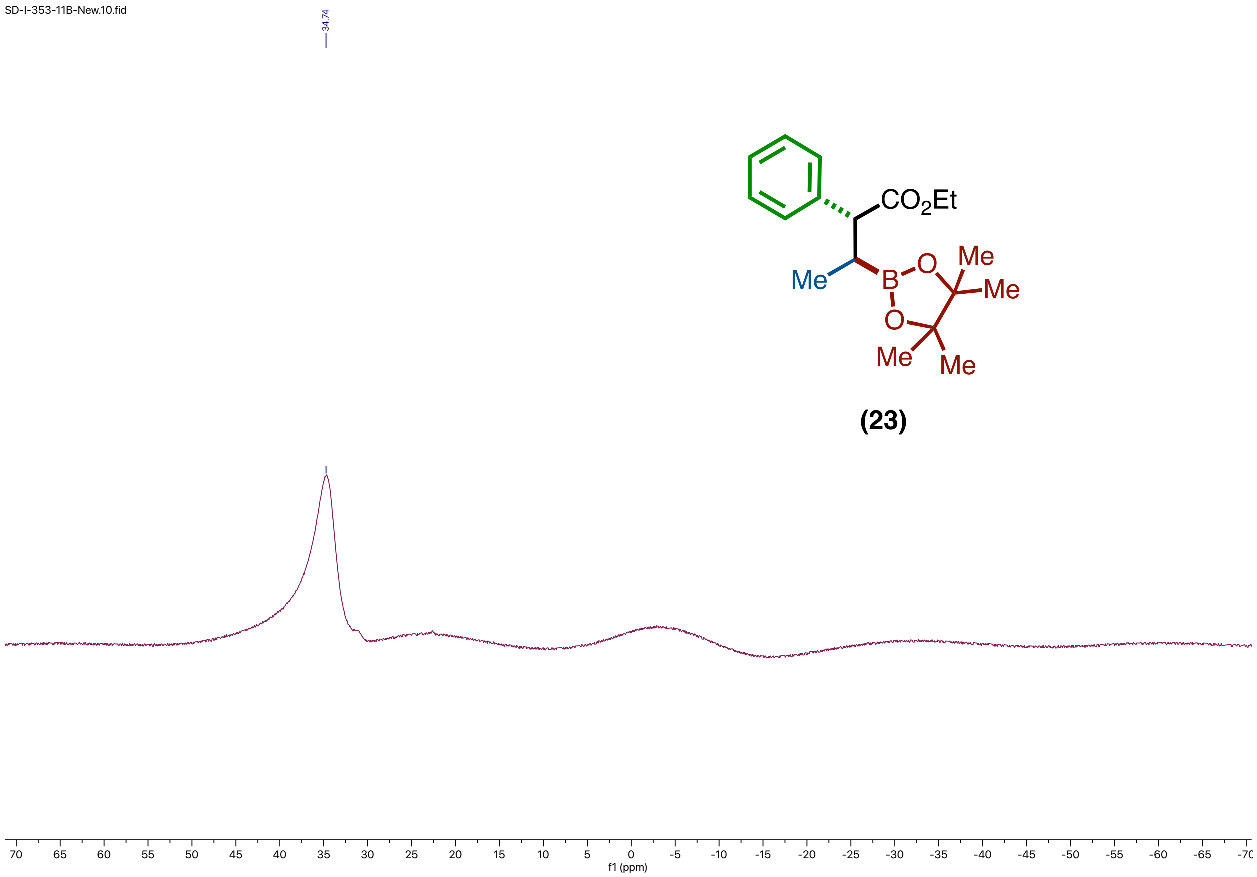
SD-I-353-11B-New.10.fid
34.74
CO2Et
Me
B
O
Me
Me
Me
Me
70 65 60 55 50 45 40 35 30 25 20 15 10 5 0 -5 -10 -15 -20 -25 -30 -35 -40 -45 -50 -55 -60 -65 -70
f1 (ppm)

(23)

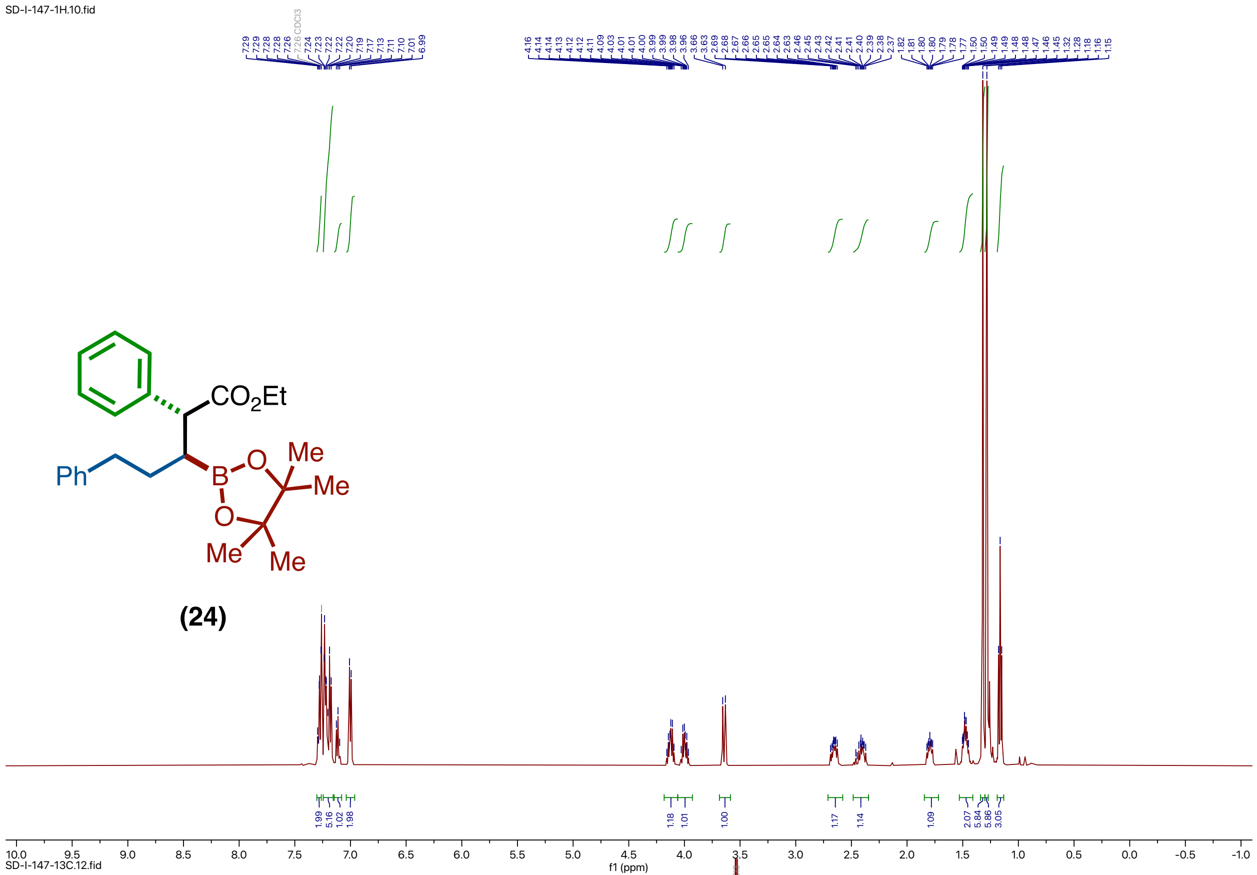
SD-I-147-1H.10.fid
CO2Et
Ph
B
O
Me
(24)
f1 (ppm)

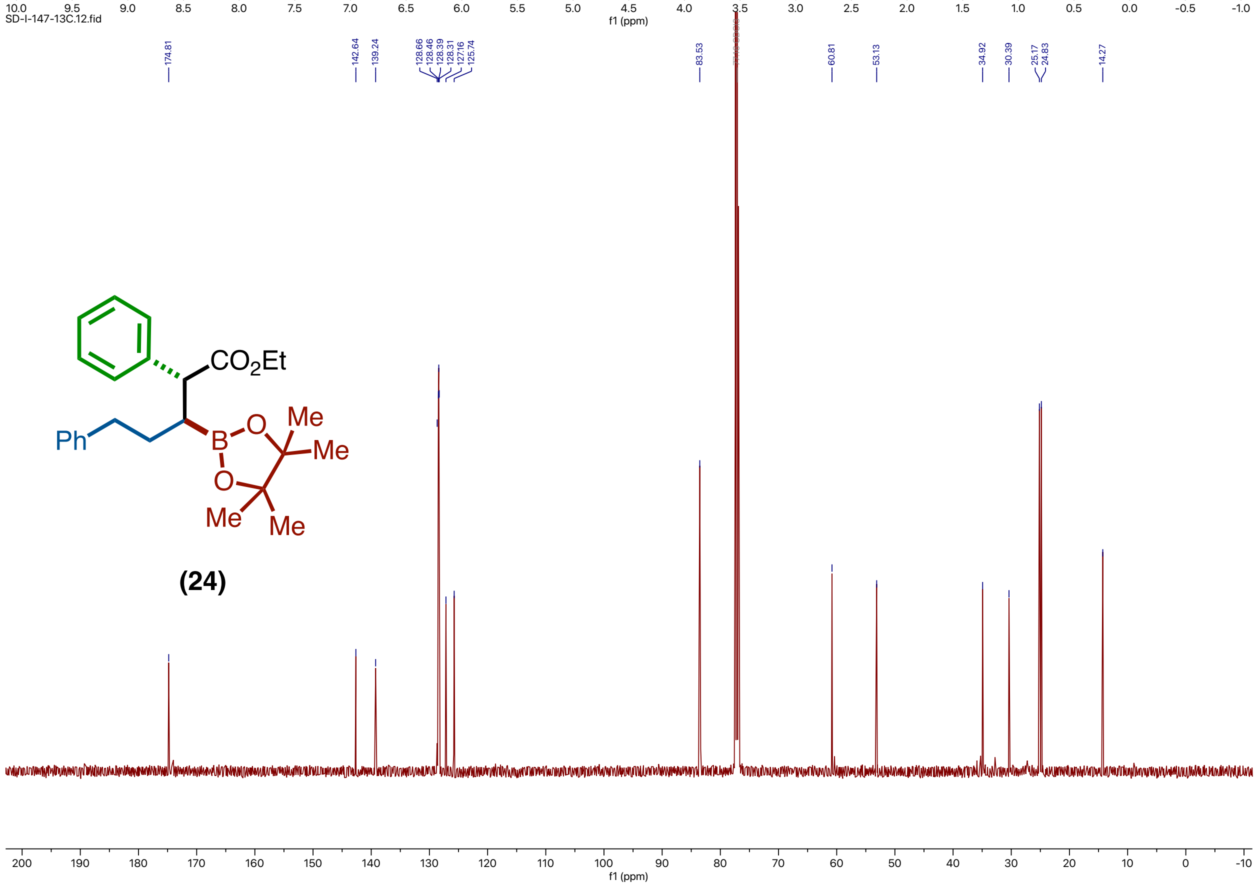
SD-I-147-13C.12.fid
174.81
142.64
139.24
128.66
128.46
128.39
128.31
127.16
125.74
83.53
60.81
53.13
34.92
30.39
25.17
24.83
14.27
CO2Et
Ph
B
O
Me
(24)
f1 (ppm)

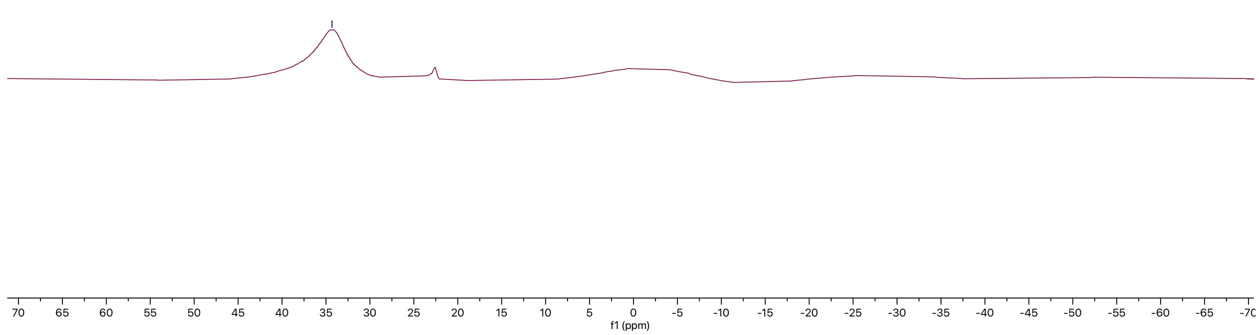
SD-I-125-11B-b500.10.fid
34.32
70 65 60 55 50 45 40 35 30 25 20 15 10 5 0 -5 -10 -15 -20 -25 -30 -35 -40 -45 -50 -55 -60 -65 -70
f1 (ppm)

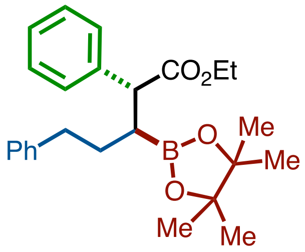
CO2Et
Ph
B
O
O
Me
Me
Me
Me

(24)

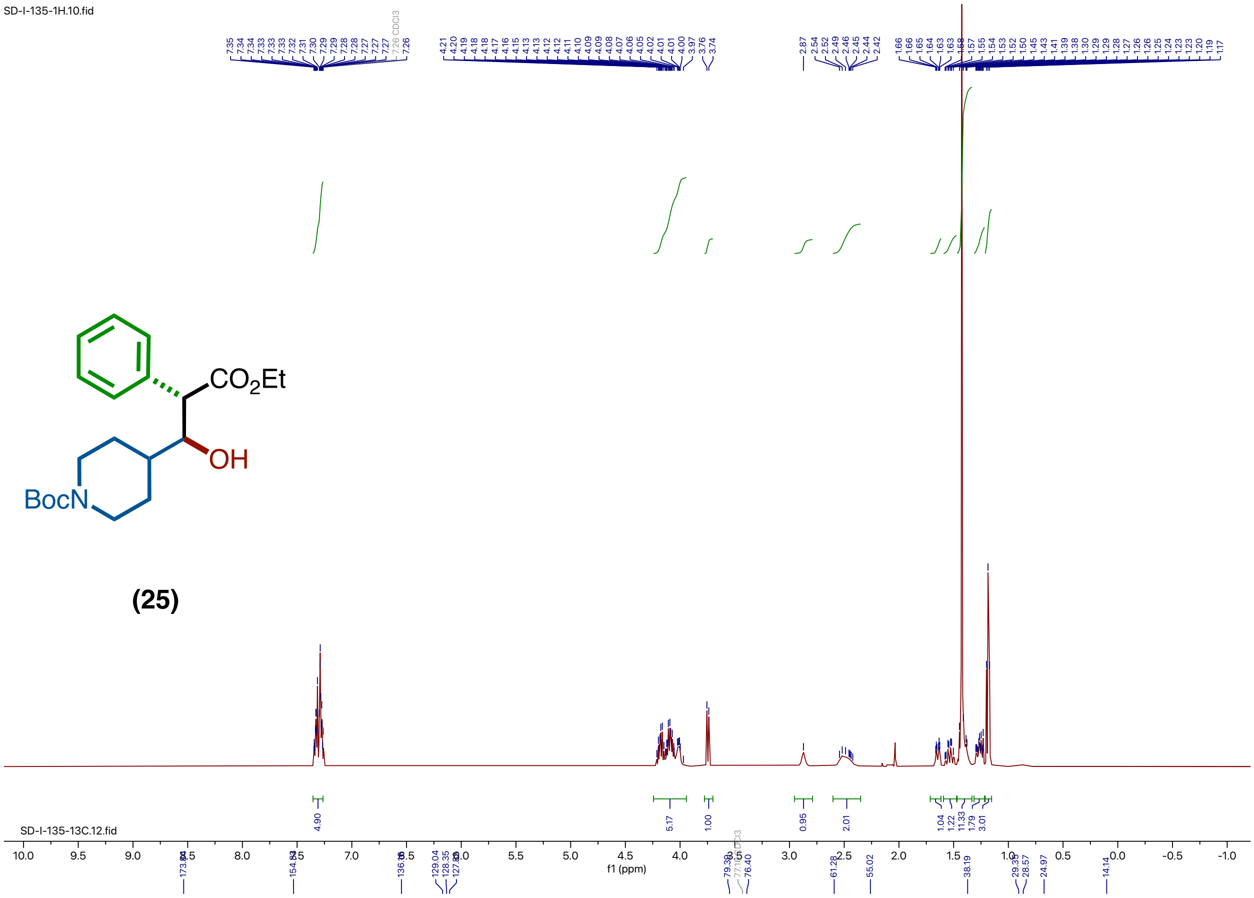
SD-I-135-1H.10.fid
CO2Et
OH
BocN
f1 (ppm)

(25)

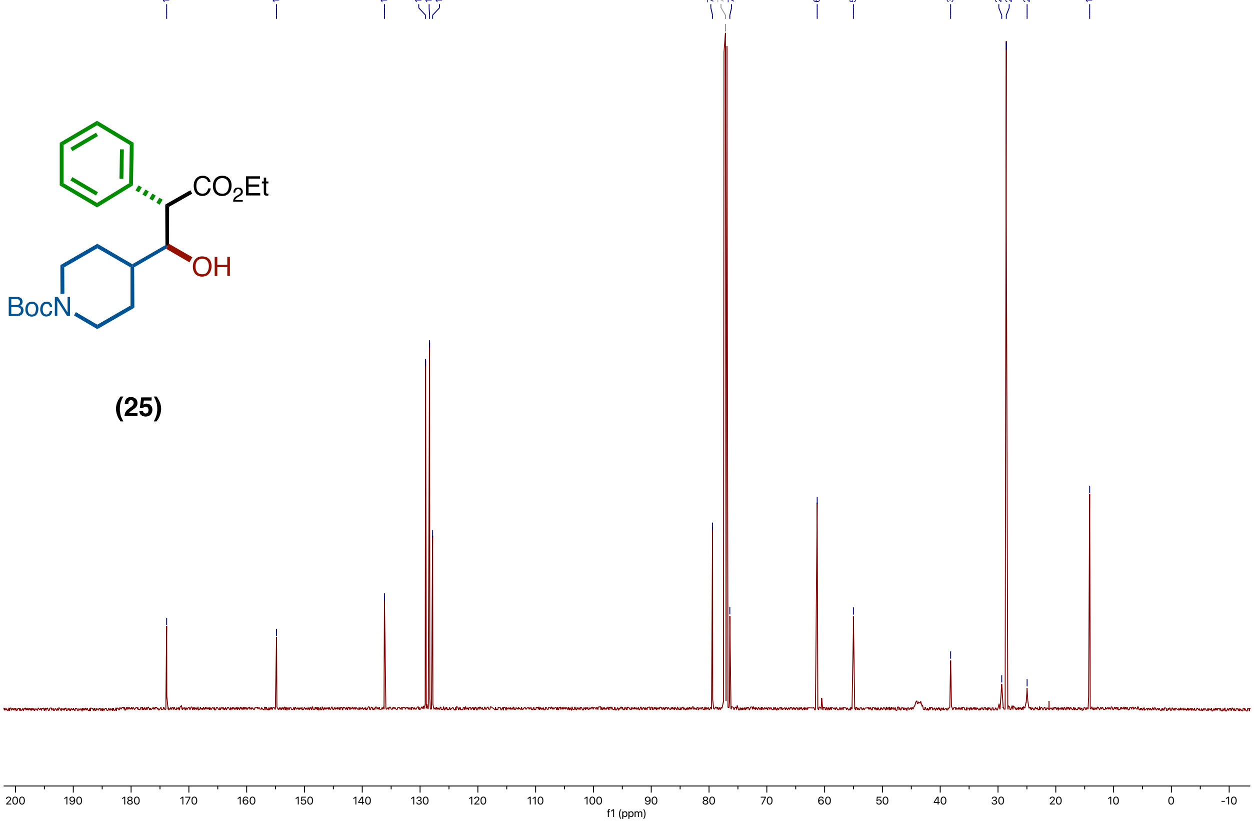
SD-I-135-13C.12.fid
CO2Et
OH
BocN
f1 (ppm)

(25)

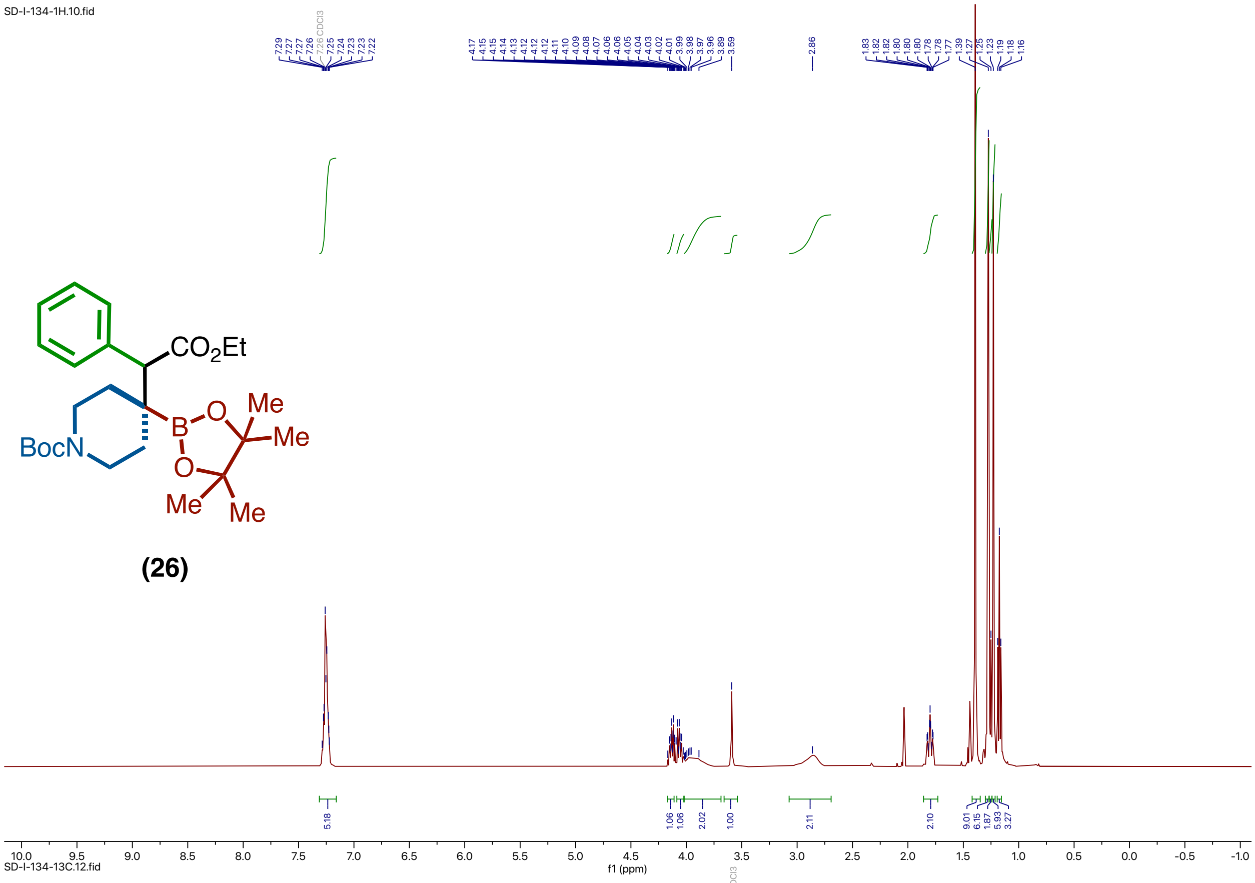
SD-I-134-1H.10.fid
7.29
7.27
7.27
7.26
7.26 CDCl3
7.25
7.24
7.23
7.23
7.22
4.17
4.15
4.15
4.14
4.13
4.12
4.12
4.12
4.11
4.10
4.09
4.08
4.07
4.06
4.06
4.06
4.05
4.04
4.03
4.02
4.01
3.99
3.98
3.97
3.96
3.89
3.59
2.86
1.83
1.82
1.82
1.80
1.80
1.80
1.78
1.78
1.77
1.39
1.27
1.25
1.23
1.19
1.18
1.16
CO2Et
BocN
B
O
O
Me
Me
Me
Me
(26)
5.18
1.06
1.06
2.02
1.00
2.11
2.10
9.01
6.15
1.87
5.93
3.27
f1 (ppm)

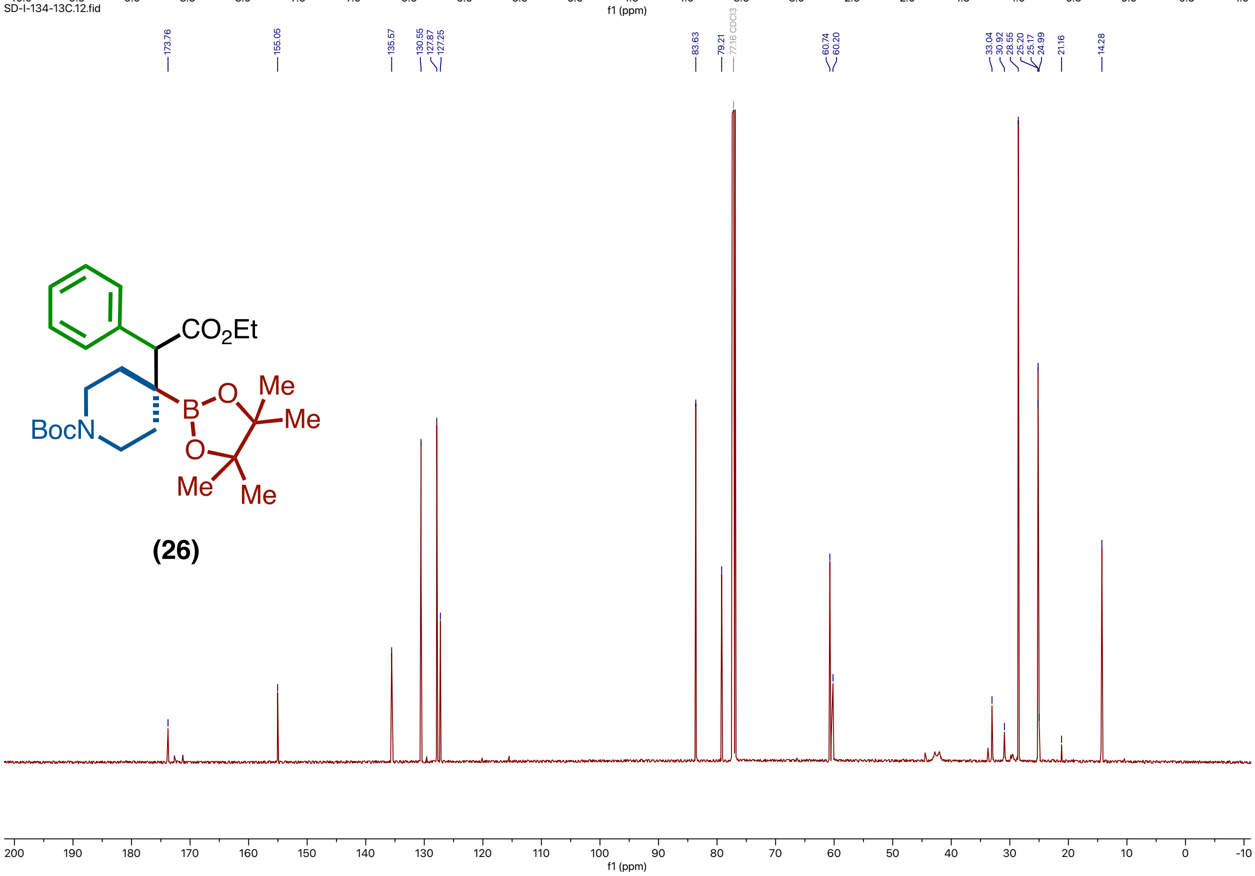
SD-I-134-13C.12.fid
173.76
155.05
135.57
130.55
127.87
127.25
83.63
79.21
77.16 CDCl3
60.74
60.20
33.04
30.92
28.55
25.20
25.17
24.99
21.16
14.28
CO2Et
BocN
B
O
O
Me
Me
Me
Me
(26)
f1 (ppm)

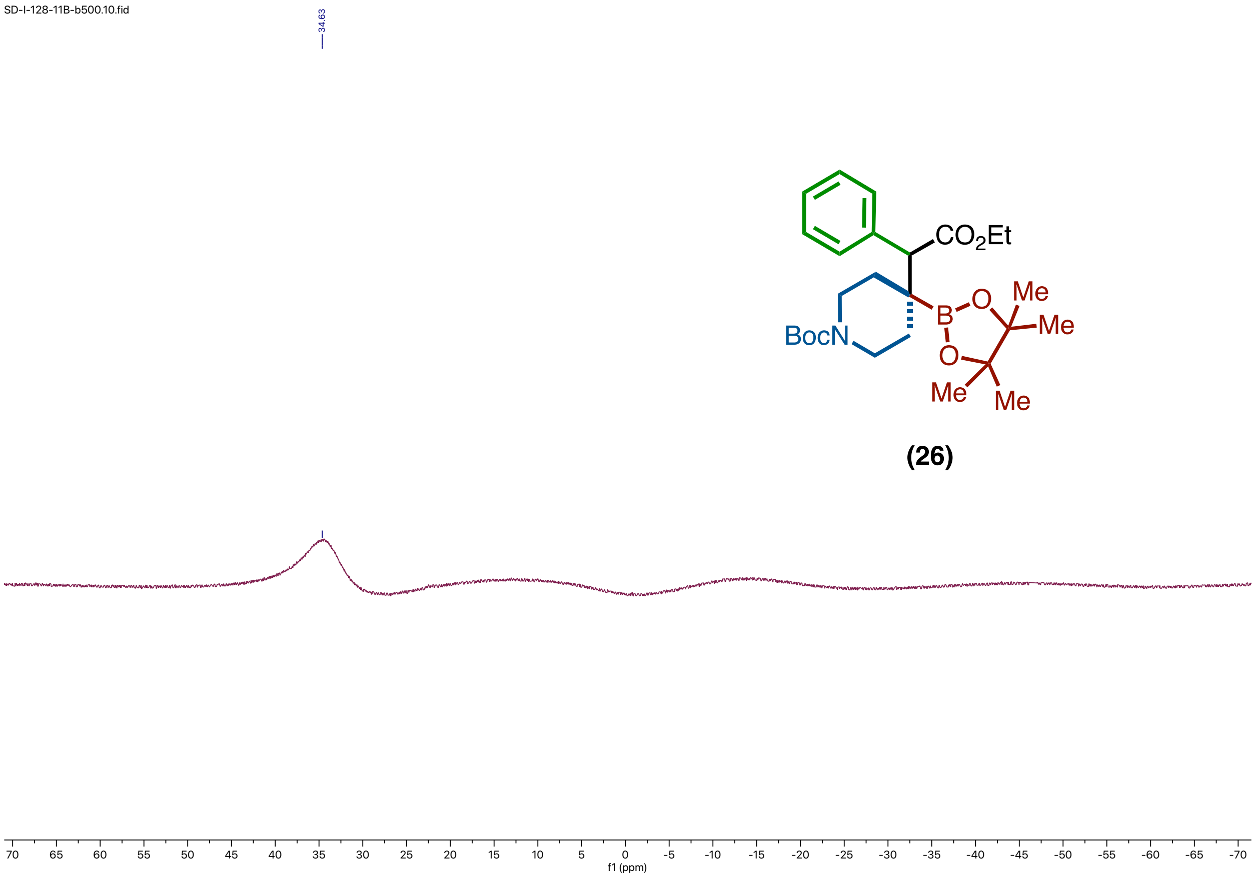
SD-I-128-11B-b500.10.fid
34.63
CO2Et
BocN
B
O
O
Me
Me
Me
Me
(26)
70 65 60 55 50 45 40 35 30 25 20 15 10 5 0 -5 -10 -15 -20 -25 -30 -35 -40 -45 -50 -55 -60 -65 -70
f1 (ppm)

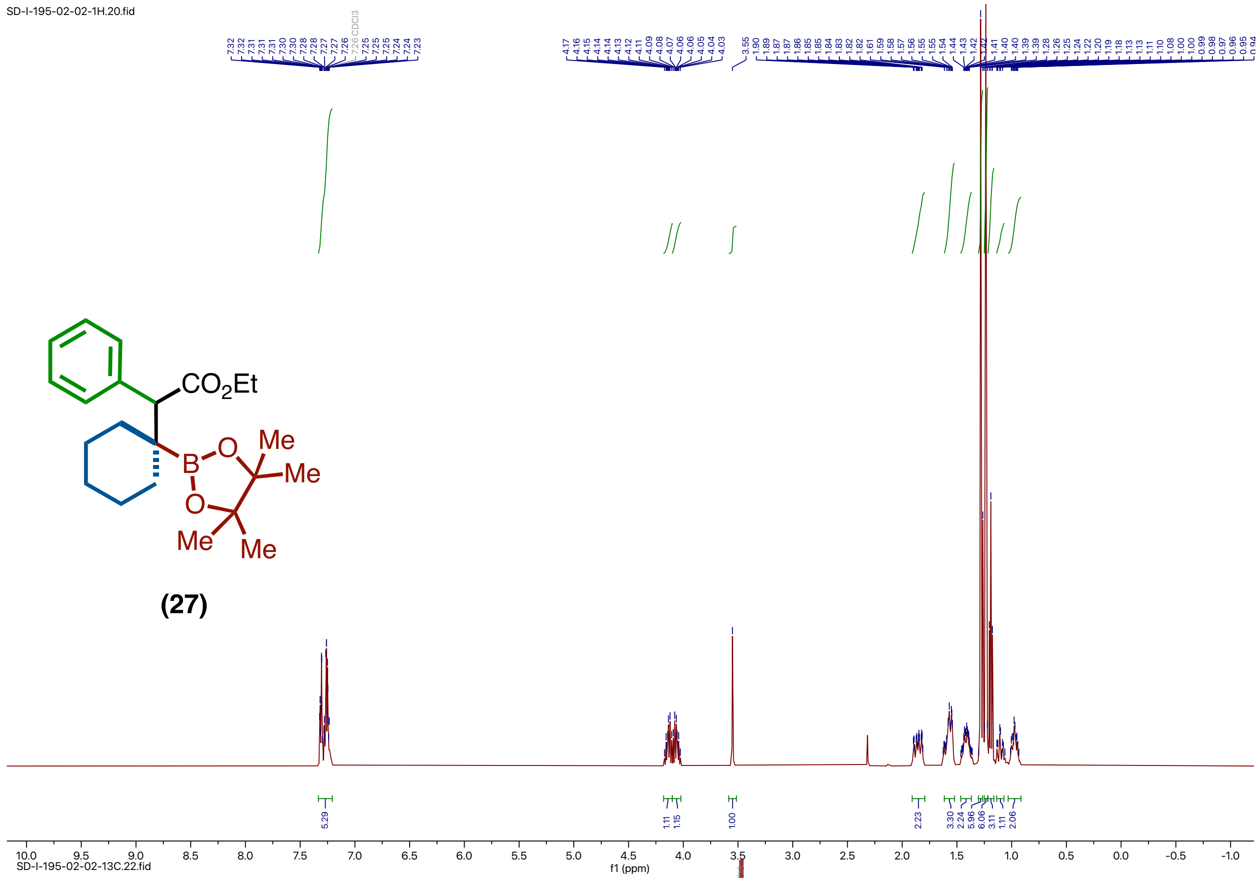
SD-I-195-02-02-1H.20.fid
CO_2Et
B
O
Me
(27)
5.29
1.11
1.15
1.00
2.23
3.30
2.24
5.96
6.06
3.11
1.11
2.06
f1 (ppm)

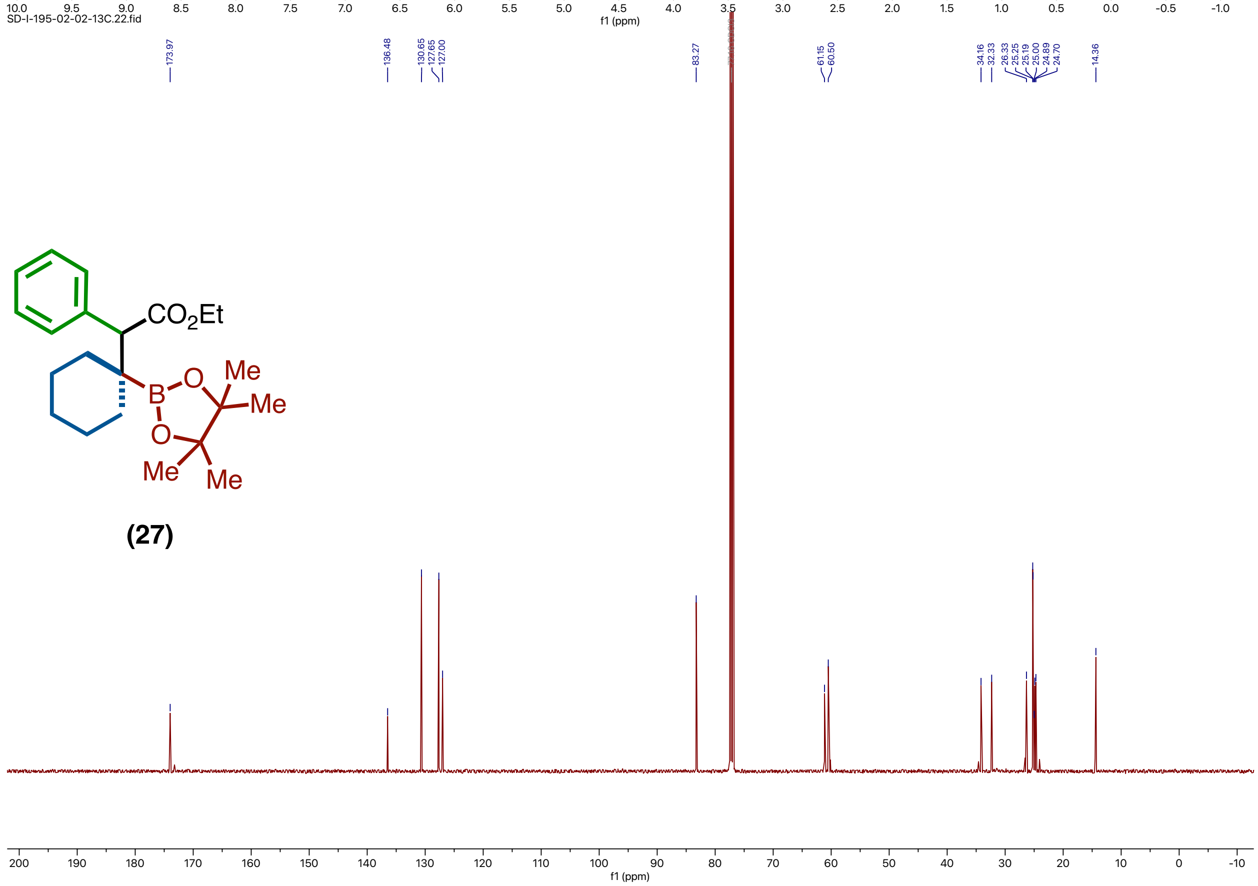
SD-I-195-02-02-13C.22.fid
173.97
136.48
130.65
127.65
127.00
83.27
61.15
60.50
34.16
32.33
26.33
25.25
25.19
25.00
24.89
24.70
14.36
CO_2Et
B
O
Me
(27)
f1 (ppm)

SD-I-195-11B-b500.10.fid

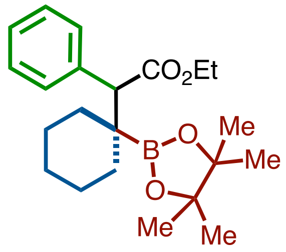

(27)

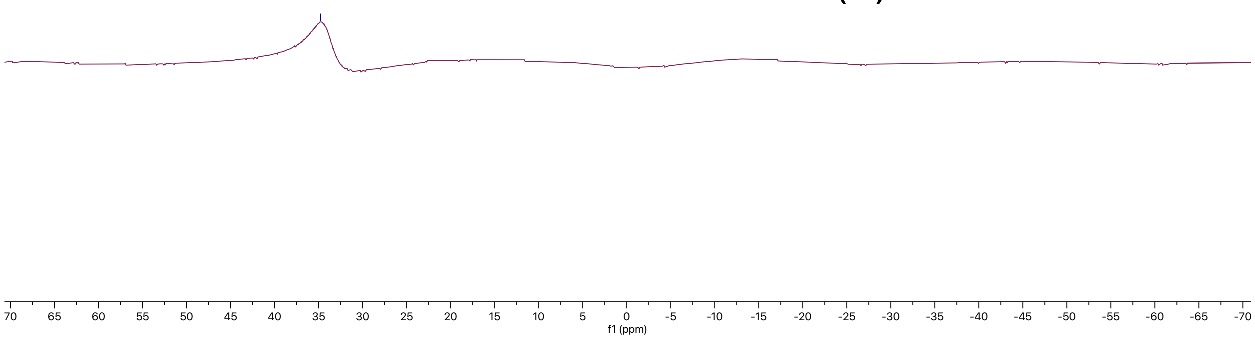

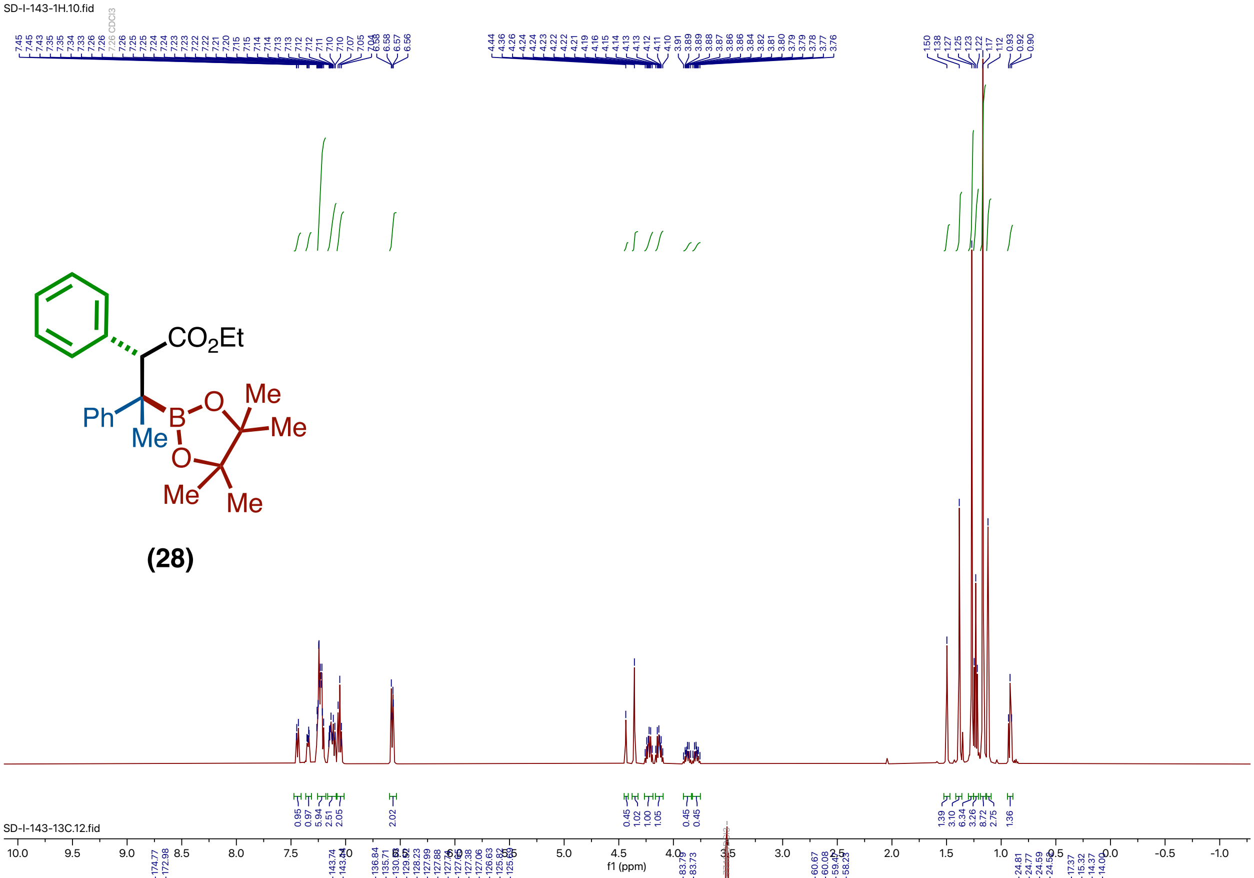
SD-I-143-1H.10.fid
CO2Et
Ph
Me
B
O
Me
Me
Me
Me
(28)
f1 (ppm)

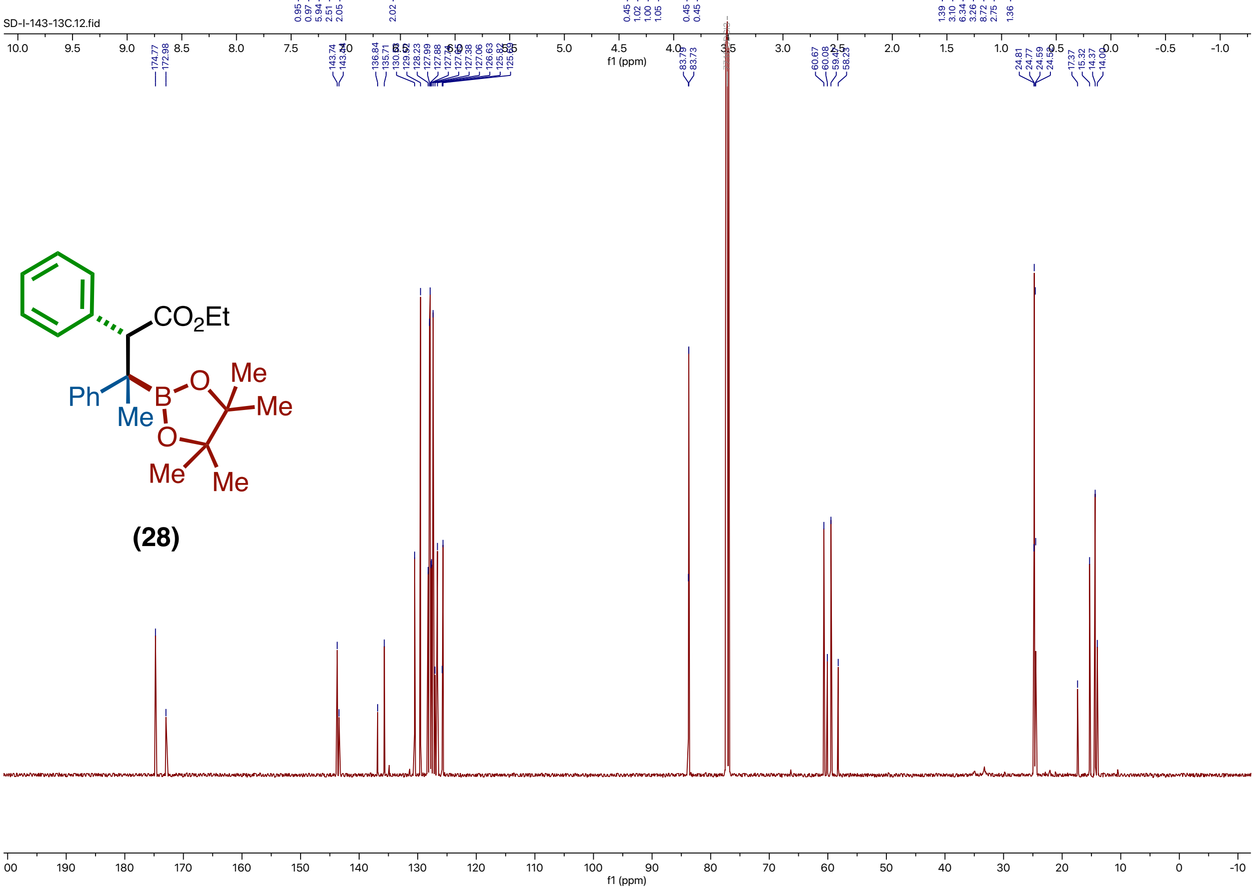
SD-I-143-13C.12.fid
CO2Et
Ph
Me
B
O
Me
Me
Me
Me
(28)
f1 (ppm)

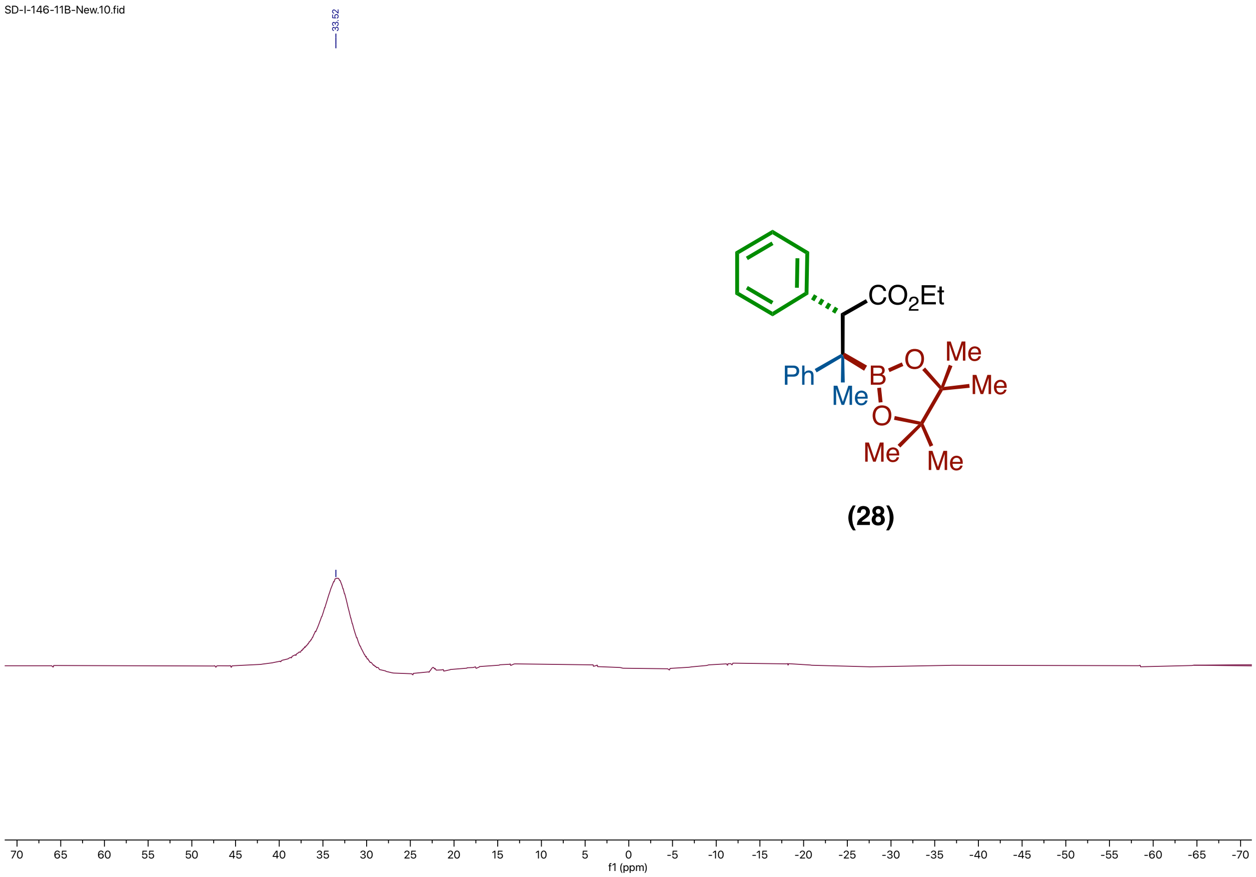
SD-I-146-11B-New.10.fid
33.52
CO2Et
Ph
Me
B
O
O
Me
Me
Me
Me
(28)
70 65 60 55 50 45 40 35 30 25 20 15 10 5 0 -5 -10 -15 -20 -25 -30 -35 -40 -45 -50 -55 -60 -65 -70
f1 (ppm)

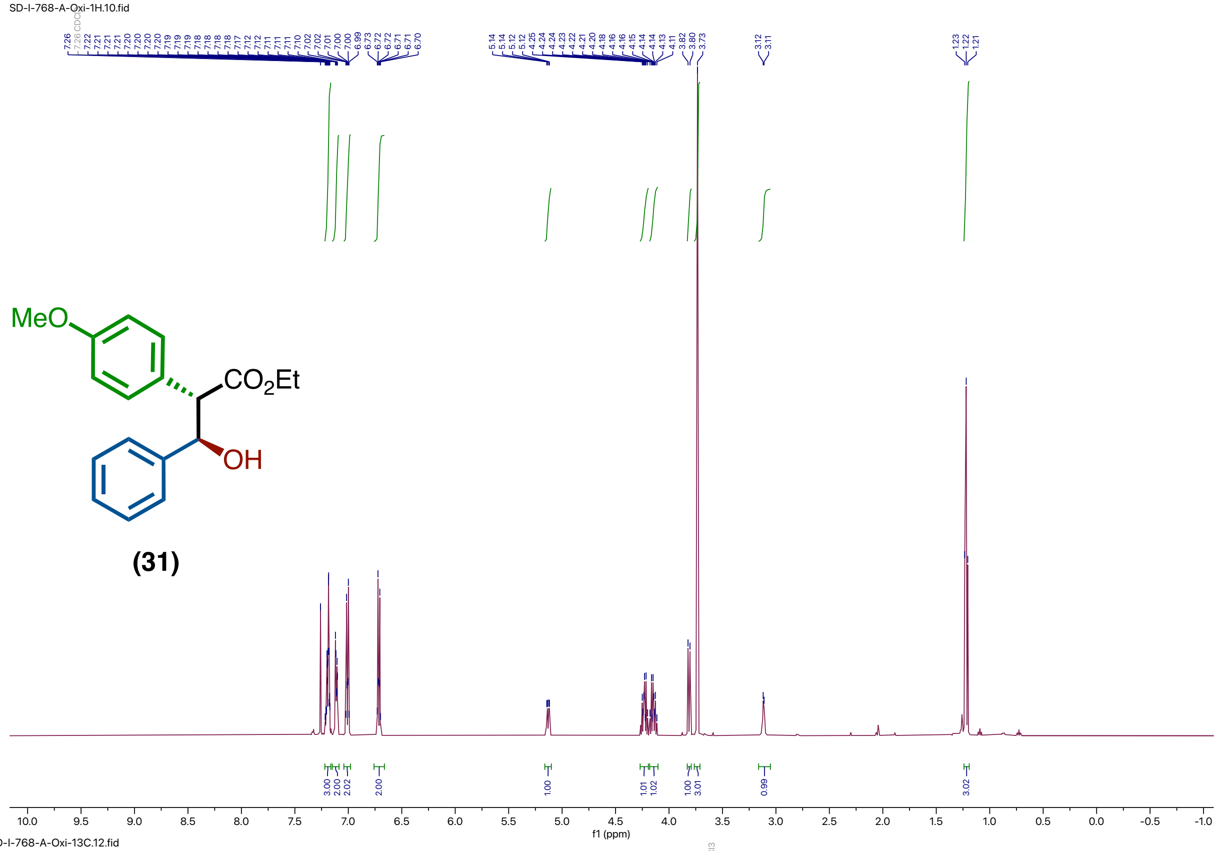
SD-I-768-A-Oxi-1H.10.fid
7.26
7.22 7.21 7.21 7.20 7.20 7.20 7.19 7.19 7.18 7.18 7.18 7.17 7.12 7.12 7.11 7.11 7.10 7.02 7.02 7.01 7.00 7.00 6.99 6.73 6.72 6.72 6.71 6.71 6.70
5.14 5.14 5.12 5.12 4.25 4.24 4.24 4.23 4.22 4.21 4.20 4.18 4.16 4.16 4.15 4.14 4.14 4.13 4.11 3.82 3.80 3.73
3.12 3.11
1.23 1.22 1.21
3.00 2.00 2.02 2.00 1.00 1.01 1.02 1.00 3.01 0.99 3.02
MeO
CO2Et
OH
(31)
f1 (ppm)

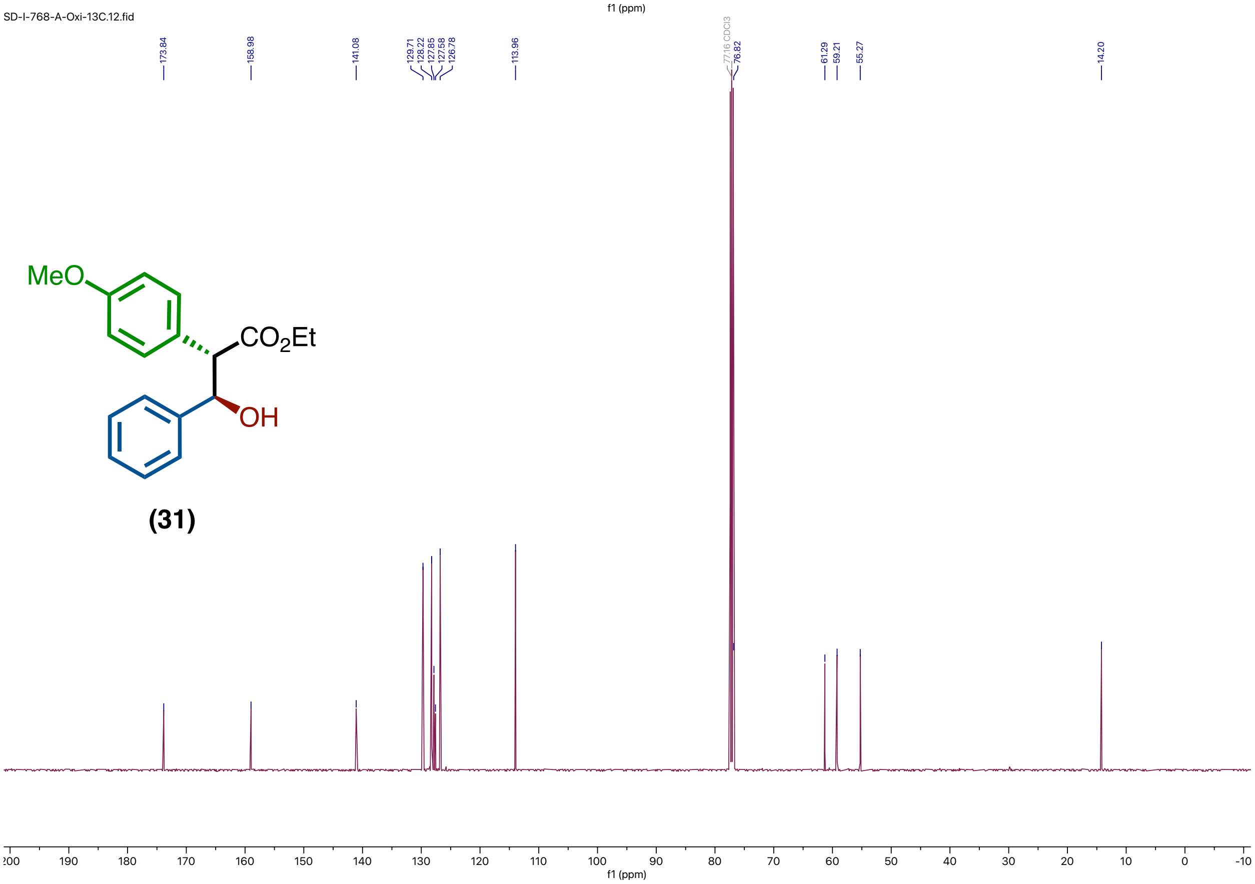
SD-I-768-A-Oxi-13C.12.fid
173.84
158.98
141.08
129.71 128.22 127.85 127.58 126.78
113.96
77.16 CDCl3
76.82
61.29 59.21 55.27
14.20
MeO
CO2Et
OH
(31)
f1 (ppm)

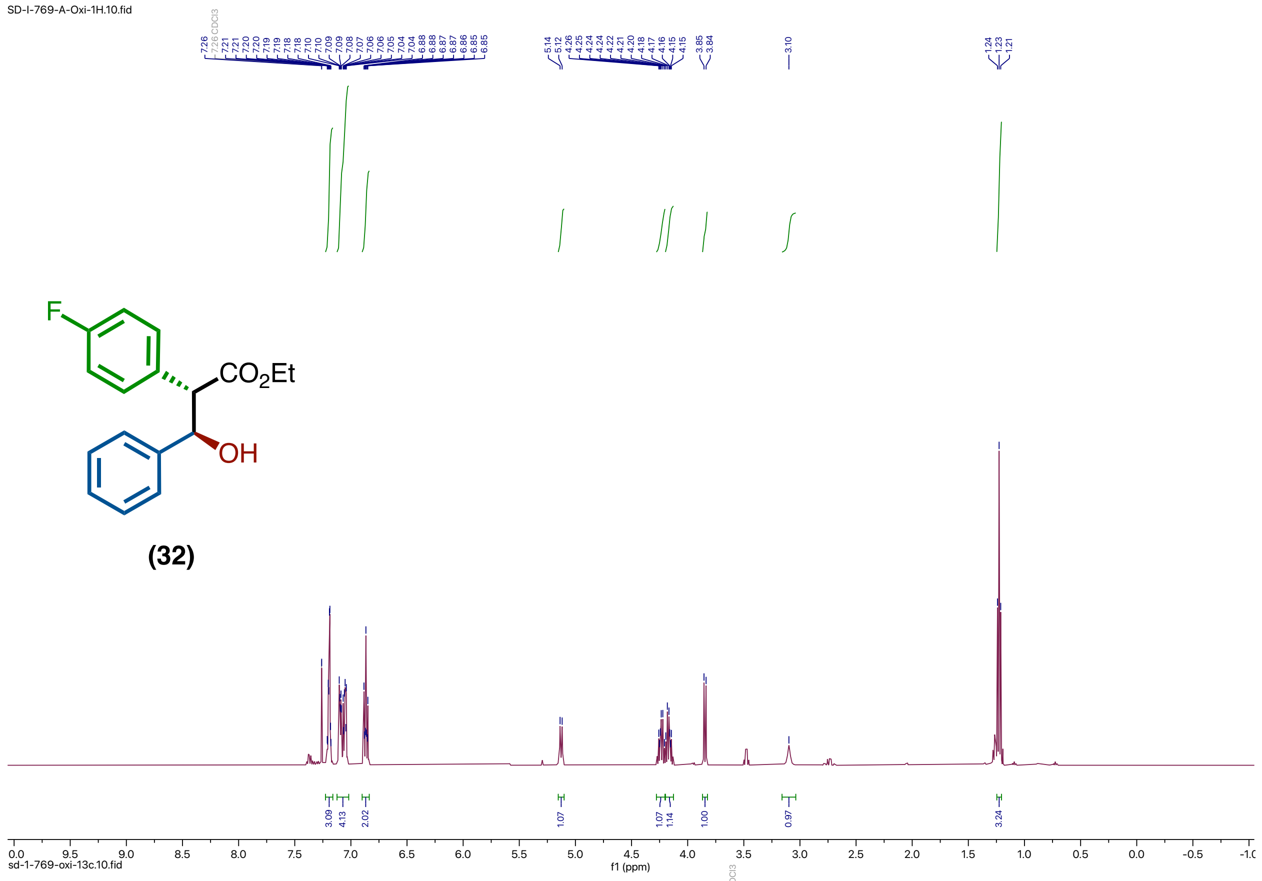

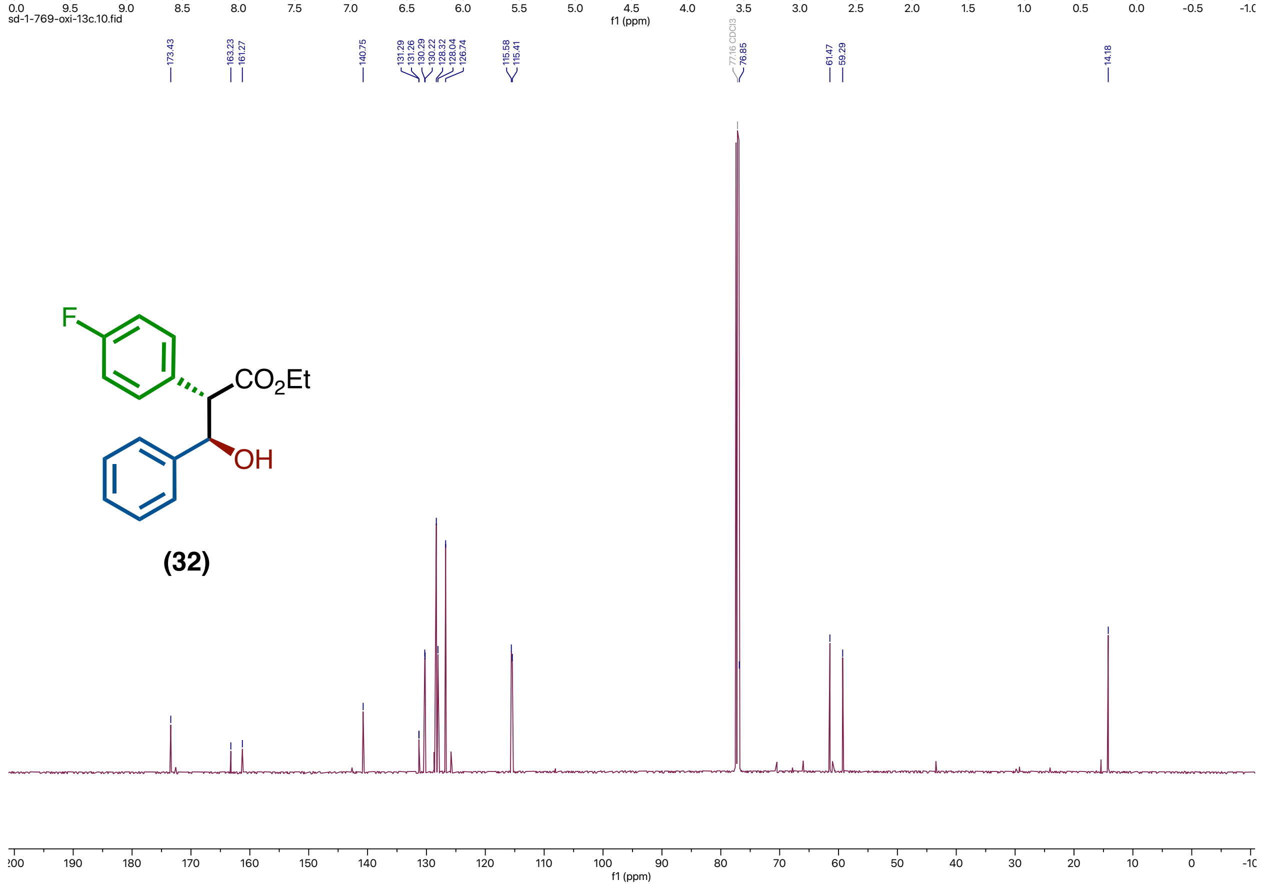

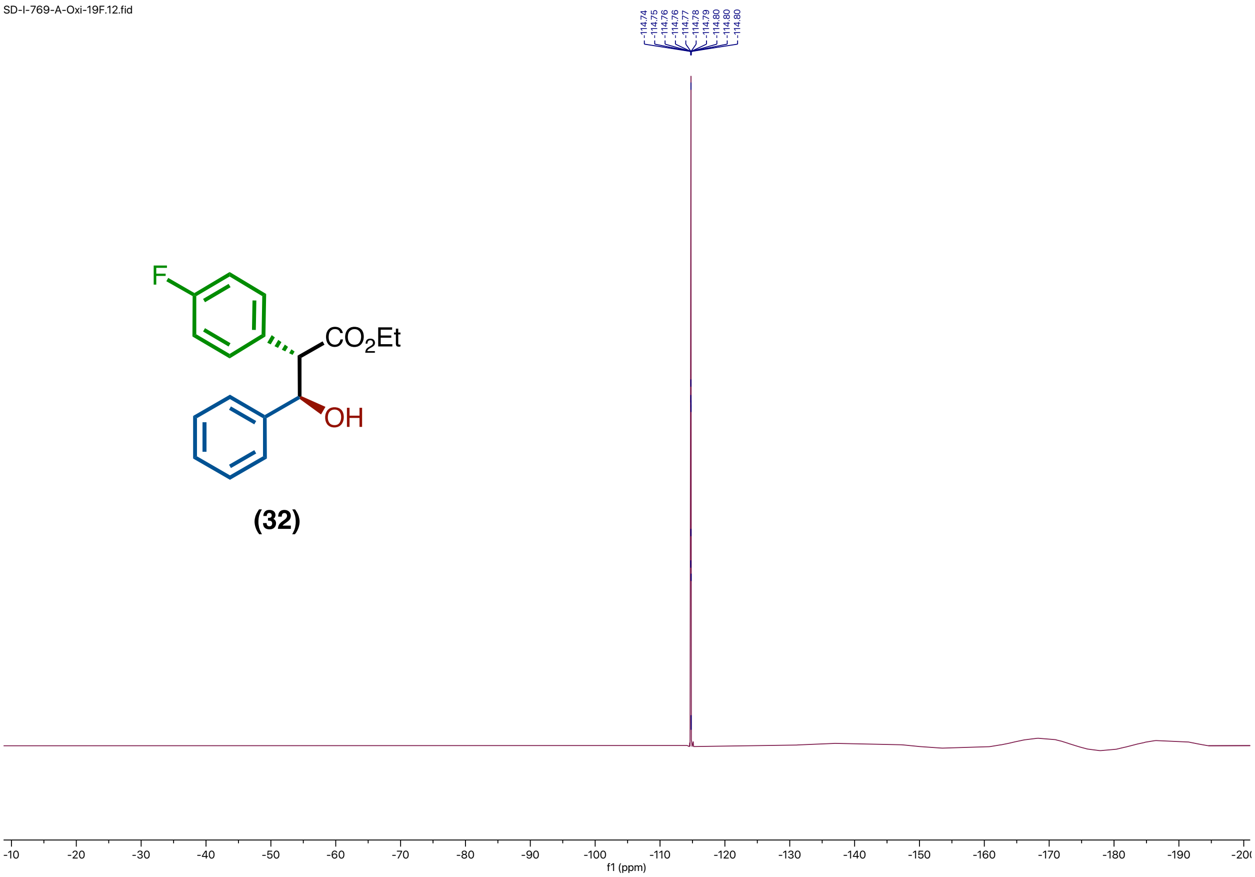
SD-I-769-A-Oxi-19F.12.fid
-114.74
-114.75
-114.76
-114.76
-114.77
-114.78
-114.79
-114.80
-114.80
-114.80
F
CO_2Et
OH
(32)
-10 -20 -30 -40 -50 -60 -70 -80 -90 -100 -110 -120 -130 -140 -150 -160 -170 -180 -190
f1 (ppm)

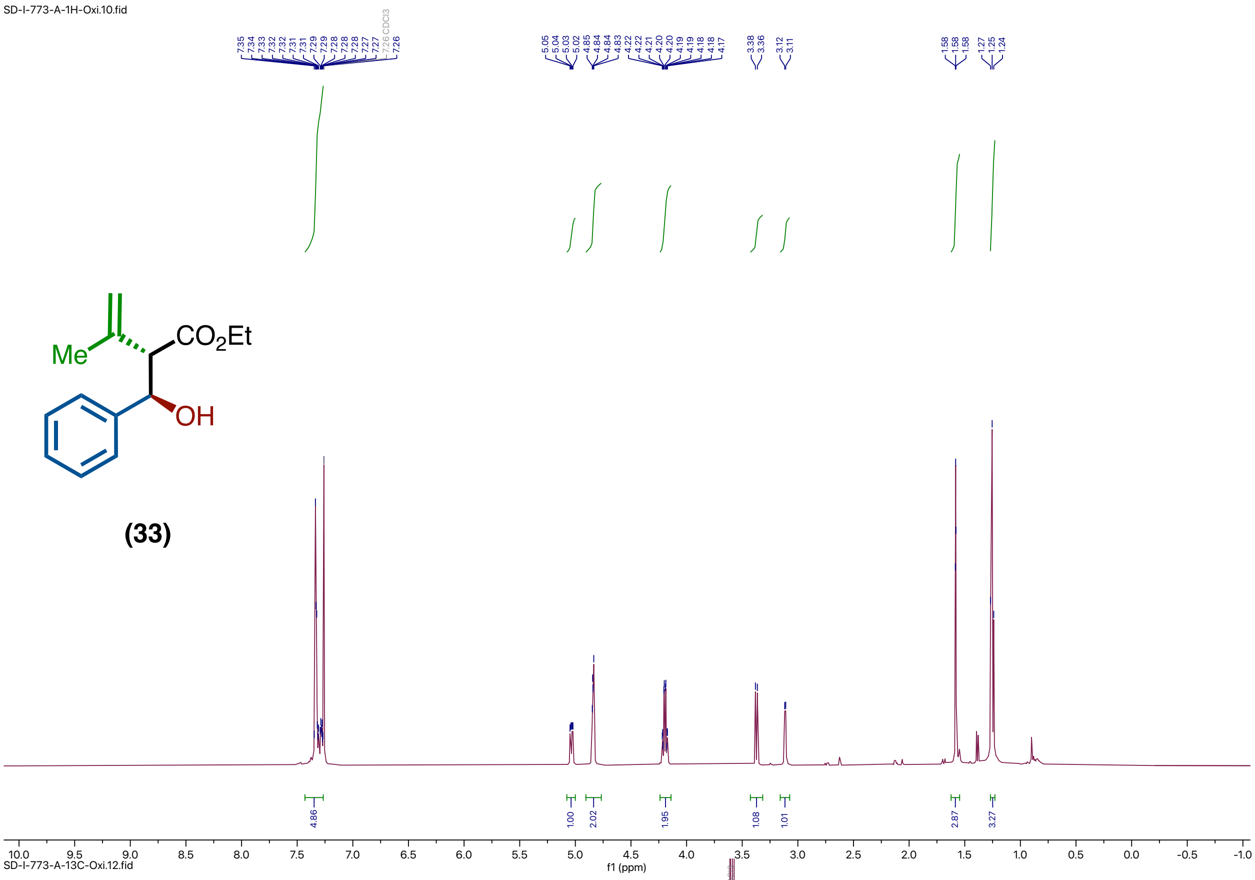
SD-I-773-A-1H-Oxi.10.fid
CO2Et
Me
OH
f1 (ppm)

(33)

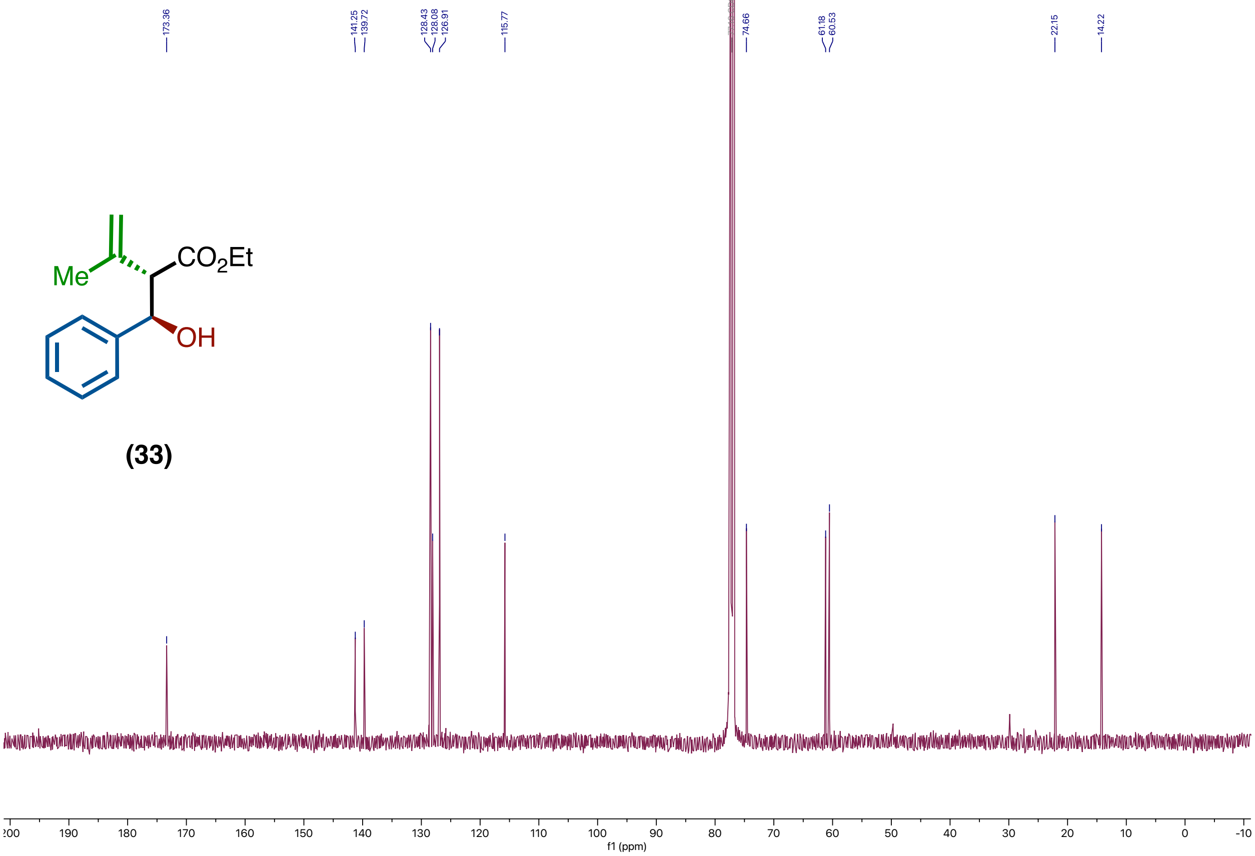
SD-I-773-A-13C-Oxi.12.fid
173.36
141.25
139.72
128.43
128.08
126.91
115.77
74.66
61.18
60.53
22.15
14.22
CO2Et
Me
OH
f1 (ppm)

(33)

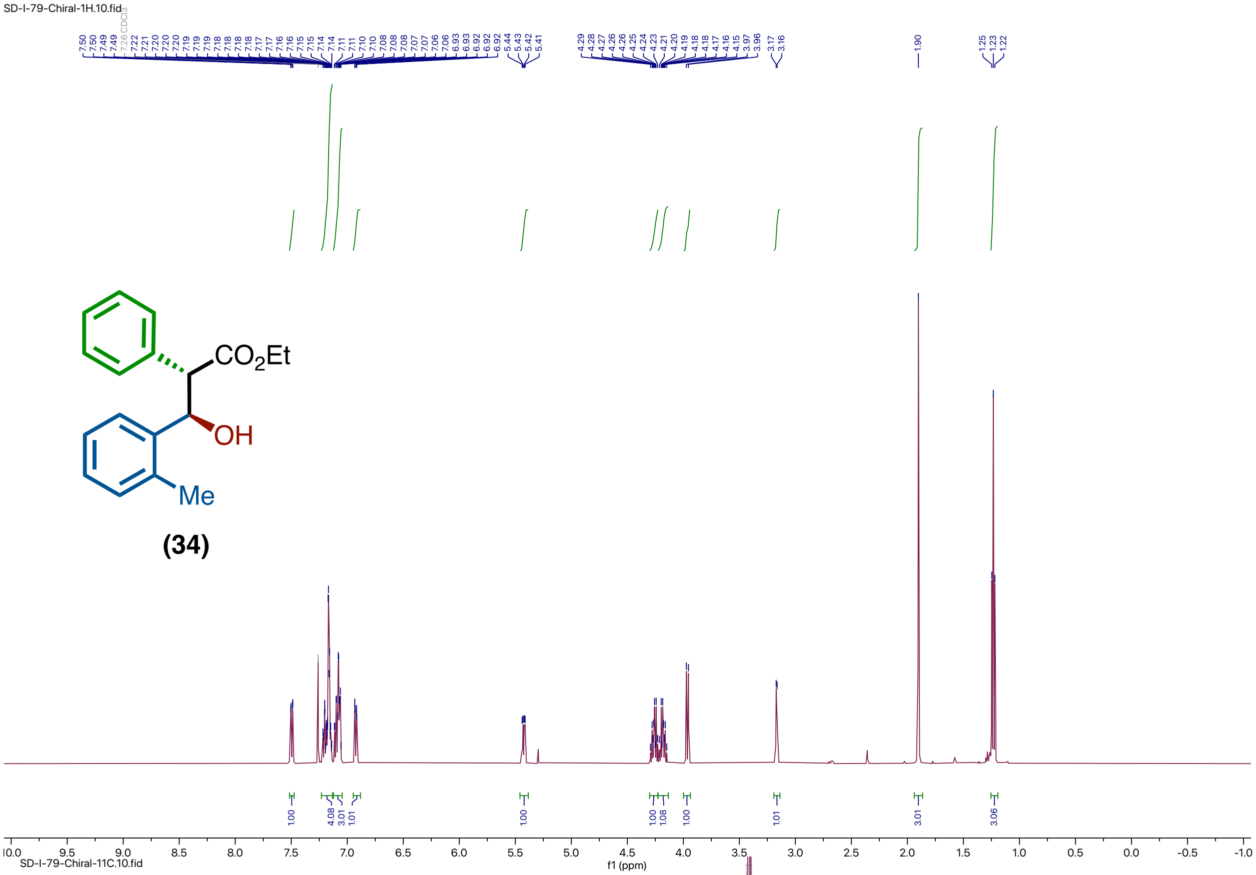
SD-I-79-Chiral-1H.10.fid
7.50 7.50 7.49 7.26 CDCl3 7.22 7.21 7.20 7.20 7.19 7.19 7.18 7.18 7.18 7.17 7.17 7.16 7.16 7.15 7.14 7.11 7.11 7.10 7.10 7.08 7.08 7.07 7.07 7.06 7.06 6.93 6.93 6.92 6.92 6.92 5.44 5.43 5.42 5.41
4.29 4.28 4.27 4.26 4.26 4.25 4.24 4.23 4.21 4.20 4.19 4.18 4.18 4.17 4.16 4.15 3.97 3.96 3.17 3.16
1.90
1.25 1.23 1.22
1.00 4.08 3.01 1.01 1.00 1.00 1.08 1.00 1.01 3.01 3.06
CO2Et
OH
Me
f1 (ppm)

(34)

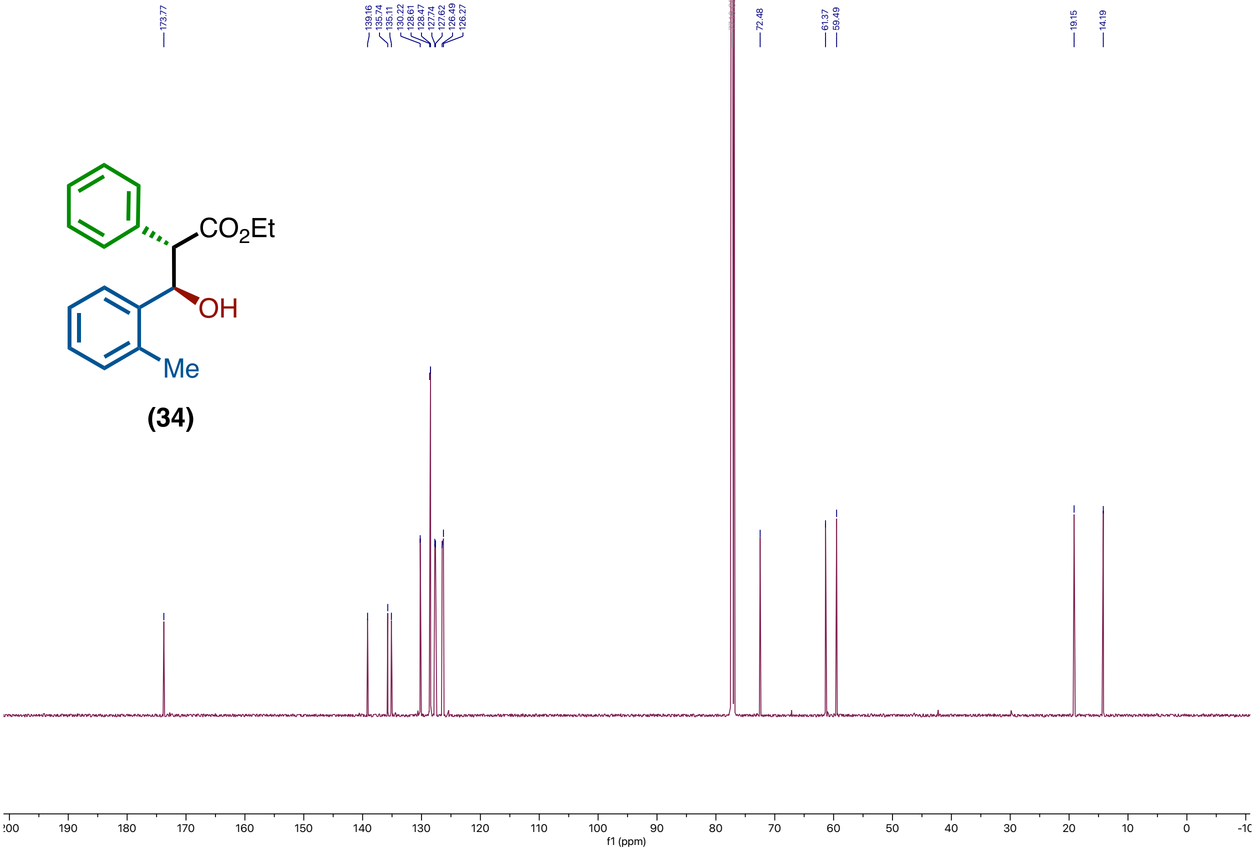
SD-I-79-Chiral-11C.10.fid
173.77
139.16 135.74 135.11 130.22 128.61 128.47 127.74 127.62 126.49 126.27
72.48
61.37 59.49
19.15 14.19
CO2Et
OH
Me
f1 (ppm)

(34)

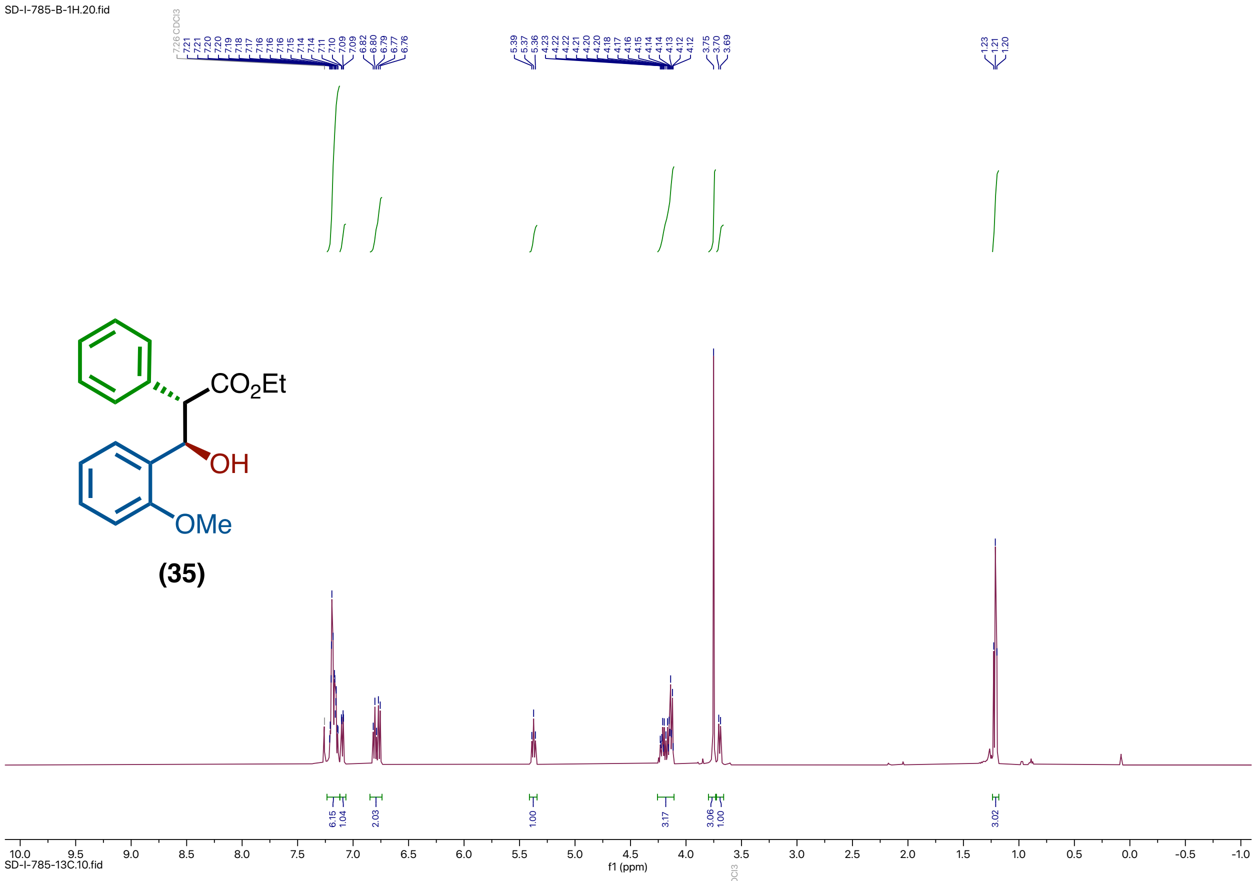
SD-I-785-B-1H.20.fid
7.26 CDCl3
7.21
7.21
7.20
7.19
7.18
7.17
7.16
7.16
7.15
7.14
7.14
7.11
7.10
7.09
7.09
6.82
6.80
6.79
6.77
6.76
5.39
5.37
5.36
4.23
4.22
4.22
4.21
4.20
4.20
4.18
4.17
4.16
4.15
4.14
4.13
4.12
4.12
3.75
3.70
3.69
1.23
1.21
1.20
CO2Et
OH
OMe
(35)
6.15
1.04
2.03
1.00
3.17
3.06
1.00
3.02
f1 (ppm)

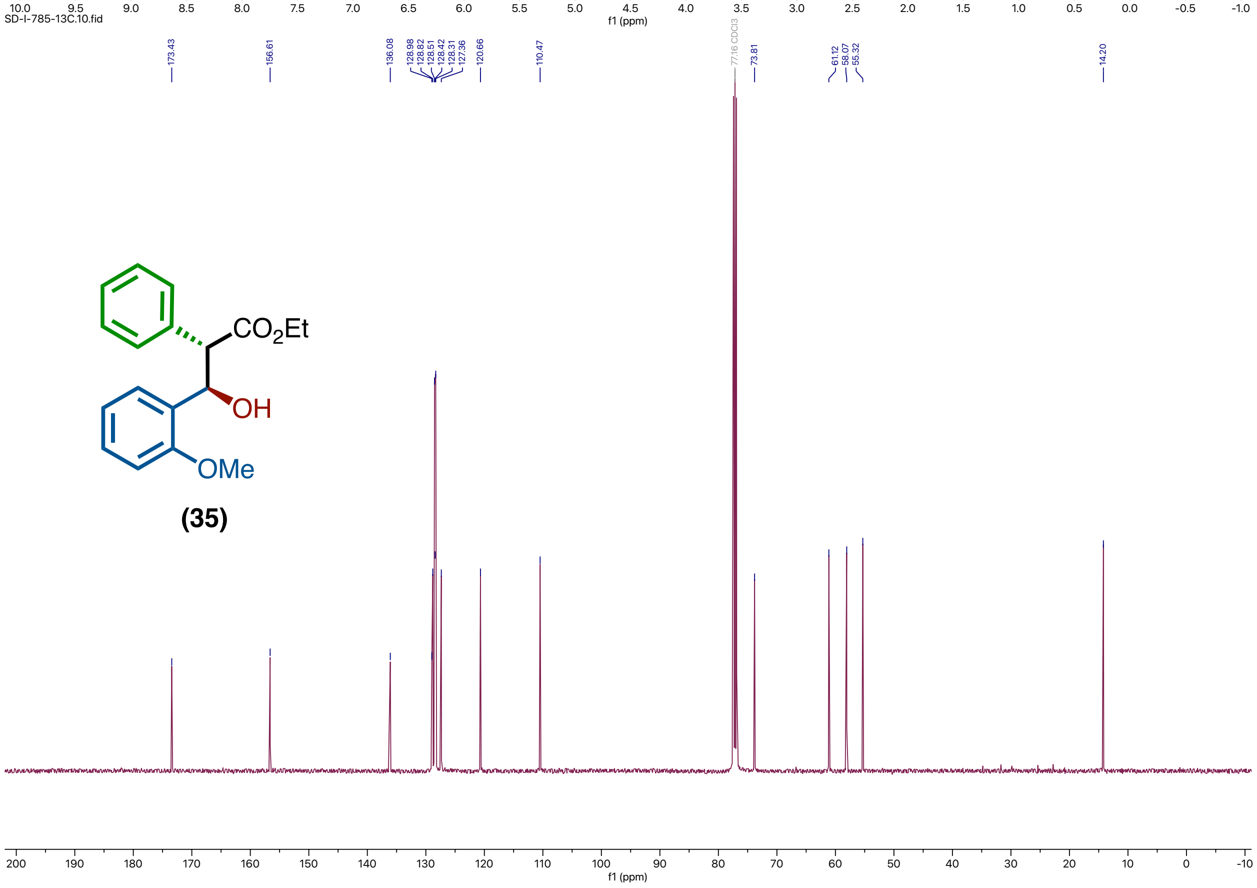
SD-I-785-13C.10.fid
173.43
156.61
136.08
128.98
128.82
128.51
128.42
128.31
127.36
120.66
110.47
77.16 CDCl3
73.81
61.12
58.07
55.32
14.20
CO2Et
OH
OMe
(35)
f1 (ppm)

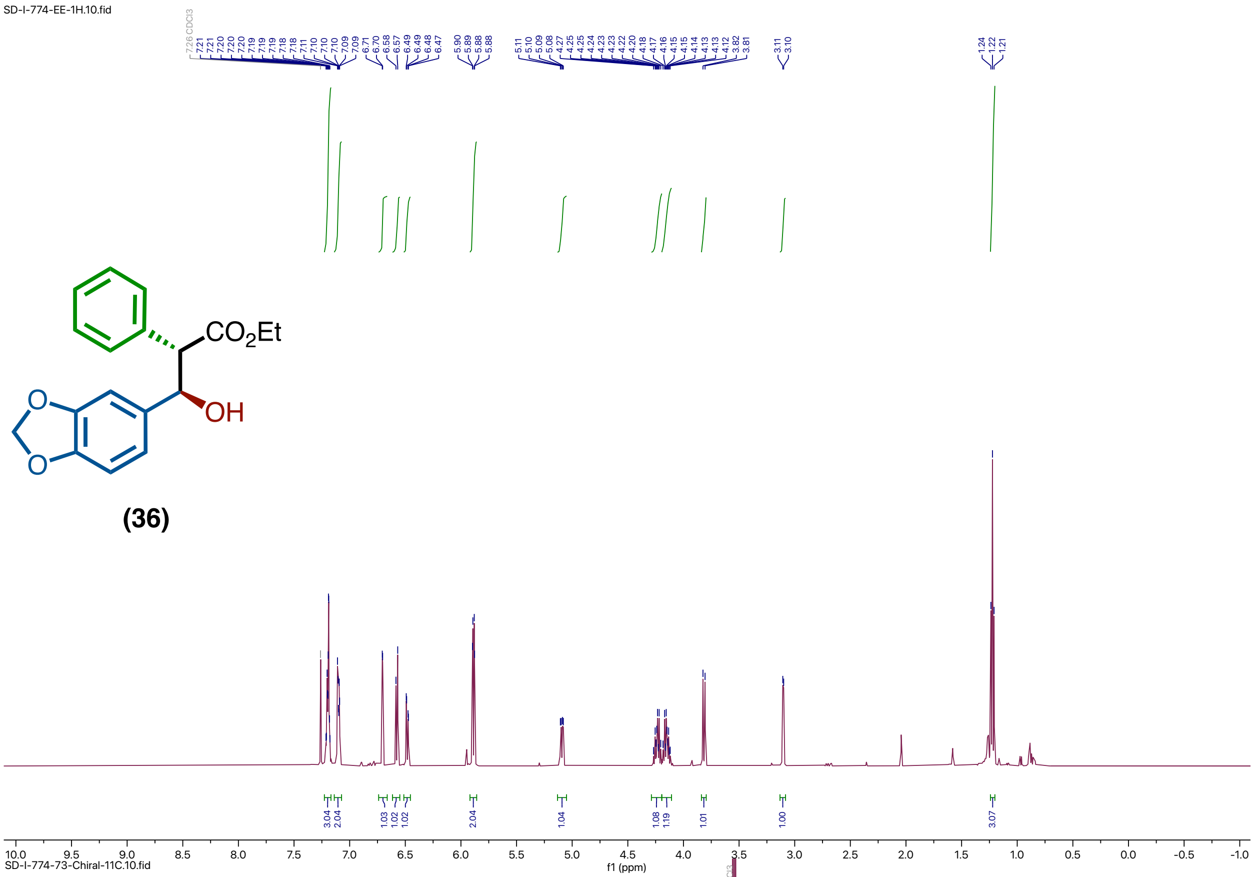
SD-I-774-EE-1H.10.fid
7.26 CDCl3
7.21 7.21 7.20 7.20 7.20 7.19 7.19 7.18 7.18 7.11 7.10 7.10 7.09 7.09 6.71 6.70 6.58 6.57 6.49 6.49 6.48 6.47 5.90 5.89 5.88 5.88 5.11 5.10 5.09 5.08 4.27 4.25 4.25 4.24 4.23 4.23 4.22 4.20 4.18 4.17 4.16 4.15 4.14 4.13 4.13 4.12 3.82 3.81 3.11 3.10 1.24 1.22 1.21
CO2Et
OH
O
O
(36)
3.04 2.04 1.03 1.02 1.02 2.04 1.04 1.08 1.19 1.01 1.00 3.07
f1 (ppm)

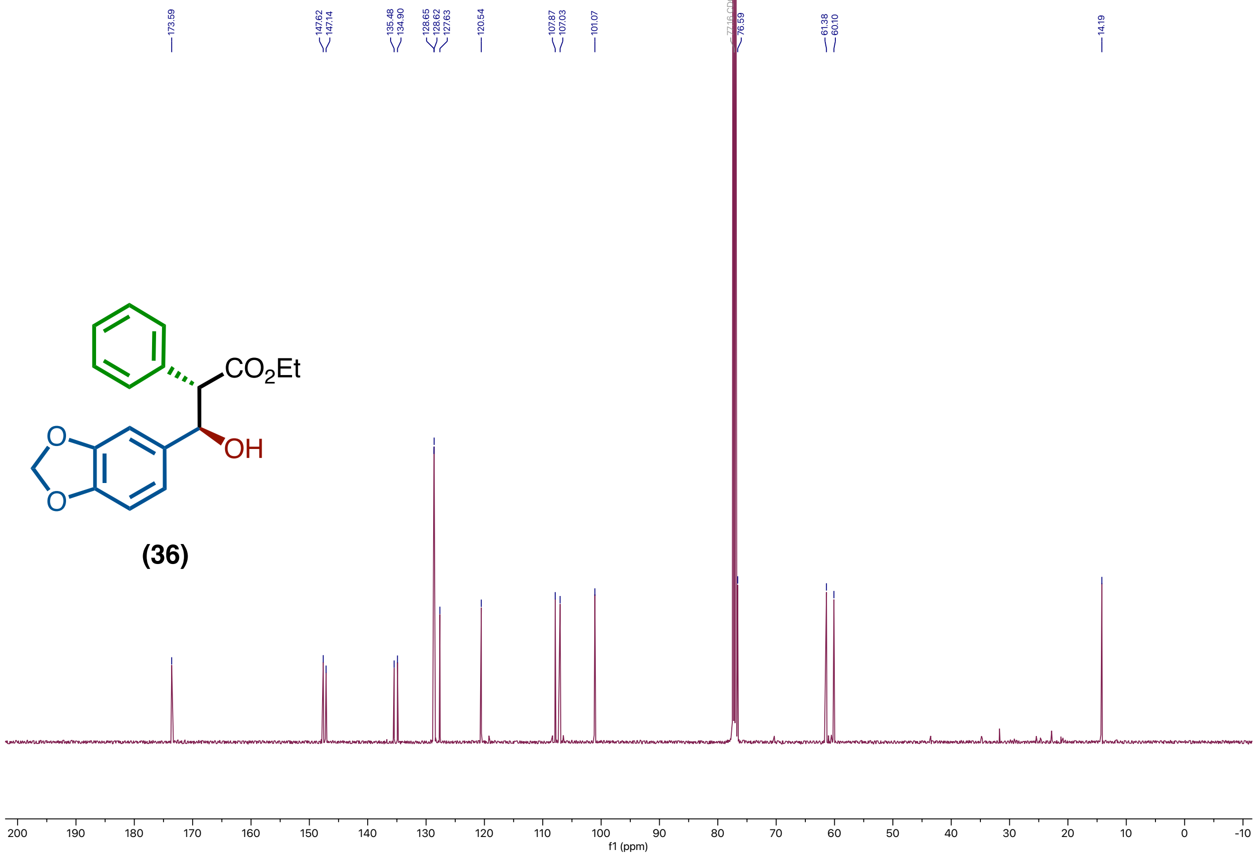
SD-I-774-73-Chiral-11C.10.fid
173.59 147.62 147.14 135.48 134.90 128.65 128.62 127.63 120.54 107.87 107.03 101.07 77.16 CDCl3 76.59 61.38 60.10 14.19
CO2Et
OH
O
O
(36)
f1 (ppm)

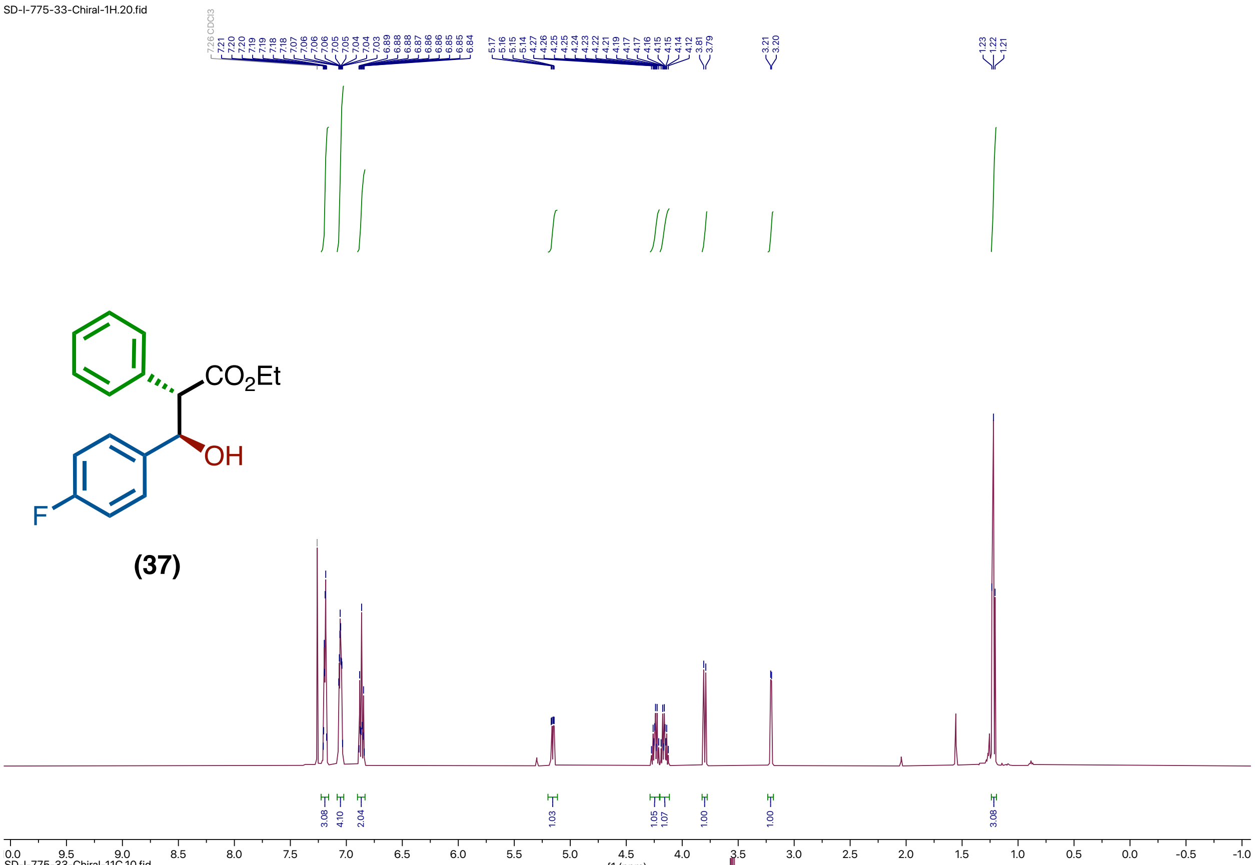
SD-I-775-33-Chiral-1H.20.fid
CO2Et
OH
F
(37)
3.08
4.10
2.04
1.03
1.05
1.07
1.00
1.00
3.08
f1 (ppm)

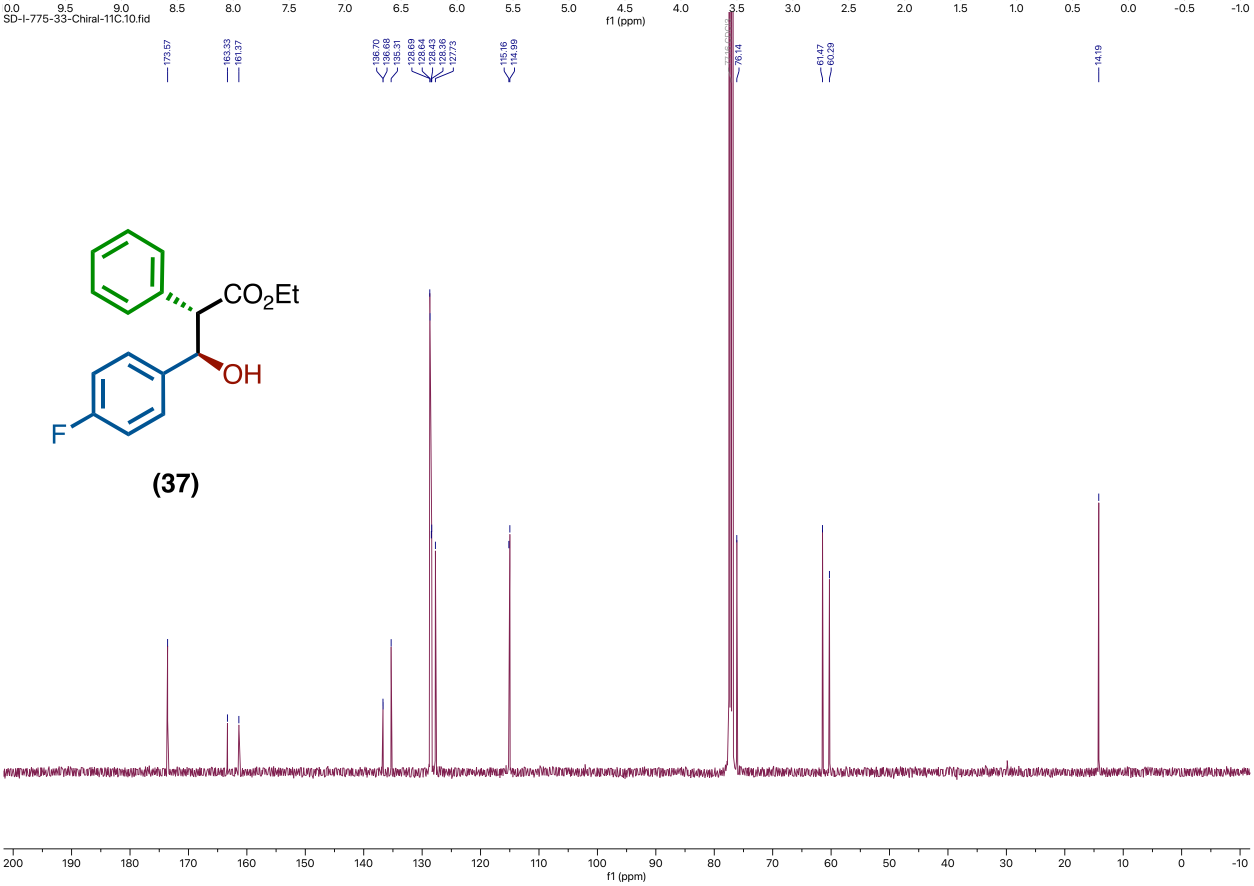
SD-I-775-33-Chiral-11C.10.fid
173.57
163.33
161.37
136.70
136.68
135.31
128.69
128.64
128.43
128.36
127.73
115.16
114.99
76.14
61.47
60.29
14.19
CO2Et
OH
F
(37)
f1 (ppm)

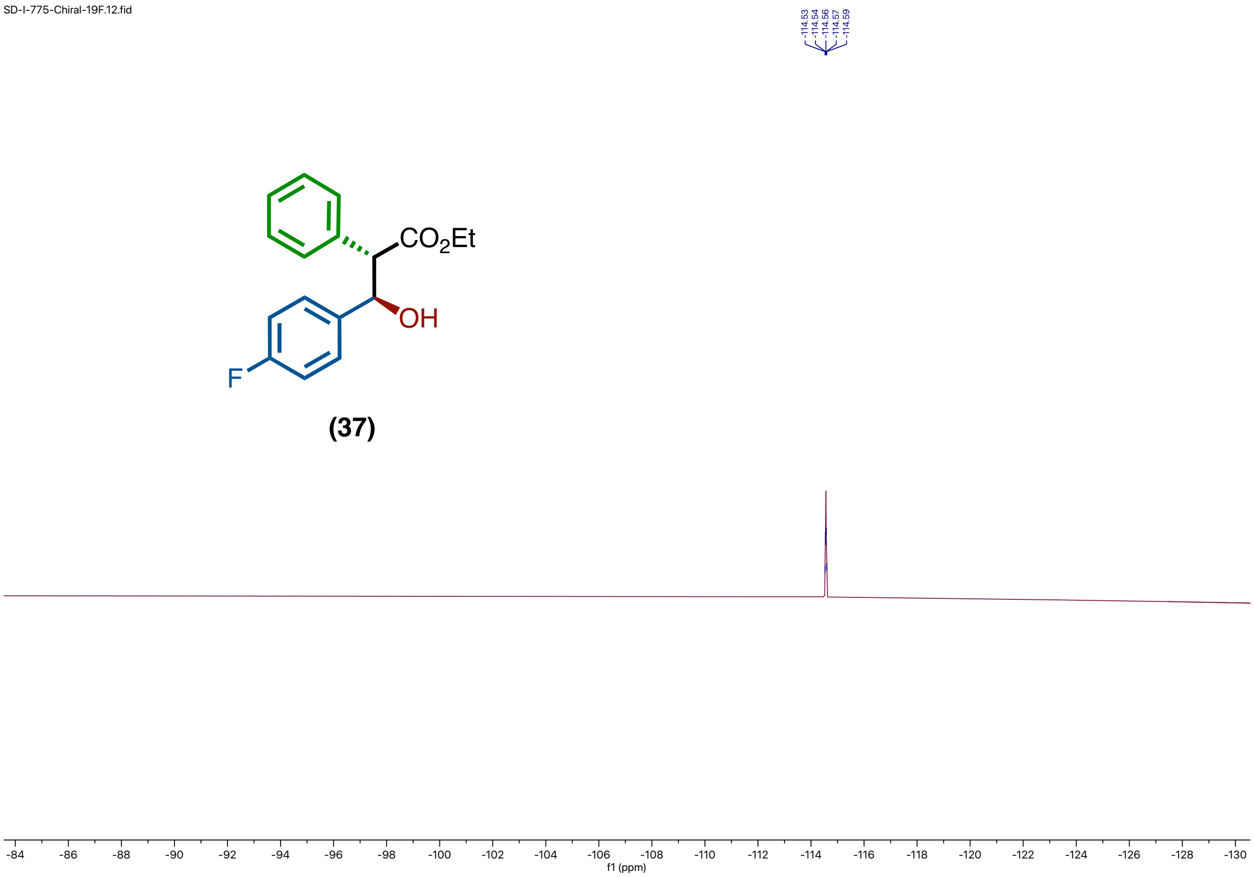
SD-I-775-Chiral-19F.12.fid
-114.53
-114.54
-114.56
-114.57
-114.59
CO2Et
OH
F
(37)
-84 -86 -88 -90 -92 -94 -96 -98 -100 -102 -104 -106 -108 -110 -112 -114 -116 -118 -120 -122 -124 -126 -128 -130
f1 (ppm)

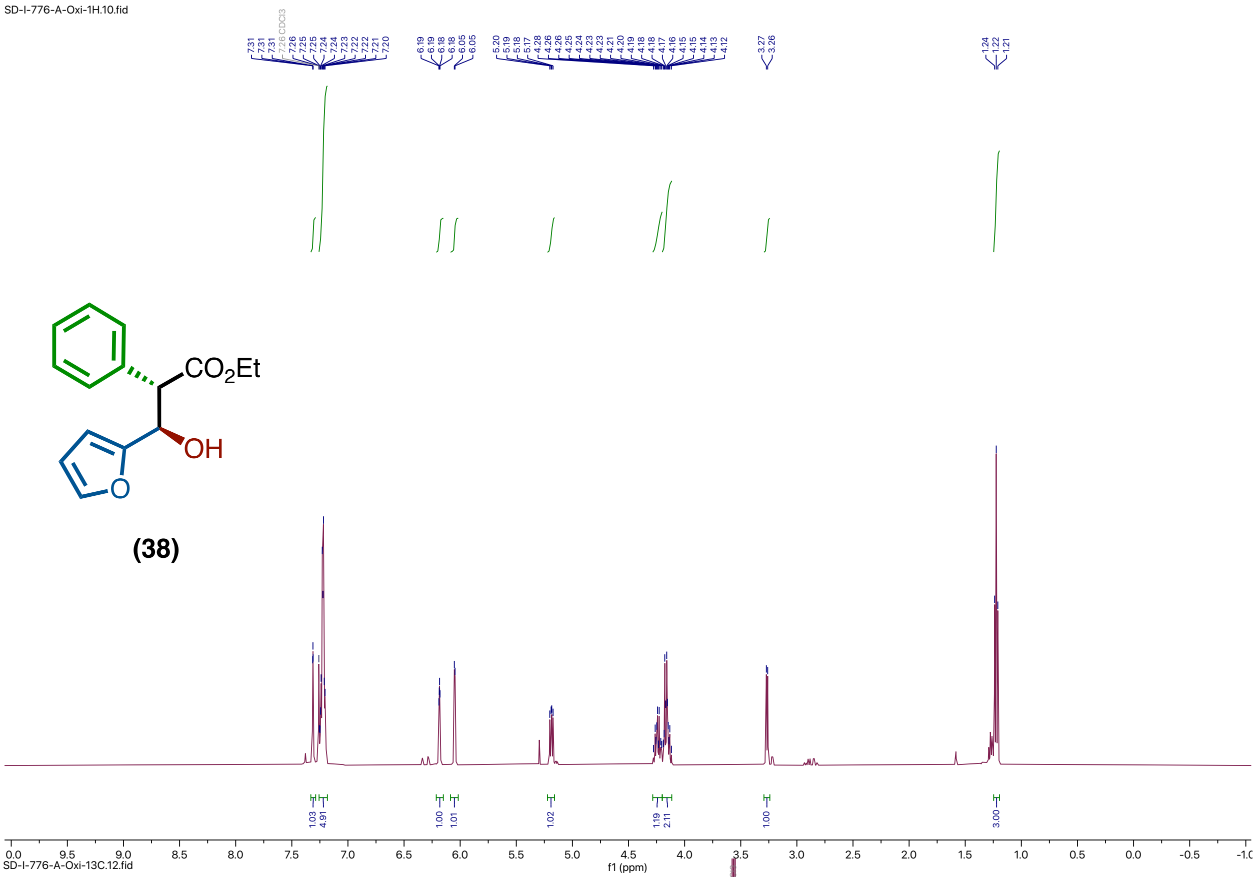
SD-I-776-A-Oxi-1H.10.fid
7.31 7.31 7.31 7.26 CDCl3 7.26 7.25 7.25 7.24 7.24 7.23 7.22 7.22 7.21 7.20
6.19 6.19 6.18 6.18 6.05 6.05
5.20 5.19 5.18 5.17 4.28 4.26 4.26 4.25 4.24 4.23 4.23 4.21 4.20 4.19 4.18 4.18 4.17 4.16 4.15 4.15 4.14 4.13 4.12
3.27 3.26
1.24 1.22 1.21
CO2Et
OH
O
(38)
1.03 4.91 1.00 1.01 1.02 1.19 2.11 1.00 3.00
f1 (ppm)

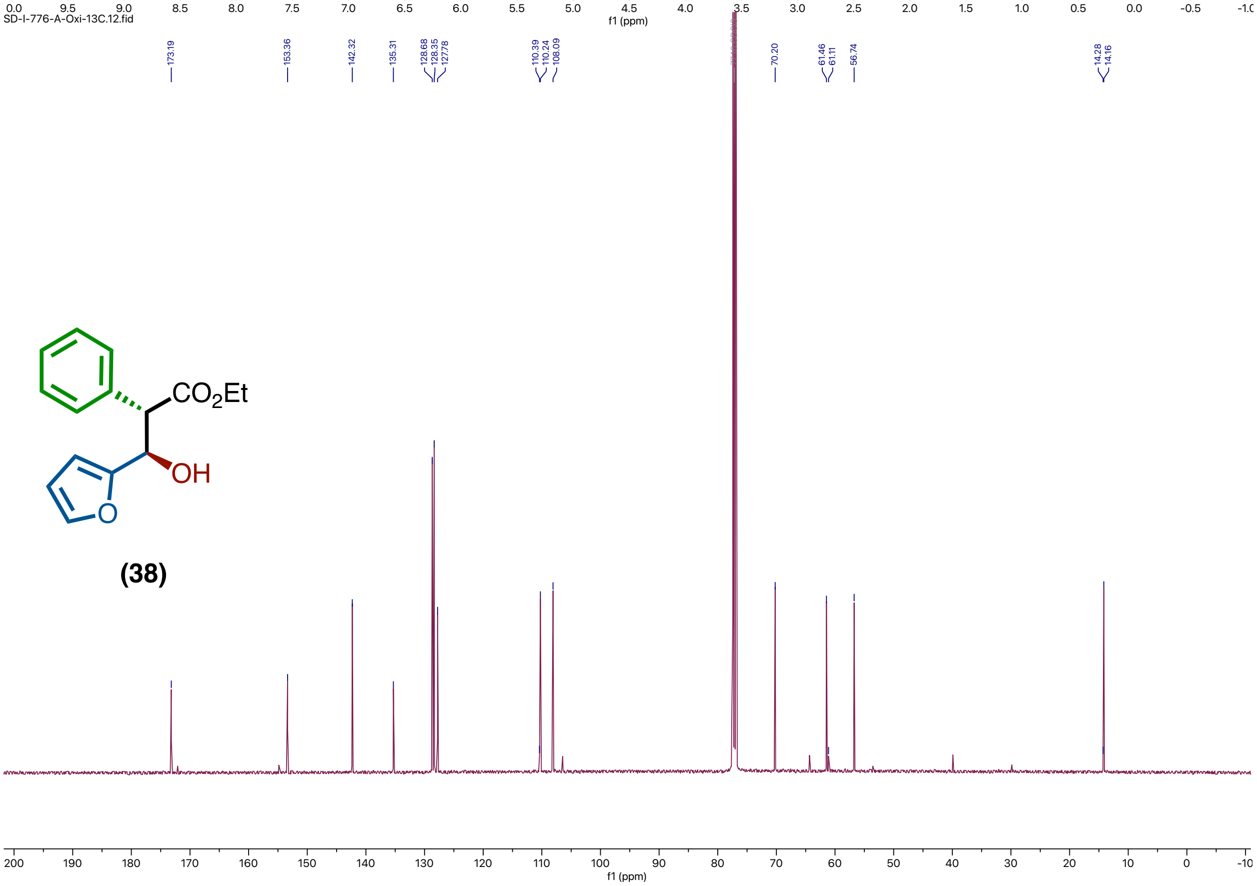
SD-I-776-A-Oxi-13C.12.fid
173.19
153.36
142.32
135.31
128.68 128.35 127.78
110.39 110.24 108.09
70.20
61.46 61.11
56.74
14.28 14.16
CO2Et
OH
O
(38)
f1 (ppm)

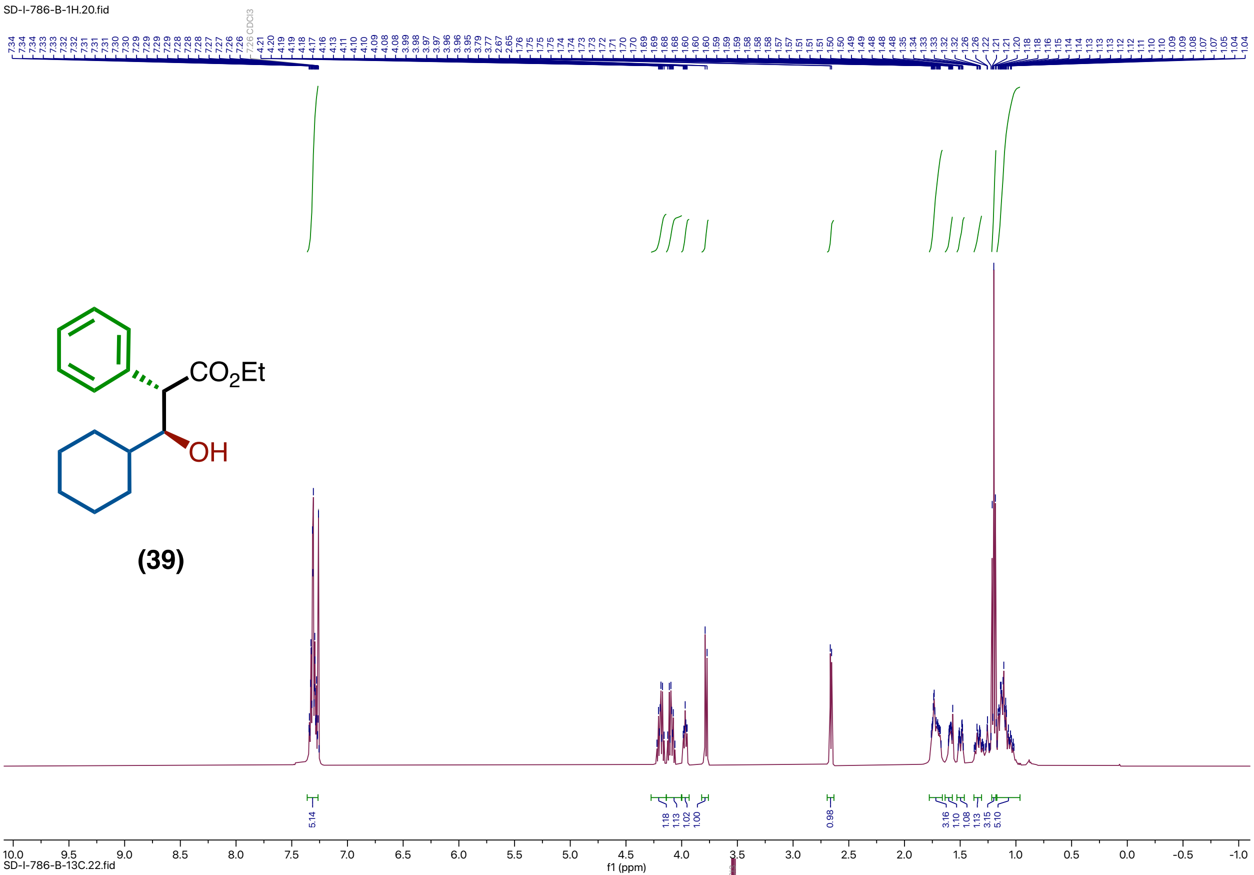

SD-I-786-B-1H.20.fid
7.34 7.34 7.34 7.33 7.33 7.32 7.32 7.31 7.31 7.31 7.30 7.30 7.29 7.29 7.29 7.29 7.28 7.28 7.28 7.27 7.27 7.26 7.26 7.26 CDCl3 4.21 4.20 4.19 4.19 4.18 4.17 4.16 4.13 4.11 4.10 4.10 4.09 4.08 4.08 3.99 3.98 3.97 3.97 3.96 3.96 3.95 3.79 3.77 2.67 2.65 1.76 1.75 1.75 1.74 1.74 1.73 1.73 1.72 1.71 1.70 1.70 1.69 1.69 1.68 1.68 1.60 1.60 1.60 1.59 1.59 1.59 1.58 1.58 1.58 1.57 1.57 1.51 1.51 1.51 1.50 1.50 1.49 1.49 1.48 1.48 1.48 1.35 1.34 1.33 1.33 1.32 1.32 1.26 1.26 1.22 1.21 1.21 1.20 1.18 1.18 1.16 1.15 1.14 1.14 1.13 1.13 1.13 1.12 1.11 1.10 1.10 1.09 1.08 1.07 1.05 1.04 1.04
CO2Et
OH
(39)
5.14
1.18 1.13 1.02 1.00
0.98
3.16 1.10 1.08 1.13 3.15 5.10
10.0 9.5 9.0 8.5 8.0 7.5 7.0 6.5 6.0 5.5 5.0 4.5 4.0 3.5 3.0 2.5 2.0 1.5 1.0 0.5 0.0 -0.5 -1.0
f1 (ppm)

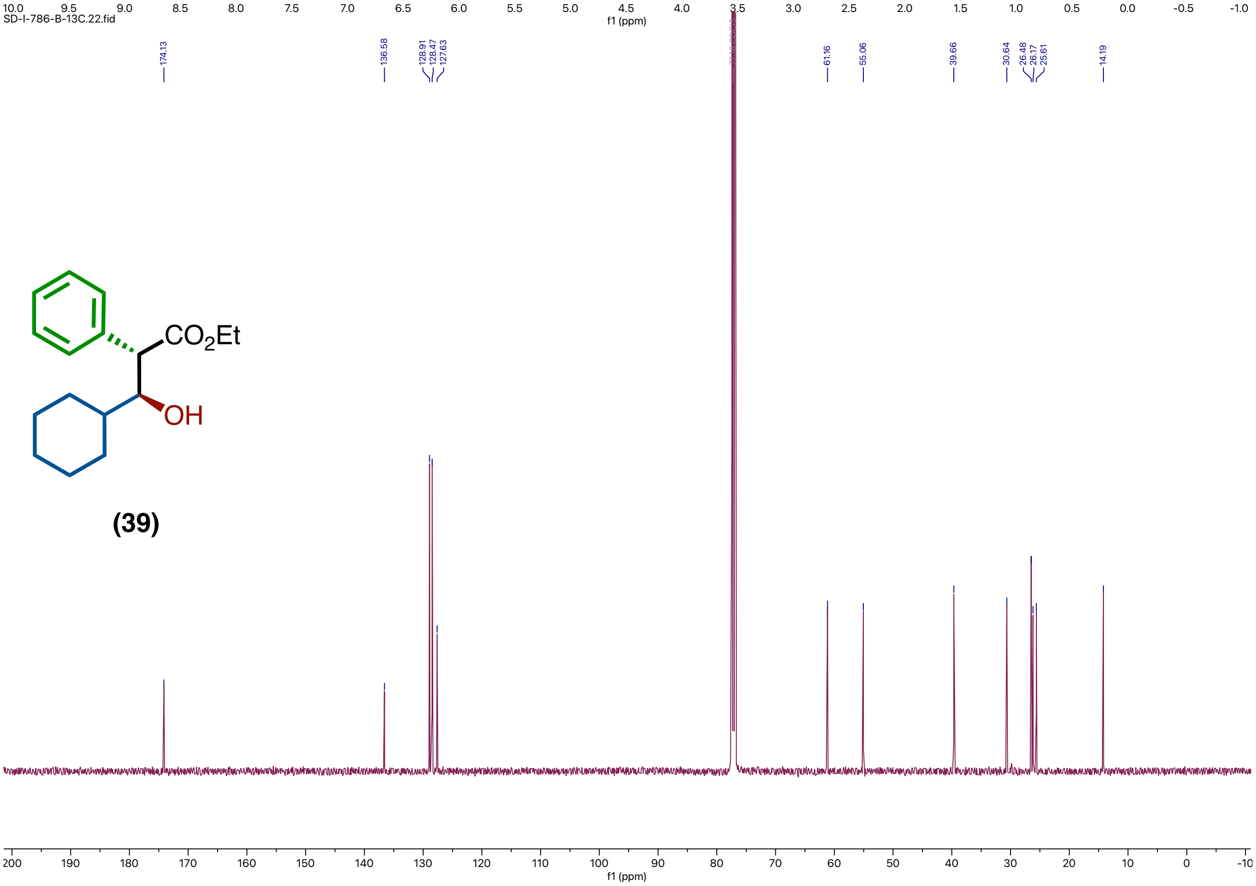

SD-I-786-B-13C.22.fid
174.13
136.58
128.91 128.47 127.63
61.16
55.06
39.66
30.64 26.48 26.17 25.61
14.19
CO2Et
OH
(39)
200 190 180 170 160 150 140 130 120 110 100 90 80 70 60 50 40 30 20 10 0 -10
f1 (ppm)

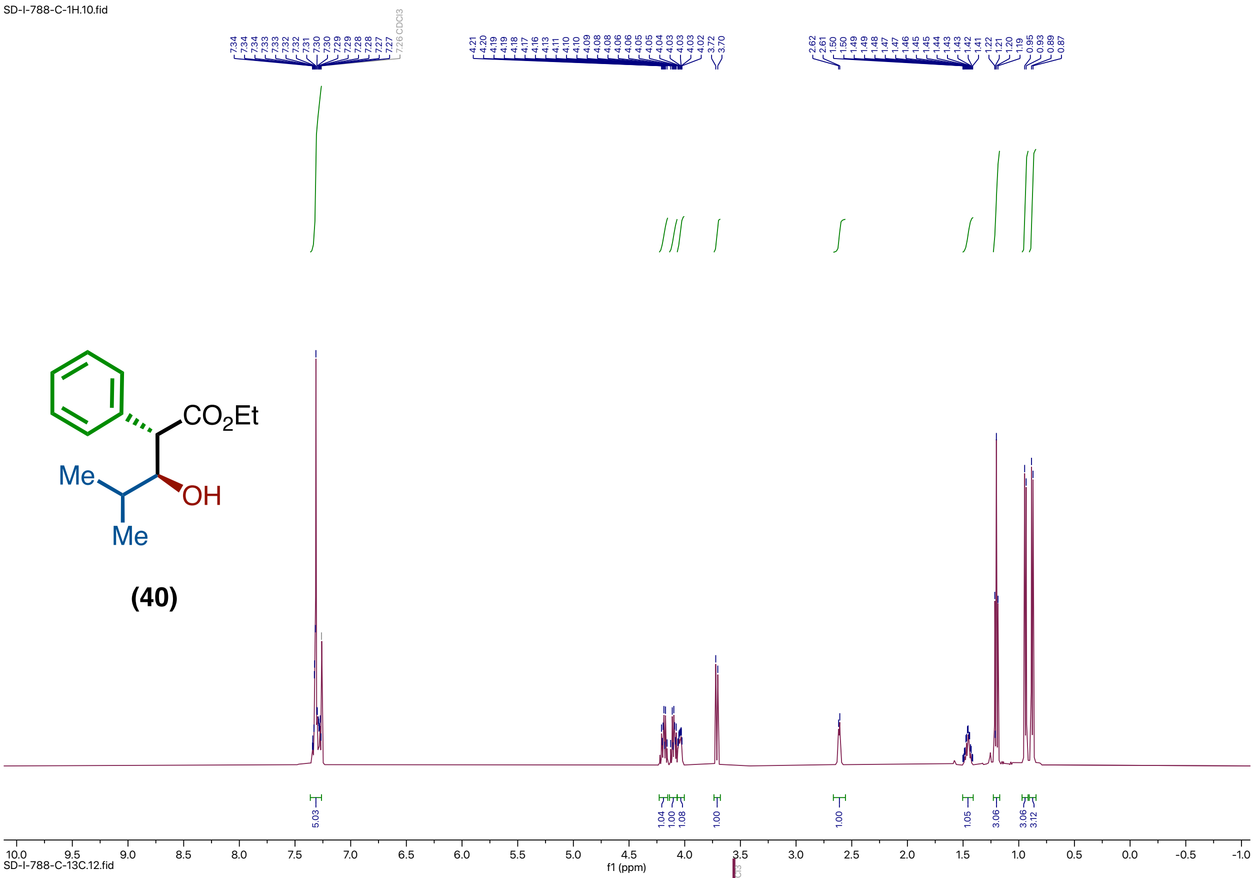
SD-I-788-C-1H.10.fid
CO2Et
Me
OH
Me
(40)
f1 (ppm)

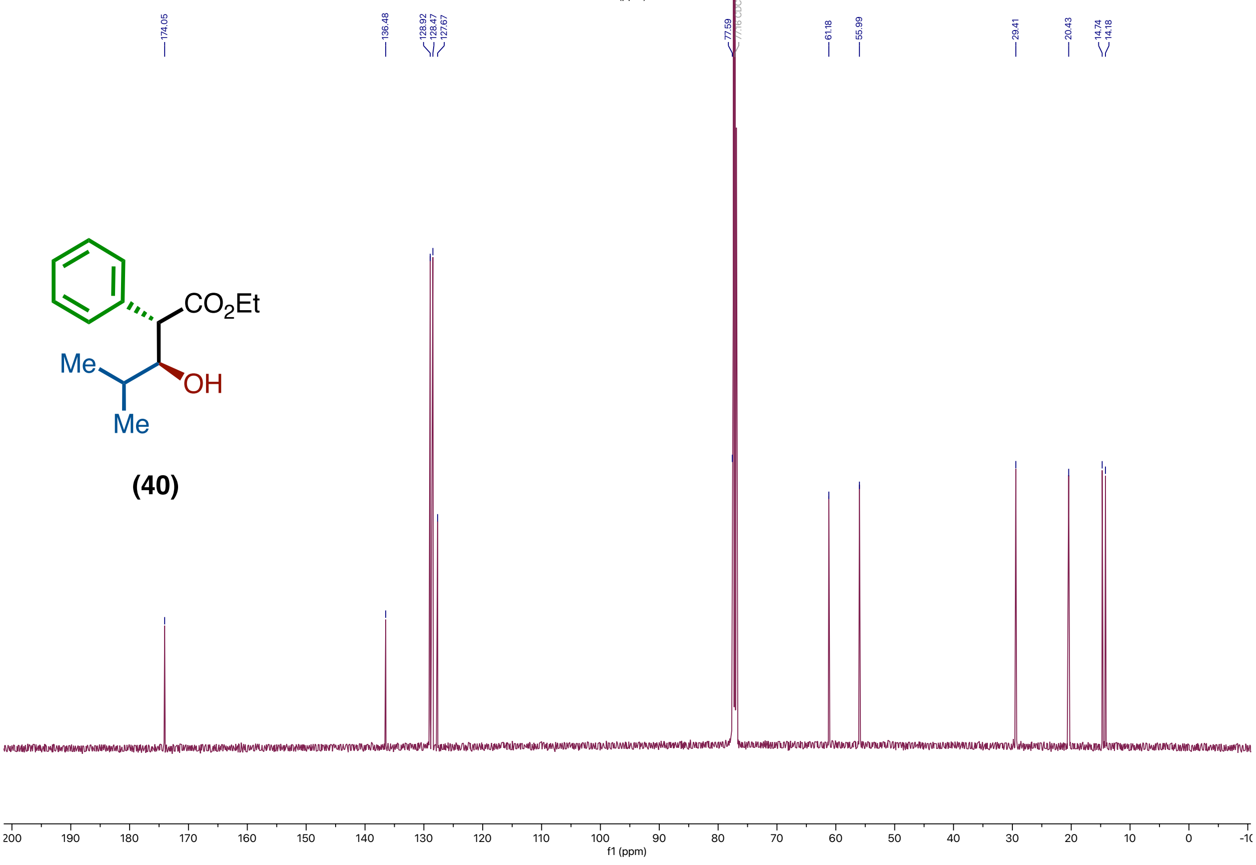
SD-I-788-C-13C.12.fid
174.05
136.48
128.92
128.47
127.67
77.59
61.18
55.99
29.41
20.43
14.74
14.18
CO2Et
Me
OH
Me
(40)
f1 (ppm)

SD-I-135-Chiral-1H.10.fid

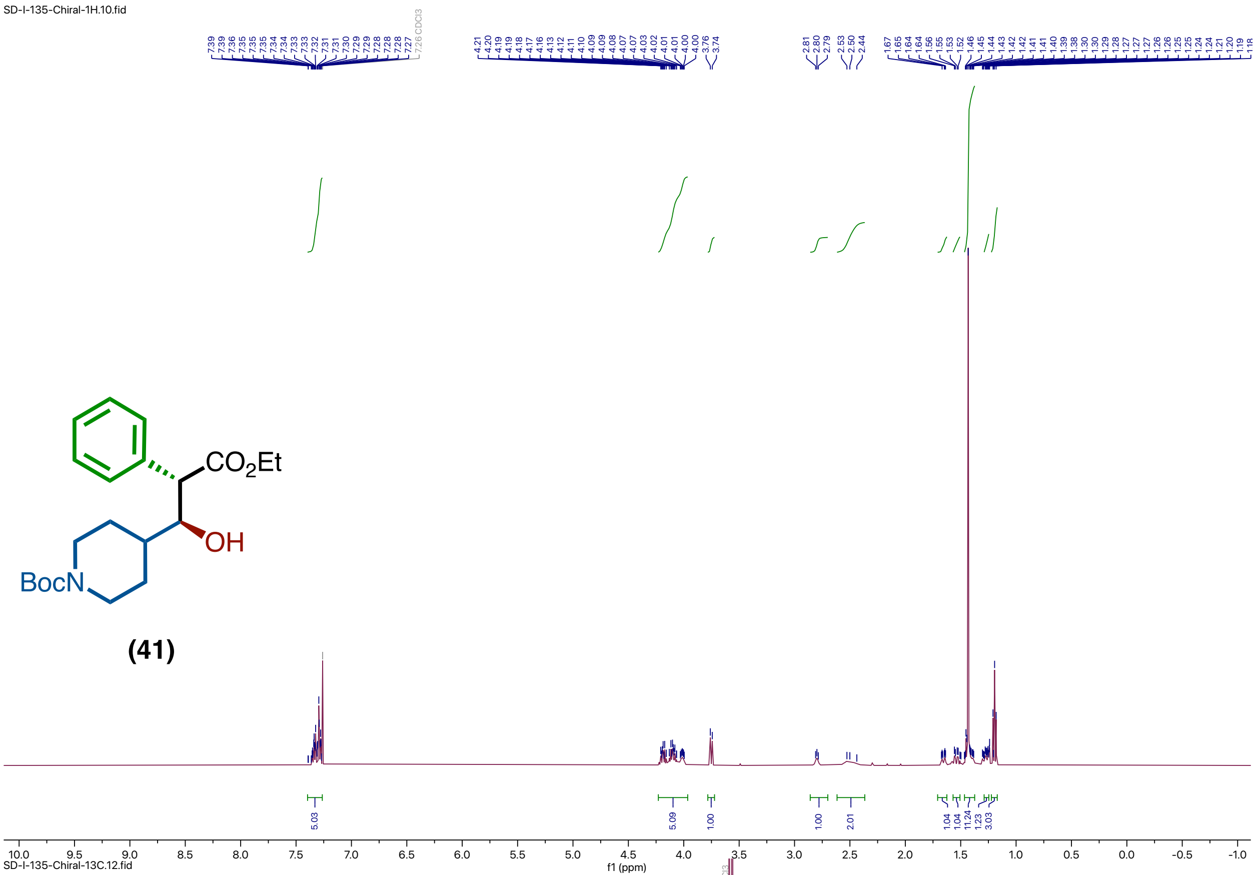

SD-I-135-Chiral-13C.12.fid

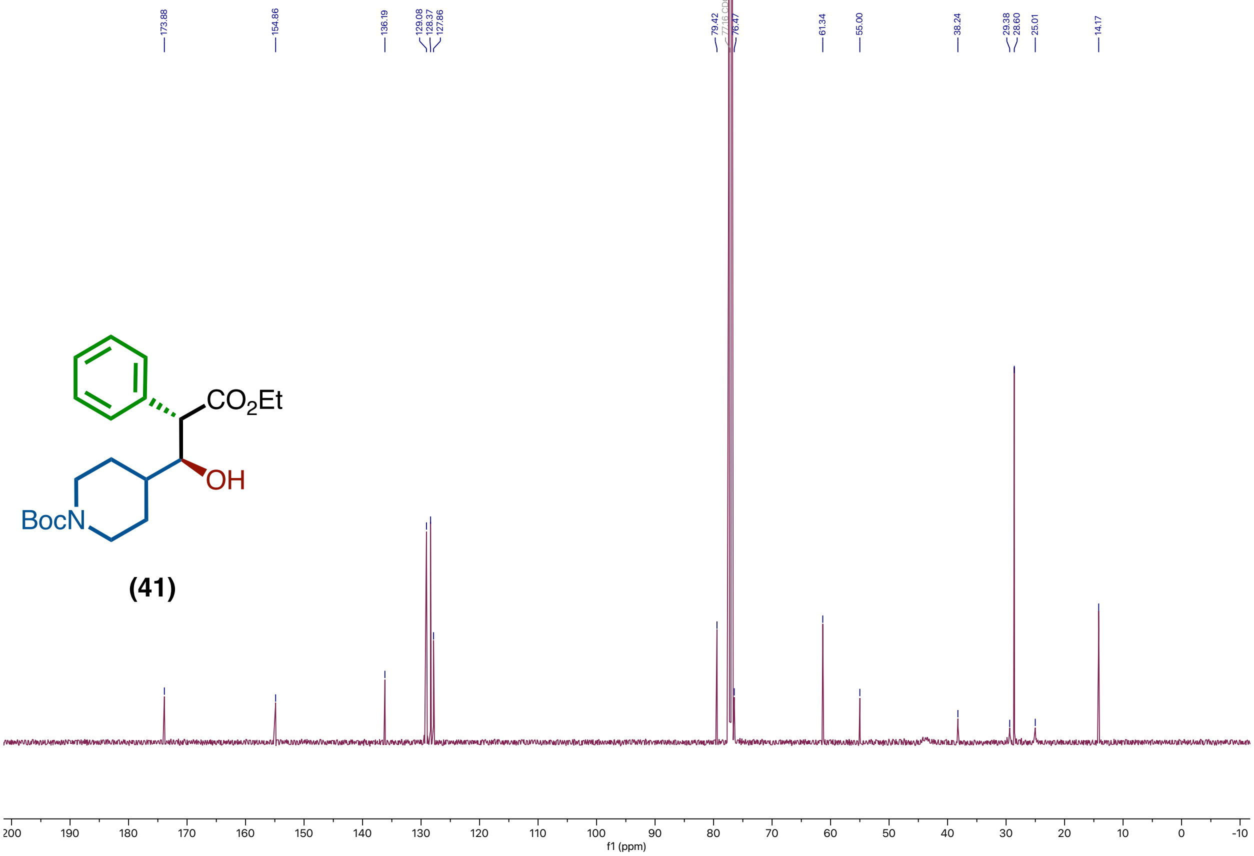

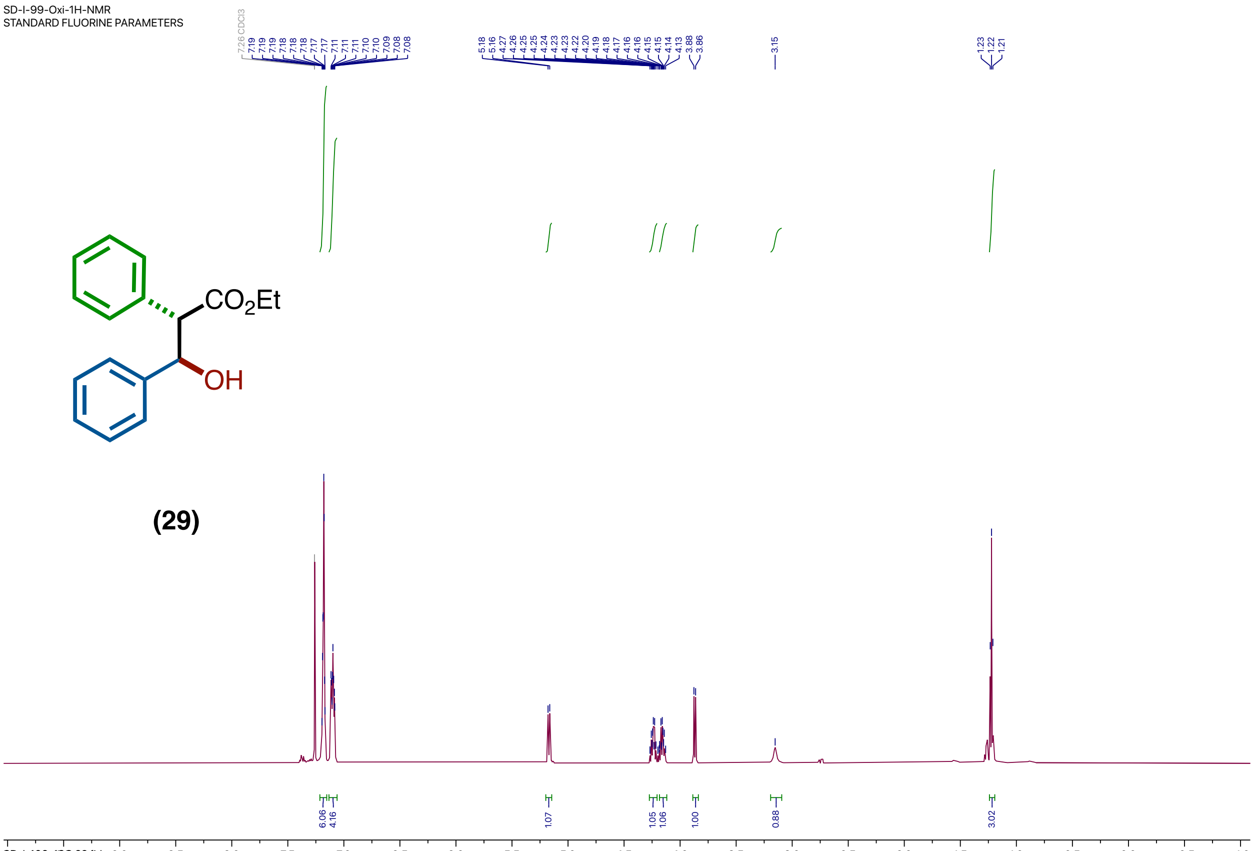
SD-I-99-Oxi-1H-NMR
STANDARD FLUORINE PARAMETERS
CO2Et
OH
(29)
f1 (ppm)

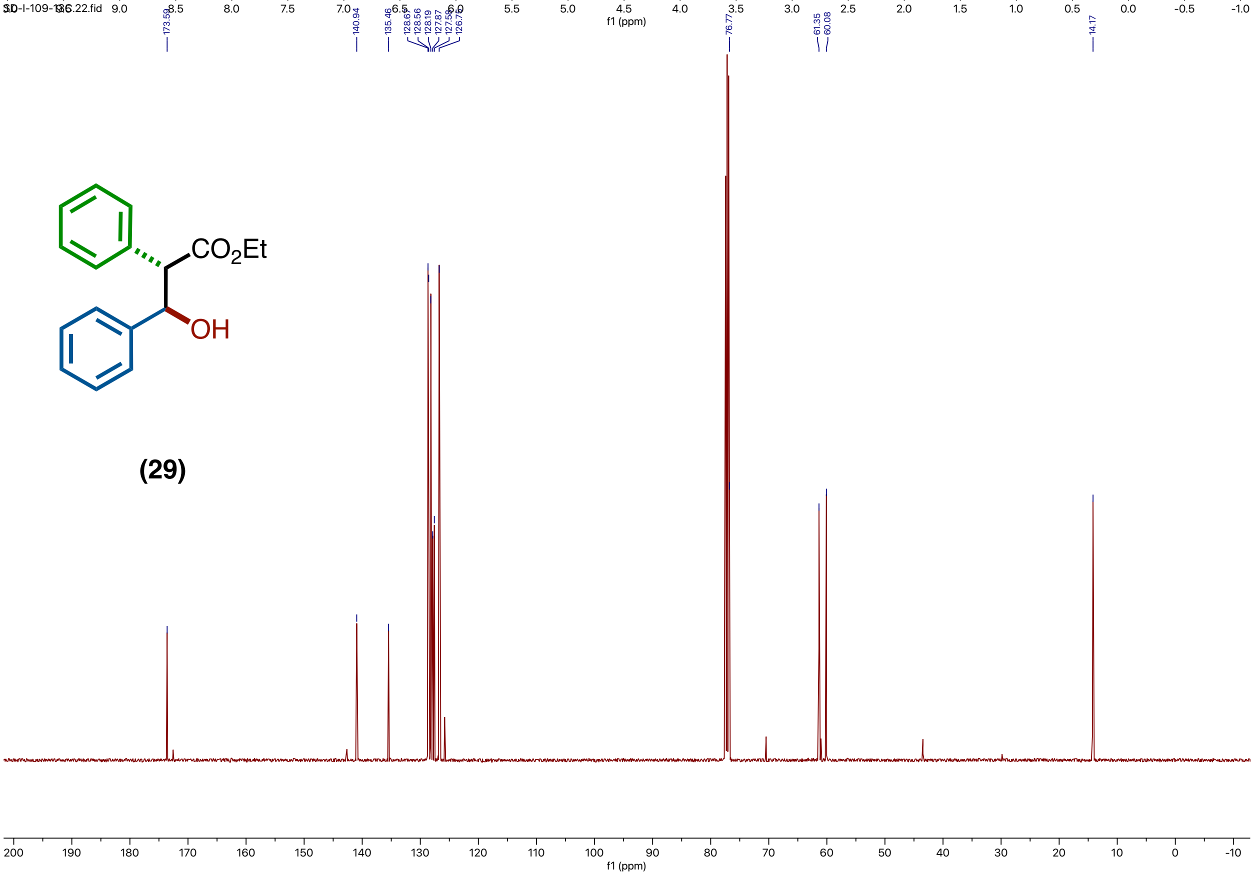
SD-I-109-13C.22.fid
CO2Et
OH
(29)
f1 (ppm)

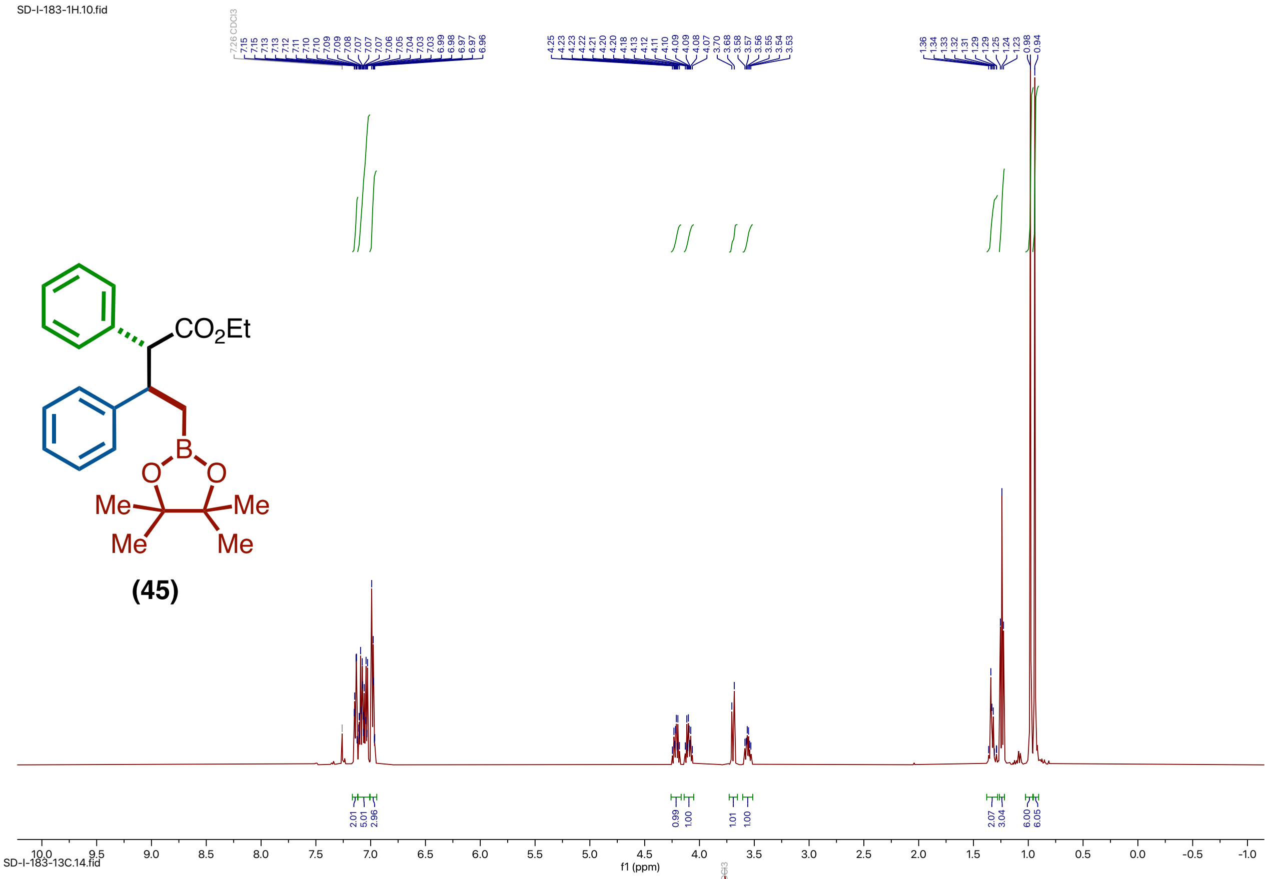
SD-I-183-1H.10.fid
7.26 CDCl3
7.15 7.15 7.13 7.12 7.11 7.10 7.10 7.09 7.09 7.08 7.07 7.07 7.07 7.06 7.05 7.04 7.03 7.03 6.99 6.98 6.97 6.96
4.25 4.23 4.23 4.22 4.21 4.20 4.20 4.18 4.13 4.12 4.11 4.10 4.09 4.09 4.08 4.07 3.70 3.68 3.58 3.57 3.56 3.55 3.54 3.53
1.36 1.34 1.33 1.32 1.31 1.29 1.29 1.25 1.24 1.23 0.98 0.94
CO2Et
B
O
O
Me
Me
Me
Me
(45)
2.01 5.01 2.96
0.99 1.00
1.01 1.00
2.07 3.04
6.00 6.05
f1 (ppm)

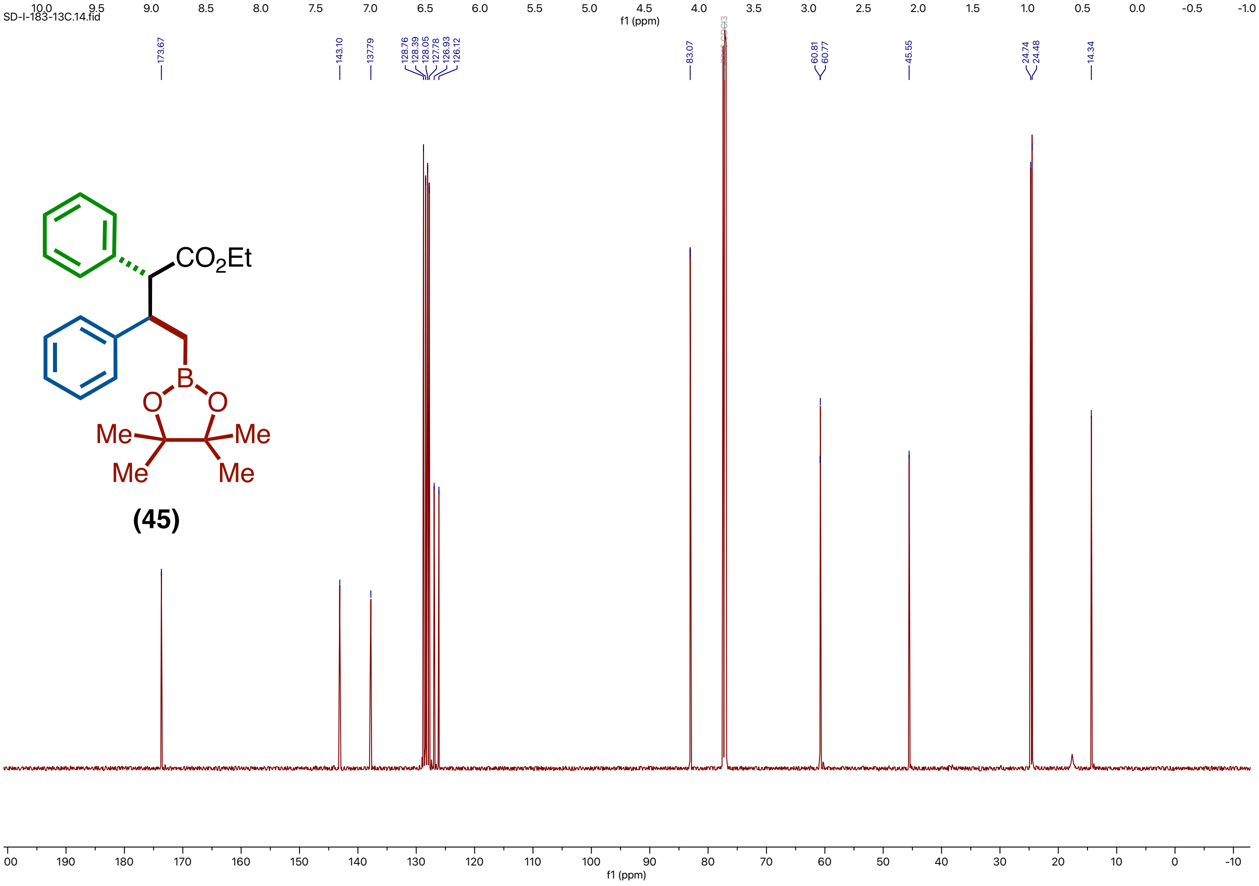
SD-I-183-13C.14.fid
173.67
143.10
137.79
128.76 128.39 128.05 127.78 126.93 126.12
83.07
60.81 60.77
45.55
24.74 24.48
14.34
CO2Et
B
O
O
Me
Me
Me
Me
(45)
f1 (ppm)

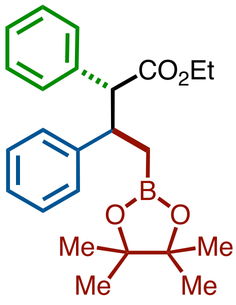

CO2Et
B
O
O
Me
Me
Me
Me

(45)

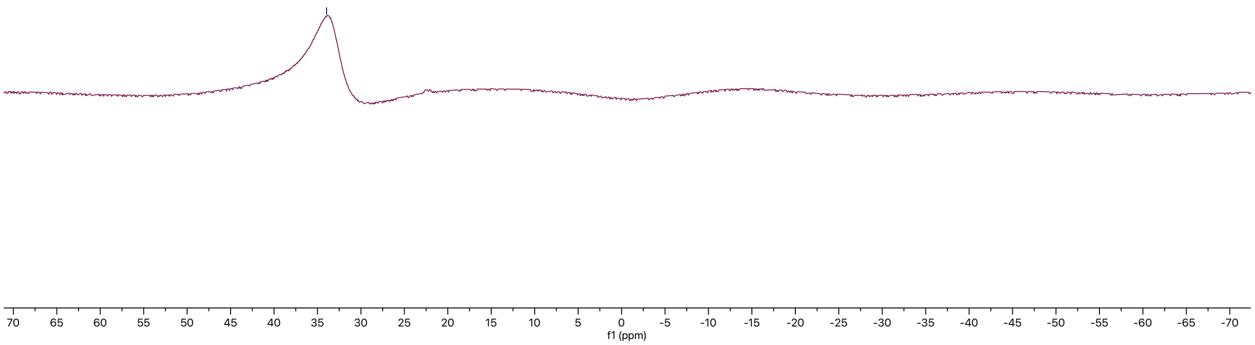

SD-I-183-11B-.10.fid
33.95
70 65 60 55 50 45 40 35 30 25 20 15 10 5 0 -5 -10 -15 -20 -25 -30 -35 -40 -45 -50 -55 -60 -65 -70
f1 (ppm)

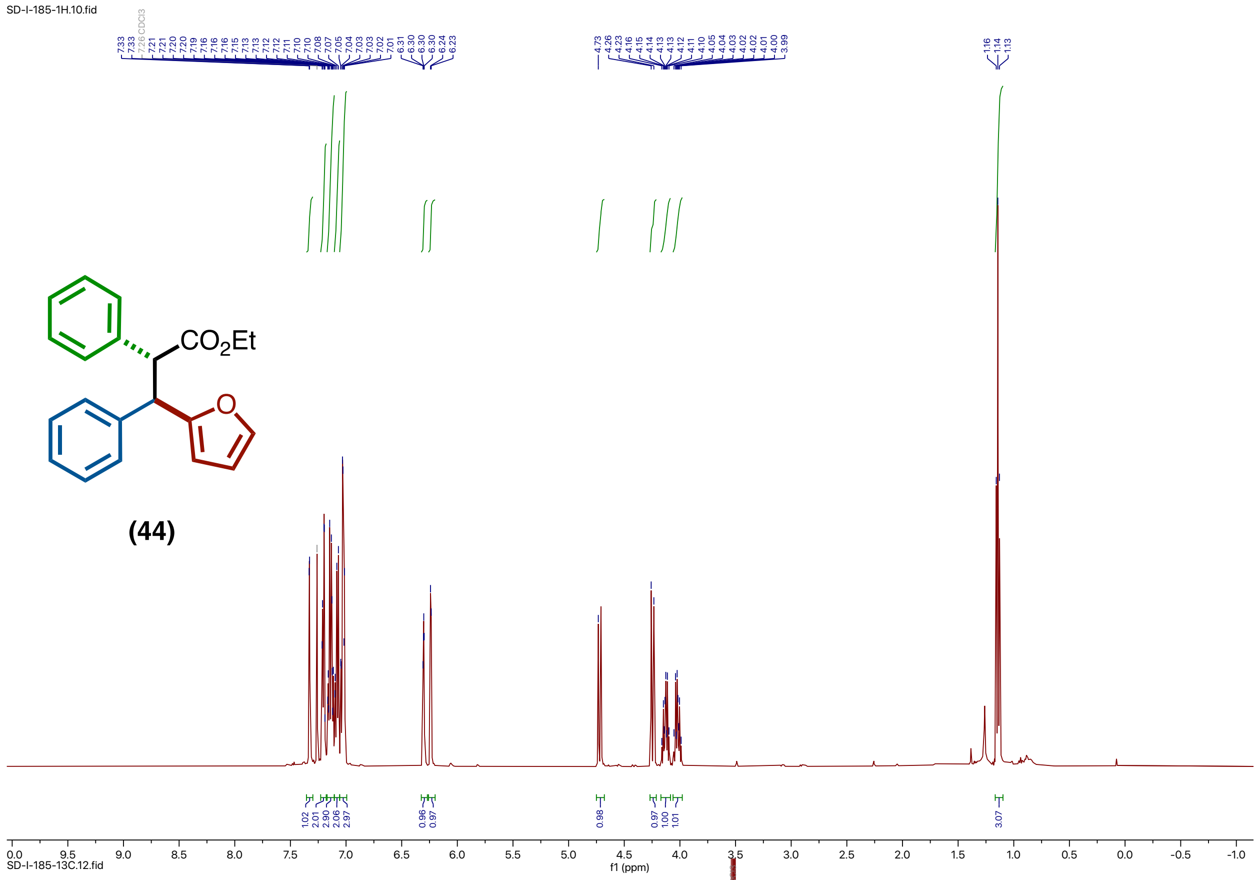
SD-I-185-1H.10.fid
7.33
7.33
7.26 CDCl3
7.21
7.21
7.20
7.20
7.19
7.16
7.16
7.16
7.15
7.13
7.13
7.12
7.12
7.11
7.10
7.10
7.08
7.07
7.05
7.04
7.03
7.03
7.02
7.01
6.31
6.30
6.30
6.30
6.24
6.23
4.73
4.26
4.23
4.16
4.15
4.14
4.13
4.13
4.12
4.11
4.10
4.05
4.04
4.03
4.03
4.02
4.02
4.01
4.00
3.99
1.16
1.14
1.13
CO2Et
O
(44)
1.02
2.01
2.90
2.06
2.97
0.96
0.97
0.98
0.97
1.00
1.01
3.07
f1 (ppm)

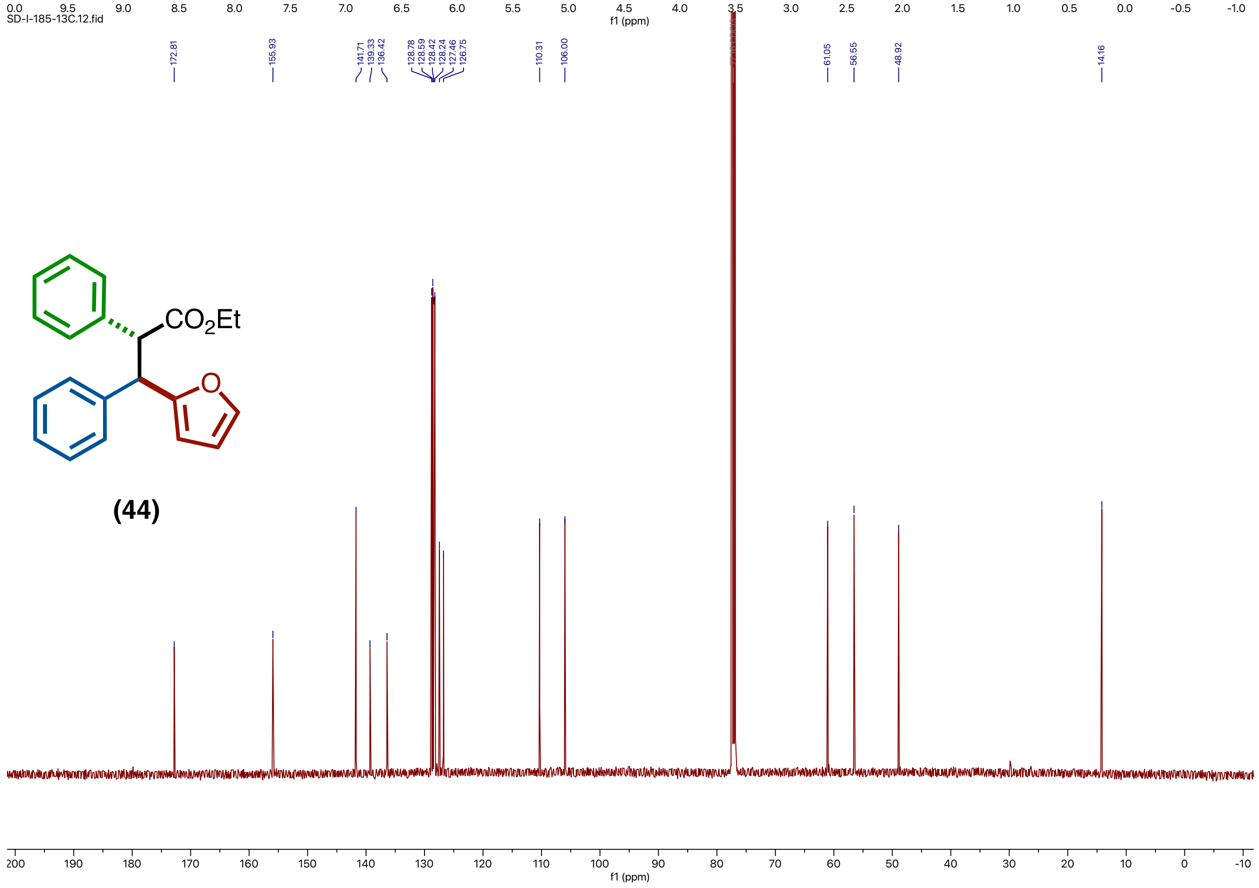
SD-I-185-13C.12.fid
172.81
155.93
141.71
139.33
136.42
128.78
128.59
128.42
128.24
127.46
126.75
110.31
106.00
61.05
56.55
48.92
14.16
CO2Et
O
(44)
f1 (ppm)

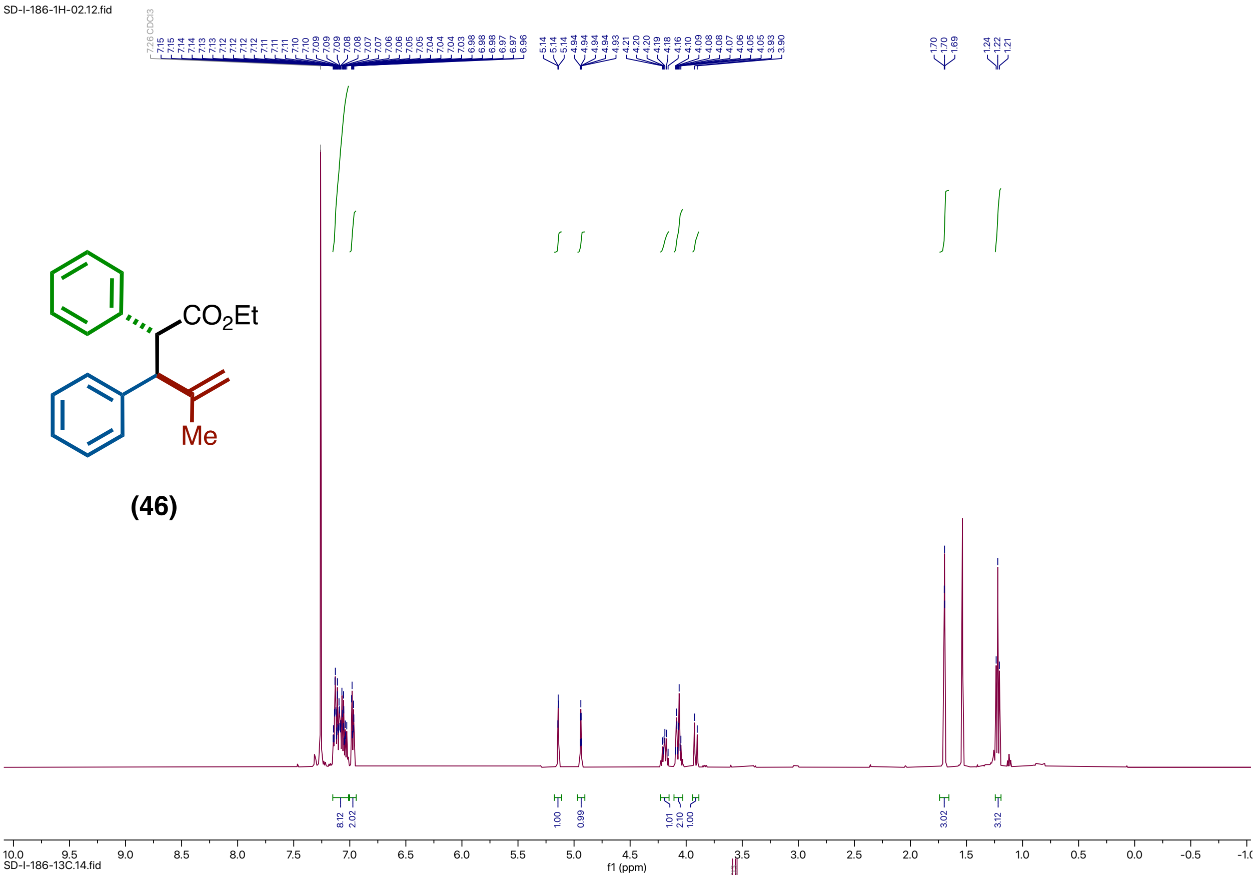
SD-I-186-1H-02.12.fid
7.26 CDCl3
7.15 7.14 7.14 7.13 7.12 7.12 7.12 7.12 7.11 7.11 7.11 7.10 7.09 7.09 7.09 7.08 7.08 7.07 7.07 7.06 7.06 7.05 7.05 7.04 7.04 7.04 7.03 6.98 6.98 6.98 6.97 6.97 6.96
5.14 5.14 5.14 4.94 4.94 4.94 4.94 4.93
4.21 4.20 4.19 4.18 4.18 4.16 4.10 4.09 4.08 4.08 4.07 4.06 4.05 4.05 3.93 3.90
1.70 1.70 1.69
1.24 1.22 1.21
CO2Et
Me
(46)
8.12 2.02 1.00 0.99 1.01 2.10 1.00 3.02 3.12
f1 (ppm)

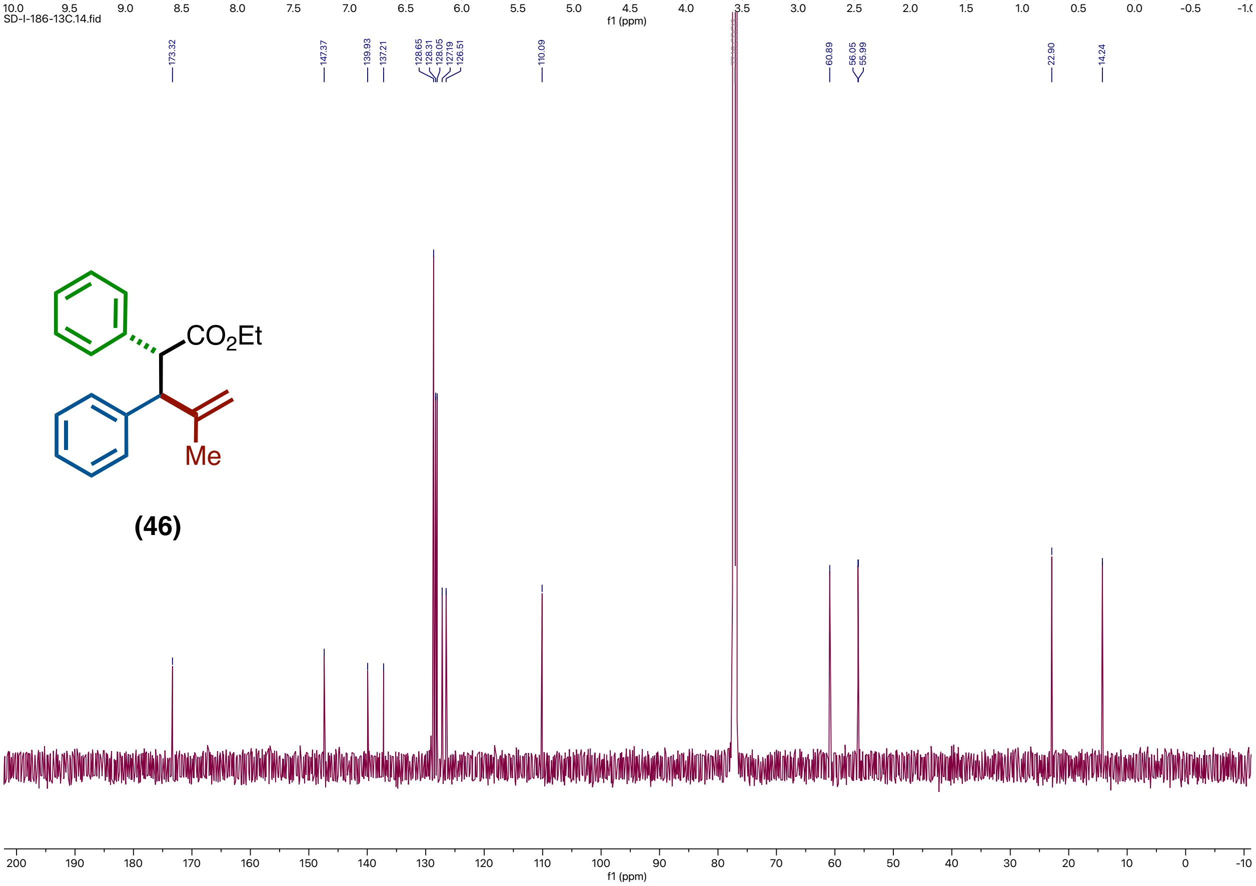
SD-I-186-13C.14.fid
173.32
147.37
139.93
137.21
128.65 128.31 128.05 127.09 126.51
110.09
60.89
56.05 55.99
22.90
14.24
CO2Et
Me
(46)
f1 (ppm)

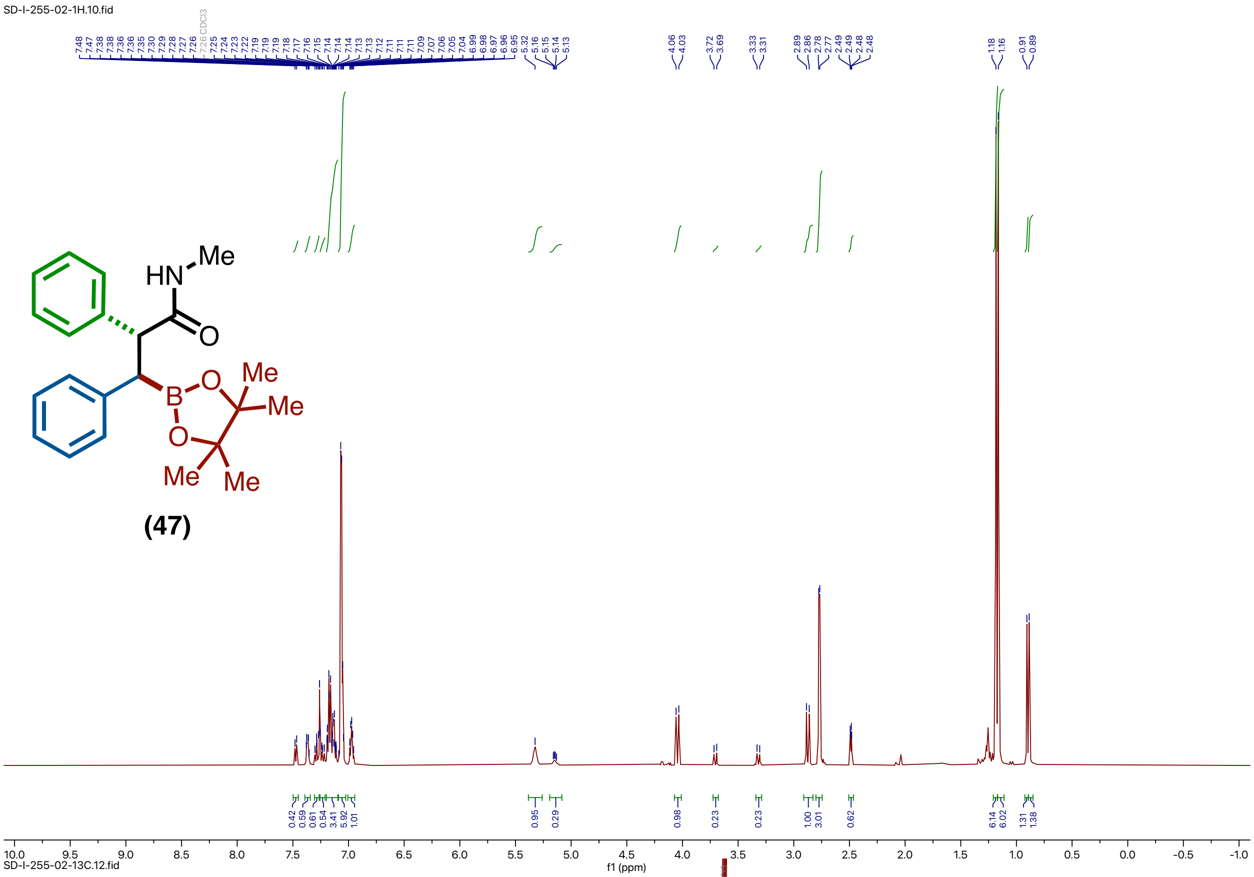
SD-I-255-02-1H.10.fid
HN
Me
O
B
O
O
Me
Me
Me
Me
(47)
f1 (ppm)

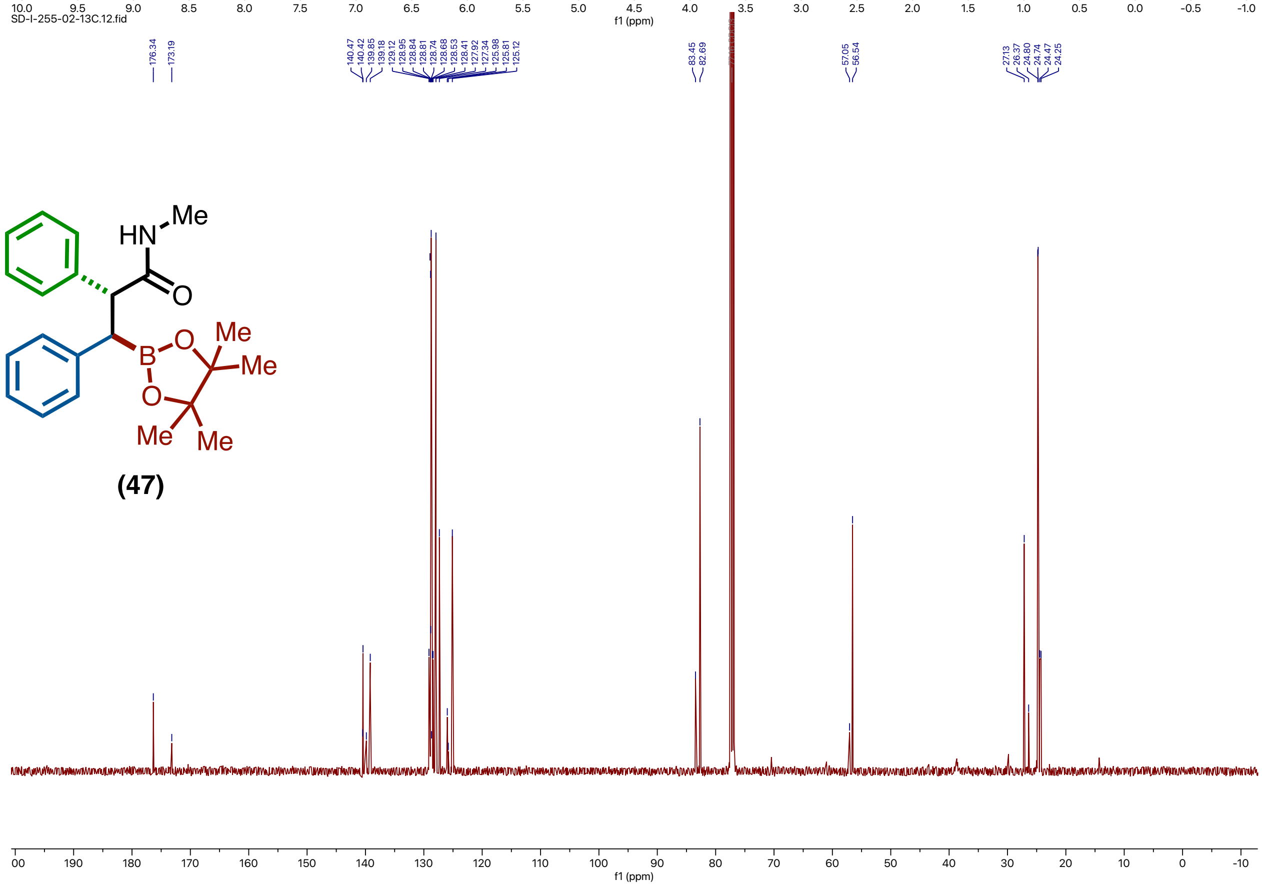
SD-I-255-02-13C.12.fid
HN
Me
O
B
O
O
Me
Me
Me
Me
(47)
f1 (ppm)

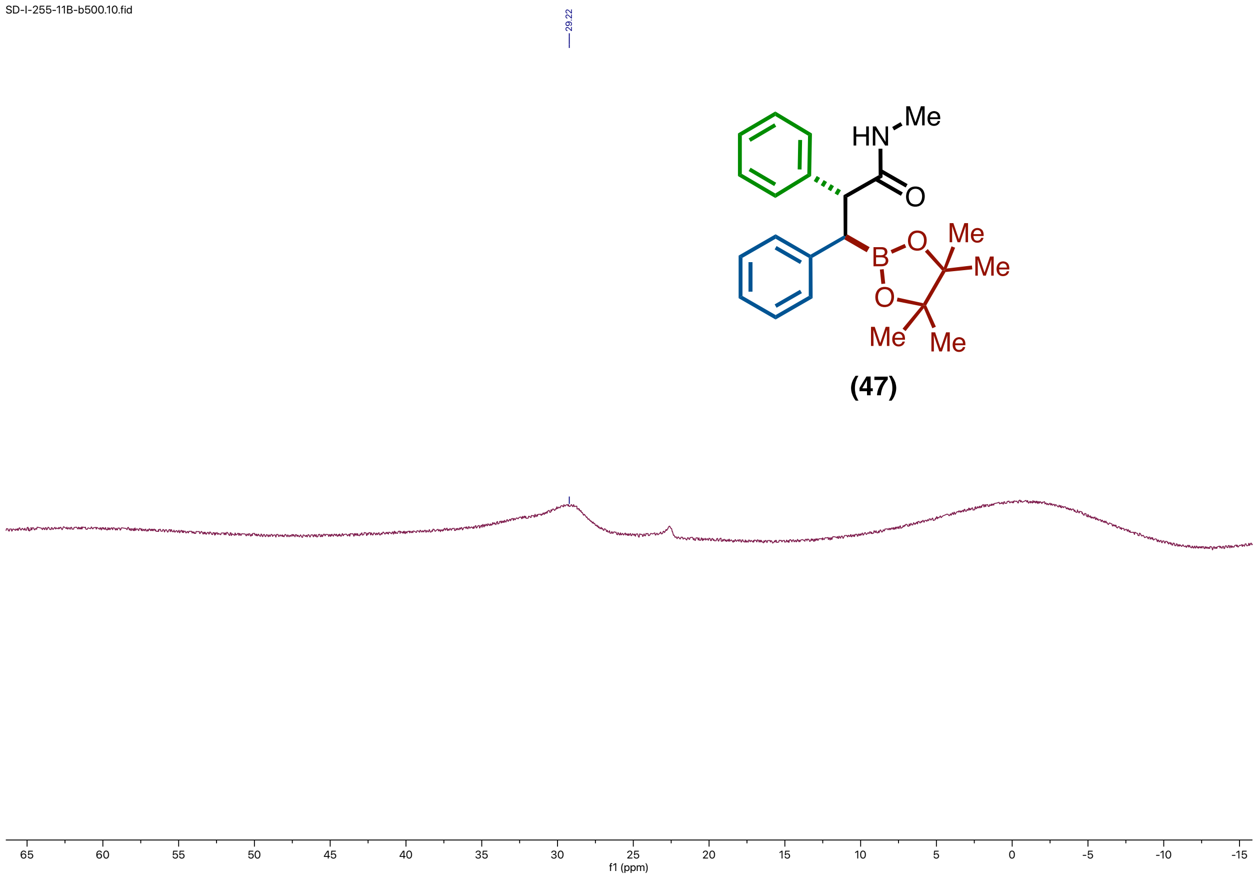
SD-I-255-11B-b500.10.fid
29.22
HN
Me
O
B
O
O
Me
Me
Me
Me
(47)
65
60
55
50
45
40
35
30
25
20
15
10
5
0
-5
-10
-15
f1 (ppm)

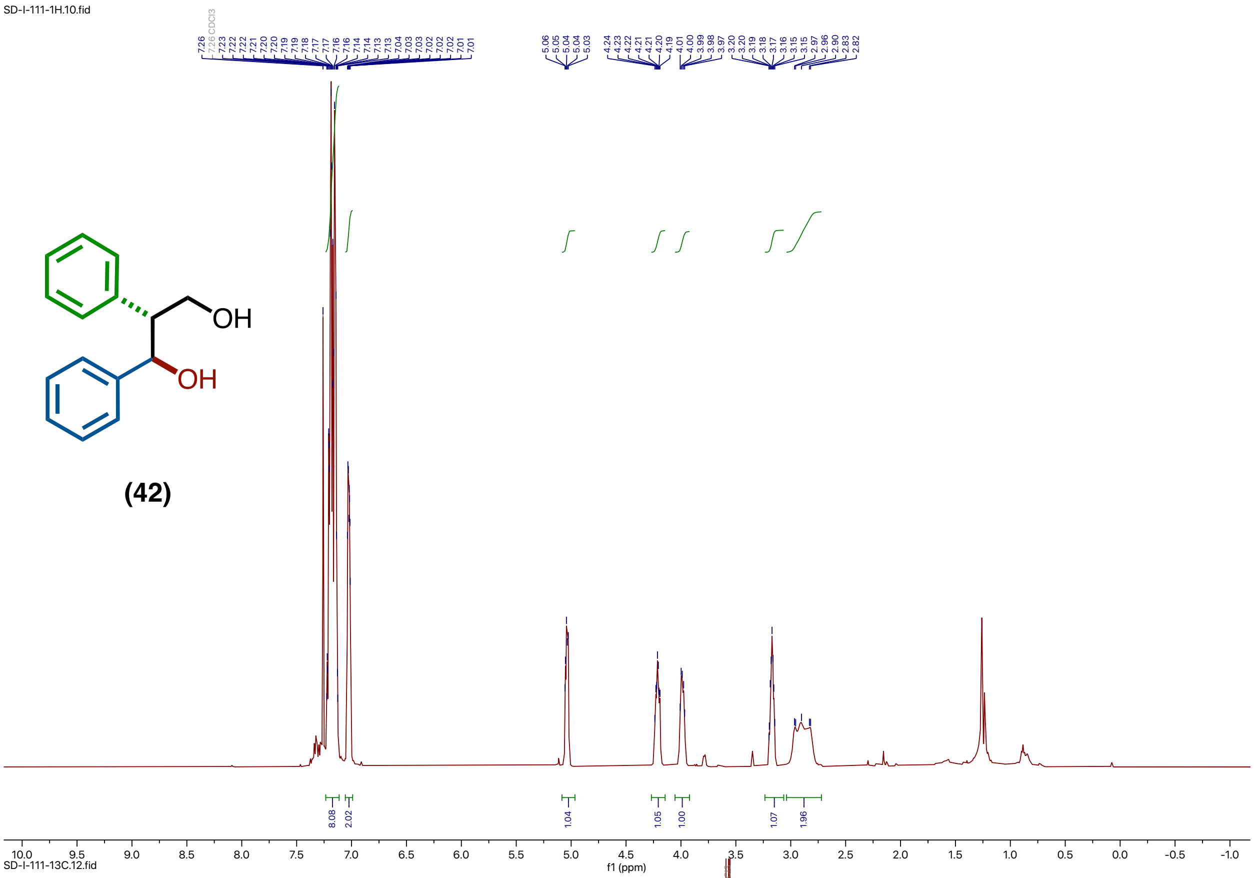
SD-I-111-1H.10.fid
7.26
7.26 CDCl3
7.23
7.22
7.22
7.21
7.20
7.20
7.19
7.19
7.18
7.17
7.17
7.16
7.16
7.14
7.14
7.13
7.13
7.04
7.03
7.03
7.02
7.02
7.02
7.01
7.01
5.06
5.05
5.04
5.04
5.03
4.24
4.23
4.22
4.21
4.21
4.20
4.19
4.01
4.00
3.99
3.98
3.97
3.20
3.20
3.19
3.18
3.17
3.16
3.15
3.15
2.97
2.96
2.90
2.83
2.82
OH
OH
(42)
8.08
2.02
1.04
1.05
1.00
1.07
1.96
f1 (ppm)

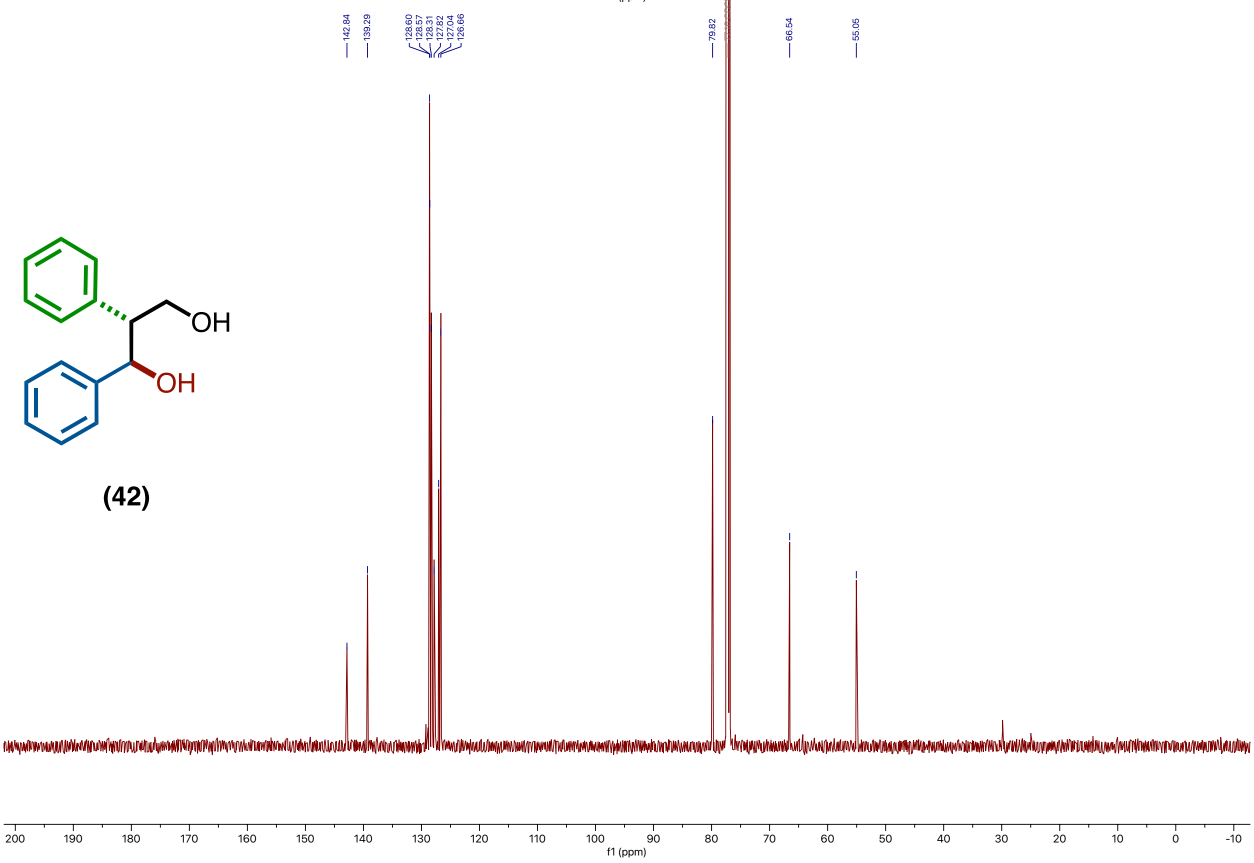
SD-I-111-13C.12.fid
142.84
139.29
128.60
128.57
128.31
127.82
127.04
126.66
79.82
66.54
55.05
OH
OH
(42)
f1 (ppm)

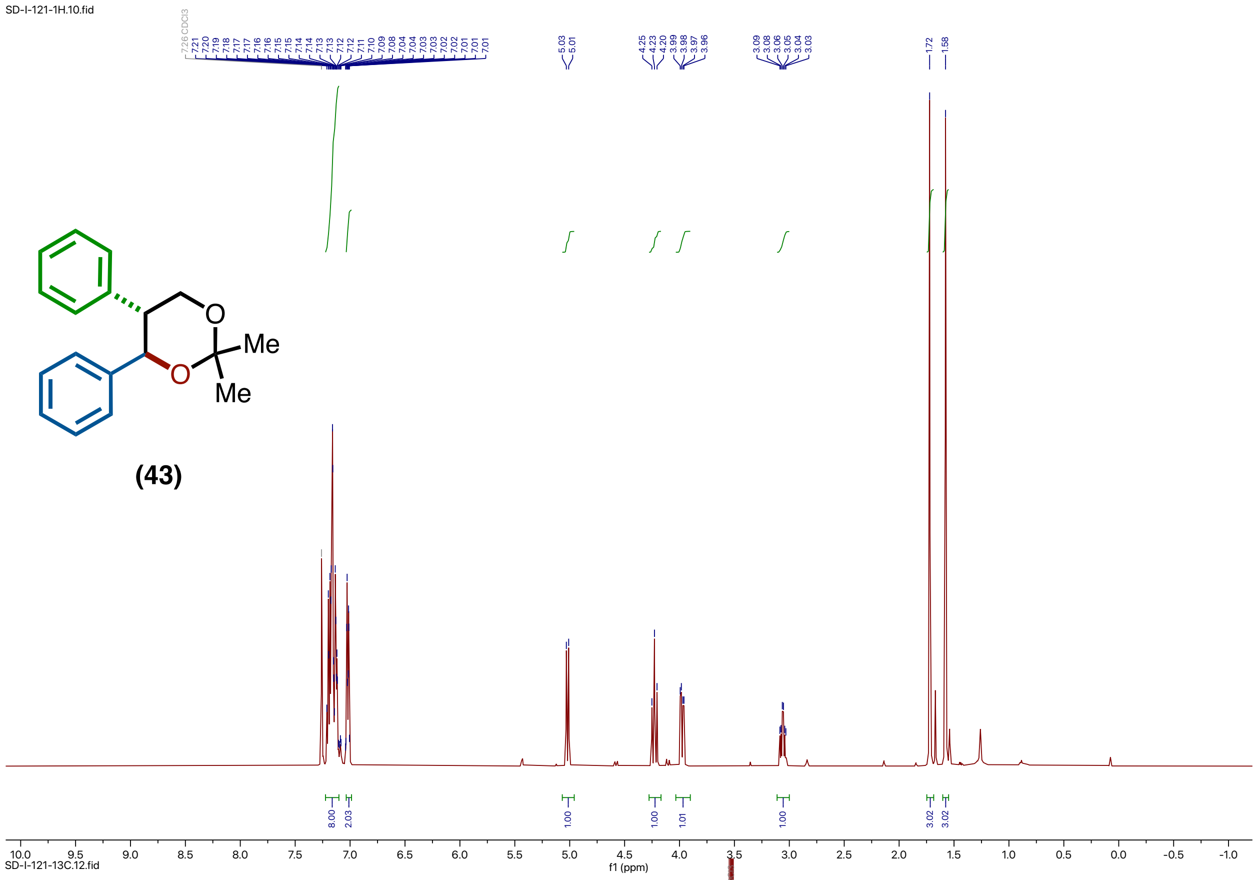
SD-I-121-1H.10.fid
7.26 CDCl3
7.21
7.20
7.19
7.18
7.17
7.17
7.16
7.16
7.15
7.15
7.14
7.14
7.13
7.13
7.12
7.12
7.11
7.10
7.09
7.08
7.04
7.04
7.03
7.03
7.02
7.02
7.01
7.01
7.01
5.03
5.01
4.25
4.23
4.20
3.99
3.98
3.97
3.96
3.09
3.08
3.06
3.05
3.04
3.03
1.72
1.58
O
Me
O
Me
(43)
8.00
2.03
1.00
1.00
1.01
1.00
3.02
3.02
f1 (ppm)

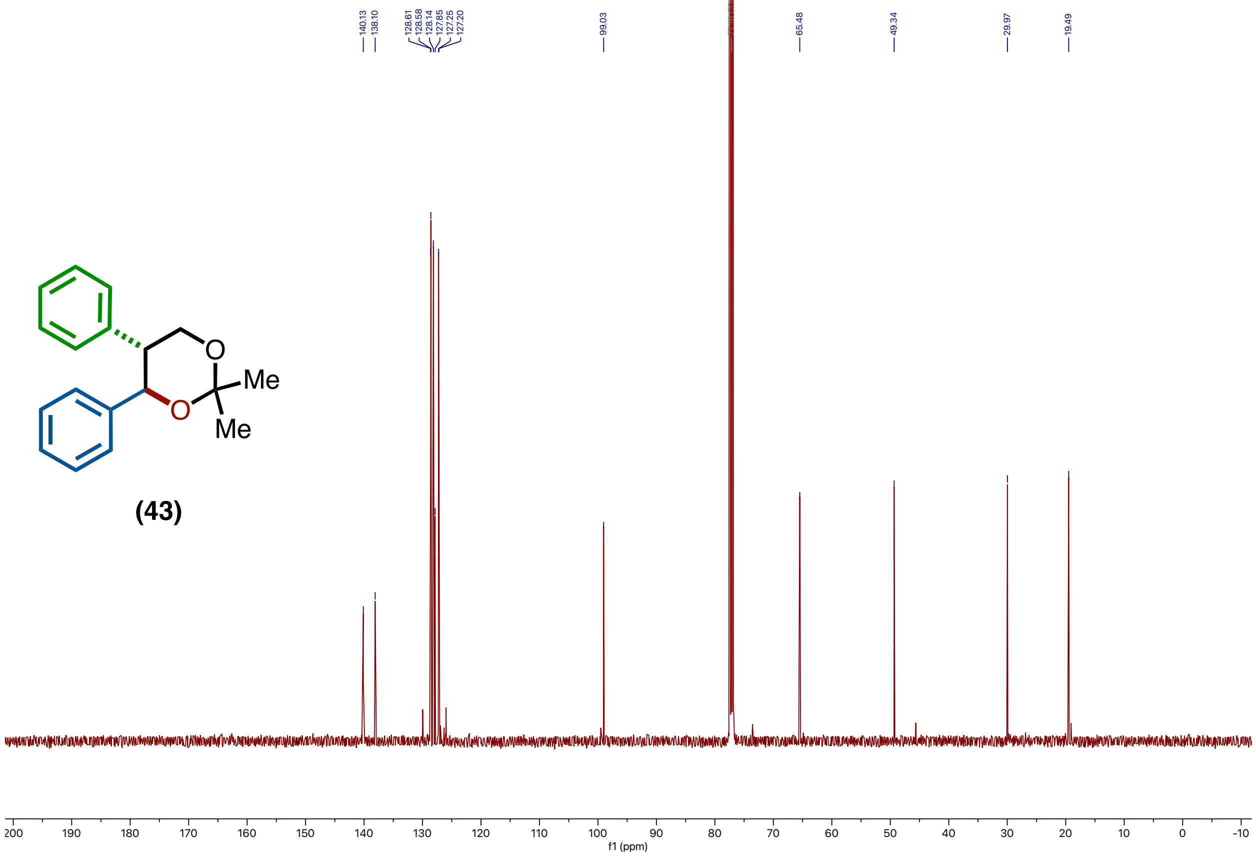
SD-I-121-13C.12.fid
140.13
138.10
128.61
128.58
128.14
127.85
127.25
127.20
99.03
65.48
49.34
29.97
19.49
O
Me
O
Me
(43)
f1 (ppm)

12. HPLC data:

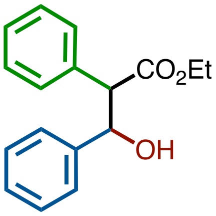

(*racemic-2*)

```
Sample Info      : C2_95:5-Hex:IPA_1 ml/min_220 nm

Additional Info : Peak(s) manually integrated
```

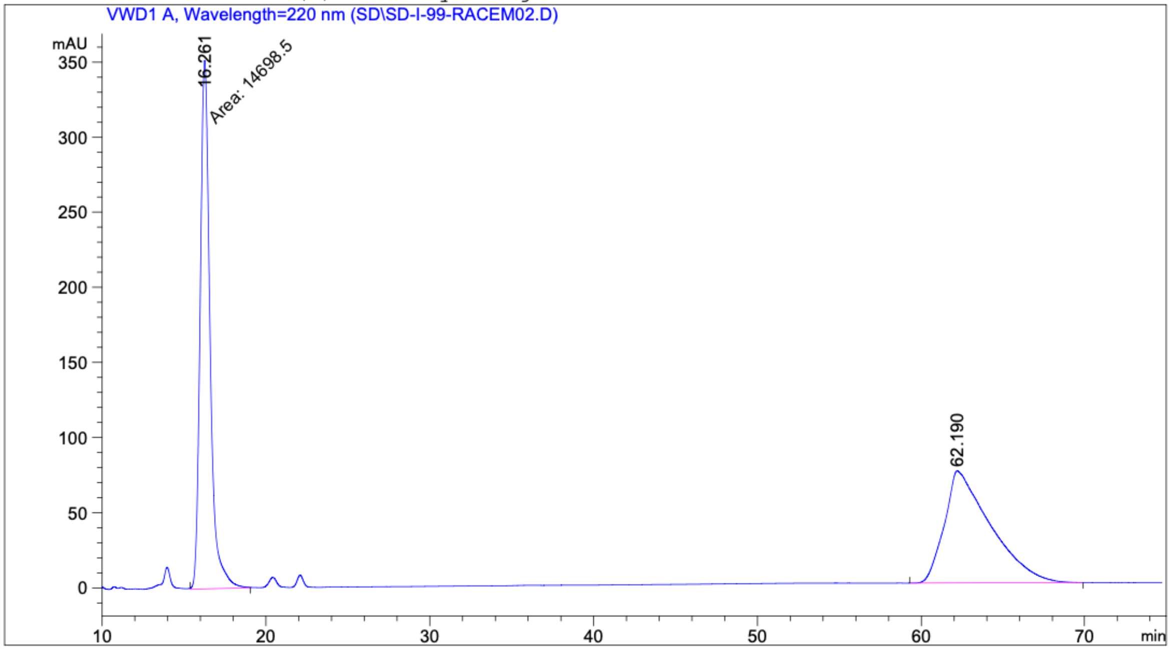


```
=====================================================================
                         Area Percent Report
=====================================================================

Sorted By             :      Signal
Multiplier:                    :      1.0000
Dilution:                      :      1.0000
Do not use Multiplier & Dilution Factor with ISTDs


Signal 1: VWD1 A, Wavelength=220 nm

Peak RetTime Type  Width     Area      Height     Area
  #   [min]         [min]   [mAU*s]     [mAU]      %
----|-------|----|-------|----------|----------|--------|
   1  16.261 MM    0.6962 1.46985e4  351.85062  50.2713
   2  62.190 BB    2.4348 1.45399e4   74.53826  49.7287

Totals :                  2.92384e4  426.38888
```

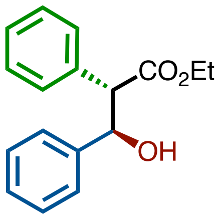

(enantioenriched-2)

```
Sample Info      : C2_95:5-Hex:IPA_1 ml/min_220 nm


Additional Info : Peak(s) manually integrated
```

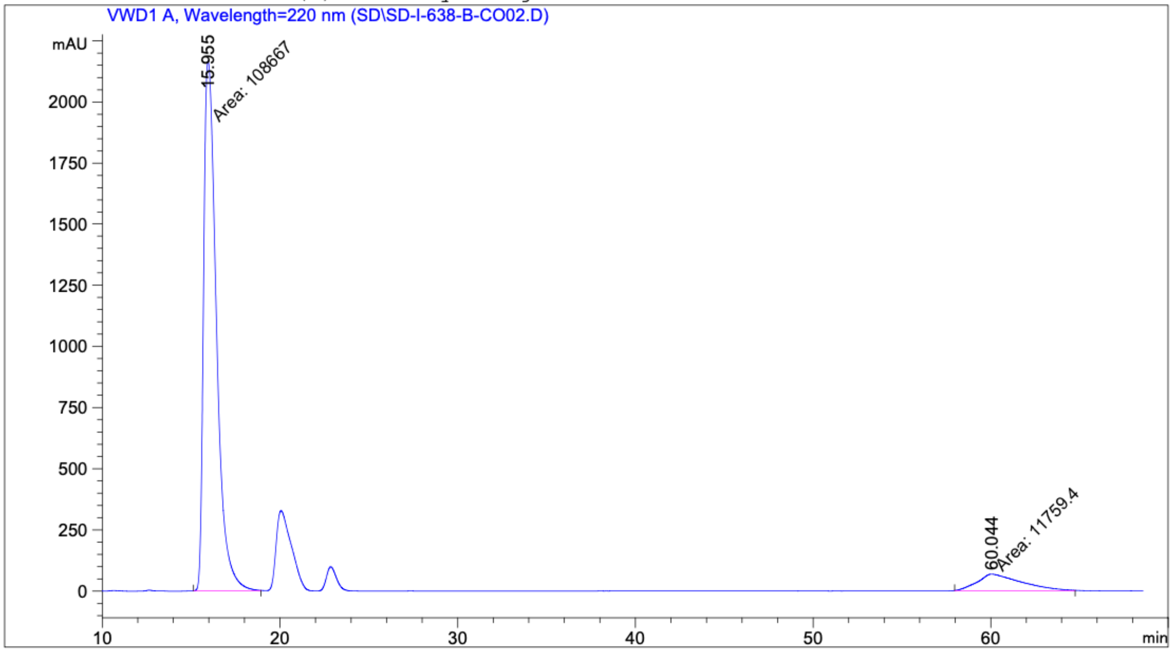


```
=====================================================================
                         Area Percent Report
=====================================================================

Sorted By             :      Signal
Multiplier:                   :      1.0000
Dilution:                     :      1.0000
Do not use Multiplier & Dilution Factor with ISTDs


Signal 1: VWD1 A, Wavelength=220 nm

Peak RetTime Type  Width     Area      Height     Area
  #   [min]        [min]   [mAU*s]     [mAU]        %
----|-------|----|-------|----------|----------|--------|
   1  15.955 MM    0.8360 1.08667e5  2166.42358  90.2352
   2  60.044 MM    2.8868 1.17594e4    67.89142   9.7648

Totals :                  1.20427e5  2234.31500
```

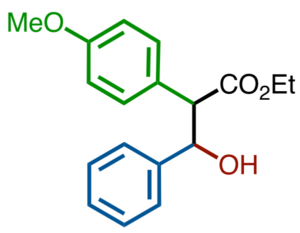

(*racemic-31*)

Sample Info : C1_90:10 Hex:IPA; 1mL/min, 220 nm

Additional Info : Peak(s) manually integrated

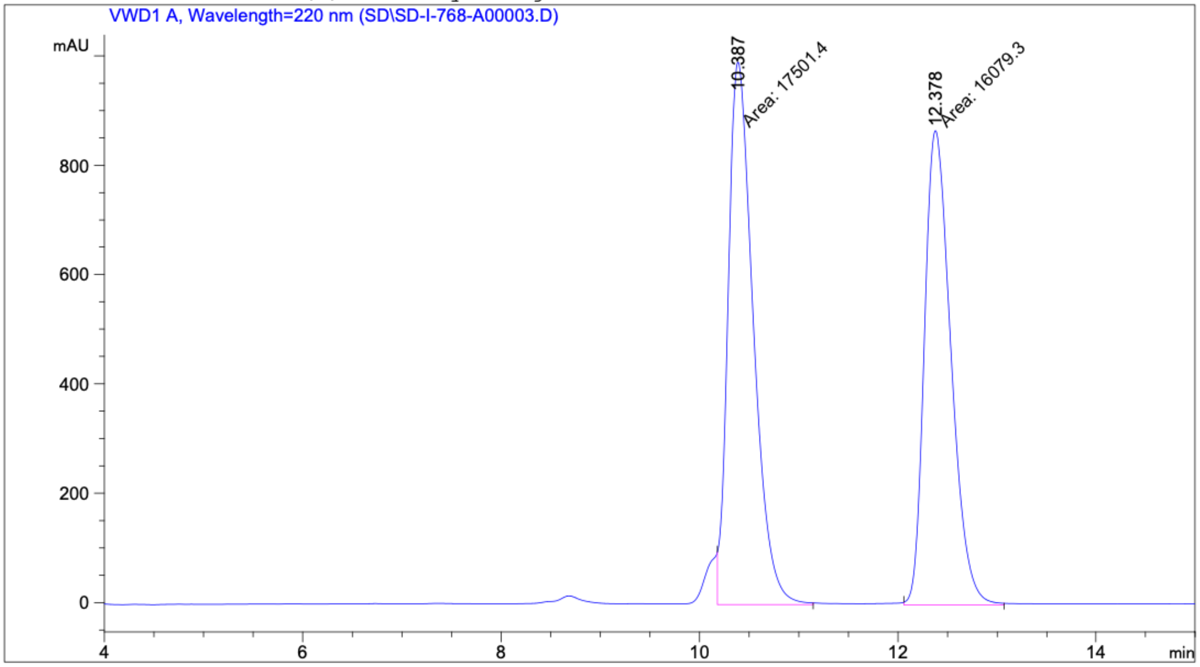

===

Area Percent Report

===

Sorted By : Signal
Multiplier: : 1.0000
Dilution: : 1.0000
Do not use Multiplier & Dilution Factor with ISTDs

Signal 1: VWD1 A, Wavelength=220 nm

Peak #	RetTime [min]	Type	Width [min]	Area [mAU*s]	Height [mAU]	Area %
1	10.387	FM	0.2940	1.75014e4	992.04059	52.1175
2	12.378	MM	0.3089	1.60793e4	867.48572	47.8825

Totals : 3.35807e4 1859.52631

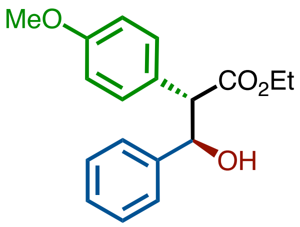

(*enantioenriched*-31)

```
Sample Info      : C1_90:10  Hex:IPA; 1mL/min, 220 nm


Additional Info : Peak(s) manually integrated
```

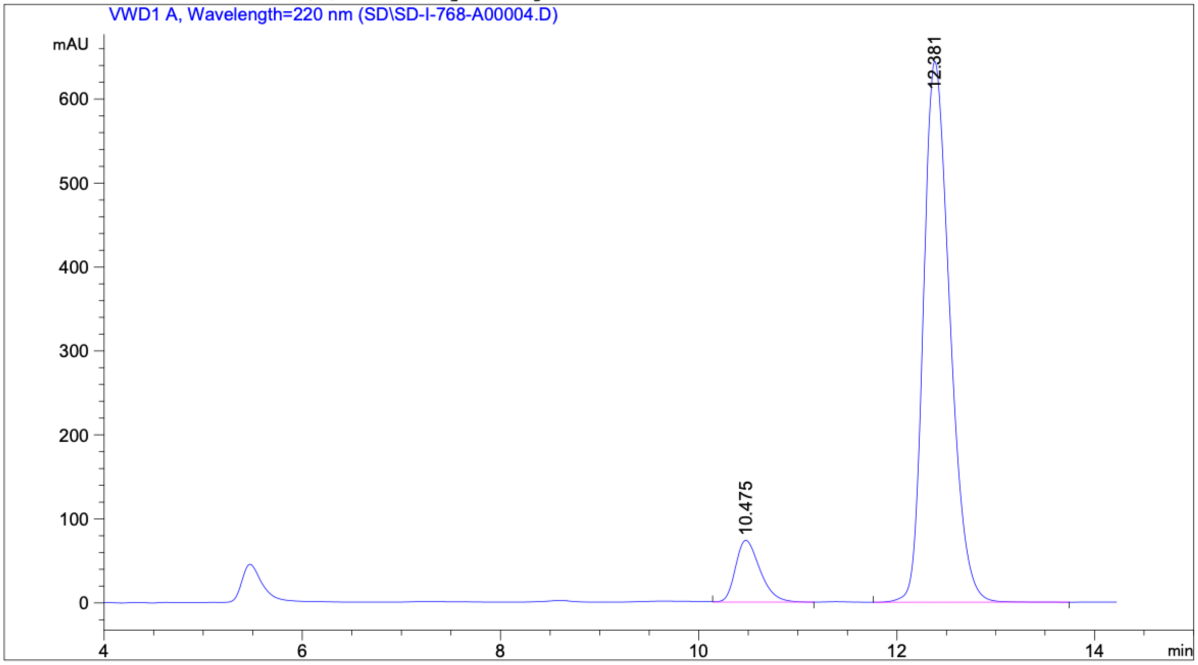


```
=====================================================================
                         Area Percent Report
=====================================================================

Sorted By             :      Signal
Multiplier:                   :      1.0000
Dilution:                     :      1.0000
Do not use Multiplier & Dilution Factor with ISTDs


Signal 1: VWD1 A, Wavelength=220 nm

Peak RetTime Type  Width     Area       Height     Area
  #   [min]        [min]   [mAU*s]      [mAU]        %
----|-------|----|-------|----------|----------|--------|
   1  10.475 VB    0.2642 1262.01343   73.56206   9.5853
   2  12.381 BB    0.2867 1.19041e4   643.98090  90.4147

Totals :                  1.31661e4   717.54295
```

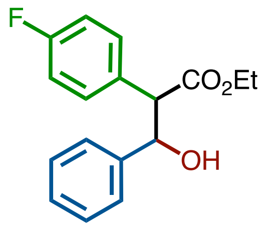

(*racemic-32*)

Sample Info : C-1_90:10_Hex:IPA;, 1mL/min, 220 nm

Additional Info : Peak(s) manually integrated

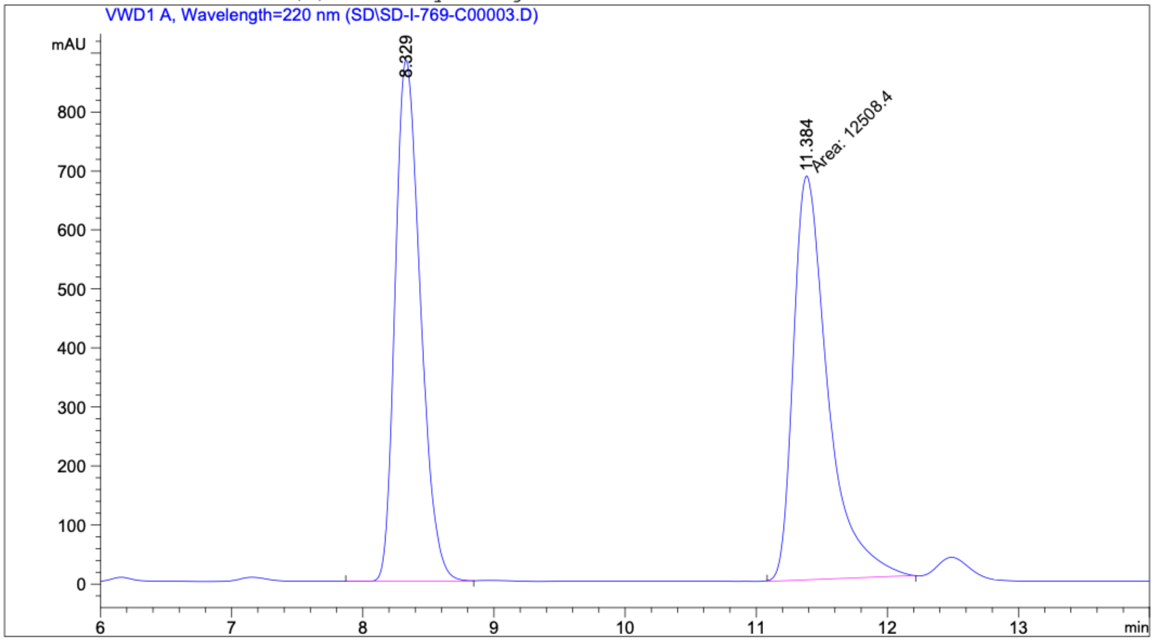

===

Area Percent Report

===

Sorted By : Signal
Multiplier: : 1.0000
Dilution: : 1.0000
Do not use Multiplier & Dilution Factor with ISTDs

Signal 1: VWD1 A, Wavelength=220 nm

Peak #	RetTime [min]	Type	Width [min]	Area [mAU*s]	Height [mAU]	Area %
1	8.329	BV	0.2082	1.18300e4	883.34167	48.6064
2	11.384	MM	0.3046	1.25084e4	684.50415	51.3936

Totals : 2.43384e4 1567.84583

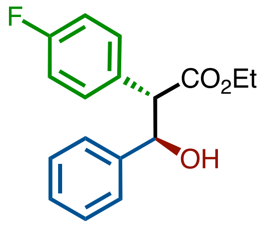

(*enantioenriched*-32)

Sample Info : C-1_90:10_Hex:IPA;, 1mL/min, 220 nm

Additional Info : Peak(s) manually integrated

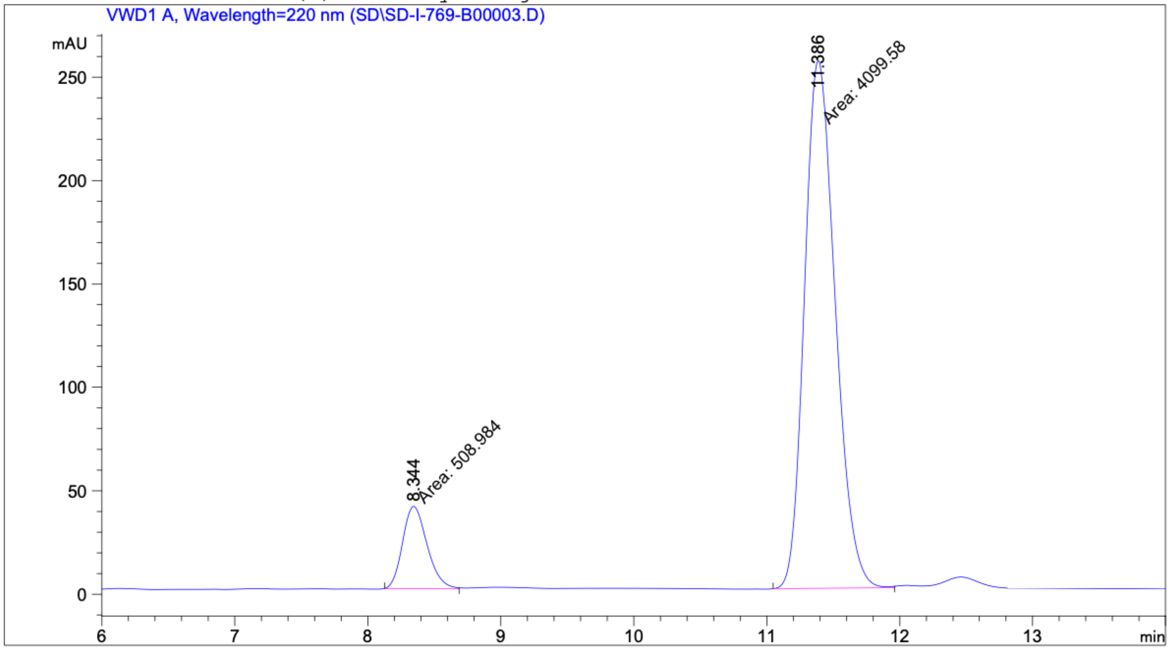

===

Area Percent Report

===

Sorted By : Signal
Multiplier: : 1.0000
Dilution: : 1.0000
Do not use Multiplier & Dilution Factor with ISTDs

Signal 1: VWD1 A, Wavelength=220 nm

Peak #	RetTime [min]	Type	Width [min]	Area [mAU*s]	Height [mAU]	Area %
1	8.344	MM	0.2132	508.98373	39.78003	11.0443
2	11.386	MM	0.2678	4099.57568	255.10527	88.9557

Totals : 4608.55942 294.88530

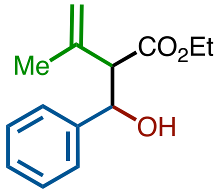

(*racemic-33*)

```
Sample Info     : IA-3_95:5  Hex:IPA;, 1mL/min, 220 nm

Additional Info : Peak(s) manually integrated
```

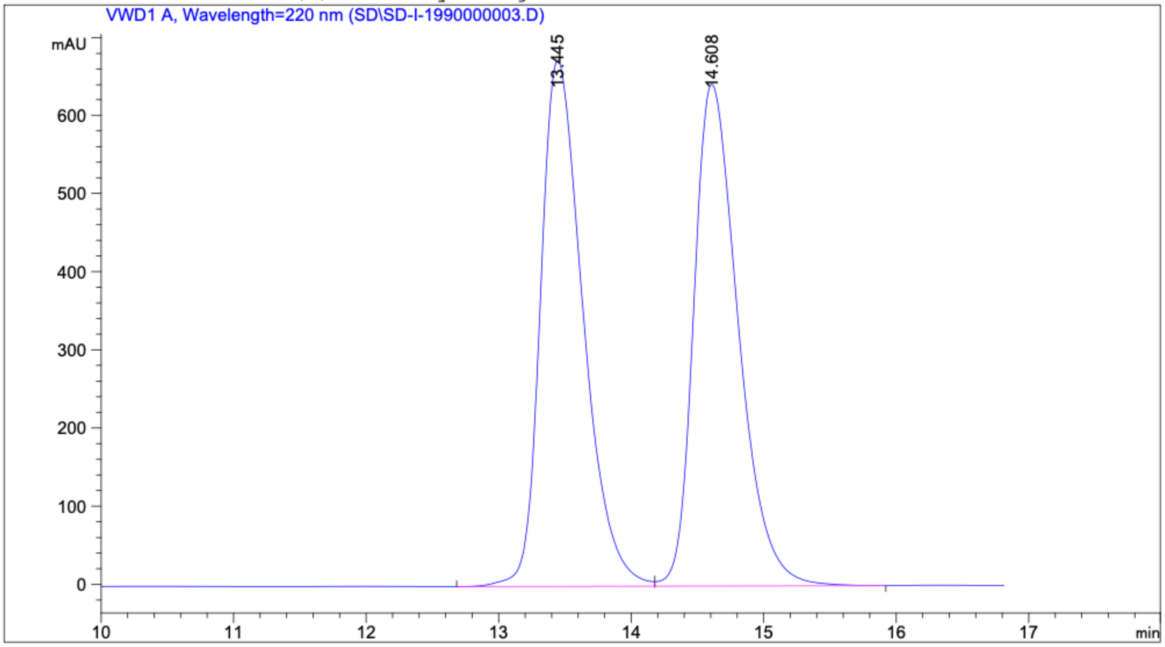


```
=====================================================================
                          Area Percent Report
=====================================================================

Sorted By             :      Signal
Multiplier:                   :      1.0000
Dilution:                     :      1.0000
Do not use Multiplier & Dilution Factor with ISTDs


Signal 1: VWD1 A, Wavelength=220 nm

Peak RetTime Type  Width     Area       Height     Area
  #   [min]        [min]   [mAU*s]      [mAU]        %
----|-------|----|-------|----------|----------|--------|
   1  13.445 BV    0.3423 1.49725e4   673.62817  50.0128
   2  14.608 VB    0.3596 1.49649e4   642.82092  49.9872

Totals :                  2.99373e4  1316.44910
```

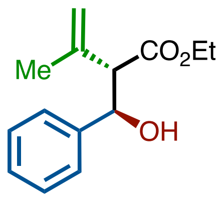

(*enantioenriched*-33)

Sample Info : IA-3_95:5_Hex:IPA_1.0 mL/min_220 nm

Additional Info : Peak(s) manually integrated

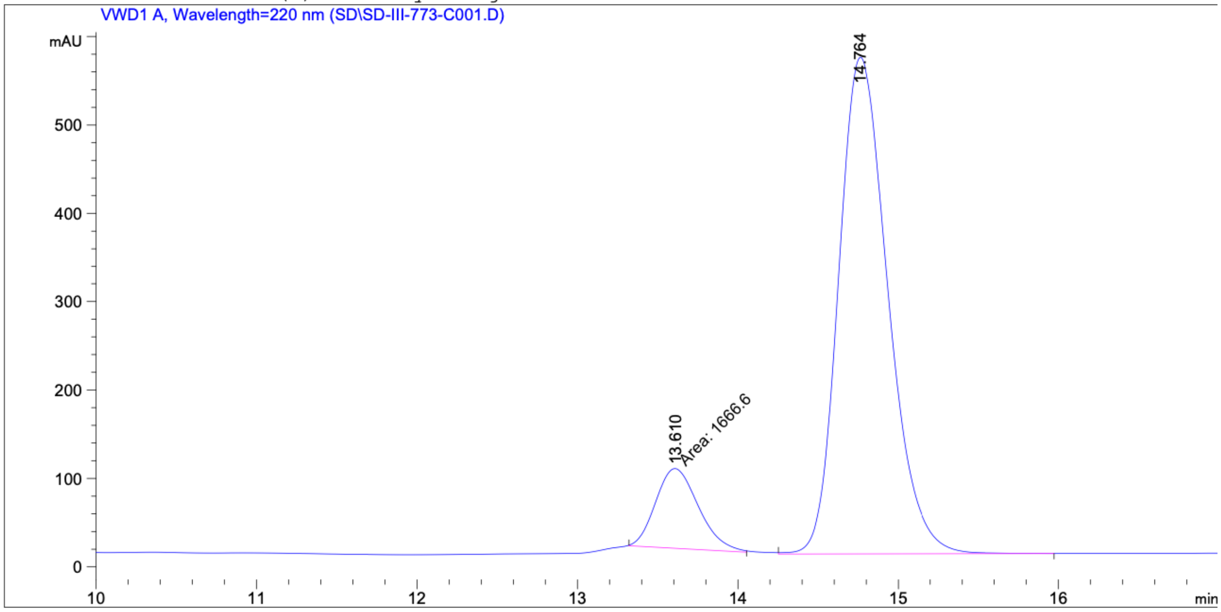

===
Area Percent Report
===

Sorted By : Signal
Multiplier: : 1.0000
Dilution: : 1.0000
Use Multiplier & Dilution Factor with ISTDs

Signal 1: VWD1 A, Wavelength=220 nm

Peak #	RetTime [min]	Type	Width [min]	Area [mAU*s]	Height [mAU]	Area %
1	13.610	MM	0.3084	1666.60059	90.06596	12.2221
2	14.764	VB	0.3315	1.19694e4	561.92102	87.7779

Totals : 1.36360e4 651.98698

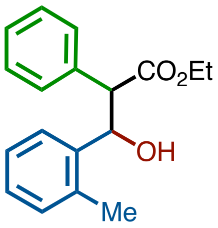

(*racemic-34*)

```
Sample Info      : C2_90:10Hex:IPA_1 ml/min_220 nm

Additional Info : Peak(s) manually integrated
```

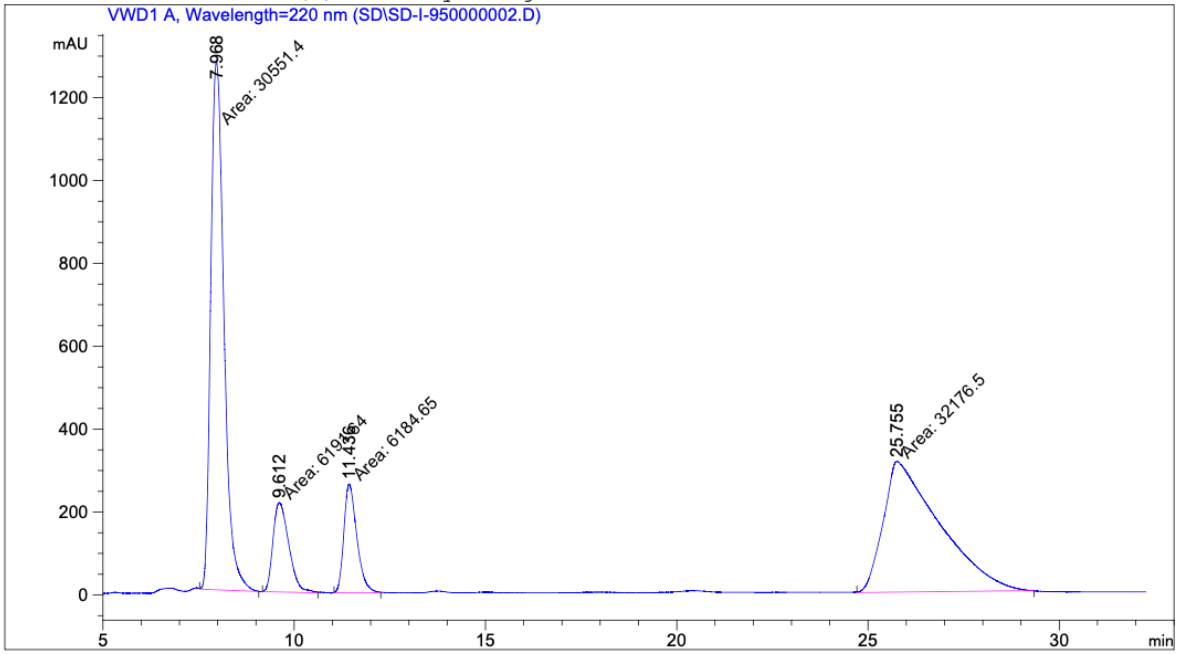


```
=====================================================================
                         Area Percent Report
=====================================================================

Sorted By              :      Signal
Multiplier:                    :      1.0000
Dilution:                      :      1.0000
Do not use Multiplier & Dilution Factor with ISTDs


Signal 1: VWD1 A, Wavelength=220 nm

Peak RetTime Type  Width     Area      Height     Area
  #   [min]        [min]   [mAU*s]     [mAU]        %
----|-------|----|-------|----------|----------|--------|
   1   7.968 MM    0.3989 3.05514e4 1276.51770  40.6787
   2   9.612 MM    0.4779 6191.64404  215.92795   8.2441
   3  11.436 MM    0.3931 6184.65039  262.20544   8.2348
   4  25.755 MM    1.7005 3.21765e4  315.37143  42.8424

Totals :                  7.51042e4 2070.02252
```

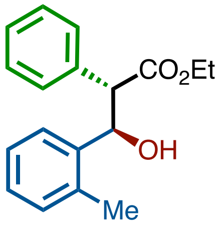

(*enantioenriched*-34)

```
Sample Info      : C2_90:10Hex:IPA_1 ml/min_220 nm

Additional Info : Peak(s) manually integrated
```

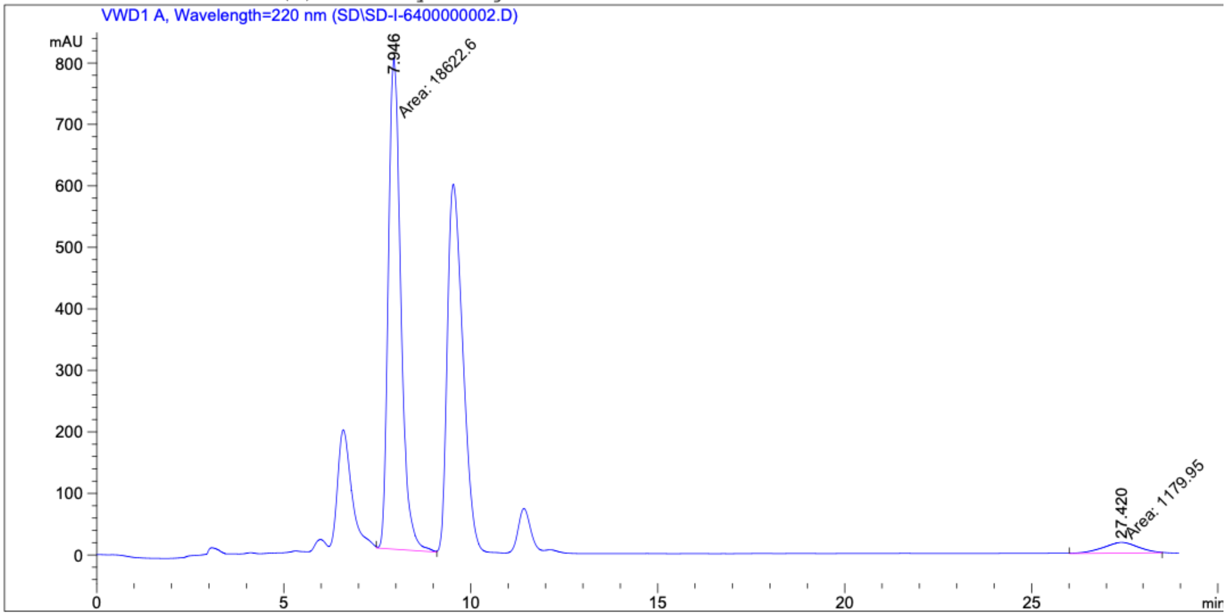


```
=====================================================================
                          Area Percent Report
=====================================================================

Sorted By             :      Signal
Multiplier:                    :       1.0000
Dilution:                      :       1.0000
Use Multiplier & Dilution Factor with ISTDs


Signal 1: VWD1 A, Wavelength=220 nm

Peak RetTime Type  Width     Area      Height     Area
  #   [min]        [min]   [mAU*s]     [mAU]        %
----|-------|----|-------|----------|----------|--------|
   1   7.946 MM    0.3883 1.86226e4   799.27350  94.0414
   2  27.420 MM    1.1209 1179.95129   17.54543   5.9586

Totals :                  1.98025e4   816.81893
```

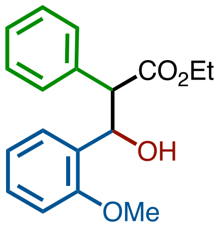

(*racemic-35*)

```
Sample Info      : IA-3_90:10_Hex:IPA;, 1mL/min, 220 nm

Additional Info : Peak(s) manually integrated
```

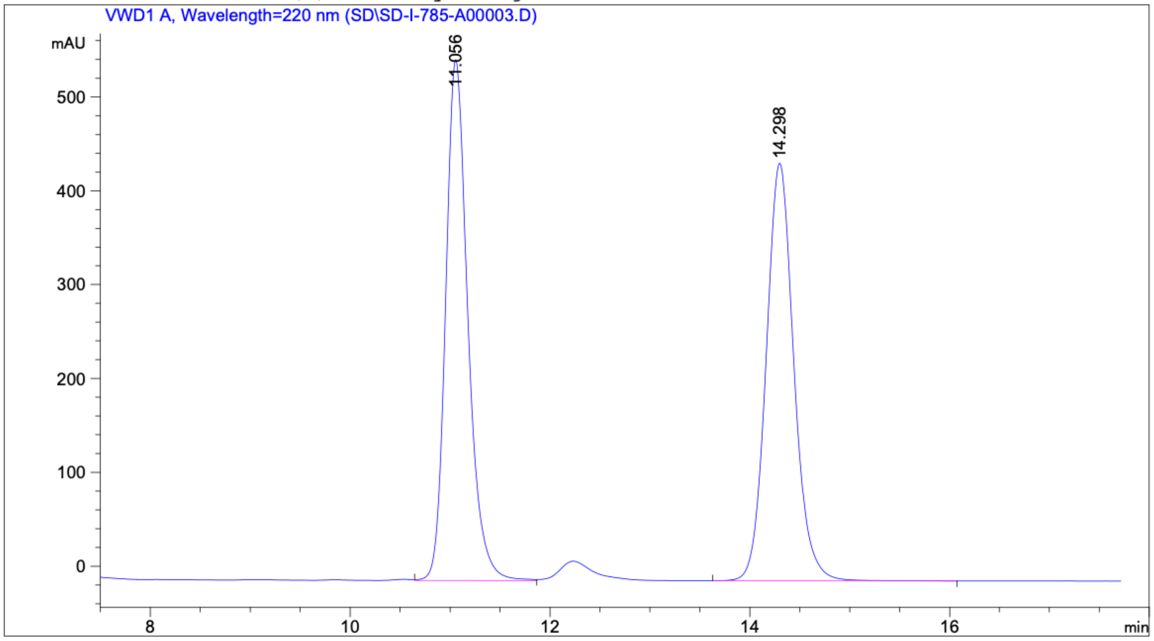


```
=====================================================================
                        Area Percent Report
=====================================================================

Sorted By              :      Signal
Multiplier:                    :      1.0000
Dilution:                      :      1.0000
Do not use Multiplier & Dilution Factor with ISTDs


Signal 1: VWD1 A, Wavelength=220 nm

Peak RetTime Type  Width     Area      Height     Area
  #    [min]        [min]   [mAU*s]     [mAU]       %
----|-------|----|-------|----------|----------|--------|
   1  11.056 VV    0.2338 8552.58398  554.80219  50.0228
   2  14.298 BB    0.2941 8544.77148  444.92685  49.9772

Totals :                  1.70974e4   999.72903
```

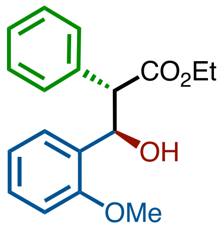

(*enantioenriched*-35)

```
Sample Info      : IA-3_90:10_Hex:IPA;, 1mL/min, 220 nm

Additional Info : Peak(s) manually integrated
```

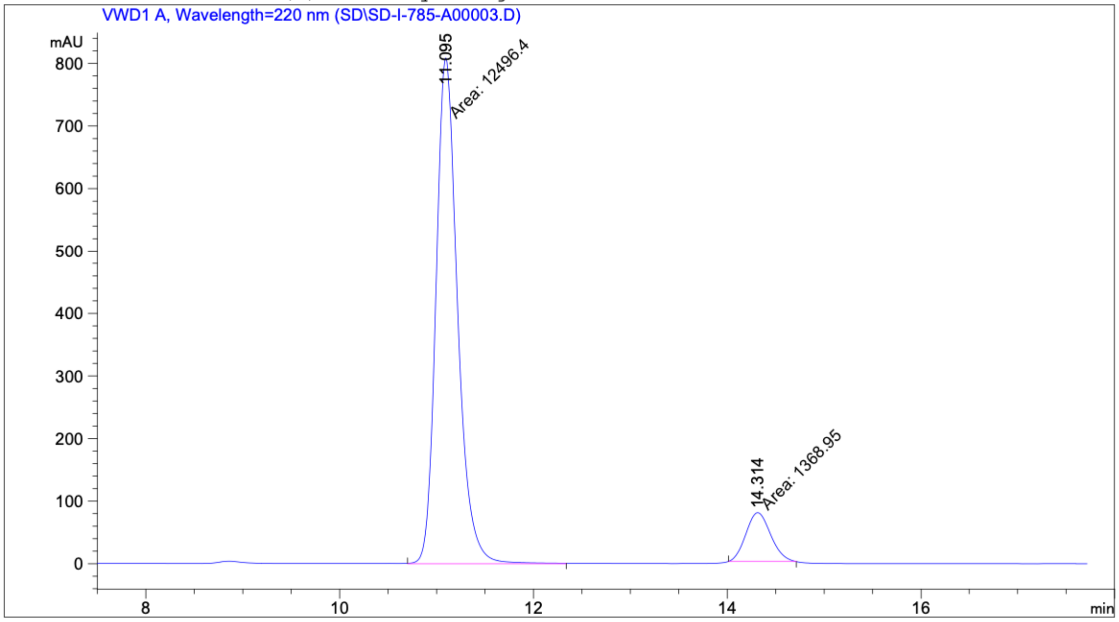


```
=====================================================================
                        Area Percent Report
=====================================================================

Sorted By              :      Signal
Multiplier:                   :      1.0000
Dilution:                     :      1.0000
Do not use Multiplier & Dilution Factor with ISTDs


Signal 1: VWD1 A, Wavelength=220 nm

Peak RetTime Type  Width     Area      Height     Area
  #   [min]        [min]   [mAU*s]     [mAU]        %
----|-------|----|-------|----------|----------|--------|
   1  11.095 MM    0.2576 1.24964e4   808.62738  90.1268
   2  14.314 MM    0.2940 1368.95471   77.60985   9.8732

Totals :                  1.38654e4   886.23723
```

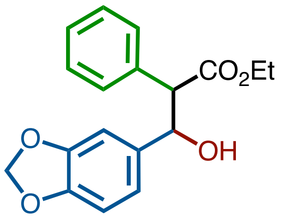

(*racemic-36)*

Sample Info : IA-3_85:15 Hex:IPA;, 1mL/min, 220 nm

Additional Info : Peak(s) manually integrated

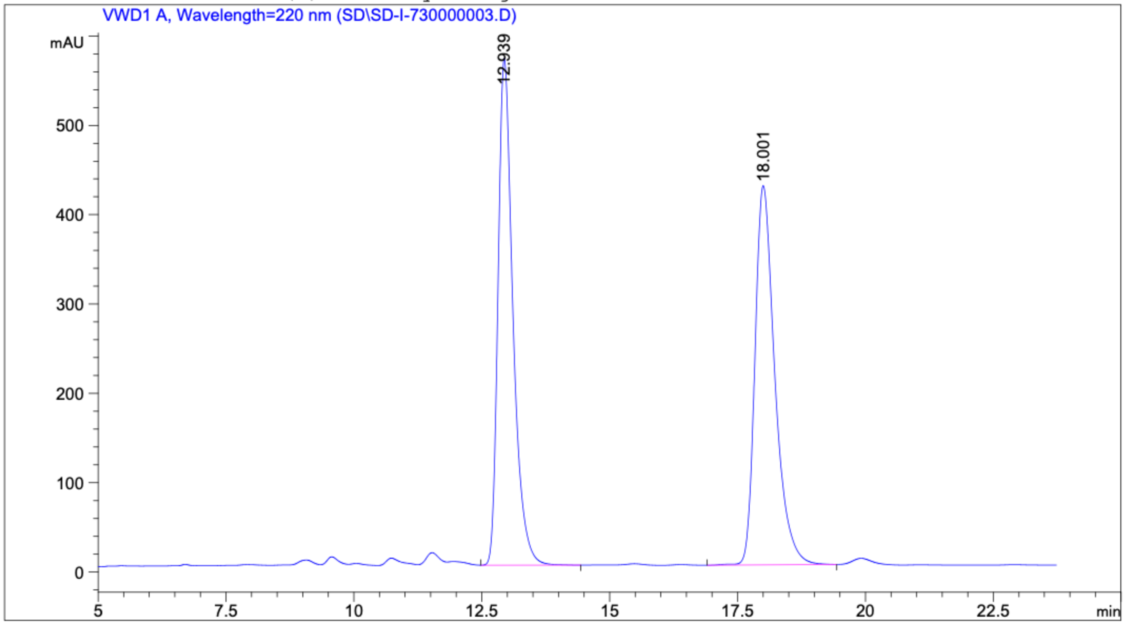

Area Percent Report

Sorted By : Signal
Multiplier: : 1.0000
Dilution: : 1.0000
Do not use Multiplier & Dilution Factor with ISTDs

Signal 1: VWD1 A, Wavelength=220 nm

Peak #	RetTime [min]	Type	Width [min]	Area [mAU*s]	Height [mAU]	Area %
1	12.939	BB	0.3048	1.14681e4	567.42523	50.0213
2	18.001	BB	0.4091	1.14584e4	424.25137	49.9787

Totals : 2.29265e4 991.67661

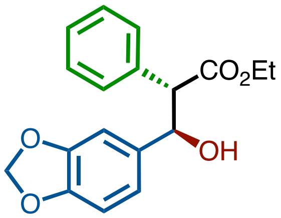

(*enantioenriched*-36)

Sample Info : IA-3_85:15 Hex:IPA;, 1mL/min, 220 nm

Additional Info : Peak(s) manually integrated

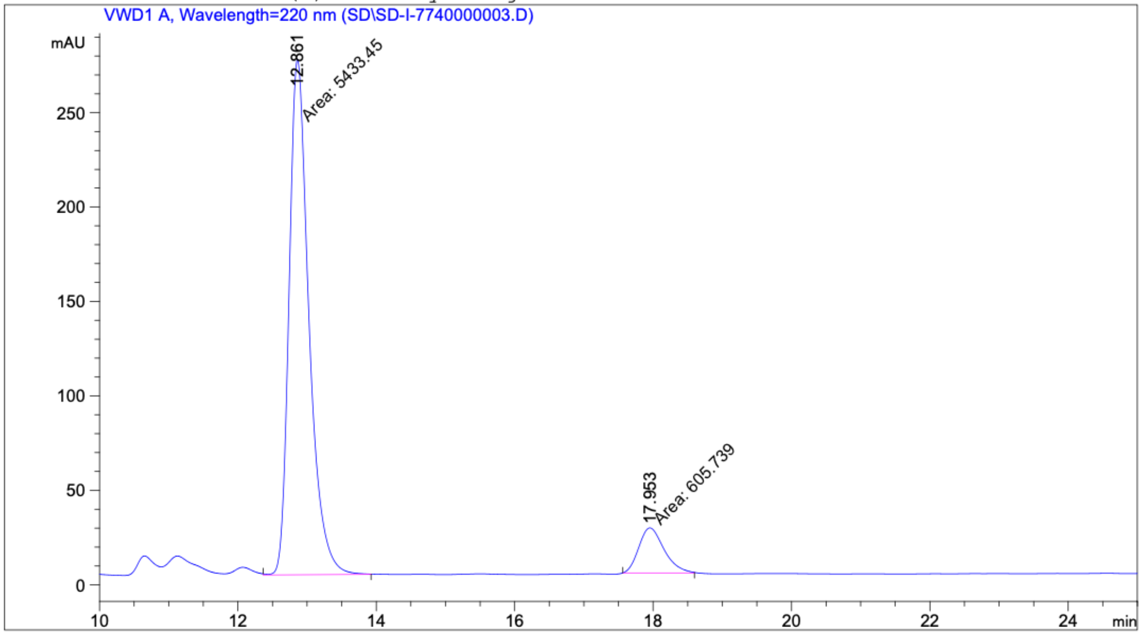

Area Percent Report

Sorted By : Signal
Multiplier: : 1.0000
Dilution: : 1.0000
Do not use Multiplier & Dilution Factor with ISTDs

Signal 1: VWD1 A, Wavelength=220 nm

Peak #	RetTime [min]	Type	Width [min]	Area [mAU*s]	Height [mAU]	Area %
1	12.861	MM	0.3318	5433.44971	272.94089	89.9699
2	17.953	MM	0.4234	605.73853	23.84590	10.0301
Totals :				6039.18823	296.78679	

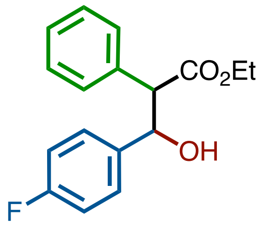

(*racemic-37*)

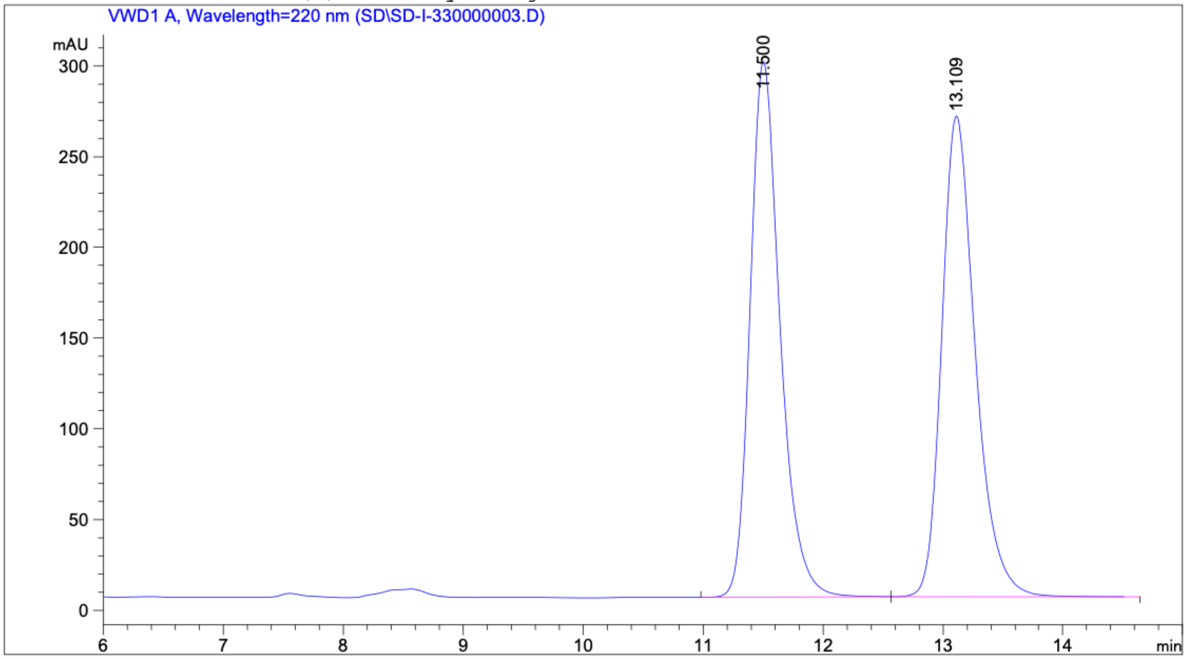


```
Sample Info     : IA-3_90:10  Hex:IPA;, 1mL/min, 220 nm

Additional Info : Peak(s) manually integrated
```

```
=====================================================================
                         Area Percent Report
=====================================================================

Sorted By             :      Signal
Multiplier:                   :      1.0000
Dilution:                     :      1.0000
Do not use Multiplier & Dilution Factor with ISTDs


Signal 1: VWD1 A, Wavelength=220 nm

Peak RetTime Type  Width     Area      Height     Area
  #   [min]        [min]   [mAU*s]     [mAU]        %
----|-------|----|-------|----------|----------|--------|
   1  11.500 BB    0.2643 5117.86621  295.08923  49.8821
   2  13.109 BB    0.2955 5142.06006  264.81903  50.1179

Totals :                  1.02599e4   559.90826
```

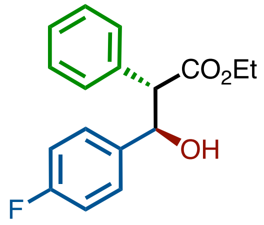

(*enantioenriched*-37)

Sample Info : IA-3_90:10 Hex:IPA;, 1mL/min, 220 nm

Additional Info : Peak(s) manually integrated

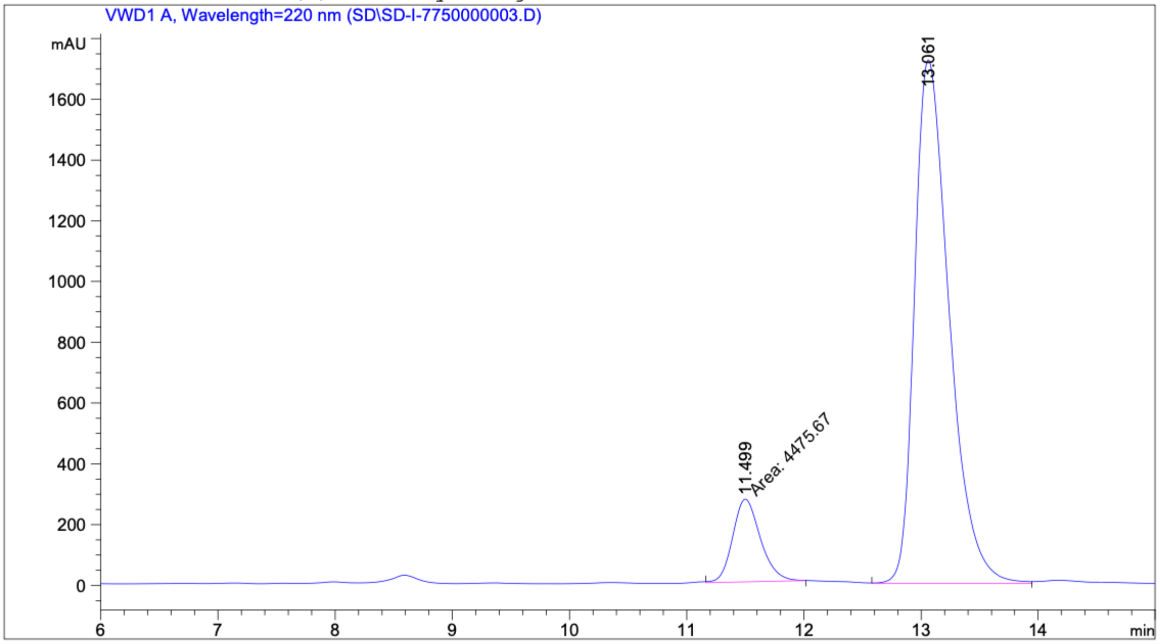

===

Area Percent Report

===

Sorted By : Signal
Multiplier: : 1.0000
Dilution: : 1.0000
Do not use Multiplier & Dilution Factor with ISTDs

Signal 1: VWD1 A, Wavelength=220 nm

Peak #	RetTime [min]	Type	Width [min]	Area [mAU*s]	Height [mAU]	Area %
1	11.499	MM	0.2745	4475.67383	271.77173	11.2268
2	13.061	VV	0.3157	3.53903e4	1722.71863	88.7732

Totals : 3.98660e4 1994.49036

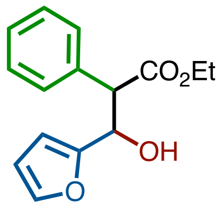

(*racemic-38*)

Sample Info : C1_90:10 Hex:IPA; 1mL/min, 220 nm

Additional Info : Peak(s) manually integrated

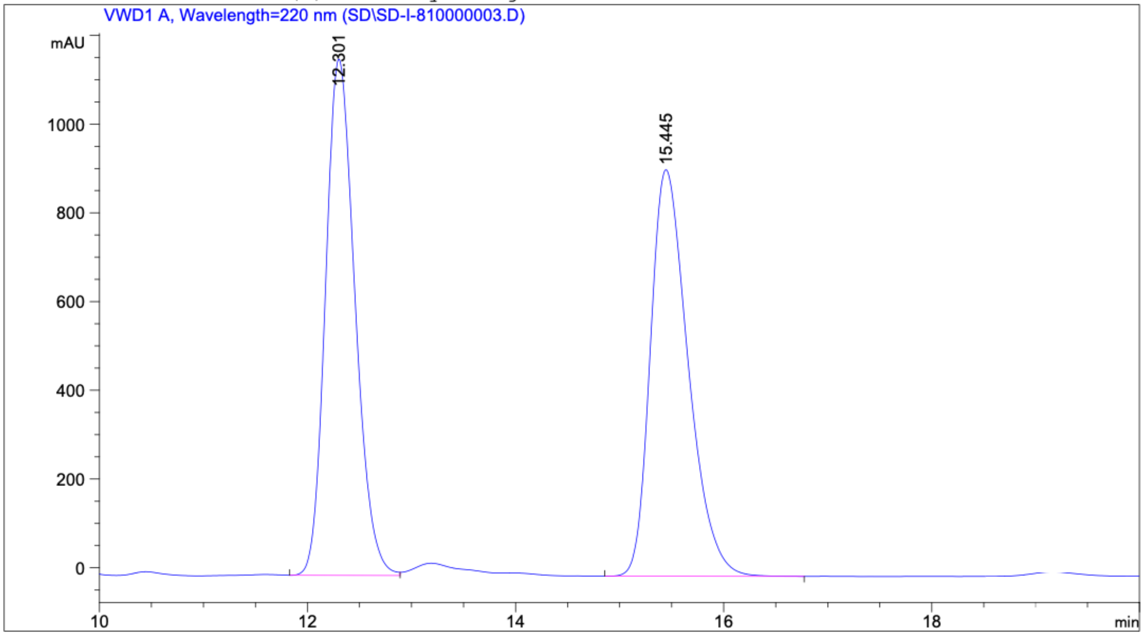

===

Area Percent Report

===

Sorted By : Signal
Multiplier: : 1.0000
Dilution: : 1.0000
Do not use Multiplier & Dilution Factor with ISTDs

Signal 1: VWD1 A, Wavelength=220 nm

Peak #	RetTime [min]	Type	Width [min]	Area [mAU*s]	Height [mAU]	Area %
1	12.301	BV	0.3047	2.29036e4	1163.44397	49.8066
2	15.445	BB	0.3879	2.30814e4	916.33984	50.1934

Totals : 4.59850e4 2079.78381

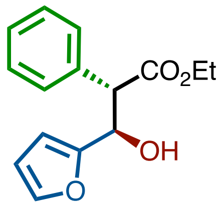

(*enantioenriched*-38)

```
Sample Info     : IA_90:10  Hex:IPA; 1mL/min, 220 nm

Additional Info : Peak(s) manually integrated
```

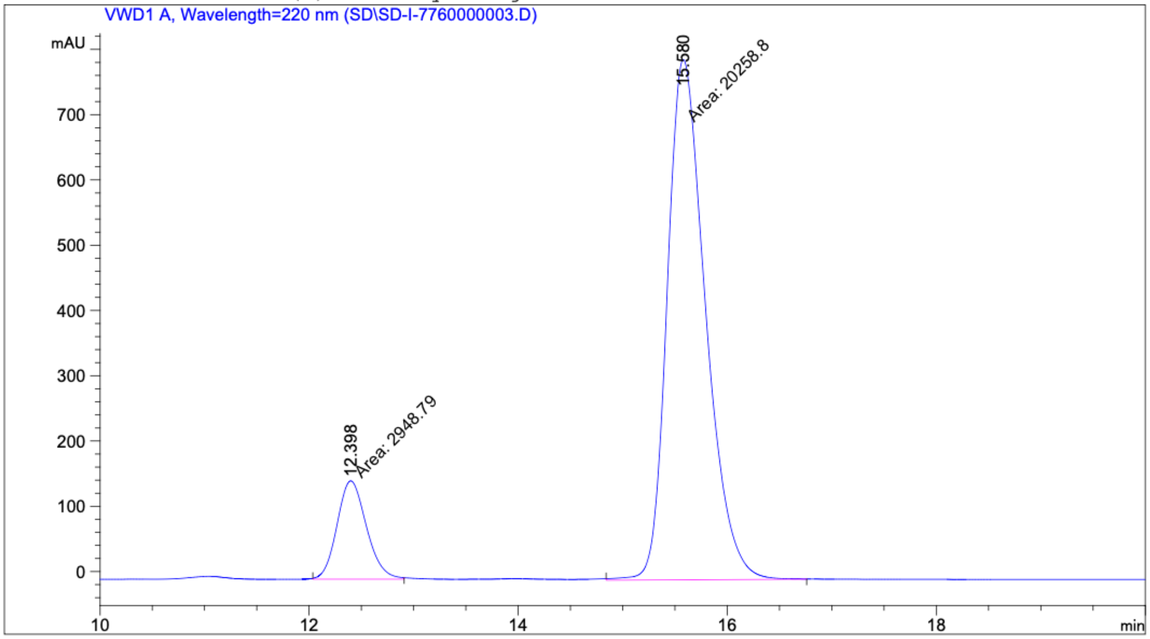


```
=====================================================================
                         Area Percent Report
=====================================================================

Sorted By             :      Signal
Multiplier:                   :      1.0000
Dilution:                     :      1.0000
Do not use Multiplier & Dilution Factor with ISTDs


Signal 1: VWD1 A, Wavelength=220 nm

Peak RetTime Type  Width     Area      Height     Area
  #   [min]        [min]   [mAU*s]     [mAU]        %
----|-------|----|-------|----------|----------|--------|
   1  12.398 MM    0.3262 2948.79150  150.66818  12.7061
   2  15.580 MM    0.4236 2.02588e4   797.12970  87.2939

Totals :                  2.32076e4   947.79788
```

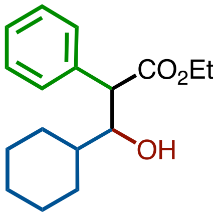

(*racemic-39*)

[illegible]

Sample Info : IA-3_90:10_Hex:IPA;, 1 mL/min, 220 nm

Additional Info : Peak(s) manually integrated

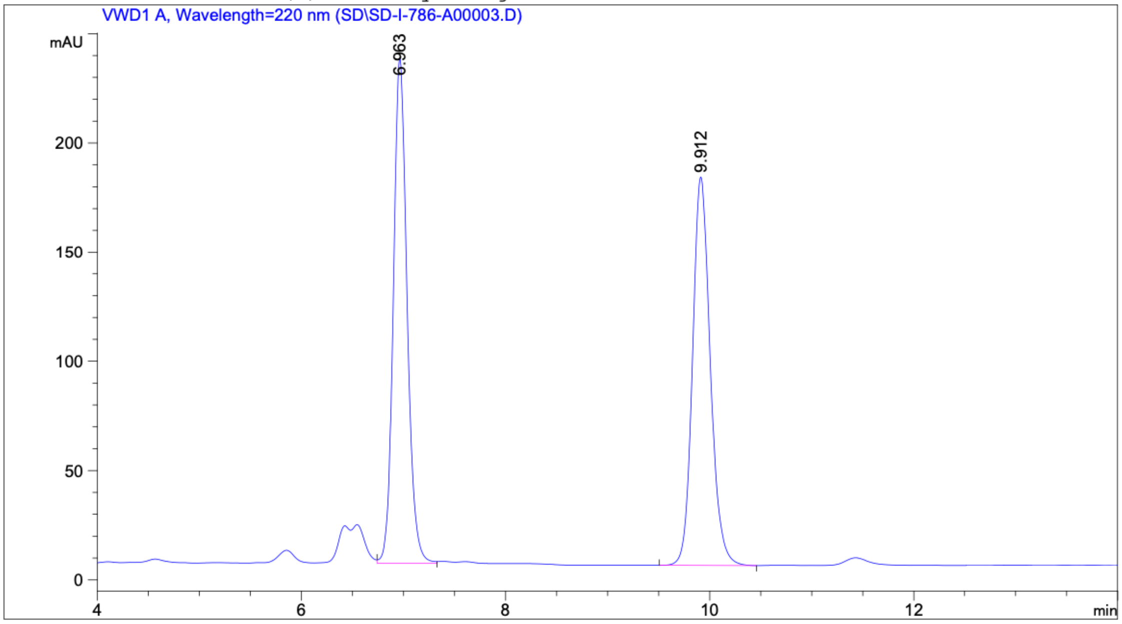

===

Area Percent Report

===

Sorted By : Signal
Multiplier: : 1.0000
Dilution: : 1.0000
Do not use Multiplier & Dilution Factor with ISTDs

Signal 1: VWD1 A, Wavelength=220 nm

Peak #	RetTime [min]	Type	Width [min]	Area [mAU*s]	Height [mAU]	Area %
1	6.963	VV	0.1419	2156.88574	231.13289	50.0949
2	9.912	BB	0.1850	2148.71436	177.80222	49.9051

Totals : 4305.60010 408.93510

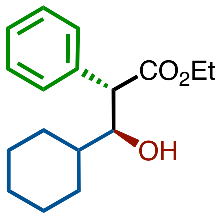

(*enantioenriched*-39)

[illegible]

Sample Info : IA-3_90:10_Hex:IPA;, 1 mL/min, 220 nm

Additional Info : Peak(s) manually integrated

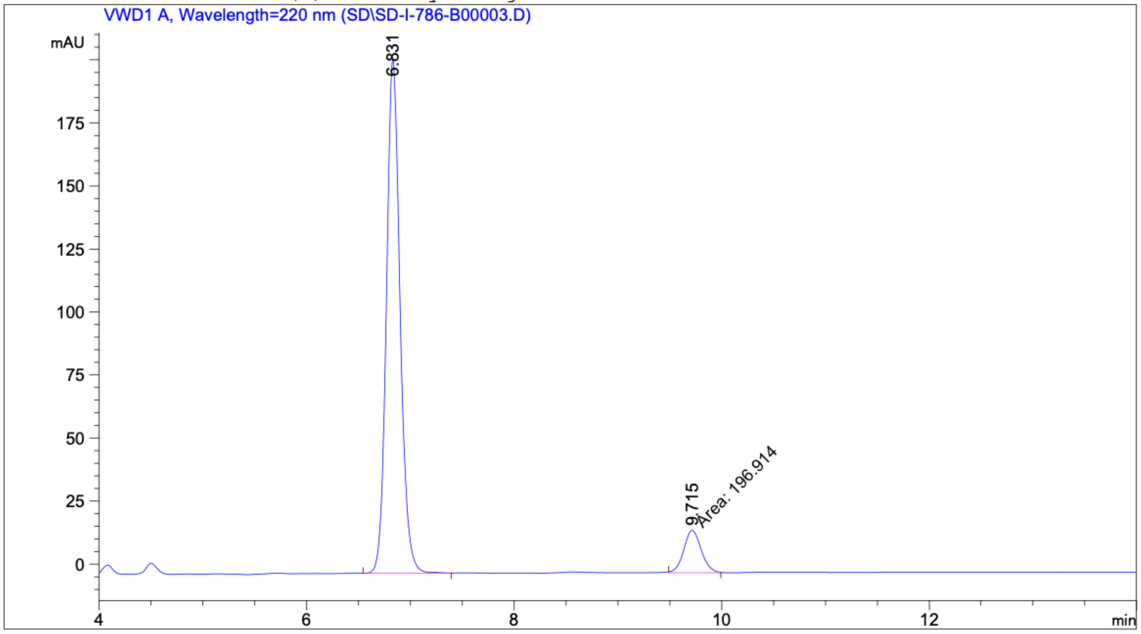

===

Area Percent Report

===

Sorted By : Signal
Multiplier: : 1.0000
Dilution: : 1.0000
Do not use Multiplier & Dilution Factor with ISTDs

Signal 1: VWD1 A, Wavelength=220 nm

Peak #	RetTime [min]	Type	Width [min]	Area [mAU*s]	Height [mAU]	Area %
1	6.831	BB	0.1395	1860.38708	203.87146	90.4285
2	9.715	MM	0.1952	196.91351	16.80945	9.5715
Totals :				2057.30060	220.68091	

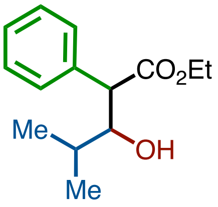

(*racemic-40*)

Sample Info   : IA_95:5_Hex:IPA_0.5 mL/min_220 nm

Additional Info : Peak(s) manually integrated

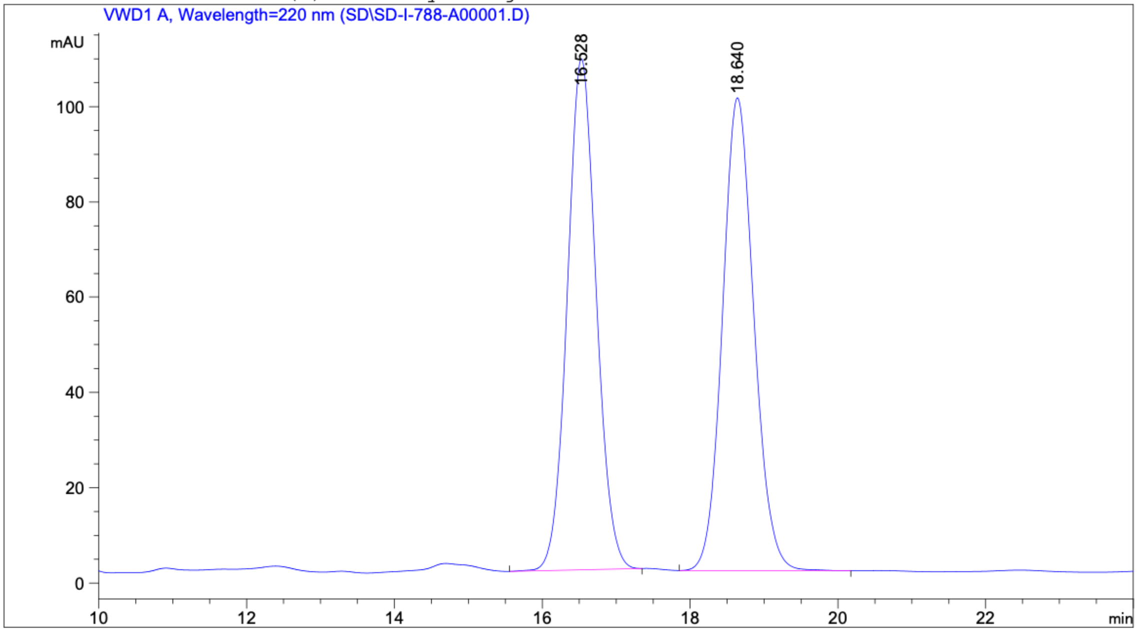

===

Area Percent Report

===

Sorted By : Signal
Multiplier: : 1.0000
Dilution: : 1.0000
Do not use Multiplier & Dilution Factor with ISTDs

Signal 1: VWD1 A, Wavelength=220 nm

Peak #	RetTime [min]	Type	Width [min]	Area [mAU*s]	Height [mAU]	Area %
1	16.528	BB	0.4191	2914.95703	107.23109	49.8617
2	18.640	BB	0.4557	2931.12964	99.21265	50.1383

Totals : 5846.08667 206.44374

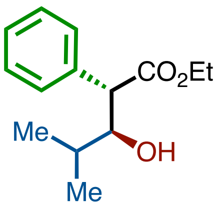

(*enantioenriched*-40)

Sample Info : IA_95:5_Hex:IPA_0.5 mL/min_220 nm

Additional Info : Peak(s) manually integrated

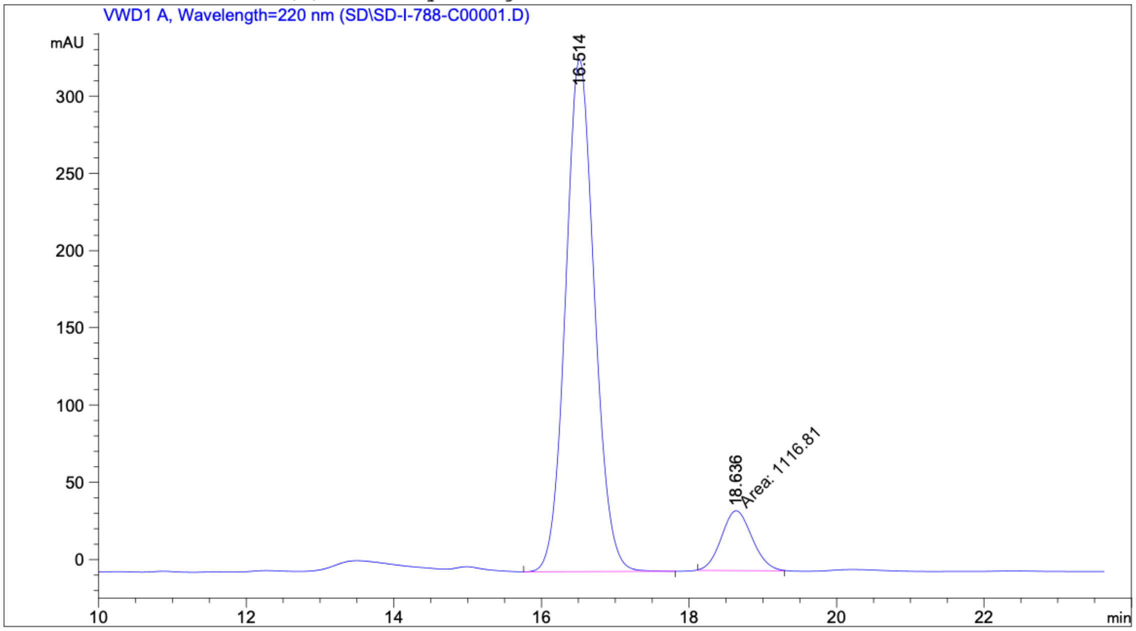

===

Area Percent Report

===

Sorted By : Signal
Multiplier: : 1.0000
Dilution: : 1.0000
Do not use Multiplier & Dilution Factor with ISTDs

Signal 1: VWD1 A, Wavelength=220 nm

Peak #	RetTime [min]	Type	Width [min]	Area [mAU*s]	Height [mAU]	Area %
1	16.514	BB	0.4198	9037.92188	331.75098	89.0021
2	18.636	MM	0.4815	1116.81079	38.65348	10.9979

Totals : 1.01547e4 370.40446

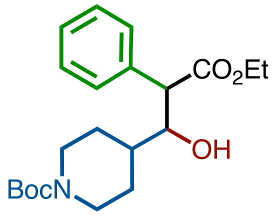

(*racemic-41*)

```
Sample Info     : C2_Hex:IPA_90:10_1ml/min_220nm

Additional Info : Peak(s) manually integrated
```

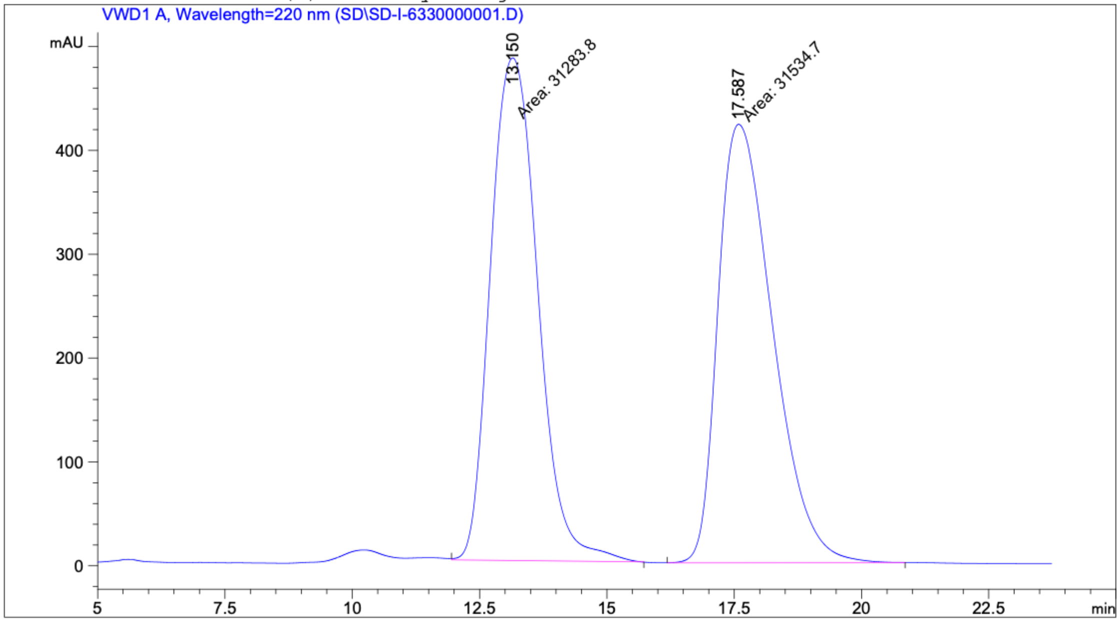


```
=====================================================================
                         Area Percent Report
=====================================================================

Sorted By             :      Signal
Multiplier:                    :      1.0000
Dilution:                      :      1.0000
Do not use Multiplier & Dilution Factor with ISTDs


Signal 1: VWD1 A, Wavelength=220 nm

Peak RetTime Type  Width     Area      Height     Area
  #   [min]        [min]   [mAU*s]     [mAU]        %
----|-------|----|-------|----------|----------|--------|
   1  13.150 MM    1.0775 3.12838e4  483.89966  49.8003
   2  17.587 MM    1.2446 3.15347e4  422.28128  50.1997

Totals :                  6.28185e4  906.18094
```

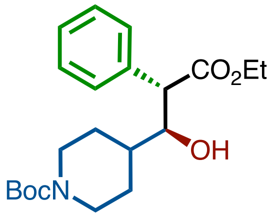

(*enantioenriched*-41)

[illegible]

```
Sample Info      : Cel-2 95:5 Hex:IPA 1.0 mL/min 254 nm


Additional Info : Peak(s) manually integrated
```

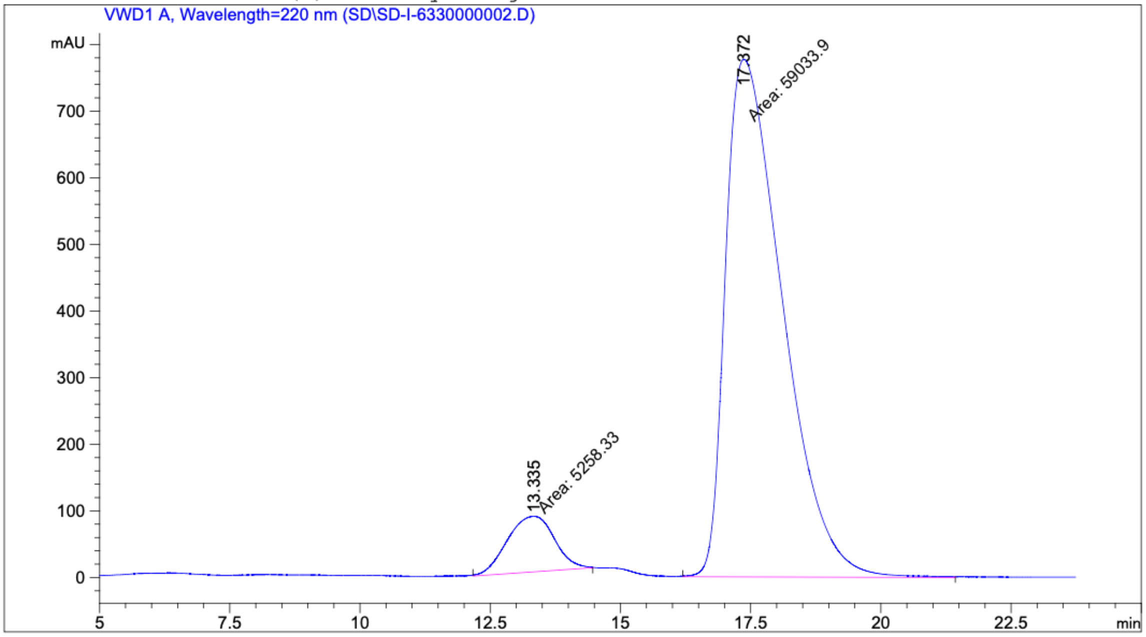


```
=====================================================================
                          Area Percent Report
=====================================================================

Sorted By             :      Signal
Multiplier:                   :      1.0000
Dilution:                     :      1.0000
Do not use Multiplier & Dilution Factor with ISTDs


Signal 1: VWD1 A, Wavelength=220 nm

Peak RetTime Type  Width     Area      Height     Area
  #   [min]        [min]   [mAU*s]     [mAU]        %
----|-------|----|-------|----------|----------|--------|
   1  13.335 MM    1.0531 5258.33057   83.21893   8.1788
   2  17.372 MM    1.2663 5.90339e4   776.97772  91.8212

Totals :                  6.42922e4   860.19666
```

13. Reference:

[1] 1 J. Huguet Clotet, L. Ozores Viturro, S. Rodriguez Ropero, P. Dalmases Barjoan, WO 2018115362, June 28, 2018.
[2] S. Diez-Gonzalez, E. C. Escudero-Adan, J. Benet-Buchholz, E. D. Stevens, A. M. Z. Slawinc, S. P. Nolan, *Dalton Trans.* **2010**, *39,* 7595
[3] J. K. Park, H. H. Lackey, M. D. Rexford, K. Kovnir, M. Shatruk, T. D. McQuade, *Org. Lett.* **2010**, *12,* 5008-5011.
[4] J. K. Park, H. H. Lackey, B. A. Ondrusek, T. D. McQuade, *J. Am. Chem. Soc.* **2011**, *133*, 2410–2413
[5] N. C. Bruno, M. T. Tudge, S. L. Buchwald, Chem. *Sci.***,** **2013**,***4***, 916-920
[6] E. Einaru, K. Shitamichi, *Angew. Chem. Int. Ed.* **2018,** *57,* 13863 –13867
[7] D. Rozsar, *Angew. Chem. Int. Ed*. **2023,** *62,* e202303391
[8] A. Kaga, H. Hayashi, *Angew. Chem. Int. Ed.* **2017,** *56*, 11807 –11811
[9] S. Xing, C. Ma, W. Liu, S. Ni, D. Zhu, *, Xu, L., Shao, X. *Org. Lett.* **2023**, *25*, 1066−1071
[10] Eli Lilly and Company *World Intellectual Property Organization*, WO9908699 A1 1999-02-25
[11] United States Dept. of Health and Human Services, United States, US6307090 B1 2001-10-23
[12] T. Jiang, T. Livinghouse, H. M**.** Lovick, *Chem. Commun*., **2011**, *47*, 12861-12863
[13] T. N. T. Nguyen, N. O. Thiell, F. Pape, J. F. Teichert, *Org. Lett.* **2016**, *18*, 10, 2455–2458
[14] R. Lui, Z. Yang, Y. Ni, K. Song, K. Shen, S. Lin, Q. Pan, *J. Org. Chem.* **2017**, *82*, 15, 8023–8030
[15] M. Yoshida, H. Otaka, T. Doi, *Eur. J. Org. Chem*. **2014**, 6010–6016
[16] F. Burg, T. Rovis, *J. Am. Chem. Soc.* **2021**, *143*, 43, 17964–17969
[17] Syngenta Crop Protection AG World Intellectual Property Organization, WO2021009229 A1 2021-0121
[18] Esfam Biotech Pty Ltd.World Intellectual Property Organization, WO2022020888 A1 2022-02-03
[19] C. Prandi, E. G. Oochiato, S. Tabasso, P. Bonfante, M. Novero, D. Scarpi, M. E. Bova, I. Milett, *Eur. J. Org. Chem.* **2011**, 20-21, 3781–3793
[20] Busacca, C. A.; Lorenz, J. C.; Grinberg, N.; Haddad, N.; Lee, H.; Li, Z.; Liang, M.; Reeves, D.; Saha, A.; Varsolona. R.; Senanayake, C. H. *Org. Lett*., **2008**, *10*, 341.
[21] J. K. Park, H. H. Lackey, M. D. Rexford, K. Kovnir, M. Shatruk, T. D. McQuade, *Org. Lett*. **2010**, *12,* 5008-5011.
[22] K. M. Logan and M. K. Brown *Angew. Chem. Int. Ed.* **2017**, *56*, 851-855.
[23] A. K. Simlandy, M. Lyu, M. K. Brown, *ACS Catal.* **2021,** *11*, 20, 12815–12820
[24] G. L. Trammel, R. Kuniyil, P. F. Crook, P. Liu, M. K. Brown, *J. Am. Chem. Soc.* **2021**, *143*, 40, 16502–16511
[25] H. Xie, B. Breit, *ACS Catal*. **2022**, *12*, 5, 3249–3255
[26] K. Toribatake, L. Zhou, A. Tsuruta and H. Nishiyama Tetrahedron **2013**, *69*, 3551e3560
